# Genetic diversity of late Neanderthals in northwestern Europe

**DOI:** 10.1038/s41586-026-10625-1

**Published:** 2026-06-24

**Authors:** Alba Bossoms Mesa, Elena Essel, Stéphane Peyrégne, Arev P. Sümer, Leonardo N. M. Iasi, Christian Heide, Divyaratan Popli, Cesare de Filippo, Marie-Theres Gansauge, Lars Gerullat, Laurin Lippik, Sarah Nagel, Birgit Nickel, Barbara Schellbach, Anna Schmidt, Johann Visagie, Antje Weihmann, Hugo Zeberg, Julia Zorn, Hélène Rougier, Isabelle Crevecoeur, Patrick Semal, Grégory Abrams, Thibaut Devièse, Stéphane Pirson, Kévin Di Modica, Pierre Cattelain, Christelle Draily, Michel Toussaint, Isabelle De Groote, Frido Welker, Cosimo Posth, Marie Soressi, Jean-Jacques Hublin, Johannes Krause, Svante Pääbo, Matthias Meyer, Janet Kelso, Benjamin M. Peter, Mateja Hajdinjak

**Affiliations:** 1 Max Planck Institute for Evolutionary Anthropology, Leipzig, Germany; 2 Genewiz Germany, Leipzig, Germany; 3 Karolinska Institutet, Stockholm, Sweden; 4 California State University, Northridge, Northridge, CA USA; 5 PACEA, UMR 5199, Université de Bordeaux, CNRS, Ministère de la Culture, Pessac, France; 6 Royal Belgian Institute of Natural Sciences, Brussels, Belgium; 7 ArcheOs - Laboratory for Biological Anthropology, Department of Archaeology, Ghent University, Ghent, Belgium; 8 Research Foundation - Flanders (FWO), Brussels, Belgium; 9 CEREGE, Aix-Marseille University, CNRS, IRD, INRAE, Collège de France, Aix-en-Provence, France; 10 Agence wallonne du Patrimoine, Namur, Belgium; 11 Centre européen d’archéométrie and Department of Geology, Liège University, Liège, Belgium; 12 Scladina Cave Archaeological Centre, Espace Muséal d’Andenne, Andenne, Belgium; 13 ASBL Centre d’Études et de Documentation Archéologiques, Musée du Malgré-Tout, Treignes, Belgium; 14 Université Libre de Bruxelles, Brussels, Belgium; 15 Globe Institute, University of Copenhagen, Copenhagen, Denmark; 16 University of Tübingen, Tübingen, Germany; 17 Senckenberg Centre for Human Evolution and Palaeoenvironment, Tübingen, Germany; 18 Leiden University, Leiden, The Netherlands; 19 CIRB (UMR 7241–U1050), Collège de France, Paris, France; 20 Department of Ecology and Evolutionary Biology, Institute for Quantitative and Computational Biosciences, University of California, Los Angeles, Los Angeles, CA USA

**Keywords:** Population genetics, Archaeology, Anthropology

## Abstract

Archaeological, osteological and genetic evidence suggests that Neanderthals lived in small groups^1,2^; however, less is known about whether these groups were part of isolated communities or belonged to larger, well-connected populations^3^. The dense concentration of broadly contemporaneous Neanderthal sites in the Meuse Basin, Belgium^4^, provides a rare opportunity to study regional populations at high resolution. Here we generated genetic data from 27 Neanderthals who lived less than approximately 52,500 years ago from ten archaeological sites in Belgium and France, including a high-coverage genome from a 45,000-year-old individual from Goyet, Belgium. We show that most of these individuals are more closely related to one another than to other contemporaneous late Neanderthals in Europe. Further, some of these individuals carry DNA from a Neanderthal lineage predating the split of late Neanderthals. Although these Neanderthals overlapped temporally with early modern humans in northwestern Europe from around 47,000 years ago, we find no evidence of recent gene flow from modern humans. They also do not show the genetic signatures of mating among close relatives found in Altai Neanderthals, suggesting that they lived in larger or better-connected groups. Moreover, genetic load did not accumulate over time, arguing against progressive genetic deterioration as a driver of Neanderthal extinction.

## Abstract (web summary)

Genetic sequencing of multiple late Neanderthals living less than 52,500 years ago provides an overview of genetic diversity and demonstrates that even low-coverage nuclear genome data can increase resolution of within-Neanderthal diversity.

## Main

Neanderthals lived in Europe and western Asia from at least around 430,000 years ago^5,6^ until around 40,000 years before present^7^ (430–40 kyr bp). The high-quality nuclear genomes of four Neanderthals^3,8–10^ provided numerous insights into the diversity and population history of Neanderthals and their interactions with early modern humans. The more recent Vindija 33.19 Neanderthal from Croatia (approximately 45 kyr bp) shows higher genetic diversity and less evidence of recent inbreeding^3^ than do the older Neanderthals from Denisova and Chagyrskaya caves (Altai Neanderthal (Denisova 5 or D5), approximately 120 kyr bp^8^; Denisova 17 (D17), approximately 110 kyr bp^10^; and Chagyrskaya 8, approximately 60 kyr bp^9^) who lived in the easternmost part of the known Neanderthal range. Further lower-coverage nuclear genomes from four late Neanderthals suggest close genetic affinities between individuals from geographically distant regions, such as Mezmaiskaya (Caucasus, Russia) and Les Cottés (Poitou, France), indicating possible long-range connectivity between late Neanderthals^11^.

Pleistocene remains are rare with generally low DNA preservation^12^, but recent methodological advances—including microsampling^11^, improved decontamination, extraction, library preparation^13–15^ and enrichment methods^1,16^—have improved the recovery of degraded DNA, enabling studies of larger numbers of Neanderthal remains. The low-coverage genomes of 11 Neanderthals from Chagyrskaya Cave revealed patterns of close kinship and low genetic diversity^1^. However, because these observations are based on a single group at the eastern periphery of the Neanderthal range, the extent to which such findings are representative of Neanderthal diversity and population structure in general remains open.

## A new fine-scale dataset

**Fig. 1:**
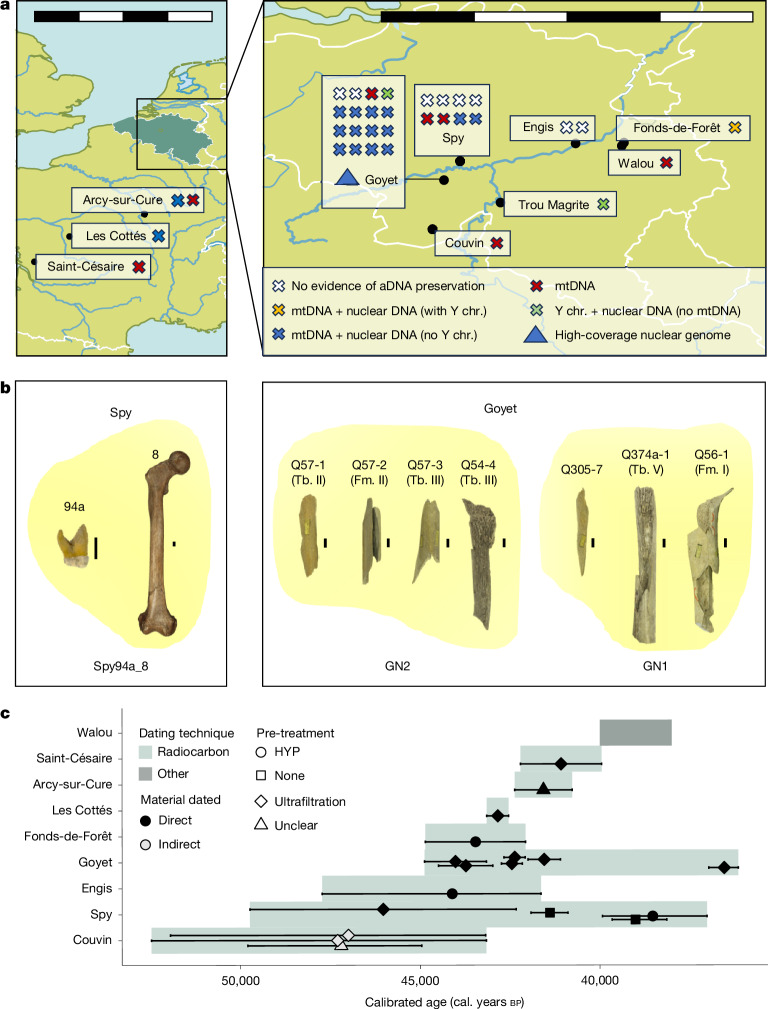
Temporal and geographical overview of the analysed Neanderthal remains. **a**, Map of the studied archaeological sites. For all sites, each cross represents a sample, coloured according to the type of genetic data generated. **b**, Skeletal remains identified as originating from the same individual (clusters are shown in yellow and individual numbers are provided at the bottom). **c**, Estimated ages for the studied Neanderthals. These are either direct radiocarbon dates, indirect dates using other bones from the same layer (Couvin) or a combination of other dating techniques (Walou). Radiocarbon ages (*n*(radiocarbon dates) = 18) are shown as means with the whiskers corresponding to ±95% probability intervals of the calibrated values (radiocarbon dates calibrated with IntCal20 (ref. ^58^) in OxCal 4.4 (ref. ^59^)). We excluded Trou Magrite because of insufficient data. Scale bars, each segment 100 km (**a**), 50 km (**a**, inset), 1 cm (**b**). aDNA, ancient DNA; Fm., femur; HYP, hydroxyproline; Tb., tibia; Y chr., Y chromosome. Maps in panel **a** made with Natural Earth (https://www.naturalearthdata.com/). Bone pictures in panel **b** of Spy 94a and Spy 8 reproduced from E. Dewamme and copyright Royal Belgian Institute of Natural Sciences (RBINS), CC BY 4.0. Other bone pictures in panel **b** adapted from refs. ^21,25^ under a Creative Commons licence, CC BY 4.0.

The Meuse Basin in Belgium offers a unique opportunity to study broadly contemporaneous late Neanderthals owing to its high concentration of Middle Palaeolithic sites^17,18^ (Fig. 1 and Supplementary Information section 1). Most of these sites were excavated during the nineteenth century, complicating the secure association with lithic assemblages and making their reliable contextualization more challenging^4^. Recent efforts, including reanalysis of faunal collections, identified further Neanderthal remains^19–22^ resulting in 14 new Neanderthal mitochondrial genomes from the region (including from Troisième Caverne of Goyet, Spy, Fonds-de-Forêt, Scladina and Trou Magrite)^4,11,21,23,24^ and three low-coverage nuclear genomes of Neanderthals from Scladina (I4-A)^23^, Spy (94a) and Goyet (Q56-1 (ref. ^11^)), which showed that the Spy 94a and Goyet Q56-1 individuals share significantly more derived alleles with each other than with any other late Neanderthal sequenced to date^11^.

However, key aspects of Neanderthal population structure in this region remain unexplored. Morphology-based analyses estimate that 80 Neanderthal remains from Spy and 101 remains from Goyet belonged to at least three and six individuals, respectively^20,25^, but the exact numbers remain unclear. Despite their genetic similarity, mortuary practices of the Neanderthals from Spy and Goyet are very different. At Spy, the anatomical connection between the bones suggests deliberate burial^26^, although this interpretation has been questioned^27^. By contrast, the Neanderthal remains at Goyet are highly fragmented, with anthropogenic modifications suggesting that the individuals were cannibalized^21^. Together with evidence for contrasting mobility patterns (sulfur isotope values suggest local foraging at Spy but non-local origins at Goyet)^28^, this points to greater diversity among late Neanderthals in northwestern Europe than is at present evident from genetic data.

Many of the sites in the Meuse Basin and surrounding areas have yielded Neanderthal remains dated to around 45 kyr bp or more recently^4^, a time when modern humans were already present in central Europe^29,30^. However, the radiocarbon dates of bone implements recovered from the archaeological sites in Belgium suggest that there was no overlap between modern humans and Neanderthals in this region^31^. Obtaining genetic data from both Neanderthals and modern humans could help to resolve this question, as direct evidence of recent mixture between them would establish an overlap.

## Individual characterization and kinship

We screened the skeletal remains of 34 late Neanderthals from seven sites in the Meuse Basin (Goyet, Spy, Couvin, Trou Magrite, Engis, Walou and Fonds-de-Forêt) and two sites in France (Saint-Césaire and Arcy-sur-Cure) for ancient DNA preservation (Fig. 1 and Extended Data Table 1). Their radiocarbon dates range from 52.48 kyr bp to 36.15 kyr bp; however, contamination with modern carbon could have biased some of the most-recent dates^21^. Shallow shotgun sequencing indicated that the proportion of endogenous DNA in most of the 175 DNA libraries was low, with a median of less than 1% of sequences mapping to the human reference genome (Supplementary Table 1.1). However, for Goyet Q56-1, this proportion was as high as 37%, which allowed us to sequence the genome of this Neanderthal to an average coverage of 22.4-fold—yielding the fifth high-coverage Neanderthal genome sequenced to date (Supplementary Table 1.2 and Supplementary Information section 2).

To generate genome-wide nuclear data from the less well-preserved specimens, we designed a hybridization capture panel (ArchaicPlus) that targets around 2.3 million single nucleotide polymorphisms (SNPs) informative about Neanderthal and Denisovan variation (Supplementary Information section 12). Using this panel, we recovered genome-wide nuclear data from 19 skeletal remains from six archaeological sites. All initial quality controls were performed at a library level. Owing to the high levels of present-day human DNA contamination, ranging from 0.4% (95% confidence interval (95% CI) 0.3–0.6%) to 90.3% (95% CI 85.5–95.2%) (Supplementary Table 2), we restricted all downstream analyses to sequences showing cytosine deamination, a characteristic of ancient DNA. This resulted in between 3,087 and 876,377 covered SNPs, with residual present-day human DNA contamination estimates ranging from 0% (95% CI 0–0.1%) to 20.7% (95% CI 6.9–44.4%) (Supplementary Table 2 and Supplementary Information section 13).

**Extended Data Fig. 1:**
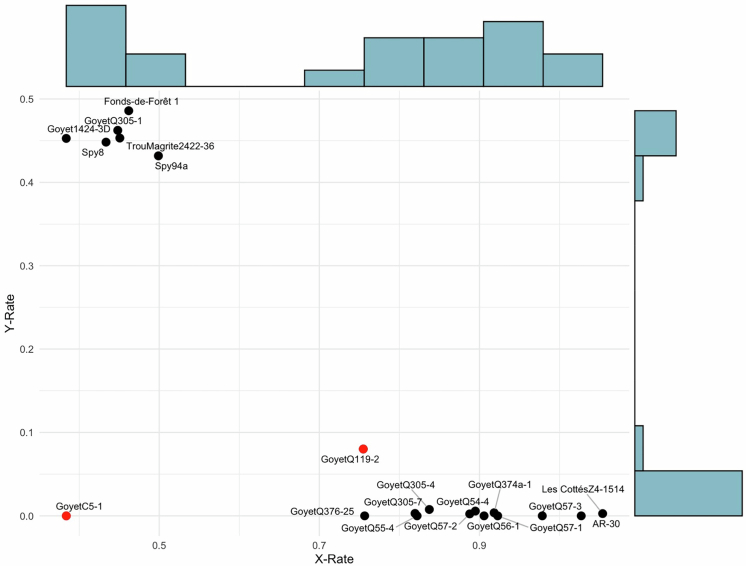
Sex determination. Average coverage ratio between the X-chromosome and the autosomes (X-axis), and the Y-chromosome and the autosomes (Y-axis). The marginal histograms (blue bars) on the top and right axes illustrate the frequency distribution of all samples along the X and Y axes, respectively. In three instances, we identified the following bones as originating from the same individual using kinship analysis (Supplementary Section 6): (1) Goyet Q56-1, Q374a-1, and Q305-7 belong to GN1, (2) Goyet Q57-1, Q57-2, and Q57-3 belong to GN2 and (3) Spy 94a and Spy 8 belong to Spy94a_8.

By comparing relative sequence coverage of the autosomes and the X chromosome (Extended Data Fig. 1 and Supplementary Information section 13), we determined that six Neanderthal remains were genetically male: the neonates Goyet Q305-1 and Trou Magrite 2422-36, the approximately 6.5–12.5-year-old child Goyet 1424-3D and three elements from mature individuals: Spy 94a, whose Y chromosome had been sequenced previously^1^, Fonds-de-Forêt 1 and Spy 8. The skeletal remains of Goyet Q119-2 and Goyet C5-1 had insufficient data for reliable sex determination, and the remaining 12 specimens were genetically female, including 10 remains representing at least four adults from Goyet, as well as Les Cottés Z4-1514 and Arcy-sur-Cure AR-30.

Next, we studied genetic relatedness among 15 skeletal remains with less than 5% contamination using KIN^32^ (Supplementary Information section 14). Among the nine specimens from Goyet, we identified two clusters of genetically identical remains: the Goyet Neanderthal 1 (GN1), composed of Goyet Q56-1, Q374a-1 and Q305-7; and the Goyet Neanderthal 2 (GN2), composed of Goyet Q57-1, Q57-2 and Q57-3 (Fig. 1b). Our analysis showed that the Goyet Q305-7 and Q374a-1 fragments, which were previously refitted to two different right tibias (Tibia III and Tibia V, respectively), were genetically identical. After re-evaluating their refitting, we concluded that Goyet Q305-7 had been wrongly refitted. We took an extra sample of Tibia III from the Goyet Q54-4 fragment, and found that it was genetically distinct from both Goyet Q374a-1 and Q305-7, and therefore did not belong to GN1. Instead, Goyet Q54-4 was genetically identical and morphologically consistent with the set of remains from GN2 (Supplementary Information section 14), demonstrating the potential of genetic analyses to inform the refitting of highly fragmented elements.

Similarly, we found that the nuclear data recovered from Spy 8, a right femur, were identical to the DNA previously sequenced from Spy 94a^11^, an upper third molar (log likelihood ratio = 2.8; Supplementary Information section 14). Thus, both could be attributed to the same adult male individual (Fig. 1b). We proceeded to merge the data assigned to the same individuals, and from this point onwards we refer to them as GN1, GN2 and Spy94a_8.

Apart from these matches, we found no close relatives among these Neanderthals up to third-degree relatedness—the detection limit of KIN (Supplementary Information section 14). At Goyet, all of the sequenced individuals were unrelated adult or adolescent female and juvenile male individuals. As it is unclear whether this assemblage represents a single community^21,25^, we refrain from making inferences about their group structure or culture. However, the fact that there is a neonate (Q305-1) whose mother is not represented among the analysed females is striking. Although it is possible that his mother remains unsampled, we find no evidence that any of the Goyet individuals were close relatives, which is in stark contrast to the Neanderthals from the Chagyrskaya Cave, where several closely related individuals were identified.

## Molecular dating of uniparental markers

**Fig. 2:**
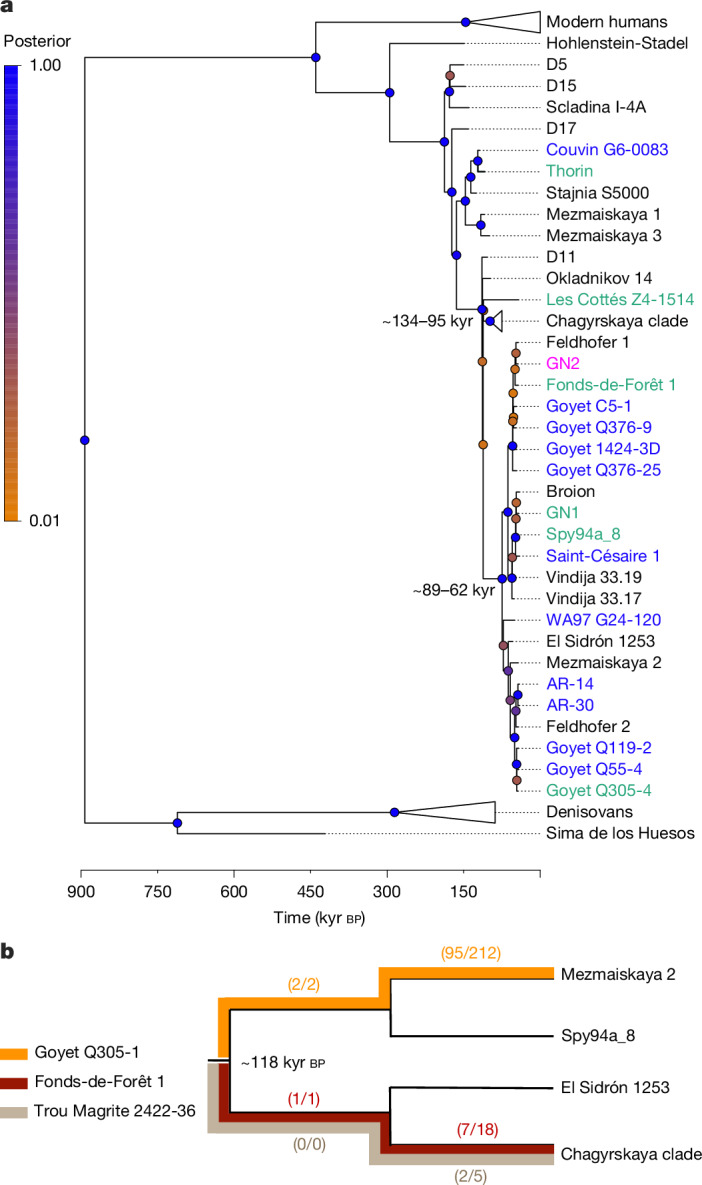
Phylogenies of uniparental markers. **a**, Bayesian mitochondrial tree reconstructed from new Neanderthal mtDNA genomes, as well as 28 previously published Neanderthals, 55 present-day humans, 10 ancient modern humans, the Sima de los Huesos hominin and 4 Denisovans using BEAST2 (v.2.1.3)^33^. Nodes are coloured based on their posterior probabilities. New mtDNA genomes are coloured in blue, mtDNA genomes that have been improved based on new data are in pink and previously published late Neanderthal mtDNA samples from the study region are in turquoise. **b**, Y chromosome cladogram including lineage assignment shown as coloured bars for the three newly sequenced male individuals (Goyet Q305-1, Trou Magrite 2422-36 and Fonds-de-Forêt 1). The numbers on each branch represent the number of shared derived alleles out of the total informative sites covered by sequences.

After mitochondrial DNA (mtDNA) enrichment and filtering for present-day human mtDNA contamination (Supplementary Information section 10 and Supplementary Table 3), we were able to reconstruct the mitochondrial genomes of 24 specimens from all sites except Engis and Trou Magrite to varying degrees of completeness (from 4.35% to 100% of the coding region; Supplementary Information section 10). We then used BEAST2 (ref. ^33^) to generate a dated, individual-level mitochondrial phylogeny using 11 new, 1 updated and 28 previously published Neanderthal mtDNA genomes (Fig. 2). We found that most of the newly reconstructed mtDNA genomes fall within a large clade consisting of late Neanderthals from southern and western Europe, as well as Mezmaiskaya 2 from Russia^34^ (Fig. 2a). We estimated their ages to be between 41.06 kyr bp (95% highest posterior density interval (HPDI) 47.55–33.10 kyr bp) and 50.51 kyr bp (95% HPDI 67.06–30.00 kyr bp) (Fig. 2a and Supplementary Table 4), and their most recent mitochondrial split time to be 74.51 kyr bp (95% HPDI 89.23–61.65 kyr bp). As shown previously^11^, the approximately 42 kyr-old Les Cottés Z4-1514 is part of a deeper mtDNA clade including individuals from Chagyrskaya and Okladnikov caves in Russia, with an estimated split time of 114.08 kyr bp (95% HPDI 134.67–94.54 kyr bp).

**Extended Data Fig. 2:**
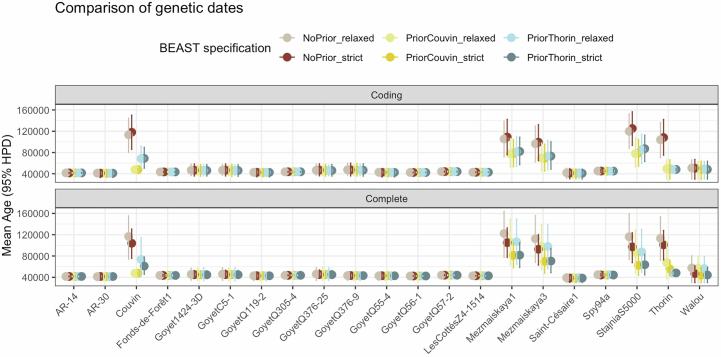
Estimated genetic ages (with their respective 95% highest posterior density intervals (HPDI)) for all of the focus Neanderthals. The colours indicate whether the priors included the radiocarbon dates available for either Thorin, Couvin, or none of them, as well as the molecular clock model (strict or uncorrelated normal). The number of SNPs from each mitochondrial genome are reported in Supplementary Table 2.4.

The exception was the mtDNA of the Neanderthal from Couvin (Couvin G6-0083, Belgium), which falls in the same deeply divergent mitochondrial lineage as the ‘Thorin’ Neanderthal (Grotte Mandrin 1600) from Mandrin (Rhône Valley, France)^2^. Relative dating estimated the age of the Couvin Neanderthal to 49.66–44.17 calibrated (cal.) kyr bp, based on the combined radiocarbon dates of several faunal bones recovered from the same layer; and direct dating yielded estimates of 53–48 cal. kyr bp for the Mandrin Neanderthal. Both estimates are close to the limits of radiocarbon dating. Molecular estimates of the ages of the Couvin and Mandrin Neanderthals are highly sensitive to the priors selected for the molecular dating. Models not constrained by radiocarbon dates estimate both individuals to be older than 100 kyr bp (Extended Data Fig. 2 and Supplementary Information section 10). By contrast, integrating radiocarbon dates associated with either specimen into the dating model shifted their age estimates to 69.05 kyr bp (95% HPDI 89.68–50.45 kyr bp) for the Couvin Neanderthal and 49.14 kyr bp (95% HPDI 66.43–30.07 kyr bp) for the Mandrin Neanderthal, that is, closer to their associated radiocarbon dates. Although the confidence intervals are wide, there is an apparent discrepancy between genetic and radiocarbon dates for the Couvin Neanderthal, similar to what has been reported for Mandrin^2^. Considering the archaeological and stratigraphic contexts of the Couvin G6-0083 molar, the incorporation of the tooth in the archaeological Unit II from an older deposit seems unlikely^35^. This raises the possibility that a divergent maternal lineage, which possibly includes the Stajnia and Forbes Quarry Neanderthals^36,37^, was widespread in western Europe, and perhaps co-existed with other late Neanderthal lineages.

We next analysed the data generated with a custom Y chromosome capture panel for three of the newly identified male individuals. We used between 2,897 and 13,351 Y chromosome SNPs to place these individuals on a tree relating high-quality Y chromosomes of Neanderthals, Denisovans and modern humans^1^, which includes Spy94a_8 (Fig. 2b and Supplementary Tables 5 and 6). In contrast to the mtDNA, in which most late western Neanderthals are part of a single clade, we find that their Y chromosomes do not cluster by geography, and none of the new Y chromosomes share the Y haplotype seen in Spy94a_8. The Y chromosomes of Fonds-de-Forêt 1 and Trou Magrite 2422-36 are most similar to the seven available Chagyrskaya Y chromosomes, whereas the Y chromosome of Goyet Q305-1 is most similar to that of Mezmaiskaya 2. The newly sequenced Y chromosomes fall within the variation of previously sequenced Neanderthal Y chromosomes and represent clades with a split time of 118 kyr bp (133–102 kyr bp)^38^.

## Heterozygosity and population size

**Fig. 3:**
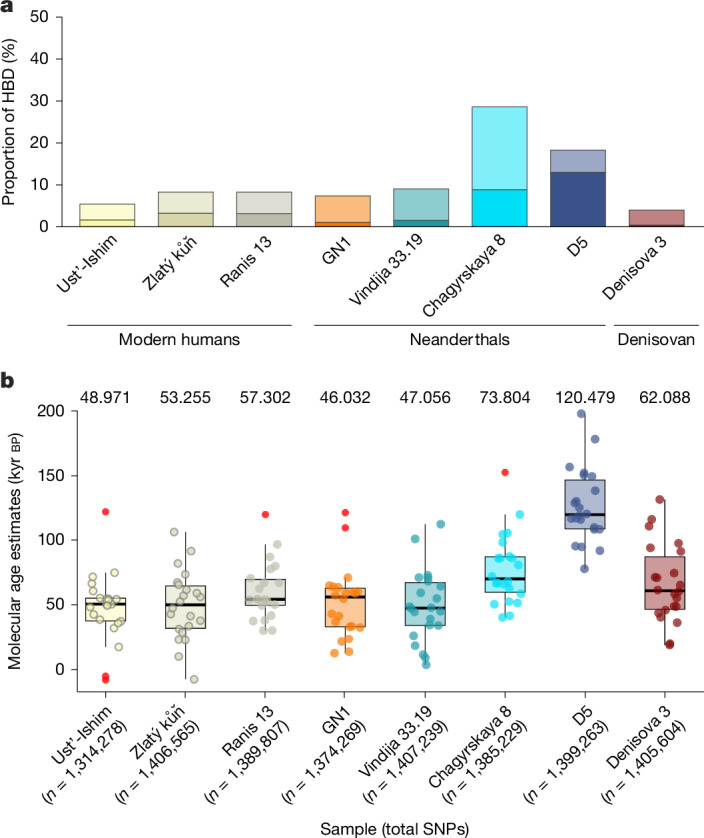
Archaic genetic diversity. **a**, Proportion of HBD in the genome of each archaic and ancient human individual using the deCODE map^60^. Each bar represents the optimal value for the parameter *π*. Light and dark colours indicate the inbreeding proportion in short (2.5–10 cM) and long (>10 cM) HBD segments, respectively. **b**, Genetic age estimates by branch shortening, taking present-day Mbuti as reference. On the *x* axis, the total number of SNPs used appears below each individual, and the weighted mean age (in kyr bp) at the top. Each point is a chromosome, and outliers are represented as red dots and were excluded from the calculation of the average age. The lower and upper hinges of the box plots represent the 25th and 75th percentiles, respectively, and the whiskers extend up to 1.5 × IQR from the hinges.

**Extended Data Fig. 3:**
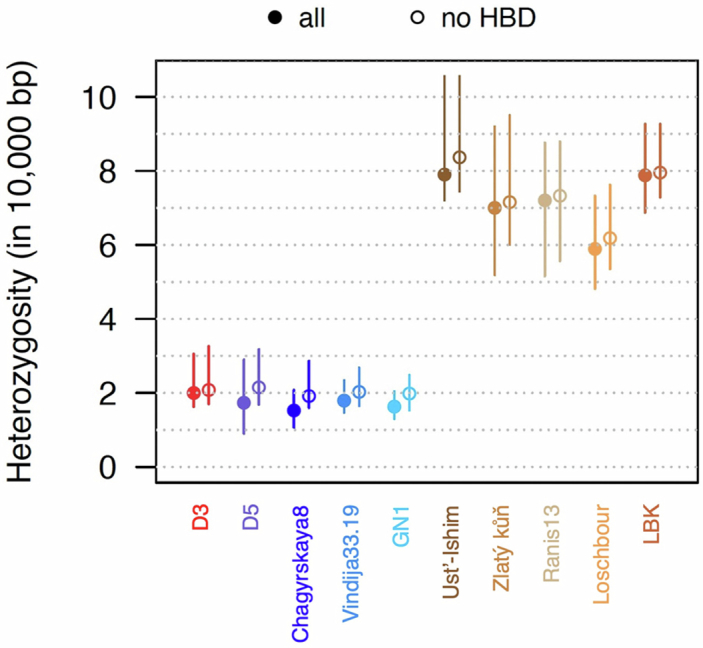
Heterozygosity estimates of the high-coverage archaic and ancient human genomes. Each data point corresponds to one genome. Filled and empty circles indicate the genome-wide heterozygosity using all sites and excluding inferred homozygous by descent (HBD) segments, respectively. The vertical lines represent the range of heterozygosity calculated on the (n = 22) autosomes of each individual.

Using the high-coverage nuclear genome obtained for the GN1 Neanderthal, we estimated the autosomal genome-wide heterozygosity of GN1 to be 1.51 × 10^−4^ (per-chromosome estimates: 1.20 × 10^−4^ to 1.83 × 10^−4^; Supplementary Information section 7), which falls within the range of known Neanderthal variation and is substantially lower than that found in present-day humans (Extended Data Fig. 3). We used patterns of heterozygosity along the genome to infer the population history of GN1 using the pairwise sequentially Markovian coalescent (PSMC)^39^. After correcting for missing data^30^, we found that the demographic history of GN1 was very similar to that of the Vindija 33.19 Neanderthal, supporting that they shared a common population history (Supplementary Information section 6). Using *f*(*A* | *B*)-statistics^8,9,11^, we estimated that the populations represented by *A* = GN1 and *B* = Vindija 33.19 split around 54 kyr bp (95% CI 55–53 kyr bp) (Supplementary Information section 16) and nuclear branch shortening estimates suggest that they were probably of similar ages, with GN1 estimated to 46.03 kyr bp (interquartile range (IQR) 63.72–34.13 kyr bp) and Vindija 33.19 to 47.06 kyr bp (IQR 68.15–35.18 kyr bp; Fig. 3b and Supplementary Information section 5). Thus, both the radiocarbon and genetic dating place Vindija 33.19 and GN1 as roughly contemporaneous, within a few thousand years before Neanderthals disappeared.

All previously published high-coverage Neanderthal genomes^3,8,9^ were shown to carry more extended regions that were homozygous by descent (HBD tracts) than do most present-day humans. The D5 and Chagyrskaya Neanderthals from the Altai region both carry a greater number of, and longer, HBD tracts than the late western Neanderthal, Vindija 33.19. We estimate the total proportion of the genome contained in HBD tracts in GN1 to be between 6.6% and 22.3% depending on the genotyping error parameter used (*π*; Fig. 3a). These values are more comparable to the Vindija 33.19 Neanderthal than to the Chagyrskaya 8 and D5 Neanderthals. Moreover, unlike the Chagyrskaya and D5 Neanderthals, which carry numerous long HBD segments (>10 centimorgans (cM)) indicative of recent inbreeding between close relatives, GN1 shows no excess of such long tracts, providing no evidence for recent inbreeding.

## Population structure of late Neanderthals

**Fig. 4:**
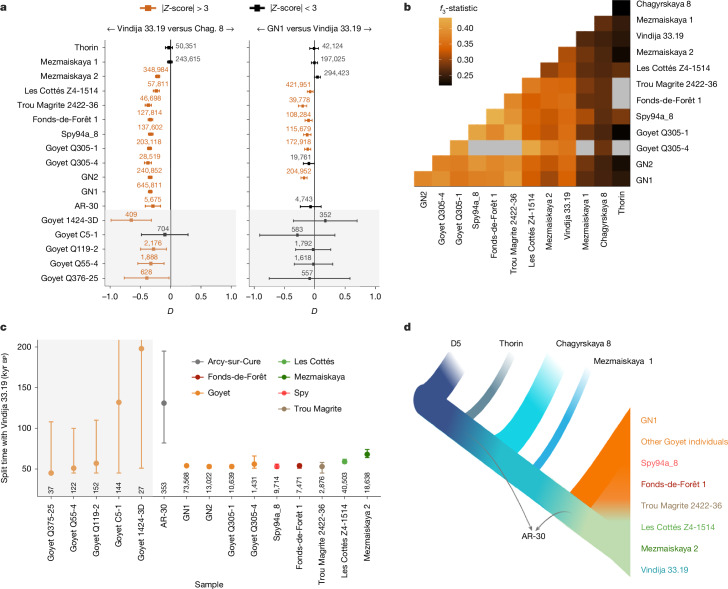
Population affinities of late Neanderthals. **a**, *D*-statistics of the form *D*(*X*, Vindija 33.19; *Y*, Mbuti), in which *X* represents either Chagyrskaya 8 (Chag., left) or GN1 (right), and *Y* represents the Neanderthals on the *y* axis. The numbers above the error bars (mean ± 3 × s.e.m.) indicate the number of SNPs used for each specific comparison (left, *n* = 1,652,202 SNPs; right, *n* = 1,310,536 SNPs), and the colour represents significance with a *Z*-score threshold of 3. **b**, Outgroup *f*_3_-statistics of the form *f*_3_(*X*, *Y*; D5), in which *X* represents the Neanderthals on the *x* axis and *Y* represents the Neanderthals on the *y* axis. Statistics with fewer than 1,000 overlapping sites appear in grey. **c**, Split time estimates with Vindija 33.19. Means and 95% CIs are given for each Neanderthal, coloured by site. **d**, The population genetic model proposed for Neanderthals. Samples shaded in grey in **a** and **c** have fewer than 7,000 informative positions covered by sequences and more than 6% present-day human contamination.

**Extended Data Fig. 4:**
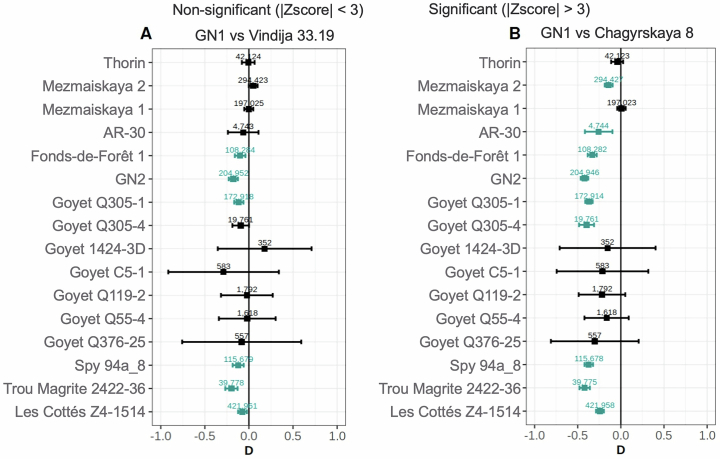
D-statistics of the form D (X, GN1; Y, D5), where “X” represents either Vindija 33.19 (facet A) or Chagyrskaya 8 (facet B), and “Y” represents the Neanderthals on the Y-axis. The errors around the mean (±3 standard errors) of the D-statistics are indicated, coloured depending on whether the associated Z-score reached the absolute threshold of 3 for significance. The numbers above the error bars indicate the number of SNPs used for each specific comparison (N = 1,310,536 SNPs).

**Extended Data Fig. 5:**
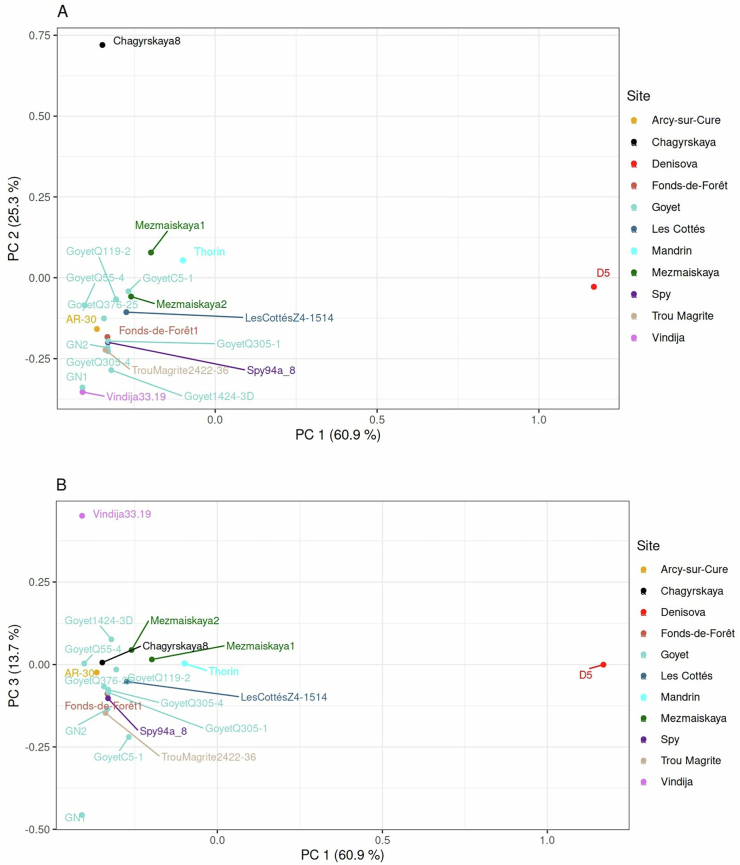
Principal Component Analysis (PCA) defined by the variation within high-coverage Neanderthals (N = 1,310,536 SNPs). A) First two principal components, B) First and third principal components.

**Extended Data Fig. 6:**
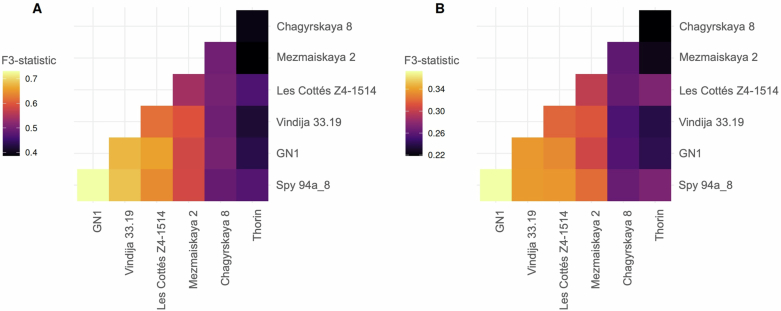
Outgroup *f3*-statistics of the form *f3(X, Y; D5)*, where “X” represents the Neanderthal on the X-axis and “Y” represents the Neanderthal on the Y-axis. A) Statistics based on unascertained data (N = 16,715,517 SNPs), and B) restricted to ArchaicPlus sites (N = 1,310,536 SNPs).

We next explored the relationships of the Neanderthals from Belgium and France to other late Neanderthals using high-coverage shotgun data and low-coverage nuclear genome data generated using ArchaicPlus hybridization capture (Extended Data Table 1). Among the individuals sequenced to high coverage, the GN1 Neanderthal is most closely related to the contemporaneous Vindija 33.19 from Croatia, sharing a common ancestor approximately 10 kyr before these individuals lived (Supplementary Information section 16). Using both *D*-statistics and outgroup *f*_3_ analyses, we found that for the Neanderthals with sufficient sequencing data from Fonds-de-Forêt, Spy, Goyet and Trou Magrite are all significantly more similar to GN1 than to Vindija 33.19 (Fig. 4a,b, Extended Data Figs. 4–6 and Supplementary Table 7; *D* = 0.093–0.198, *Z*-score = 2.97–10.20). The *D*-statistics also show that all of the analysed late Neanderthals share significantly more alleles with Vindija 33.19 than with the Chagyrskaya 8 or D5 Neanderthals (Fig. 4a; *D* = 0.215–0.37,6 *Z*-score = 7.01–32.51 for the former; and *D* = 0.481–0.580, *Z*-score = 14.40–72.53 for the latter). We estimated the population split time between each of the low-coverage Neanderthal genomes and the high-coverage Vindija 33.19 genome using *f*(*A* | *B*)-statistics together with the inferred change in Neanderthal population size over time^8,39^ (Supplementary Table 8). We estimated that the Neanderthals studied here separated from a common ancestor with the Vindija 33.19 Neanderthal approximately 54 kyr bp (95% CI 58–51 kyr), concordant with the result for GN1 (Fig. 4c). Overall, this indicates that these late Neanderthals were more closely related to each other than to the other contemporaneous Neanderthals from Vindija and Mezmaiskaya, concordant with their geographical location (Fig. 4d).

**Extended Data Fig. 7:**
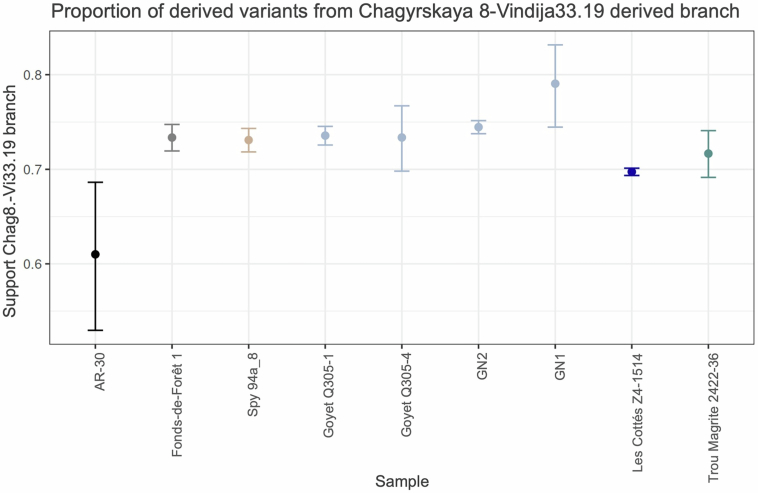
Proportion of derived variants specific to either Chagyrskaya 8, Vindija 33.19 or both. Means ± 95% confidence intervals (CI) are given for each Neanderthal sample with <5% present-day DNA contamination, coloured by the archaeological site (N = 159; 3,900; 4,984; 7,766; 657; 15,392; 358; 54,829; 1,320 SNPs, from left to right).

Although the AR-30 Neanderthal from Arcy-sur-Cure (approximately 42 kyr bp)^40,41^ is genetically closer to Vindija 33.19 than to Chagyrskaya 8 (*D* = 0.293, *Z*-score = 7.01), estimates based on *f*(*A* | *B*)-statistics indicate that she belongs to a population that split approximately 131 kyr bp (95% CI 195–82 kyr bp) from a common ancestor with the Vindija 33.19 Neanderthal (Fig. 4c). This is much earlier than the split estimated for the Neanderthals from Goyet, Fonds-de-Forêt, Spy and Trou Magrite. This difference in split times is driven by AR-30 sharing a smaller proportion of the derived variants on the Vindija 33.19–Chagyrskaya 8 branch (Extended Data Fig. 7 and Supplementary Information section 16). An excess of ancestral alleles in AR-30, perhaps due to faunal contamination, could increase the split time. However, using the metagenomic pipeline quicksand^42^, we found no sequences assigned to any mammalian family besides Hominidae, making it unlikely that the signal is due to faunal contamination. Similarly, the estimated modern human contamination of 1.3% (95% CI 0.3–2.8%) is too low to explain this signal. The excess of ancestral variants could also be compatible with gene flow into AR-30 from a Neanderthal lineage that diverged sometime between the split of the Chagyrskaya 8 and D5 Neanderthals (Supplementary Information section 16). However, more data from AR-30 would be needed to validate this hypothesis.

## No recent modern human ancestry

**Fig. 5:**
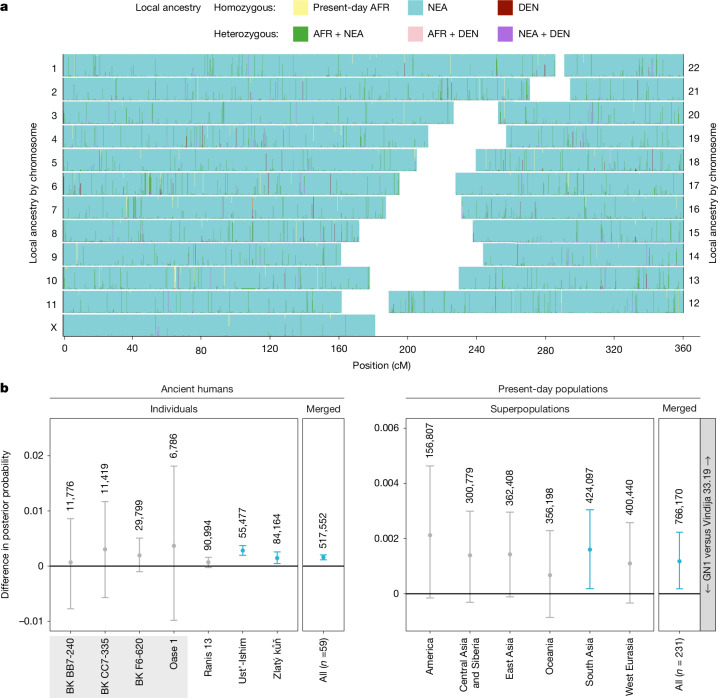
Gene flow between GN1 and modern humans. **a**, Modern human, Denisovan and Neanderthal ancestry in GN1, coloured according to the admixfrog posteriors. **b**, Difference in posterior estimates of the probability of derived alleles on an introgressed segment matching the diagnostic positions of Vindija 33.19 versus GN1 in six selected ancient modern humans that did not substantially contribute to present-day modern human populations (left, those with very recent Neanderthal ancestry are shaded in grey) and six continental superpopulations clustering present-day individuals with ancestry other than African ancestry from the Simons Genome Diversity Project data (right). Merged, all individuals pooled together. The segments were called using the D5, Chagyrskaya 8, Vindija 33.19 and GN1 Neanderthals as reference for Neanderthals. Points indicate the mean posterior difference and error bars indicate the 95% HDPI. Estimates in blue indicate a significantly better match to one of the Neanderthals. The total number of unique positions across all introgressed segments is indicated above each point. AFR, African; DEN, Denisovan; NEA, Neanderthal.

**Extended Data Fig. 8:**
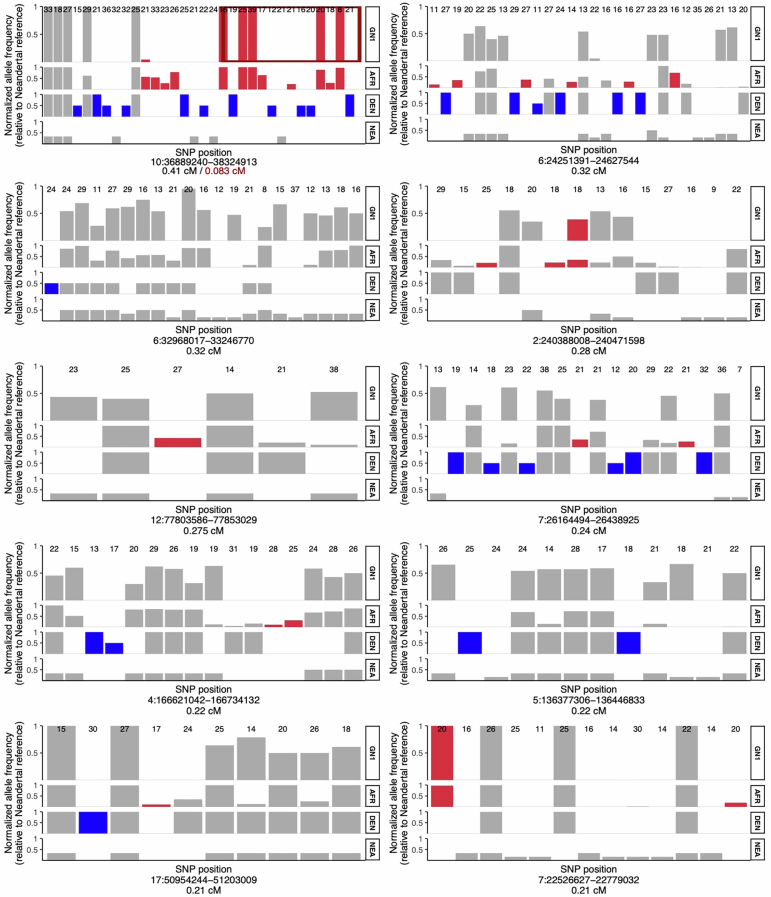
Allelic frequencies at each position of the modern-human (African, AFR) segment identified in the genome of GN1. Each plot represents an identified fragment, plots are sorted by genetic length. DEN, Denisovans; NEA, Neanderthals.

The estimated ages of the Neanderthals in this study, based on either radiocarbon or molecular dating, are between 49.8 cal. kyr bp and 40 cal. kyr bp (Supplementary Table 24). This overlaps with the presence of early modern humans in Europe^30,43^, the estimated time of gene flow between Neanderthals and modern humans^30,44^, and genetic evidence of modern humans with recent Neanderthal ancestry at both Peştera cu Oase (Romania)^45^ and Bacho Kiro Cave (Bulgaria)^46^. Moreover, the AR-30 Neanderthal was recovered from an archaeological layer that also yielded a bone attributed to modern humans^41^. We used admixfrog^47^ (v.0.7.2) to test for recent modern human introgression in these late Neanderthal genomes. As these Neanderthals lived at most 500 generations after the first possible interactions with early modern humans, we would expect multiple introgressed modern human DNA tracts longer than 1 cM in their genomes if introgression took place. We identified potential tracts of modern human DNA in four of the ten Neanderthals, but the longest tract we found was only 0.41 cM long, inconsistent with recent introgression (Fig. 5a and Supplementary Table 36). Because all of the putative modern human tracts we identified were short, and lacked fixed differences between Neanderthals and modern humans (Extended Data Fig. 8 and Supplementary Information section 17), they are consistent with previous evidence of much older gene flow from modern humans to Neanderthals or with incomplete lineage sorting^48^.

Although we now have nuclear data from 16 Neanderthals who lived less than 50 kyr ago, we have not yet identified any Neanderthal with recent modern human ancestry. This is in contrast to early modern humans in which all 13 of the genomes of individuals predating 40 kyr in Eurasia have Neanderthal ancestry, and four of them have Neanderthal ancestors only 4–10 generations before they lived^45,46^. This apparent asymmetry of gene flow, which has been noted previously^49^, may reflect the dynamics of introgression, with Neanderthal gene flow into modern humans occurring early during the expansion of modern humans into Eurasia and all later modern humans being descended from the admixed population. By contrast, at the time of introgression, Neanderthals were more widespread and any introgression probably affected only local groups. An alternative is that the absence of modern human DNA in late Neanderthals could reflect demographic imbalances, biases in mate choice, the incorporation of the offspring of Neanderthal–modern human couples in primarily modern human groups or differential fitness of offspring.

## Neanderthal ancestry in modern humans

Previous studies have shown that the Neanderthal ancestry in both present-day and ancient modern humans is more closely related to the Vindija 33.19 Neanderthal than to the Chagyrskaya 8 and D5 Neanderthals^3,9^. Given the new high-coverage genome of a late Neanderthal from Goyet, GN1, we re-examined which of the sequenced Neanderthals is most closely related to the Neanderthals that introgressed into modern humans using two complementary approaches.

**Extended Data Fig. 9:**
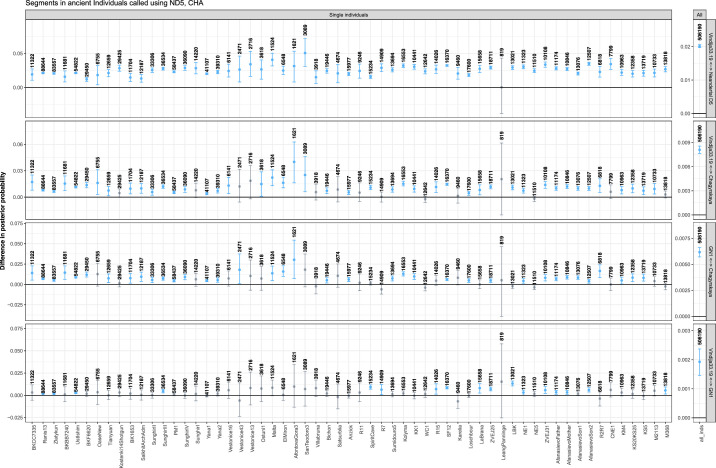
Difference in posterior estimates of the probability of derived alleles on an introgressed segment matching the diagnostic positions of: Vindija 33.19 vs. D5, Vindija 33.19 vs. Chagyrskaya 8, GN1 vs. Chagyrskaya 8 and Vindija 33.19 vs. GN1 Neanderthals, for 58 ancient non-African individuals (left panel) and all individuals pooled (right panel). The segments were called using the D5 and Chagyrskaya 8 Neanderthals as reference for Neanderthals. Points indicate the mean posterior difference and error bars indicate the 95% HDPI. Blue coloured individuals’ estimates indicate a significantly better match to one of the Neanderthals. The total number of unique positions across all introgressed segments is indicated above each point.

**Extended Data Fig. 10:**
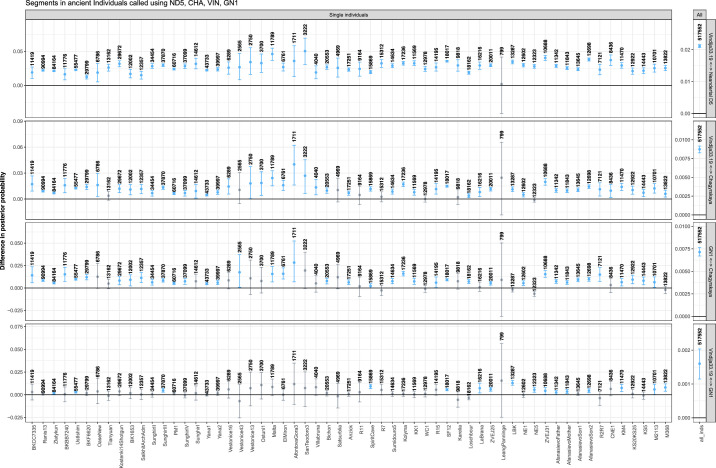
Difference in posterior estimates of the probability of derived alleles on an introgressed segment matching the diagnostic positions of: Vindija 33.19 vs. D5, Vindija 33.19 vs. Chagyrskaya 8, GN1 vs. Chagyrskaya 8 and Vindija 33.19 vs. GN1 Neanderthals, for 58 ancient non-African individuals (left panel) and all individuals pooled (right panel). The segments were called using the D5, Chagyrskaya 8, Vindija 33.19 and GN1 Neanderthals as reference for Neanderthals. Points indicate the mean posterior difference and error bars indicate the 95% HDPI. Blue coloured individuals’ estimates indicate a significantly better match to one of the Neanderthals. The total number of unique positions across all introgressed segments is indicated above each point.

First, we used admixfrog to identify Neanderthal ancestry segments in a panel of ancient and present-day modern human genomes (Supplementary Information section 8.1) and then applied a Bayesian binomial model to estimate the genetic distance and identify the closest matching Neanderthal. Second, we used stratified *D*-statistics^9^ (Supplementary Information section 8) to focus on low-frequency allele bins in which introgressed variants are expected to be enriched. In both analyses, we found that the Neanderthal ancestry in all individuals excluding African people is significantly closer to Vindija 33.19 and GN1 than to Chagyrskaya 8 or the Neanderthal D5. As Vindija 33.19 and GN1 stem from closely related Neanderthal populations, their comparison is particularly challenging. Although there is a tendency for the Neanderthal ancestry in present-day humans to be more similar to Vindija 33.19 than to GN1, this is significant only when pooling all present-day individuals (Fig. 5b, Extended Data Figs. 9 and 10 and Supplementary Information section 8). This suggests that most admixture probably occurred between modern humans and Neanderthals more closely related to Vindija 33.19 than with the late Neanderthals from Belgium and France. By contrast, Neanderthal ancestry in the four Pleistocene modern humans with very recent Neanderthal ancestors is not significantly closer to Vindija 33.19 than to GN1 (Fig. 5b). Thus, it is possible that early modern humans received some ancestry from Neanderthals closer to GN1, resulting in an overall non-significant *D*-statistic.

## Neanderthal disappearance

The Neanderthals studied here are some of the last known individuals from northwestern Europe^4,7^, who lived shortly before the disappearance of Neanderthals. Compared with modern humans, Neanderthals had a much lower effective population size, with weaker purifying selection and possibly reduced fitness^50,51^. It has been suggested that a loss of genetic diversity may have contributed to their disappearance^8,24,52^. We compared three measures of genetic load that have been shown to increase over time in other extinct^53^ or critically endangered species^54^. For coding variants, we measured the ratio of missense to synonymous variants (pN/pS), as well as testing genome-wide shifts towards more deleterious variants using polyPhen2 (ref. ^55^). Furthermore, we used a second conservation score, phyloP^56^, which is defined for both coding and non-coding variants (Supplementary Information section 4). We found a depletion of non-synonymous variants in all Neanderthals (for GN1: pN/pS = 0.76, Fisher’s exact test *P* = 0.0006). However, there is no significant difference between the ratios in early versus late Neanderthals for either pN/pS (Fisher’s exact test, *P* = 0.0959) or for the two conservation scores (polyPhen2: Wilcoxon rank-sum test, *W* = 116.5, *P* = 0.08; phyloP: *t*-test, *t* = 0.42, *P* = 0.65; Supplementary Information section 4). Because we also observed no increase in homozygous tracts (Fig. 3) or decrease in heterozygosity (Extended Data Fig. 3), these results are inconsistent with models in which accumulating genetic load or reduction in diversity over time was the primary driver of the disappearance of Neanderthals.

## Conclusions

The sequencing of the high-coverage Neanderthal genome (GN1) establishes a critical reference point for investigating the genetic diversity of late Neanderthals in Belgium and France shortly before their disappearance. Complementing this, we combined laboratory and computational methods tailored to highly fragmented ancient DNA to recover genome-wide data from a larger set of further Neanderthal remains with poor DNA preservation. Together, these genomes provide an overview of late Neanderthal genetic diversity, and demonstrate that even low-coverage nuclear genome data, when integrated with palaeoanthropological context, can substantially increase the resolution of within-Neanderthal diversity. Although the individuals at Goyet form a distinct genetic cluster together with the other Neanderthals from the Meuse Valley, their isotopic values indicate that they were not local to the region^28^. Furthermore, the assemblage consists of cannibalized remains of unrelated female and two juvenile individuals, all indicating that they were not a family group. Notably, we found low genetic differentiation within and between the Neanderthals from archaeological sites in the Meuse Basin. Considering also that GN1 and Vindija 33.19 lacked the long HBD tracts present in the Chagyrskaya 8 and D5 Neanderthals, we conclude that these western Neanderthals had higher genetic diversity and greater connectivity than did the Denisova and Chagyrskaya Neanderthals from the Altai region, suggesting that mixing between close relatives was not a feature common to all Neanderthal groups. Future studies from different time periods or from other parts of Eurasia will be key to determining whether the patterns observed here represent a local anomaly or a broader feature of Neanderthal social organization. Both the nuclear data of Arcy-sur-Cure and the mtDNA genome of Couvin hint at a more complex population structure of late Neanderthals, but higher-quality data will be needed to corroborate this. Taken together, the genetic data indicate substantial connectivity among late Neanderthal groups in northwestern Europe, consistent with isolation-by-distance patterns. Rather than reflecting a permanent biogeographical crossroads, this connectivity probably resulted from episodic phases of regional openness in a broader framework of climatically driven population contraction and expansion during the Middle and Upper Pleistocene^57^. Finally, we found that although late Neanderthals in Europe overlapped with early modern humans, none of the Neanderthal genomes analysed show evidence of recent modern human introgression. Moreover, the closer relationship of Neanderthal segments in present-day humans to the Vindija 33.19 Neanderthal than to GN1 indicates that gene flow between modern humans and Neanderthals probably occurred predominantly outside northwestern Europe.

## Methods

### DNA extraction and library preparation

All laboratory work was conducted in dedicated ancient DNA clean room facilities, at the Max Planck Institute for Evolutionary Anthropology, the Royal Belgian Institute of Natural Sciences or the University of Tübingen. Bone powder (1–57.1 mg; Supplementary Table 1.1) was obtained from 35 skeletal remains using a sterile dentistry drill. More than one-third of the powdered samples underwent a pretreatment with either 0.5% hypochlorite solution or a phosphate buffer, in an attempt to reduce present-day contamination^13^, before DNA extraction using a silica-based protocol optimized for recovery of short, degraded DNA fragments^14^. Single-stranded sequencing libraries were prepared using between 5 μl and 30 μl of the extracts^15^, for most libraries using automated liquid handling (10.17504/protocols.io.kqdg32bdpv25/v1)^61^. A subset of the earliest libraries were prepared using a modified single-stranded DNA protocol with partial uracil-specific excision reagent (USER) treatment, reducing uracil content while preserving terminal deamination signals^62^. To enable multiplex sequencing, each library was indexed with a pair of unique 7 or 8 base pair (bp) barcodes^63,64^. We quantified library yields by quantitative PCR or digital droplet PCR^15^ (10.17504/protocols.io.bp2l6xwd5lqe/v2)^65^, and quantified library preparation efficiency using spiked-in control oligonucleotides^66^. Negative controls were included in all extraction, library preparation and sequencing steps to monitor for contamination.

### Shotgun sequencing

To screen for endogenous DNA preservation, libraries were shallowly sequenced on Illumina platforms (Supplementary Information section 1.2). Sequencing data processing included adapter trimming and read merging using leeHom^67^ (v.1.2.18, https://bioinf.eva.mpg.de/leehom/), followed by mapping to the human reference genome (hg19/GRCh37) with BWA^68^ (v.0.5.10-evan.9-1-g44db244, https://github.com/mpieva/network-aware-bwa) using parameters optimized for ancient DNA^69^. PCR duplicates were removed using bam-rmdup (https://github.com/mpieva/biohazard-tools/*)* and reads were filtered for a minimum length of 35 bp and a mapping quality of 25. DNA authenticity was evaluated by determining the frequency of deamination-induced C-to-T substitutions at the ends of reads, requiring more than 10% substitutions at terminal bases as evidence for the presence of authentic ancient DNA. Contamination levels were assessed by comparing deamination rates before and after conditioning on damage at one end^70^ (Supplementary Table 1.1).

### Mitochondrial capture

Libraries were enriched for mtDNA through two rounds of in-solution hybridization capture (Supplementary Information section 10). We used probes tiling the revised Cambridge reference sequence of the human mtDNA genome (rCRS^71^) for all samples except A57813, which was captured with an array containing mitochondrial genomes from 241 mammalian species in addition to the rCRS reference^72^. After sequencing on Illumina MiSeq or HiSeq platforms, reads were processed with leeHom^67^ and mapped using BWA^39^ (v.0.5.10) with ancient DNA parameters^69^ to both rCRS and a circularized Vindija 33.16 mitochondrial reference. After duplicate removal and quality filtering (mapQ > 25, length > 35 bp), authenticity was confirmed by requiring at least 10% C-to-T damage. Contamination from modern human DNA was conservatively estimated by counting fragments with modern human-specific alleles at lineage-diagnostic positions in the rCRS alignment^73^. We compared the effect of using all sequences versus only those with putative terminal deamination, as well as two different thresholds for contamination (10% and 25%), and found no significant differences in the resulting mitochondrial consensus genomes. Consensus calling was performed with different parameters, requiring a consensus support of either 66% or 80% and a minimum coverage of either 3× or 4×, all while requiring a base quality score of 20 and masking transitions at the three last bases at the ends of the reads to mitigate errors due to damage-induced substitutions.

We constructed a dated phylogeny with a multiple alignment, generated using mafft with 1,000 iterations^74^ (v.7.453), of the new Neanderthal mtDNA genomes, as well as previously published mtDNA genomes from 23 Neanderthals^1,2,4,8,11,21,23,34,75–80^, 55 present-day humans^71,81^, 10 ancient modern humans^16,70,82–86^, the Sima de los Huesos hominin^70^ and 4 Denisovans^84,87–89^ using BEAST2 (v.2.1.3)^33^. We determined that TrN as the substitution model^90^ described our data best, and set the proportion of invariant sites of 0.8. We ran this analysis multiple times, using all sequences or only those in the coding portion of the mitochondria, as well as testing different models with different clocks and trees, and using different priors for the tip ages of Couvin G8-0083 and Thorin. Moreover, we also explored the viability of combining non-overlapping radiocarbon dates for GN2, and the effect that using different dates from the different specimens would have on the posterior dates. These analyses are described in more detail in Supplementary Information section 10.

### Y chromosome capture

Three out of the four genetically male individuals had enough sequences mapping to the non-recombining portion of the Y chromosome^91^ for downstream analyses (Supplementary Information section 11). Owing to the low coverage, we did not construct a de novo phylogeny. Instead, the new data of Goyet Q305-1, Fonds-de-Forêt 1 and Trou Magrite 2422-36 were used to place the new individuals into an existing high-quality phylogeny of archaic Y chromosomes^1^ to determine their affiliations with previously sequenced male Neanderthals.

### GN1 high-coverage genome

From the Goyet Q56-1 specimen (GN1 individual), three single-stranded, non-UDG-treated libraries with around 37% endogenous DNA were sequenced to high coverage (Supplementary Information section 2). To improve sequencing efficiency, we used gel excision to remove fragments shorter than 35 bp (ref. ^15^), and pooled the remaining molecules for sequencing on an Illumina HiSeqX platform at SciLife. Reads were processed using leeHom for adapter trimming and merging, and aligned to the hg19 reference with BWA using the same software, ancient DNA parameters and reference sequence described above. PCR duplicates were removed (bam-rmdup) and only fragments with mapQ > 25 and length > 35 bp were retained for downstream analysis. We assessed present-day human DNA contamination in each library using five complementary methods: AuthentiCT^92^ (v.1.0.1), a likelihood-based divergence method^69,93^, a conditional substitution approach^6,36^, excess Y chromosome reads^8,93^ and *f*-statistics comparing all versus deaminated reads with present-day populations from the Simons Genome Diversity Project^94^ (Supplementary Tables 1.3 and 2.1). We merged the data from the three libraries using SAMtools^68^ (v.1.3.1-21), resulting in a genome-wide coverage of 22.4-fold, and carried out genotyping using snpAD^95^ (v.0.3.11).

### Population history of GN1

The demographic history of the GN1 individual was inferred using PSMC^39^ (v.0.6.5; Supplementary Information section 6). The analysis was run using the pipeline available at GitHub (https://github.com/StephanePeyregne/calibratePSMC), with standard parameters and correcting for missing data^30^. We calculated the genetic age of GN1 using a molecular branch shortening approach^8^, which assumes that older individuals have accumulated fewer lineage-specific mutations. To do this, we counted derived transversions (to mitigate ancient DNA damage) on the GN1 lineage, using four great ape outgroups, and calibrated assuming a divergence of 13 million years ago with a mutation rate of 0.5 × 10^−9^ per bp per year^8^.

### Genetic diversity of GN1

We calculated the genome-wide heterozygosity for GN1 as the ratio of heterozygous sites to the total number of callable sites and compared this value with those of other high-coverage genomes of interest (Supplementary Information section 7). We then identified HBD segments, indicative of recent inbreeding, by using a method tailored for ancient DNA data^3,8,30^. This method accounts for genotyping errors through a parameter, *π*, and has been optimized to increase sensitivity for longer segments while reducing false positives for shorter segments (≥2.5 cM and <10 cM). In the absence of a Neanderthal recombination map, recombination levels were calculated using three recombination maps: a constant rate, the African-American map^96^ and the deCODE map^60^. The total proportion of the genome found in such HBD segments was calculated and compared with those of other high-coverage Neanderthal genomes.

### Functional analysis of GN1

We identified derived variants in the different Neanderthal lineages, focusing only on bi-allelic sites in which the global allele frequency of the derived allele was lower than 0.5% among the modern individuals from the gnomAD genome (v.3.1.2) or gnomAD exome (v.4.0) datasets^97^. Then, we annotated those that were specific to the high-coverage Neanderthal genomes using the Ensembl Variant Effect Predictor (v.112, https://www.ensembl.org/info/docs/tools/vep/index.html). To look for biological pathways that might be overrepresented, we then performed a functional enrichment analysis of Neanderthal-specific protein-coding and regulatory variants using the GOfuncR R package (v.1.23.2, https://github.com/sgrote/GOfuncR) to test for overrepresentation in Gene Ontology and Human Phenotype Ontology categories^98^, and using the ABAEnrichment R package^99^ for overrepresentation in the Allen Brain Atlas (http://www.brain-map.org). The associated family-wise error rates associated enabled us to account for multiple testing. Finally, to test for selection in the Neanderthal lineage, we focused on polymorphic sites in Neanderthals and performed McDonald–Kreitman tests contrasting non-synonymous fixed differences (dN), synonymous fixed differences (dS), non-synonymous polymorphisms (pN) and synonymous polymorphisms (pS).

### ArchaicPlus nuclear capture

For 43 libraries with low endogenous DNA content, we performed in-solution hybridization capture using a custom ArchaicPlus array, with around 2.3 million sites chosen to specifically target archaic variation and quantify present-day human contamination (Supplementary Information sections 12 and 13). Data from captured libraries were sequenced on HiSeq4000 and NextSeq platforms (Illumina), and demultiplexed and processed bioinformatically as described in the ‘Shotgun sequencing’ section, with the difference that they were mapped to a modified hg19 reference that included a third allele (distinct from the reference and alternative) to mitigate reference bias. Contamination was estimated using an approach that modelled contamination as the linear combination of the frequencies of derived alleles in endogenous and exogenous sequences^1,23^, as well as AuthentiCT^92^ (Supplementary Table 5). Because the resulting contamination estimates were generally higher than 2%, all subsequent analyses were conservatively restricted to deaminated sequences (C-to-T substitutions in the terminal 3 bp). Libraries originating from the same specimen were merged using SAMtools merge v.1.3.1 (ref. ^68^).

### Kinship and genetic sex determination

Genetic sex was determined by calculating the ratio of observed read coverage on the sex chromosomes versus the autosomes, normalized by the number of targeted SNPs on the ArchaicPlus array (Extended Data Fig. 1 and Supplementary Information section 13.3). Individuals were assigned as genetically female or male based on normalized X chromosome and Y chromosome ratios. To identify skeletal remains from the same or closely related individuals, we used the software KIN^32^, a likelihood-based method that detects up to third-degree relatives using low-coverage data while modelling contamination. The analysis was run on the curated capture dataset after applying strand-specific filtering to mitigate ancient DNA damage using a custom script. The average pairwise difference between unrelated individuals (P0) was estimated from the highest-quality samples to calibrate the analysis.

### Population affinities

We investigated the population affinities of the low-coverage Neanderthal genomes using *D*-statistics and *f*-statistics (Supplementary Information section 15). We computed the statistics using admixr^100^ (v.0.9.1), which relies on ADMIXTOOLS^101^ (v.7.0). First, we computed the *D*-statistics of the form *D*(High-coverage Archaic 1, High-coverage Archaic 2; Test Neanderthal, Mbuti) to test whether a test individual shared more alleles with one of two high-coverage archaic individuals used in the ArchaicPlus ascertainment (for example, Vindija 33.19 or Chagyrskaya 8) (Supplementary Table 7.1). To perform the same tests with samples that were not part of the ArchaicPlus ascertainment, such as GN1, it was necessary to remove sites specific to Vindija 33.19 and Chagyrskaya 8 to control for ascertainment bias, and, to gain further power, we used a closer outgroup, Neanderthal D5 (Supplementary Table 7.2). Using this corrected SNP set, we then calculated outgroup *f*_3_-statistics of the form *f*_3_(Test Neanderthal 1, Test Neanderthal 2, Neanderthal D5) (Supplementary Table 7.3) and *D*-statistics of the form *D*(Test Neanderthal 1, Test Neanderthal 2; Test Neanderthal 3, Neanderthal D5) to investigate population structure (Supplementary Table 7.4).

### Split times

Population split times were estimated using *f*(*A* | *B*)-statistics, which measure the proportion of derived alleles in a test individual (*A*) that are also derived in a reference high-coverage individual (*B*) ^93^ (Supplementary Information section 16 and Supplementary Table 8). These values, which correlate with genetic divergence time and range from 0 (very deep split time, no shared variation) to 0.5 for individuals from the same population, were calibrated into years bp by using the PSMC-derived demographic models and the branch shortening-based genetic age estimates for the high-coverage reference individuals (see ‘Population history of GN1’). It should be noted that ascertainment bias in the ArchaicPlus array precluded reliable split time estimation against non-ascertainment genomes (for example, Goyet Q56-1 could not be *B*) from the capture data.

### Local ancestry

To detect recent introgressed segments from modern humans or Denisovans in the genomes of Neanderthals, we used admixfrog^47^ (v.0.7.2; Supplementary Information section 17), a hidden Markov model that probabilistically assigns local ancestry to genomic segments. This approach explicitly models contamination, enabling the use of all sequencing data. The reference panel included three high-coverage Neanderthals (Vindija 33.19, D5 and Chagyrskaya 8)^3,8,9^, Denisova 3 (ref. ^69^) and sub-Saharan African populations from the 1000 Genomes Project^102^, polarized against the chimpanzee genome. Samples with coverage below 0.02× were excluded owing to inconclusive posteriors. In the remaining individuals, we called segments with a cut-off of 0.2 cM.

### Relationship to modern humans

To determine which of the sequenced Neanderthals is closest to the population that contributed the ancestry found in modern humans with ancestries other than African ancestry, we used two methods (Supplementary Information section 8). First, we assigned archaic lineages to introgressed segments identified with admixfrog^47^ (v.0.7.2), in a panel of 58 ancient and 231 present-day genomes other than African ones^44^. This was done using a Bayesian framework to calculate the posterior probability of a segment matching either GN1 or Vindija 33.19 as reference, in a set of around 2.5 million diagnostic sites. Second, we used stratified *D*-statistics of the form *D*(Neanderthal 1, Neanderthal 2; modern human, chimpanzee), conditioning on alleles that have a low frequency in African populations to enrich for variants that entered the modern human gene pool through Neanderthal introgression and restricting the analysis to transversions^9^.

### Reporting summary

Further information on research design is available in the Nature Portfolio Reporting Summary linked to this article.

## Online content

Any methods, additional references, Nature Portfolio reporting summaries, source data, extended data, supplementary information, acknowledgements, peer review information; details of author contributions and competing interests; and statements of data and code availability are available at 10.1038/s41586-026-10625-1.

## Supplementary information

Supplementary Information Supplementary sections 1–17, including Figs. 1–84, Tables 10–36 and references.

Reporting Summary

Supplementary Table 1 General characteristics and summary statistics of the shotgun sequencing data.

Supplementary Table 2 General characteristics and summary statistics of the mitochondrial capture data.

Supplementary Table 3 General characteristics and summary statistics of the Y chromosome capture data.

Supplementary Table 4 Variants in the non-recombinant portions of the Y chromosome.

Supplementary Table 5 General characteristics and summary statistics of the nuclear capture data.

Supplementary Table 6 Posteriors for the tip dates analysed in BEAST2.

Supplementary Table 7 *D*-statistics and *f*-statistics of nuclear capture data.

Supplementary Table 8 Population split times estimates between Neanderthals from present-day Belgium and France and other high-coverage Neanderthals.

Supplementary Table 9 Archaeological overview of the Neanderthals from the Meuse Basin with nuclear data.

Peer Review File

## Figures and Tables

**Extended Data Table 1:**
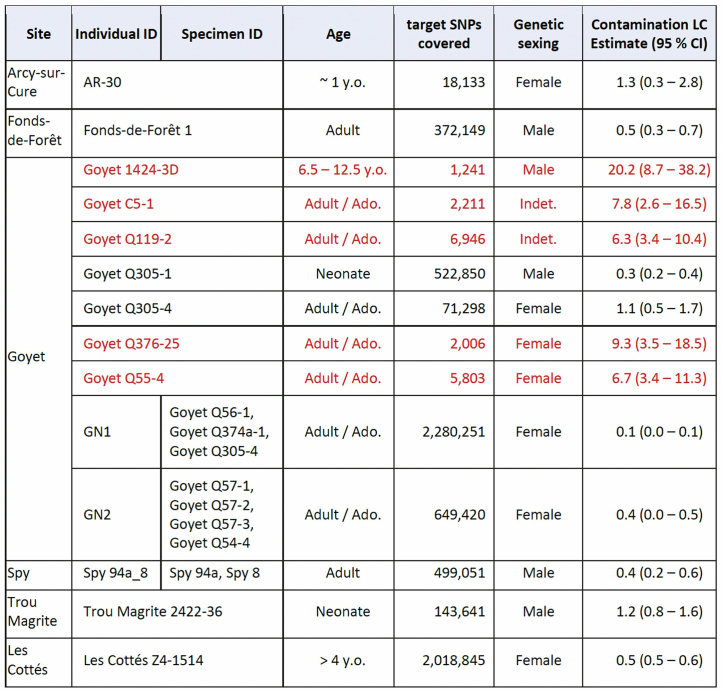
Overview of the nuclear data and archaeological information for the Neanderthal specimens

Samples in red exceeded the threshold for present-day human contamination (>2%) and were included only in analyses that are robust to contamination. LC: Linear Combination; CI: confidence interval; ID: identifier, y.o. = years old, Ado. = Adolescent, Indet. = Indeterminate.

## Data Availability

The DNA sequences from Neanderthals from this study have been deposited in the European Nucleotide Archive under accession number PRJEB98484. The FASTA files of the reconstructed mitochondrial genomes as well as the genotype calls of the nuclear genomes have been deposited in the Edmond repository (10.17617/3.F9N73O).

## Code availability

We did not develop code central to the main work described in this Article.
