# Supplementary material for: Genetic diversity of late Neanderthals in northwestern Europe
Source: Nature. 2026 Jun 24;655(8122):409–17. doi: 10.1038/s41586-026-10625-1 (PMC13345965; doi:10.1038/s41586-026-10625-1)
Supplement: Supplementary file 1 — Supplementary sections 1–17, including Figs. 1–84, Tables 10–36 and references. [file 41586_2026_10625_MOESM1_ESM.pdf]

nature portfolio

https://doi.org/10.1038/s41586-026-10625-1

Supplementary information

Genetic diversity of late Neanderthals in northwestern Europe

In the format provided by the authors and unedited

Supplementary Information

1. Archaeological context *2*

2. The Goyet Q56-1 or Goyet Neandertal 1 (GN1) high-coverage genome *16*

3. Genotyping the GN1 high-coverage genome *23*

4. Private variants of GN1 .... *27*

5. Branch shortening *37*

6. Demographic history *40*

7. Genetic diversity *49*

8. GN1 relation to modern humans *52*

9. Genetic screening *61*

10. Mitochondrial captures *65*

11. Y chromosome captures *101*

12. ArchaicPlus nuclear capture ascertainment *109*

13. ArchaicPlus captures *115*

14. Genetic kinship *120*

15. Population affinities *126*

16. Split times *147*

17. Local ancestry inference *158*

1. Archaeological context

1.1 Fonds-de-Forêt (Trooz, Liège Province, Belgium)

The Bay Bonnet caves, also referred to as the Fonds-de-Forêt caves, are situated in the Magne Valley (50°35'35"N, 5°41'56"E, Municipality of Trooz, Province of Liège), a tributary valley of the Vesdre River. These two caves open on the left bank of the valley, approximately 1500 meters upstream from the confluence, and lie only about ten meters apart.

Initial exploration was carried out by Philippe-Charles Schmerling between 1830 and 1831, followed sporadically by various collectors until 1895. That year marked the beginning of systematic excavations by Ferdinand Tihon, who uncovered two human remains in the first cave (the more upstream one)[1]. He also provided the first stratigraphic descriptions of both caves. Following his work, numerous scholars and collectors continued investigations at the site, culminating in a brief excavation in 2003 focused on securing the cave (installation of protective grids)[2].

According to Tihon's observations, the Première Caverne of Fonds-de-Forêt comprises four distinct stratigraphic layers. The third layer stands out as the richest, containing most of the archaeological finds, including two human remains: a femur and an upper molar, which is now lost. This deposit—designated as Layer F—was later confirmed by geologist A. Rutot during his 1907 excavations[3], which also yielded bone retouchers which gave similar ages to those obtained on the Neandertal femur 41,900-44,300 cal BP (OxA-X-2767-13)[4,5].

The majority of the archaeological assemblage is composed of Quina Mousterian artifacts. Nevertheless, much of the archaeological material was found to be mixed across the stratigraphic layers, as well as between the two caves[6].

The femur (Fonds-de-Forêt 1) has been identified as Neandertal since its discovery[1]. The presence of carnivore tooth marks on the surface of the femur[7], in conjunction with the absence of other skeletal elements attributable to the same individual, suggests that the femur was likely transported into the cave by carnivores, possibly as part of scavenging behavior. It has been dated to 38,800 ± 900 (OxA-X-2767-13) and 39,500 ± 1,100 (OxA-38322)[5].

1.2 Grotte du Renne (Arcy-sur-Cure, Yonne, France)

Grotte du Renne, located at Arcy-sur-Cure in the Yonne department of Burgundy (47°35′27"N, 3°45'45"E), opens onto a limestone cliff on the right bank of the Cure River. This site forms part of a larger karstic complex comprising fifteen cavities, and lies approximately 250 meters upstream from the Grand Grotte, the region's most prominent decorated cave.

The site was first discovered by Pierre Poulain in 1939, but systematic excavation only began in the 1940s under the direction of André Leroi-Gourhan, until 1963. These excavations produced a detailed stratigraphy, yielding a remarkable sequence with more than a dozen archaeological layers[8,9]. Of special note are Layers X, IX, and VIII, which have been assigned to the Châtelperronian techno-complex. It is in these upper layers that the hominin remains were recovered, including over 64 remains, 38 of which could be attributed to Neandertals[10,11].

Radiocarbon dating of faunal and human remains from Layers VIII and IX suggests an occupation timeframe from 44,500 to 41,000 cal BP[12,13], or possibly 39,500 cal BP[14]. A cranial vault fragment from Layer X (AR-14), which was initially identified by ZooMs (Zooarchaeology by Mass Spectrometry), was directly dated to 40,680 and 42,335 cal BP[15], supporting the association between Neandertals and Châtelperronian material culture. Mitochondrial DNA was also recovered from AR-14, as well as from another cranial vault fragment (AR-30) identified in the same manner from Layer X[15].

In the inventory conducted in 2019 of the palaeoanthropological collection attributed to the Chatelperronian layers, a neonate’s ilium (AR-63) was identified. The geometric morphometrics analyses revealed that it fell outside known Neandertal variation, suggesting it might be a modern human[11]. To date, its taxonomic assignment remains unique within the remains unearthed from Layer X, with the rest of the remains being assigned to Neandertals. There have been concerns regarding possible post-depositional mixing layers, due in part to bioturbation and excavation methodology[16,17]. The most parsimonious hypothesis would correspond to AR-63 being intrusive from the overlaying Aurignacian layers, but further taphonomic and spatial studies have yet to be carried out to address this[11].

1.3 La Roche-à-Pierrot (Saint-Césaire, Charente-Maritime, France)

La Roche-à-Pierrot, commonly referred to as the Saint-Césaire rock shelter, is located near the village of Saint-Césaire in the Charente-Maritime department of southwestern France (45°44'56"N, 0°30'19"W), east of the Coran River, a tributary of the Charente. The site opens at the base of a limestone cliff and lies approximately 300 meters from the modern riverbed.

The site was discovered in 1976 by François Lévêque during a systematic regional survey and was subsequently excavated from 1976 to 1987 under his direction[18,19]. These excavations recovered a well-preserved stratified sequence of twelve levels, from basal Mousterian (Level I) to Châtelperronian and early Aurignacian layers. Of these, Level EJ is of particular significance due to the discovery of an almost complete skeleton (Saint-Césaire 1) in direct association with Châtelperronian lithic material including characteristic elements such as backed points and endscrapers, alongside bone tools, which

alongside Arcy-sur-Cure, challenged previous assumptions about the behavioral and cultural capabilities of late Neandertals[19,10]. The earliest thermoluminescence dates available for that layer, from 20 flints near the Saint Césaire 1 remains, ranged from 42,200 to 39,900 cal BP[20].

The remains of Saint Césaire 1 comprise a nearly complete cranium, mandible, postcranial skeleton, and teeth, and belong to a young adult individual (Saint-Césaire 1), which has been widely accepted as a late Neandertal[21]. The tibia from Saint Césaire 1 has been directly radiocarbon dated to 42,210 to 39,950 cal BP (OxA-18099), consistent with the dating of the Châtelperronian in Arcy-sur-Cure described above[13].

The possible post-depositional mixing of artifacts has been proposed, considering that micromorphological, taphonomic and typo-technological studies suggest disturbances[22,23], with complex site-formation processes at play[24]. Thus, it is contested whether the Saint-Césaire 1 remains can be associated with the Châtelperronian techno-complex.

1.4 Les Cottés (Saint-Pierre-de-Maillé, Vienne, France)

Les Cottés is located on the outskirts of the village of Saint-Pierre-de-Maillé, in the Vienne department of west-central France (46.694502° N, 0.842936° E). The interior of the cave was excavated over a century ago, while the deposits preserved just outside the entrance—along a continuous 13-meter-long section—were systematically excavated by M. Soressi and her team between 2006 and 2018.

Les Cottés preserves stratified archaeological layers from the late Middle Palaeolithic (Mousterian) and early Upper Palaeolithic (Châtelperronian, Protoaurignacian, and Aurignacian), all very rich in artifacts and skeletal remains[25–28]. The full archaeological sequence measures approximately 3 meters in thickness. Individual cultural layers are generally separated by sterile or low-density deposits ranging from a few centimeters to over 50 centimeters, depending on the specific area.

Radiometric dating of both bones and sediments indicates that the site was occupied from at least 45,000 to around 35,000 years ago[29,30]. The Neandertal tooth analyzed in this study (CTS-Z4-1514) was found in the Protoaurignacian layer (US 04 inf). It was piece-plotted using a hand-held computer connected to a total station and was excavated in 2008. The root of the tooth was directly radiocarbon dated to a calibrated age of 43,410–42,920 cal BP (±1σ) and 43,740–42,720 cal BP (±2σ) (MAMS-26196), using the IntCal13 calibration curve[31].

The association of this Neandertal tooth with a Protoaurignacian assemblage is both surprising and challenging, particularly given the relatively older age of the tooth compared to the average radiocarbon ages of faunal remains from the same layer, which range between ~41,000 and 37,000 cal BP[29].

Compound-specific nitrogen isotope analysis of collagen amino acids extracted from the root of the tooth revealed that this female Neandertal[31] exhibited exceptionally high $\partial 15N$ values, consistent with a diet primarily composed of terrestrial mammal meat[32].

1.5 Schmerling Cave (Flémalle, Liège Province, Belgium)

Located in the village of Les Awirs (50°35'28"N, 5°24'35"E, Municipality of Flémalle, Province of Liège), the Schmerling Cave—formerly known as “Trou Caheur” or “Deuxième Grotte d’Engis”—is part of a broader karstic system first documented by Philippe-Charles Schmerling [33]. This complex includes two main cave chambers and a gallery. Schmerling named the chambers sequentially from east to west as the “Première Grotte d’Engis” and the “Deuxième Grotte d’Engis”[33], while a third component, later called the “Troisième caverne d’Engis” by Dupont[34], was eventually interpreted as an extension of the second cave[35]. In 1939, the site was renamed “Grotte Schmerling” by the Chercheurs de la Wallonie, an association of dedicated amateur researchers, in recognition of Schmerling’s pioneering work.

Today, the cave entrance lies within the remnants of a heavily altered alum quarry, approximately 750 meters from the Meuse River. Repeated excavations during the 19th and 20th centuries have significantly modified the original morphology of the site, leaving only the rear portion of the cave intact.

Schmerling’s initial investigations began in the winter of 1829–1830 and led to the discovery of multiple human remains, most notably two cranial vaults—one belonging to an adult (Engis 1) and the other to a juvenile (Engis 2), which was also associated with isolated teeth and a maxillary fragment. Radiocarbon dating places the adult remains (Engis 1) in the Neolithic period (4590 ± 80 BP, OxA-746; 4920 ± 50 BP, Beta-154814)[36], likely linked to a collective burial context. The juvenile remains were found deeper within the cave, alongside a tooth attributed to an Elephantidae. Even if Engis 2 is the very first Neandertal ever found, the child was not attributed to this species until over a century after its discovery[37]. Based on the dental histology, the age of the child has been estimated around 3 years old. Several radiocarbon dates have been obtained on the Neandertal remains. The latest and most accurate gives an age between 41,600-47,800 cal BP to Engis 2 (OxA-38394)[5]. No lithic artifact can be directly associated with Engis 2.

At the time, Schmerling interpreted the co-presence of human, faunal, and lithic material as evidence of their contemporaneity, although stratigraphic or sedimentological distinctions were not taken into account[38]. This conclusion was later challenged, sparking renewed scientific interest. Excavations resumed nearly four decades later under the direction of E. Dupont in 1868[34]. Further exploration occurred at various times throughout the 19th century[39,40], at the beginning of the 20th century[6], and

again between 1907 and 1956[41,42]. Despite this extensive research history, no additional Neandertal remains have been identified at the site.

1.6 Spy (Jemeppe-sur-Sambre, Namur Province, Belgium)

Located in the Orneau Valley in the Mosan Basin, the cave of Betche aux Rotches in Spy (50°28'49"N, 4°40'28"E, Municipality of Jemeppe-sur-Sambre, Province of Namur) is one of the richest prehistoric sites in Belgium. Numerous field operations have been carried out at the site since the 19th century. They include official excavations as well as collections by amateurs, which have both yielded Neandertal remains[43,44]. The Neandertal fossils represent three different individuals, two adult partial skeletons Spy I and II, first recovered in 1886[40], and the ca. 1.5-year-old Spy VI child represented by two mandibular fragments and four teeth isolated among the fauna from the 1952-1954 excavations[45]. The Spy Neandertals have been directly dated several times (see[46,5]). The most recent dates were obtained by dating the hydroxyproline of the collagen of five Neandertal bones and teeth; one of them was too young due to contamination with recent animal collagen[5]. Calibrating the others using IntCal20[47] in OxCal 4.4[48], after combining two dates on a maxillary bone and associated tooth, yielded a range between 54,320 and 42,160 years calBP for the Spy Neandertals. The two adult Neandertals were found in the deepest "fauna-bearing level" of the site's terrace[49] whereas the Spy VI child comes from a reworked context. Both Middle Palaeolithic and Lincombian-Ranisian-Jerzmanowician (LRJ) artifacts were recovered at Spy, however the early date of the discoveries has resulted in a poor understanding of the archaeostratigraphy of the site[50]. Radiocarbon dates on the LRJ at Ranis, Germany, overlap with the age of the Spy Neandertals although the association of the LRJ to modern human remains at Ranis does not make a Neandertal – LRJ association the most parsimonious hypothesis at Spy[51]. On another hand, the overlap of hydroxyproline radiocarbon dates of Mousterian bone retouchers from other Belgian sites (from 53,000 to 39,900 years calBP)[4] with those of the Spy Neandertals supports the possibility of a Mousterian context for the latter.

One of the Spy Neandertal teeth (upper right M3 Spy 94a) had previously been sampled for palaeogenomic study[31]. In the present study, seven skeletal elements were analyzed in addition to further sequencing of Spy 94a. Altogether, they belong to various parts of the skeleton and include cranio-facial elements (Spy 94a and upper left I1 Spy 92b), upper limb bones (right scapula Spy 572a, right humeri Spy 5A and 14B, and right hand phalanx Spy 430a), and lower limb bones (right femur Spy 8 and left femur Spy 16). All of them are mature elements and are associated with the two adult Neandertals Spy I and II. Some of the individual associations proposed[52] have to be taken cautiously given the lack of field data due to the early date of the excavations at Spy[53,54]. However, we can securely propose that the two teeth Spy 92b and 94a belong to two different individuals because of their refitting

with facial elements of each of the two adult Neandertals[46]. The same goes for the two right humeri Spy 5A and 14B that preserve overlapping anatomical portions.

1.7 Troisième caverne of Goyet (Gesves, Namur Province, Belgium)

The Troisième caverne of Goyet (50°26′39″N, 5°0′51″E, Municipality of Gesves, Province of Namur) is located in the Samson Valley, a tributary of the Meuse River. It was excavated on multiple occasions in the second half of the 19th century and the beginning of the 20th century, as well as at the end of the 20th century. The main excavations were conducted by E. Dupont in 1868 who identified five "fauna-bearing levels" at the site[55]. Subsequent work identified rich archaeological evidence of human occupations from the Mousterian, the LRJ, as well as from later periods of the Upper Palaeolithic, Neolithic, and historical times[6,56,57]. Since the early excavations yielded mixed materials from different periods in the same layers (see[58]), an interdisciplinary reassessment of the faunal and human collections from the site was initiated in 2008. Combining the results of morphometrics, taphonomy, stable isotope, dating, and genetic analyses, this project has resulted in the identification of both Neandertal and Upper Palaeolithic human remains[59]. The Goyet Neandertal collection is the largest assemblage of Neandertal remains in Northern Europe and shows evidence of cannibalism and of bones used as retouchers[57]. Several of the Neandertal remains have been directly dated by AMS radiocarbon dating[57] and provide a range between 41,000 and 45,000 years calBP when calibrated with the IntCal20 calibration curve[47]. As is the case for Spy (see above), this makes an attribution of the Goyet Neandertals to a Middle Palaeolithic context the most parsimonious hypothesis.

The initial inventory of the Goyet Neandertal collection included 99 skeletal remains representing a minimum of four adult/adolescent individuals and one child. The child is represented by a tooth (Goyet 1424-3D), the only human specimen found amongst the material from A. de Loë's early 20th century excavations – all the other ones come from E. Dupont's excavations at the site[57]. Mitochondrial DNA had been successfully extracted from 10 of the adult/adolescent remains (left parietal fragment Goyet C5-1, left rib fragment Goyet Q119-2, right femur fragments Goyet Q56-1 and Q57-2, and right and left tibia fragments Goyet Q55-4, Q57-1, Q57-3, Q305-4, Q305-7, and Q374a-1)[57]. A preliminary palaeogenomic analysis was also conducted for the femur fragment Goyet Q56-1[31]. In the present study, all 10 previously sampled specimens were analyzed further along with seven additional ones. They include four adult/adolescent elements (a lower left second premolar – Goyet 2878-2D, two right rib fragments – Goyet Q376-9 and Q376-25, and a right tibia fragment – Goyet Q54-4) as well as the child's lower left second incisor Goyet 1424-3D. The last two are newly identified Neandertal remains representing a child and a neonate: left clavicle Goyet D183-4 and right femur Goyet Q305-1, respectively[60].

1.8 Trou de l'Abîme (Couvin, Namur Province, Belgium)

The Trou de l'Abîme site, also known as the Cavernes du Trou de l'Abîme, is located in the municipality of Couvin (50°03'02"N, 4°29'52"E), in the province of Namur, Wallonia, Belgium. The site opens on the northern slope of the Eau Noire Valley, a tributary of the Meuse River, and lies within a karstic limestone formation at the base of a steep cliff of more than 100m.

Initial exploration of the cave was undertaken in 1888 by P. Gérard, but the site remained relatively understudied until the 1980s. A renewed archaeological campaign between 1984 and 1987 led to the identification of a significant Middle Palaeolithic sequence including Mousterian lithic artifacts and faunal remains[61,62]. Layer II is the principal archaeological horizon at the site and contains a rich Mousterian assemblage, including flakes and sidescrapers, two of which exhibit bifacial retouch and one produced on a laminar blank[63]. Burned bones in this layer, interpreted as deriving from a reworked hearth, support the hypothesis of repeated human occupation. The faunal assemblage includes both large and small mammals, with *Equus* and *Bos* among the most prominently represented taxa[64].

A series of radiocarbon dates on faunal and modified remains from Layer II indicates an occupation during the middle of Marine Isotope Stage (MIS) 3. Dates include >45,700 cal BP (46,820 ± 3,290 BP; LV-2559)[61] around 49,800 to 44,900 cal BP on an *Equus sp.* tooth (44,500 +1,100/-800 BP; GrA-40444)[65], and 52,500 to 43,100 cal BP on a *Bos sp.* phalange bearing cut marks (43,600 ± 1,900 BP; OxA-34120)[4]. A bone retoucher made from an *Equus* tooth from the same context yielded a date of 52,000 and 43,100 cal BP (43,400 ± 1,800 BP; OxA-34121[5], situating the site within the later Mousterian timeframe in Belgium.

It was during these excavations in 1984 that a human tooth (G6-0083) was discovered. It was identified as a lower right deciduous second molar (RdM2), and later attributed to a Neandertal child of approximately 5–6 years of age, with enamel development patterns and wear stages consistent with other European Neandertal deciduous teeth[65].

1.9 Trou Magrite (Pont-à-Lesse, Namur Province, Belgium)

The site of Trou Magrite is located at Pont-à-Lesse in the Lesse Valley (50°13'19"N, 4°54'49"E, Municipality of Dinant, Province of Namur). It was first excavated by E. Dupont in 1867[66], and the most recent fieldwork at the site was conducted by L. Straus and M. Otte in 1991-92[67]. Trou Magrite yielded rich lithic assemblages, osseous artifacts, mobiliary art, and numerous faunal remains. The archaeological record covers a broad time range spanning from the Middle and Upper Palaeolithic to the Mesolithic, Neolithic, and Iron Age. An important Middle Palaeolithic collection is present, probably representing several occupation phases during the Late Pleistocene as the 1991-92 fieldwork

uncovered deposits yielding Mousterian materials that may correspond to MIS 5 to 3[67]. Correlations with E. Dupont's work are impossible in part because the materials from the different "fauna-bearing levels" that he defined in the field were mixed post-excavation[68].

Several human remains were recovered and identified as Palaeolithic humans by E. Dupont, but they have been only partially published thus far[69]. In 2015, we initiated an interdisciplinary re-assessment of the collections from E. Dupont's work at Trou Magrite in order to update the inventory of human remains already identified at the Royal Belgian Institute of Natural Sciences and to check for the presence of human remains that may have been previously overlooked. This resulted in the identification of two Neandertal fossils that were isolated from the faunal collection: an upper right canine (TM 2419-10) and a left femur diaphysis (TM 2422-36)[70]. They represent an adult and a neonate, respectively. While no endogenous DNA was recovered from the tooth, the palaeogenetic analysis of the TM 2422-36 femur is included in the present study. Despite their lack of associated context, we have not attempted to directly radiocarbon date the Trou Magrite Neandertal remains as a precaution for the preservation of such small elements and because of signs of potential poor preservation of biological components and sources of contamination (e.g., varnish).

1.10 Walou Cave (Trooz, Liège Province, Belgium)

The cave of Walou (50°35'26"N, 5°41'40"E, Municipality of Trooz, Province of Liège), was first discovered by M. Briffoz in 1965. It has been excavated in the framework of distinct research projects, first from 1985 to 1990[71] and then from 1996 to 2004, revealing numerous successive prehistoric occupations[63,72,73] . In 1997, a Neandertal permanent lower left third premolar, WA97 G24–120**,** was discovered in Layer CI-8[74,73,75]. This layer yielded the main Mousterian occupation of the site with ca. 1280 lithic objects[72]. Most of the artifacts are blank flakes, as well as secondary by-products and splinters. The core reduction technique is essentially unifacial, unipolar and centripetal, with an important production of backed pieces. Some rare Levallois cores with débitage of a preferential flake were also found. There are very few retouched tools (n = 49), from which the most frequent are sidescrapers. The use of backed flakes (natural or "éclats débordants") as tool support is typical for this occupation.

The initial distribution of the assemblage was disturbed by solifluction processes that affected the layer after human occupation. The large number of bear and hyena bones also suggests reworkings. No human action such as cut marks was observed on the animal bones found in association with the human tooth and the lithic artifact.

The detailed study of the premolar led to its attribution to a Neandertal individual [75]. The dental morphology and metrics of the Walou P_3 plot within the Neandertal distribution are significantly distinct

from recent modern humans. Isotopic analyses also tend to portray Walou as a Neandertal, with $\delta^{13}C$= -22.6‰ and $\delta^{15}N$= 9.3‰, which are similar to other Neandertals from Northern Europe[75].

No direct date is so far available on this tooth. However, the stratigraphic sequence benefits from a coherent chronostratigraphic framework, as a result of tephrostratigraphy (Rocourt and Laacher See Tephras) and the excellent correlation with the loess sequences of Middle Belgium, strengthened by radiocarbon, TL and ESR dating[76,63,73]. The Neandertal tooth is, therefore, highly likely dated to ca. 38-40 ka BP based on this contextual and chronological data[63,73,77].

References

1. Tihon, F. Les cavernes préhistoriques de la vallée de la Vesdre. Fouilles à Fond-de-Forêt (2ème article). *Ann Soci Archéo Brux.* 145–173 (1989).
2. Toussaint, M. & Pirson, S. Trooz/Forêt : sondages d'évaluation aux grottes de Fonds de Forêt. *Chron. Archéologie Wallonne* 99–103 (2004).
3. Rutot, A. Résultat des fouilles effectuées dans la caverne de Fond-de-Forêt (Province de Liége). *Bull. Inst. Archéologique Liégeois XXXIX* 151–160 (1909).
4. Abrams, G. *et al.* Investigating the co-occurrence of Neanderthals and modern humans in Belgium through direct radiocarbon dating of bone implements. *J. Hum. Evol.* **186**, 103471 (2024).
5. Devièse, T. *et al.* Reevaluating the timing of Neanderthal disappearance in Northwest Europe. *Proc. Natl. Acad. Sci.* **118**, e2022466118 (2021).
6. Ulrix-Closset, M. *Le Paléolithique Moyen Dans Le Bassin Mosan En Belgique*. (Universa, 1975).
7. Camarós, E. *et al.* Hunted or Scavenged Neanderthals? Taphonomic Approach to Hominin Fossils with Carnivore Damage. *Int. J. Osteoarchaeol.* **27**, 606–620 (2017).
8. Leroi-Gourhan, A. *Etude Des Restes Humains Fossiles Provenant Des Grottes d'Arcy-Sur-Cure*. (Masson, 1958).
9. Leroi-Gourhan, A. & Leroi-Gourhan, A. Chronologie des grottes d'Arcy-sur-Cure (Yonne). in *Chronologie des grottes d'Arcy-sur-Cure (Yonne)* 1–64 (Gallia Préhistoire, 1964).
10. Hublin, J.-J., Spoor, F., Braun, M., Zonneveld, F. & Condemi, S. A late Neanderthal associated with Upper Palaeolithic artefacts. *Nature* **381**, 224–226 (1996).
11. Gicqueau, A. *et al.* Anatomically modern human in the Châtelperronian hominin collection from the Grotte du Renne (Arcy-sur-Cure, Northeast France). *Sci. Rep.* **13**, 12682 (2023).
12. Higham, T. *et al.* Chronology of the Grotte du Renne (France) and implications for the context of ornaments and human remains within the Châtelperronian. *Proc. Natl. Acad. Sci.* **107**, 20234–20239 (2010).

13. Hublin, J.-J. *et al.* Radiocarbon dates from the Grotte du Renne and Saint-Césaire support a Neandertal origin for the Châtelperronian. *Proc. Natl. Acad. Sci.* **109**, 18743–18748 (2012).
14. Banks, W. & d'Errico, F. La chronologie des niveaux d'occupation châtelperroniens de la grotte du Renne. In Le Châtelperronien de la grotte du Renne (Arcy-sur-Cure, Yonne, France). in 83–99 (PALEO: Revue d'Archéologie Préhistorique., 2019).
15. Welker, F. *et al.* Palaeoproteomic evidence identifies archaic hominins associated with the Châtelperronian at the Grotte du Renne. *Proc. Natl. Acad. Sci.* **113**, 11162–11167 (2016).
16. Mellars, P. Neanderthal symbolism and ornament manufacture: The bursting of a bubble? *Proc. Natl. Acad. Sci.* **107**, 20147–20148 (2010).
17. Caron, F., d'Errico, F., Del Moral, P., Santos, F. & Zilhão, J. The Reality of Neandertal Symbolic Behavior at the Grotte du Renne, Arcy-sur-Cure, France. *PLoS ONE* **6**, e21545 (2011).
18. Lévêque, F. & Vandermeersch, B. *Découverte de Restes Humains Dans Un Niveau Castelperronien à Saint-Césaire (Charente-Maritime).* (1980).
19. Lévêque, F., Backer, A. M. & Guilbaud, M. *Context of a Late Neandertal: Implications of Multidisciplinary Research for the Transition to Upper Paleolithic Adaptations at Saint-Césaire, Charante-Maritime, France.* (1993).
20. Mercier, N. *et al.* Thermoluminescence dating of the late Neanderthal remains from Saint-Césaire. *Nature* **351**, 737–739 (1991).
21. Trinkaus, E., Churchill, S. E., Ruff, C. B. & Vandermeersch, B. Long Bone Shaft Robusticity and Body Proportions of the Saint-Césaire 1 Châtelperronian Neanderthal. *J. Archaeol. Sci.* **26**, 753–773 (1999).
22. Gravina, B. *et al.* No Reliable Evidence for a Neanderthal-Châtelperronian Association at La Roche-à-Pierrot, Saint-Césaire. *Sci. Rep.* **8**, 15134 (2018).
23. Galland, A., Queffelec, A., Caux, S. & Bordes, J.-G. Quantifying lithic surface alterations using confocal microscopy and its relevance for exploring the Châtelperronian at La Roche-à-Pierrot (Saint-Césaire, France). *J. Archaeol. Sci.* **104**, 45–55 (2019).
24. Todisco, D. *et al.* A multiscalar and multiproxy geoarchaeological approach to site formation processes at the Middle and Upper Palaeolithic site of La Roche-à-Pierrot, Saint-Césaire, France. *Quat. Sci. Rev.* **315**, 108218 (2023).
25. Roussel, M. & Soressi, M. Une nouvelle séquence du Paléolithique supérieur ancien aux marges sud-ouest du Bassin parisien: les Cottés dans la Vienne. in *Le Paléolithique supérieur ancien de l'Europe du Nord-Ouest. Réflexions et synthèses à partir d'un projet collectif de recherche sur le centre et le sud du Bassin parisien; actes du colloque de Sens (15-18 Avril 2009)* 283–298 (Société Préhistorique Française, 2013).
26. Rendu, W. *et al.* Subsistence strategy changes during the Middle to Upper Paleolithic transition reveals specific adaptations of Human Populations to their environment. *Sci. Rep.* **9**, (2019).

27. Porter, S. T., Roussel, M. & Soressi, M. A Comparison of Châtelperronian and Protoaurignacian Core Technology Using Data Derived from 3D Models. *J. Comput. Appl. Archaeol.* **2**, 41–55 (2019).
28. Falcucci, A., Peresani, M., Roussel, M., Normand, C. & Soressi, M. What's the point? Retouched bladelet variability in the Protoaurignacian. Results from Fumane, Isturitz, and Les Cottés. *Archaeol. Anthropol. Sci.* **10**, 539–554 (2018).
29. Talamo, S., Soressi, M., Roussel, M., Richards, M. & Hublin, J.-J. A radiocarbon chronology for the complete Middle to Upper Palaeolithic transitional sequence of Les Cottés (France). *J. Archaeol. Sci.* **39**, 175–183 (2012).
30. Jacobs, Z., Li, B., Jankowski, N. & Soressi, M. Testing of a single grain OSL chronology across the Middle to Upper Palaeolithic transition at Les Cottés (France). *J. Archaeol. Sci.* **54**, 110–122 (2015).
31. Hajdinjak, M. *et al.* Reconstructing the genetic history of late Neanderthals. *Nature* **555**, 652–656 (2018).
32. Jaouen, K. *et al.* Exceptionally high $\delta^{15}N$ values in collagen single amino acids confirm Neandertals as high-trophic level carnivores. *Proc. Natl. Acad. Sci.* **116**, 4928–4933 (2019).
33. Schmerling, P.-C. *Recherches Sur Les Ossemens Fossiles Découverts Dans Les Cavernes de La Province de Liège*. vol. Premier volume (P.-J. Collardin, Liège, 1833).
34. Dupont, É. Sur une nouvelle exploration des cavernes d'Engis. *Bull. Académie R. Sci. Lett. B.-arts Belg.* **2e série**, 504–510 (1872).
35. Vandebosch, A. La grotte Schmerling à Engis. Bulletin des Chercheurs de la Wallonie. **XV**, 558–563 (1952).
36. Toussaint, M. & Pirson, S. Neandertal Studies in Belgium: 2000–2005. *Period Biol* **108**, (2006).
37. Fraipont, C. *Les Hommes Fossiles d'Engis*. (Masson et Cie, Paris, 1936).
38. Toussaint, M. & Pirson, S. Aperçu historique des recherches concernant l'homme préhistorique dans le karst belge aux XIXe et XXe siècles : archéologie, géologie, paléoanthropologie, paléontologie, datations, in: Evin, J. (Ed.). in *Un siècle de construction du discours scientifique en préhistoire.* vol. 2 117–142 (Société préhistorique française, 2007).
39. Fraipont, J. Nouvelle exploration des cavernes d'Engis. *Ann. Société Géologique Belg.* 187–191 (1885).
40. Fraipont, J. La poterie en Belgique à l'âge du Mammouth. Ire partie. La poterie de la grotte d'Engis. *Rev. Anthropol.* **3e série**, 385–399 (1887).
41. Vandebosch, A. Les grottes d'Engis. *Bull. Cherch. Wallonie* 121–125 (1939).
42. Destexhe-Jamotte, J. La grotte Schmerling à Engis (province de Liège). *Bull. Société Belg. Géologiques Archéologiques Cherch. Wallonie* **16**, 105–127 (1957).
43. Semal, P. *et al.* History of excavations, discoveries and collections. in *Spy cave. 125 years of multidisciplinary research at the Betche aux Rotches (Jemeppe-sur-Sambre, Province of Namur,*

Belgium vol. Volume 1 13–39 (Royal Belgian Institute of Natural Sciences, Royal Belgian Society of Anthropology and Praehistory & NESPOS Society, Anthropologica et Præhistorica, Brussels, 2013).

44. Rougier, H., Crevecoeur, I., Decerf, M., Jungels, C. & Semal, P. Mise au jour d'une nouvelle partie de la collection de François Beaufays (dit " l'Horloger ") contenant des vestiges humains de Spy (Prov. de Namur, BE). *Notae Praehistoricae* **43**, 43–51 (2023).
45. Crevecoeur, I. *et al.* The Spy VI child: A newly discovered Neandertal infant. *J. Hum. Evol.* **59**, 641–656 (2010).
46. Semal, P. *et al.* New data on the late Neandertals: Direct dating of the Belgian Spy fossils. *Am. J. Phys. Anthropol.* **138**, 421–428 (2009).
47. Reimer, P. J. *et al.* The IntCal20 Northern Hemisphere Radiocarbon Age Calibration Curve (0–55 cal kBP). *Radiocarbon* **62**, 725–757 (2020).
48. Bronk Ramsey, C. Bayesian Analysis of Radiocarbon Dates. *Radiocarbon* **51**, 337–360 (2009).
49. Fraipont, J. & Lohest, M. La Race humaine de Néanderthal ou de Canstadt en Belgique. Recherches ethnologiques sur des ossements humains, découverts dans des dépôts quaternaires d'une grotte à Spy et détermination de leur âge géologique. *Arch. Biol. (Liege)* **7**, 587–757 (1887).
50. Pirson, S. *et al.* The stratigraphy of Spy cave. A review of the available lithostratigraphic and archaeostratigraphic informatio. in *Spy cave. 125 years of multidisciplinary research at the Betche aux Rotches (Jemeppe-sur-Sambre, Province of Namur, Belgium)* vol. Volume 1 91–131 (Royal Belgian Institute of Natural Sciences, Royal Belgian Society of Anthropology and Praehistory & NESPOS Society, Anthropologica et Præhistorica, Brussels, 2013).
51. Mylopotamitaki, D. *et al.* Homo sapiens reached the higher latitudes of Europe by 45,000 years ago. *Nature* **626**, 341–346 (2024).
52. Fraipont, J. & Lohest, M. La Race humaine de Néanderthal ou de Canstadt, en Belgique. Recherches ethnologiques sur des ossements humains, découverts dans des dépôts quaternaires d'une grotte à Spy et détermination de leur âge géologique. Note préliminaire. *Bull. Académie R. Sci. Belg.* **12**, 741–784 (1886).
53. Hrdlička, A. *The Skeletal Remains of Early Man*. (Smithsonian Miscellaneous collections, Washington, 1930).
54. Rougier, H. *et al.* Collections de la Grotte de Spy : (re)découvertes et inventaire anthropologique. *Notae Praehistoricae* **24**, 181–190 (2004).
55. Dupont, É. *L'Homme Pendant Les Âges de La Pierre Dans Les Environs de Dinant-Sur-Meuse*. (C. Muquardt Ed., Bruxelles, 1872).
56. Flas, D. The Middle to Upper Paleolithic transition in Northern Europe: the Lincombian-Ranisian-Jerzmanowician and the issue of acculturation of the last Neanderthals. *World Archaeol.* **43**, 605–627 (2011).

57. Rougier, H. *et al.* Neandertal cannibalism and Neandertal bones used as tools in Northern Europe. *Sci. Rep.* **6**, 29005 (2016).
58. Germonpré, M. A reconstruction of the spatial distribution of the faunal remains from Goyet, Belgium. *Notae Praehistoricae* **21**, 57–65 (2001).
59. Rougier, H. *et al.* The Troisième caverne of Goyet (Belgium): An exceptional site with both Neandertal and Upper Paleolithic human remains. in vol. 5 211 (2016).
60. Fotiadou, C. M. *et al.* Mitochondrial DNA insights into the demographic history of late Neanderthals. *Proc. Natl. Acad. Sci. USA* (in prep.).
61. Cattelain, P., Otte, M. & Ulrix-Closset, M. Les "Cavernes de l'Abîme" à Couvin. *Notae Praehistoricae* **6**, 15–28 (1986).
62. Cattelain, P. *et al.* Le Trou de l'Abîme à Couvin. in *Le Paléolithique moyen en Belgique. Mélanges Marguerite Ulrix-Closset* 297–304 (ERAUL, Liège, 2011).
63. Pirson, S., Draily, C. & Toussaint, M. *La Grotte Walou à Trooz (Belgique). Fouilles de 1996 à 2004*. vol. 1: les sciences de la terre (Namur, 2011).
64. Abrams, G. & Cattelain, P. Le Trou de l'Abîme à Couvin (Prov. Namur, Belgique) Bilan archéozoologique préliminaire de la couche II (fouilles 1984-1987). *Archéo-Situla* **34**, 33-40. (2014).
65. Toussaint, M. *et al.* The Neandertal lower right deciduous second molar from Trou de l'Abîme at Couvin, Belgium. *J. Hum. Evol.* **58**, 56–67 (2010).
66. Dupont, É. Découverte d'objets gravés et sculptés dans le Trou Magrite à Pont-à-Lesse. *Bull. Académie R. Sci. Lett. B.-arts Belg.* **24**, 129–132 (1867).
67. Otte, M. & Straus, L. G. *Le Trou Magrite : Fouilles 1991-92. Résurrection d'un Site Classique En Wallonie.* (ERAUL 69, Liège, 1995).
68. Jimenez, E.-L., Smolderen, A., Jadin, I. & Germonpré, M. Exhumation de la collection faunique d'Édouard Dupont provenant du Trou Magrite (Pont-à-Lesse) Quelles données et quelles perspectives pour une collection du XIXe siècle ? *Notae Praehistoricae* **36/2016**, 167–190 (2016).
69. Twiesselmann, F. Belgium. in *Catalogue of Fossil Hominids - Part 2: Europe* 5–13 (Trustees of the British Museum (Natural History), London, 1971).
70. Rougier, H. *et al.* New Neandertal remains from Trou Magrite, Belgium. in *PESHE* vol. 8 166 (2019).
71. Dewez, M. *et al. Recherches à La Grotte Walou à Trooz (Province de Liège, Belgique), Premier Rapport de Fouille.* (1993).
72. Draily, C. *La Grotte Walou à Trooz (Belgique). Fouilles de 1996 à 2004*. vol. 3: l'archéologie (Namur, 2011).
73. Draily, C., Toussaint, M. & Pirson, S. *La Grotte Walou à Trooz (Belgique). Fouilles de 1996 à 2004*. vol. 2: les sciences de la vie et les datations (Namur, 2011).

74. Draily, C., Yernaux, G., Cordy, J. M. & Toussaint, M. Découverte d'une dent humaine dans une couche moustérienne de la grotte Walou à Trooz (fouille 1997). *Notae Praehistoricae* **19**, (1999).
75. Toussaint, M. *et al.* The Late Neandertal permanent lower left third premolar from Walou Cave (Trooz, Belgium) and its context. *Am. J. Phys. Anthropol.* **164**, 193–202 (2017).
76. Pirson, S., Court-Picon, M., Damblon, F. & Haesaerts, P. Belgian cave entrance and rock-shelter sequences as palaeoenvironmental data recorders: the example of Walou cave. *Geol. Belg.* **94**, 275–286 (2006).
77. Pirson, S. *et al.* Chronostratigraphic context of the Middle to Upper Palaeolithic transition: Recent data from Belgium. *Quat. Int.* **259**, 78–94 (2012).

2. The Goyet Q56-1 or Goyet Neandertal 1 (GN1) high-coverage genome

2.1 Data generation and processing

After the initial screening (described in Supplementary Information 2), we selected a Goyet Q56-1 lysate (Lys1891) which yielded a library with the highest percentage of endogenous DNA (37.63%), high complexity (with an estimated genomic coverage of 12.41-fold) and low levels of present-day human DNA contamination (library A13725, Supplementary Table 10, Supplementary Data Table 1) to generate two additional single-stranded non-UDG treated libraries for deeper sequencing (A18175 and A18477, respectively) using the same library preparation approach as described in Supplementary Information Section 10[1]. Following amplification and double indexing, molecules <35 base pairs (bp) were removed from the libraries A13725, A18175 and A18477 by gel-excision (size selection) as described[1]. After heteroduplex removal, we measured DNA concentration in the resulting libraries (F9127, F9128 and F9129) using Agilent 2100 Bioanalyzer DNA 1000 chip, and pooled them together for deeper sequencing on an Illumina HiSeqX platform at SciLife, Sweden.

We sequenced the pool of three Goyet Q56-1 libraries together with four ΦX174 spiked-in libraries[2] on a total of 17 lanes of Illumina HiSeqX in a double index configuration (2x76 cycles)[3]. Base calling was done using Bustard (Illumina). We merged forward and reverse reads and trimmed Illumina adaptors using *leeHom*[4]. We used the distinct combinations of two seven base pair indices at the ends of the sequences to identify which sequences belonged to each library and demultiplexed the data with *jivebunny* (version 0.2.1, https://github.com/mpieva/jivebunny).

We aligned the sequences assigned to each library to the human reference genome (hs37d5 or hg19) using BWA (version 0.5.10)[5] with ancient DNA parameters (-n 0.01 -o 2 -l 16500)[6]. Next, we removed duplicate sequences for each library using bam-rmdup (https://github.com/mpieva/biohazard-tools/), and filtered the unique reads for a minimum mapping quality of 25, base quality 20 and sequence length of at least 35 bp.

We estimated present-day human DNA contamination levels in each library by using five complementary approaches:

- AuthentiCT, a Hidden Markov Model that uses damage patterns as hallmarks for endogenous DNA[7]);
- A maximum likelihood divergence-based method, which identifies contaminants based on the excess of derived alleles[6,8]);
- The 'conditional substitutions' approach, which tests for the presence of differentially deaminated groups of sequences within a given library[2,9];

- An excess of sequences mapping to the Y chromosome in female individuals[8,10];
- By direct comparison of all vs deaminated sequences using an f-statistics of the form *D(Goyet Q56-1all, Goyet Q56-1deaminated; Test, Mbuti)* where "*Test*" is a present-day population from the Simons Genome Diversity Project (SGDP)[11] and three Mbuti individuals from SGDP are used as an outgroup.

While most of the approaches yielded contamination estimates of up to 3%, the estimates using AuthentiCT are significantly higher, with at least 10% of estimated contaminant sequences. These estimates, as well as a lack of bias in the f-statistics results (Supplementary Data Table 1) point towards AuthentiCT substantially over-estimating the levels of present-day human DNA contamination.

Supplementary Table 10 Summary statistics of the newly sequenced libraries for Goyet Q56-1.

Heteroduplex fixed, gel-excised library ID	Index library ID	AuthentiCT Contamination estimates (standard error)	Philip Johnson Contamination estimates (standard error)	Conditional Substitution Contamination estimates (standard error)	Y chromosome excess estimates (standard error)
F9127	A13725	**9.0%** (± 0.7%)	**0.19%** (± 0.02%)	**2.63%** (± 0.012%)	**3.4%** (± 0.026%)
F9128	A18175	**10.9%** (± 0.6%)	**0.16%** (± 0.03%)	**2.75%** (± 0.025%)	**3.34%** (± 0.026%)
F9129	A18477	**10.7%** (± 0.6%)	**0.18%** (± 0.03%)	**2.56%** (± 0.025%)	**3.41 %** (± 0.031%)

2.2 Additional quality controls of the sequenced libraries

As additional quality controls for each library, we verified that (1) the coverage distribution was uniform throughout the mappability tracks map35_100% described[10] (Supplementary Figure 1), (2) the sequence length distribution and the terminal substitution patterns were comparable to those of other archaic genomes sequenced to date (Supplementary Figures 2 and 3), and (3) the lineage assignment[2] matched our expectations for a Late Neandertal, with a clear support for the Neandertal branch, and with more shared derived alleles with the ~45,000-year-old Vindija 33.19 Neandertal than the two other high-quality Neandertal genomes (Supplementary Figure 4).

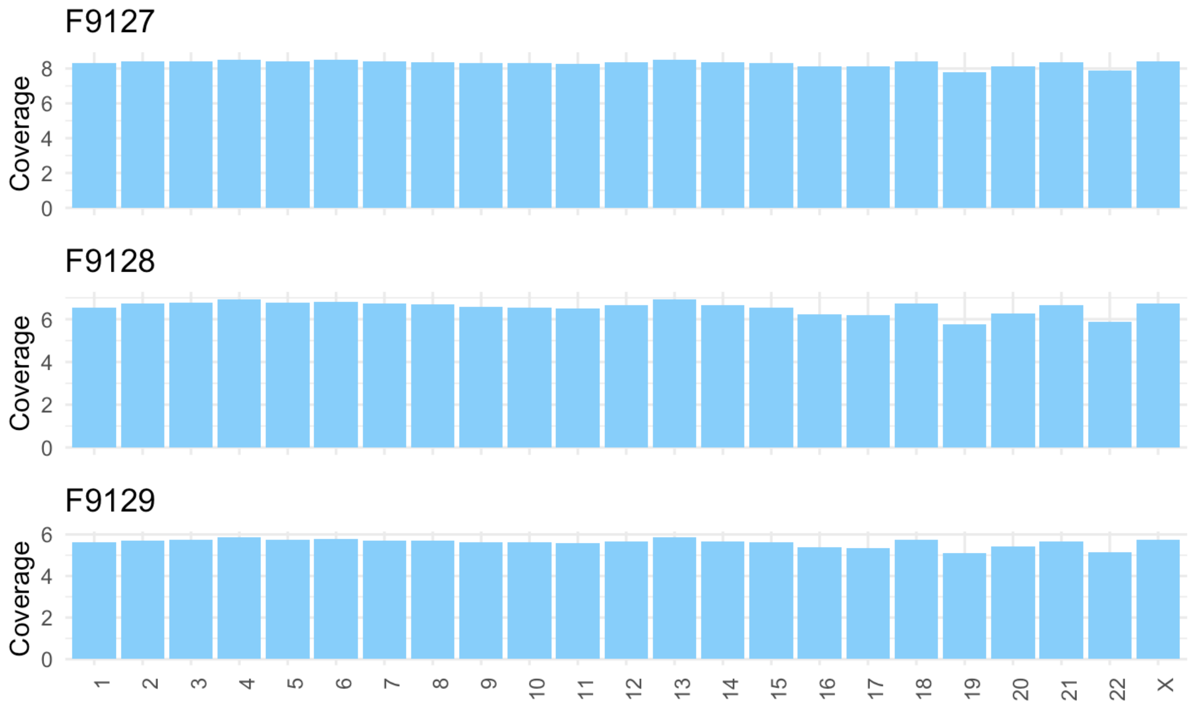

Supplementary Figure 1 The average depth of coverage by chromosome and library, on the genomic regions defined by mappability track map35_100% [10].

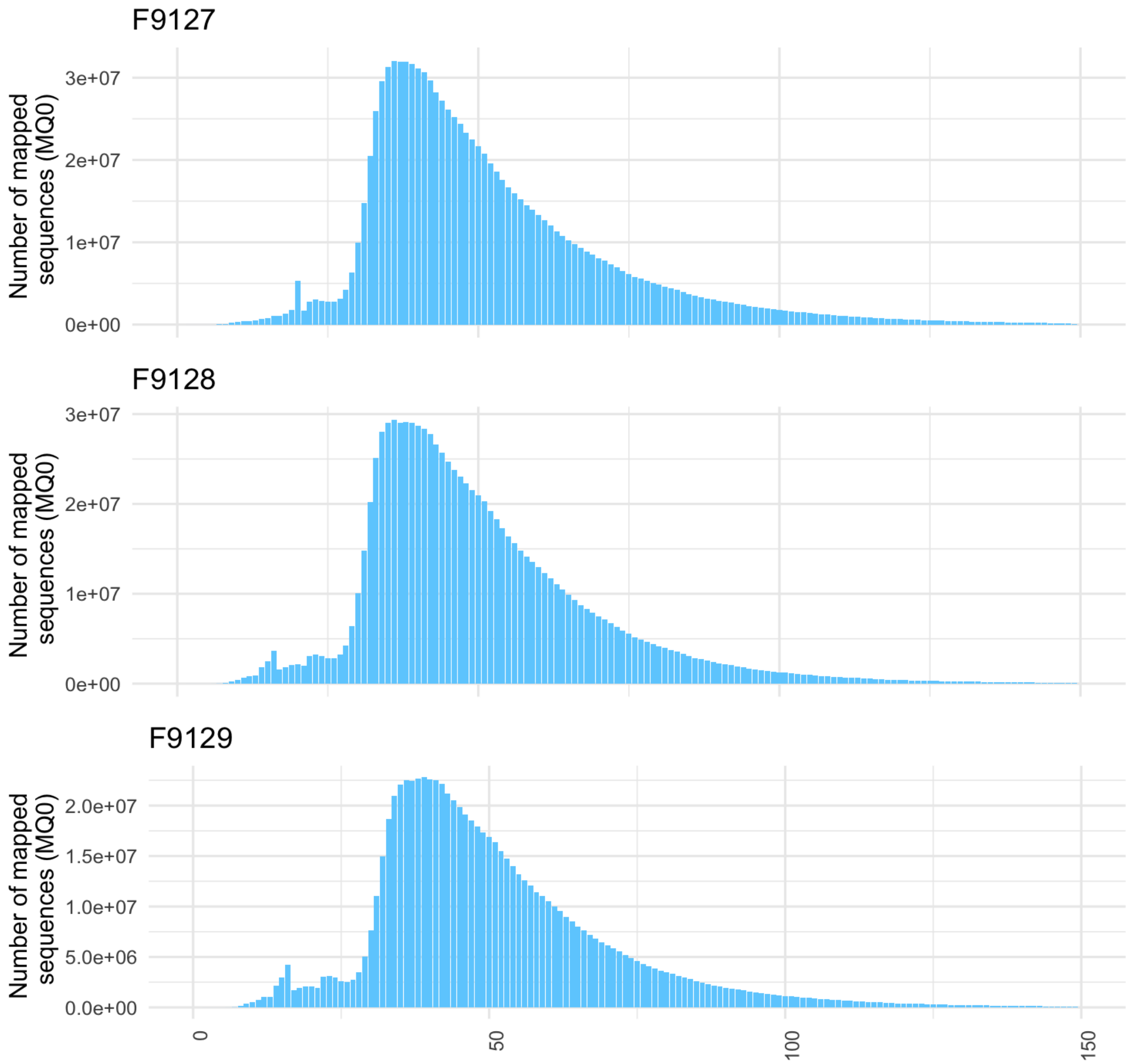

Supplementary Figure 2 Fragment length distribution per each deeply sequenced library (after size selection).

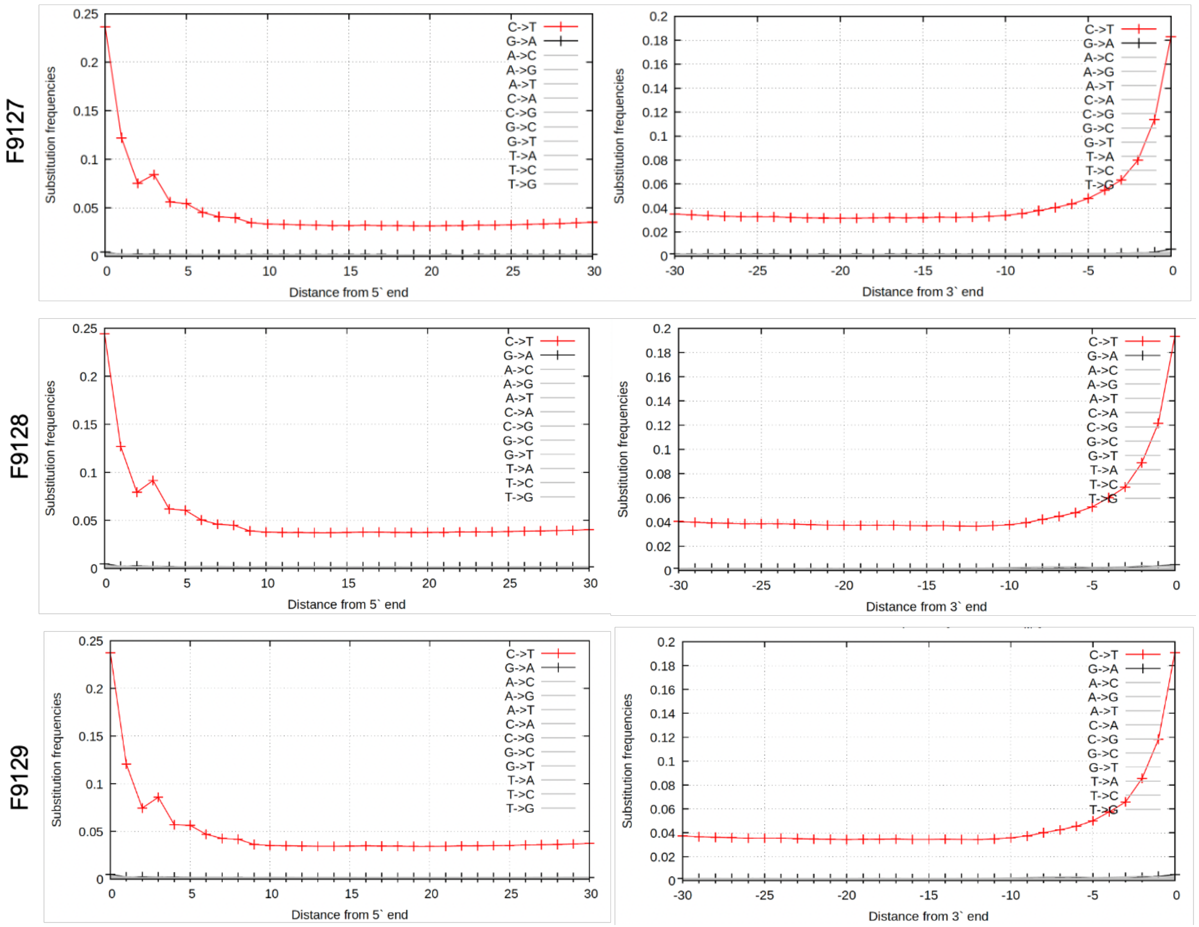

Supplementary Figure 3 Terminal substitution patterns by library, with C-to-T substitutions indicated in red, G-to-A substitutions in black, and all other nucleotide substitutions in grey.

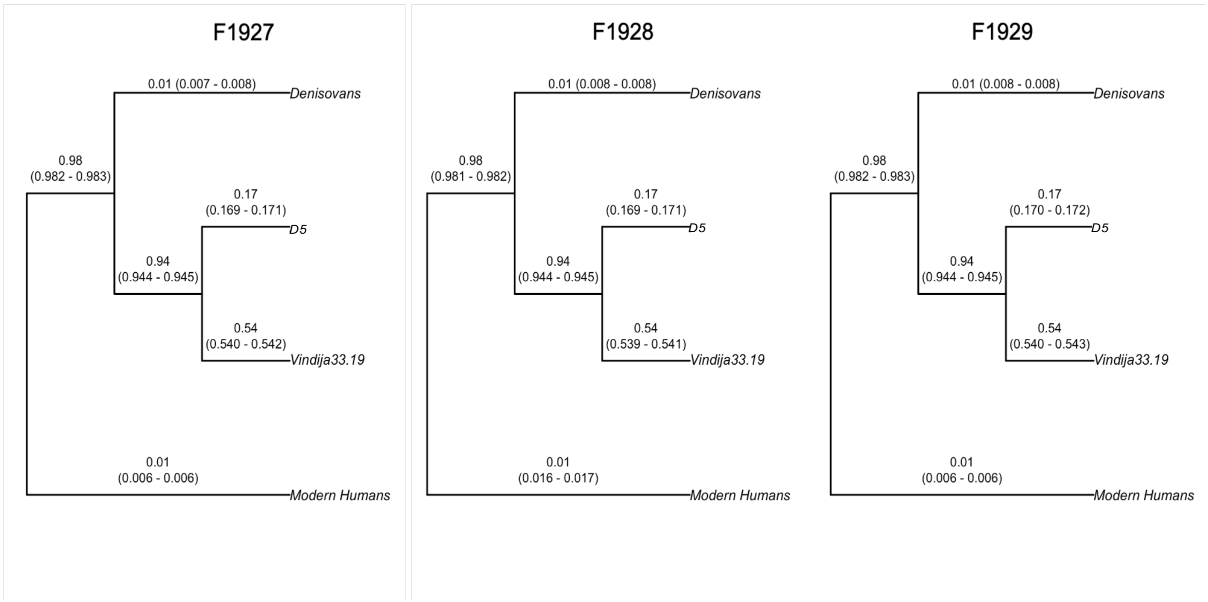

Supplementary Figure 4 Lineage assignment by library. Support (from 0 to 1) is indicated above each branch, with the corresponding exact 95% binomial confidence intervals.

We merged all the data available for Goyet Q56-1 together, combining the deeply sequenced libraries described in this section with the published low-coverage Goyet Q56-1 genome[12], using *samtools* (version 1.3.1-21)[13]. Finally, we used GATK (version 1.3.14)[14] for indel realignment. We estimated the nuclear coverage for each sequencing library by counting the number of bases in the part of sequences that overlap confidently alignable regions of the human genome (map35_100 track) on all autosomes and dividing by the total length of alignable regions. Summing over all data, we estimated a genome-wide coverage of 22.4-fold. Chromosome X shows a similar coverage to that of the autosomes, confirming that Goyet Q56-1 stems from a female individual.

References

1. Gansauge, M.-T., Aximu-Petri, A., Nagel, S. & Meyer, M. Manual and automated preparation of single-stranded DNA libraries for the sequencing of DNA from ancient biological remains and other sources of highly degraded DNA. *Nat. Protoc.* **15**, 2279–2300 (2020).
2. Meyer, M. *et al.* Nuclear DNA sequences from the Middle Pleistocene Sima de los Huesos hominins. *Nature* **531**, 504–507 (2016).
3. Kircher, M., Sawyer, S. & Meyer, M. Double indexing overcomes inaccuracies in multiplex sequencing on the Illumina platform. *Nucleic Acids Res.* **40**, e3 (2012).
4. Renaud, G., Stenzel, U. & Kelso, J. leeHom: adaptor trimming and merging for Illumina sequencing reads. *Nucleic Acids Res.* **42**, e141 (2014).
5. Li, H. & Durbin, R. Fast and accurate long-read alignment with Burrows–Wheeler transform. *Bioinformatics* **26**, 589–595 (2010).
6. Meyer, M. *et al.* A High-Coverage Genome Sequence from an Archaic Denisovan Individual. *Science* **338**, 222–226 (2012).
7. Peyrégne, S. & Peter, B. M. AuthentiCT: a model of ancient DNA damage to estimate the proportion of present-day DNA contamination. *Genome Biol.* **21**, 246 (2020).
8. Green, R. E. *et al.* A Draft Sequence of the Neandertal Genome. *Science* **328**, 710–722 (2010).
9. Bokelmann, L. *et al.* A genetic analysis of the Gibraltar Neanderthals. *Proc. Natl. Acad. Sci.* **116**, 15610–15615 (2019).
10. Prüfer, K. *et al.* The complete genome sequence of a Neanderthal from the Altai Mountains. *Nature* **505**, 43–49 (2014).
11. Mallick, S. *et al.* The Simons Genome Diversity Project: 300 genomes from 142 diverse populations. *Nature* **538**, 201–206 (2016).
12. Hajdinjak, M. *et al.* Reconstructing the genetic history of late Neanderthals. *Nature* **555**, 652–656 (2018).

13. Li, H. *et al.* The Sequence Alignment/Map format and SAMtools. *Bioinformatics* **25**, 2078–2079 (2009).
14. Auwera, G. A. V. de & O'Connor, B. D. *Genomics in the Cloud: Using Docker, GATK, and WDL in Terra*. (O'Reilly, Beijing Boston Farnham Sebastopol Tokyo, 2020).

3. Genotyping the GN1 high-coverage genome

We genotyped the data of Goyet Q56-1 or GN1 using *snpAD* (version 0.3.11)[1], a software that takes into account the effect of ancient DNA damage in generating genotype calls. Input files for *snpAD* were prepared using sequences that had a mapping quality of at least 25 and a length of at least 35 bases, and using bases with a quality of at least 30. Only positions within the 35mer mappability track[2] were considered. Since the GN1 data consist of both Uracil-DNA-Glycosylase (UDG) and Endonuclease VIII treated libraries (library A9122)[3], as well as non-UDG treated libraries (A9229 and A9349 from Hajdinjak et al.[3] and the newly generated libraries[3]F9127, F9128 and F9129[3], we processed these two types of data separately to generate input files for *snpAD*. For the UDG treated library, we set an offset of 31 base pairs to the position-profile in *snpAD*, thus accounting for the deviations from the expected ancient damage patterns expected as a result of the UDG pre-treatment (https://bioinf.eva.mpg.de/snpAD/) and in order to be able to combine input files for *snpAD* parameter estimation. Error rates and genotype frequencies were estimated independently for each chromosome. These parameters were then used to call the most likely genotype at each site with snpAD. For all comparative analyses with other high-coverage Neandertals (including Neandertals – Neandertal D5[2], Chagyrskaya 8[4] and Vindija 33.19[5], we reprocessed their genotypes in the same way.

In order to further reduce the fraction of erroneous calls in high-coverage individuals, we generated a GN1 genome-specific quality filter (i.e., 'Manifesto filter') that excludes sites within the extreme 2.5% of the GC-corrected coverage distribution, simple repeats (excluding regions that overlapped with the USCS tracks for repetitive elements in http://hgdownload.soe.ucsc.edu/goldenPath/hg19/database/simpleRepeat.txt.gz and http://hgdownload.soe.ucsc.edu/goldenPath/hg19/database/rmsk.txt.gz) and indels (identified using GATK version 1.3-14-g348f2db, http://www.broadinstitute.org/gsa/wiki). Similar filters were used for the published archaic genomes (http://ftp.eva.mpg.de/neandertal). We calculated GC content in windows of 51 bases and assigned the value for the middle position for each base in the genome. GC-coverage was calculated for each position using GN1 sequences aligning with high confidence and excluding low-quality bases (MQ $\geq$ 25, base-quality $\geq$ 30, map35_100 regions), and binned according to GC content.

Moreover, only sites that overlap confidently alignable regions of the human genome (35mer mappability filter) were retained for all downstream analyses. Our filter also had a coverage cut-off, requiring a minimum of 10-fold coverage. As a result, after filtering, we retained 1,718,722,531 positions.

Finally, as a quality control of the generated genotypes, we compared the allelic imbalance (i.e., the relative frequency of the alternative alleles at heterozygous positions) to the other high-coverage Neandertals (Supplementary Figure 5). Moreover, following previous studies (see [6], we calculated the transition/transversion ratio (TS/TV) as a quality check for genotype quality. Consistent with the previous studies, we obtained an average TS/TV ratio of ~2, which is expected for both ancient and modern humans (Supplementary Table 11).

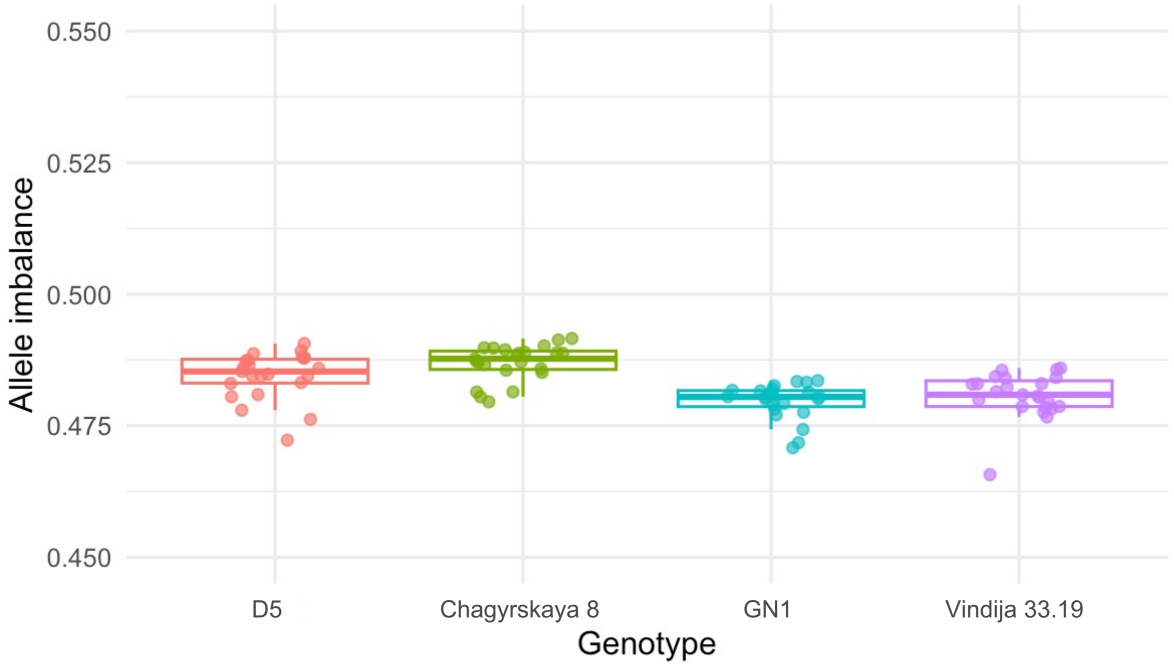

Supplementary Figure 5 Allelic imbalance of the high-coverage Neandertal genotypes. Each data point represents a chromosome.

Supplementary Table 11 Transition to transversion ratios and coverage estimates for each chromosome of GN1 for sequences longer than 35 base pairs.

Chromosome	Number of sites	Mean depth	TS/TV ratio
1	146,582,382	22.98	2.04
2	161,226,751	23.38	1.97
3	133,073,424	23.47	1.94
4	125,696,071	23.90	1.92
5	119,265,922	23.51	1.94
6	113,116,524	23.64	2.0
7	97,463,348	23.40	1.97
8	97,336,069	23.29	1.84
9	73,788,544	23.04	1.91
10	86,205,263	22.99	2.03
11	87,118,042	22.80	1.97
12	85,812,843	23.22	2.0
13	66,180,348	23.85	1.99
14	58,636,474	23.18	1.99
15	51,327,485	22.98	1.98
16	48,178,264	22.16	1.8
17	47,166,730	22.06	2.15
18	52,564,454	23.40	2.02
19	28,481,655	21.02	2.1
20	41,350,660	22.25	2.13
21	22,799,625	23.24	2.01
22	21,155,597	21.24	2.2

References

1. Prüfer, K. snpAD: an ancient DNA genotype caller. *Bioinformatics* **34**, 4165–4171 (2018).
2. Prüfer, K. *et al.* The complete genome sequence of a Neanderthal from the Altai Mountains. *Nature* **505**, 43–49 (2014).
3. Hajdinjak, M. *et al.* Reconstructing the genetic history of late Neanderthals. *Nature* **555**, 652–656 (2018).
4. Mafessoni, F. *et al.* A high-coverage Neandertal genome from Chagyrskaya Cave. *Proc. Natl. Acad. Sci.* **117**, 15132–15136 (2020).
5. Prüfer, K. *et al.* A high-coverage Neandertal genome from Vindija Cave in Croatia. *Science* **358**, 655–658 (2017).
6. Sümer, A. P. *et al.* Earliest modern human genomes constrain timing of Neanderthal admixture. *Nature* **638**, 711–717 (2025).

4. Private variants of GN1

We have identified single nucleotide substitutions in the lineages leading to different groups of Neandertals. For this, we relied on the genotype calls from the following archaic human genomes: Neandertals Vindija 33.19[1], D5[2], Chagyrskaya 8[3] and GN1, as well as the Denisovan D3[4], all filtered as described in Section 3. Moreover, we kept only bi-allelic sites where the global allele frequency of the derived allele was lower than 0.5% among the modern individuals from the gnomAD genomes (version 3.1.2) or gnomAD exomes (version 4.0) datasets when available[5]. We also required that in each genetic ancestry group, as classified by gnomAD, the derived allele frequency among present-day humans is lower than 5%. If no corresponding entry could be found in the gnomAD data, we assumed that all individuals carried the reference allele.

We lifted over the GRCh38-mapped gnomAD genomes and exomes datasets to GRCh37 coordinates using picard LiftoverVcf (version 2.18.29, https://gatk.broadinstitute.org/hc/en-us/articles/360037060932-LiftoverVcf-Picard). We determined the ancestral state by requiring the chimpanzee (panTro4, GCA_000001515.4) allele as well as at least one of the gorilla (gorGor3, GCA_000151905.1) and orangutan (ponAbe2, GCA_000001545.3) alleles to be present and all available alleles to match.

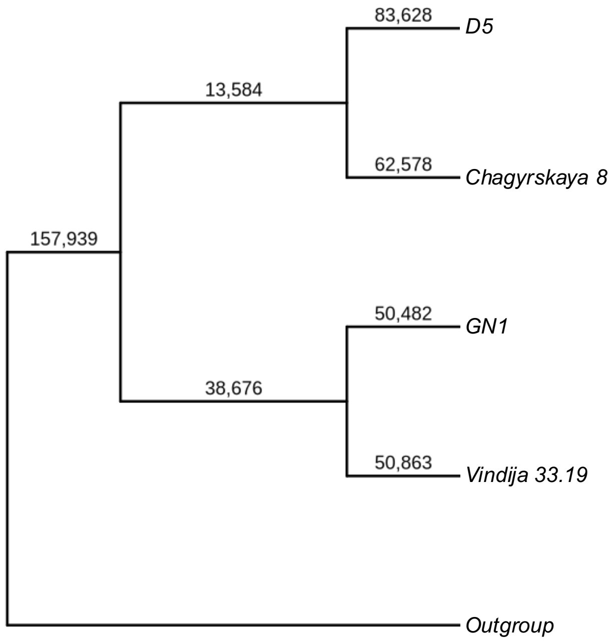

Supplementary Figure 6 Numbers of positions where the individual high-coverage Neandertal genomes on the respective branches each carry at least one derived allele.

We were able to find 157,939 derived variants shared by all available high-coverage Neandertal genomes. On the branch leading to early eastern Neandertals (D5 and Chagyrskaya 8), we identified

13,585 such variants. In contrast, in the lineage leading to late western Neandertals, represented by Vindija 33.19 and GN1, we found 38,676 shared derived sites. Finally, we identified 50,482 GN1-specific private mutations (Supplementary Figure 6), which we functionally analysed (Supplementary Figure 7).

We annotated the derived mutations private to GN1 via the Ensembl Variant Effect Prediction (VEP) pipeline (version 112, https://www.ensembl.org/info/docs/tools/vep/index.html). Of the variants overlapping coding sequences (CDS of protein-coding genes according to Gencode, Release 19), 268 variants were classified as synonymous changes ("synonymous_variant") while 392 were predicted to be non-synonymous, protein-changing variants ("missense_variant" or more severe; Supplementary Figure 7 and 8, Supplementary Table 12). After normalizing for the number of sites in the genome, all Neandertals have significantly fewer non-synonymous than synonymous sites (Fisher's exact test, $p = 0.0006$). While the late Neandertals have slightly more non-synonymous sites, a Chi-square test reveals that the differences in pN/pS ratios between Neandertals are not significant ($\chi^2 = 4.0878$, $df = 3$, $p = 0.25$). And comparing late Neandertals (GN1 and Vindija 33.19) with the earlier Neandertals from the Altai also results in non-significance difference of ratios (Fisher's exact test, $p = 0.0959$).

Supplementary Table 12 Ratios of non-synonymous / synonymous changes in high-coverage Neandertals. The numbers of occurrences are normalized by a modern human variation baseline obtained from bi-allelic SNVs in the VEP-annotated gnomAD v2 database. p-value: Fisher's exact test on synonymous, non-synonymous variable sites vs total sites in the genome. Syn – synonymous

Individual	Number of syn. changes	Number of syn. changes, normalized by 494,722 occurrences in gnomAD	Number of non-syn. changes	Number of non-syn. changes, normalized by 955,266 occurrences in gnomAD	pN/pS Ratio	normalized pN/pS Ratio	p-value
D5	421	0.000851	579	0.000606	1.38	0.71	2E-7
Chagyrskaya 8	376	0.000760	466	0.000488	1.24	0.64	3E-10
GN1	268	0.000542	392	0.000410	1.46	0.76	0.0006
Vindija 33.19	281	0.000568	421	0.000441	1.50	0.78	0.001

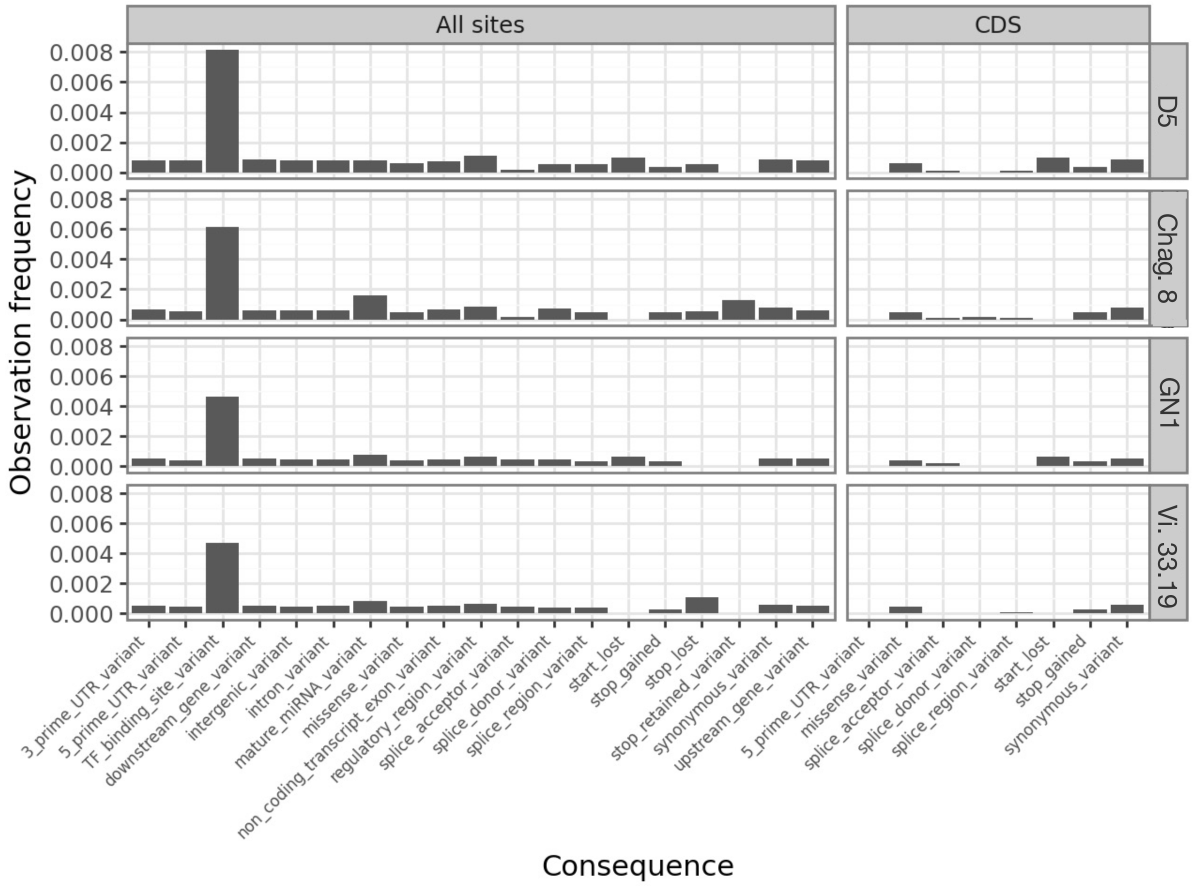

Supplementary Figure 7 Neandertal-specific variants classified by Ensembl's VEP. In the left panel we show all variants; in the right one only those that fall into coding sequences. The numbers of occurrences are normalized by a modern human variation baseline obtained from bi-allelic SNVs in the VEP-annotated gnomAD v2 database.

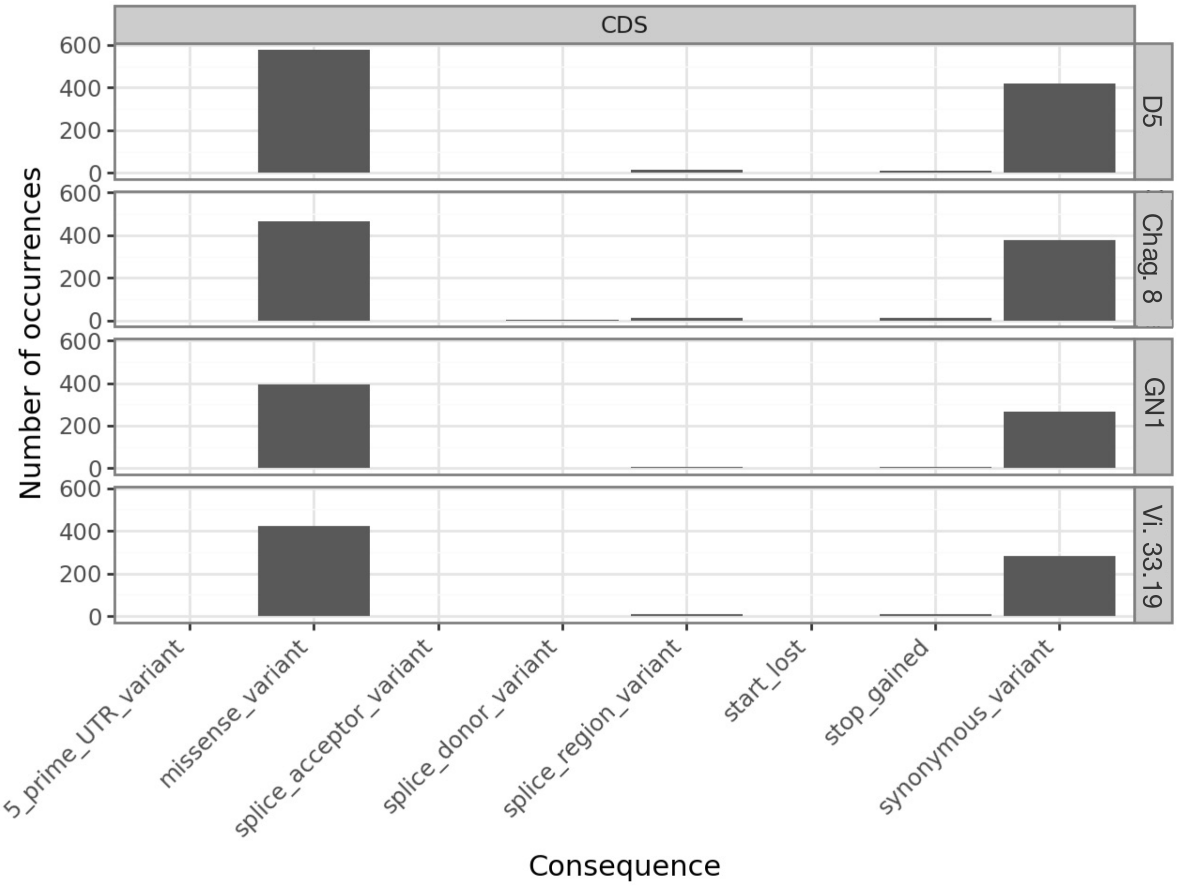

Supplementary Figure 8 Numbers of occurrences of Neandertal-specific variants affecting coding sequences as classified by Ensembl's VEP.

To evaluate the functional impact of protein-changing substitutions, we used PolyPhen2 (version 2.2.3, release 405c[6]), which predicts the effects of amino acid substitutions on protein structure and function based on sequence homology, 3D protein structures, and other data sources. The private and shared variants were annotated via Ensembl VEP with PolyPhen2 scores representing the predicted probability that a substitution is deleterious. Values closer to 1 signify more confidence in the deleteriousness of a variant. Scores < 0.45 are classified as "Benign", between 0.45 and 0.9 as "Possibly Damaging", and scores > 0.9 as "Probably Damaging". The difference between early and late Neandertals variants classified as "Possibly Damaging" and "Probably Damaging" (scores > 0.45) was not significant (Wilcoxon rank sum test, W=116.5, p=0.08; Supplementary Figure 9).

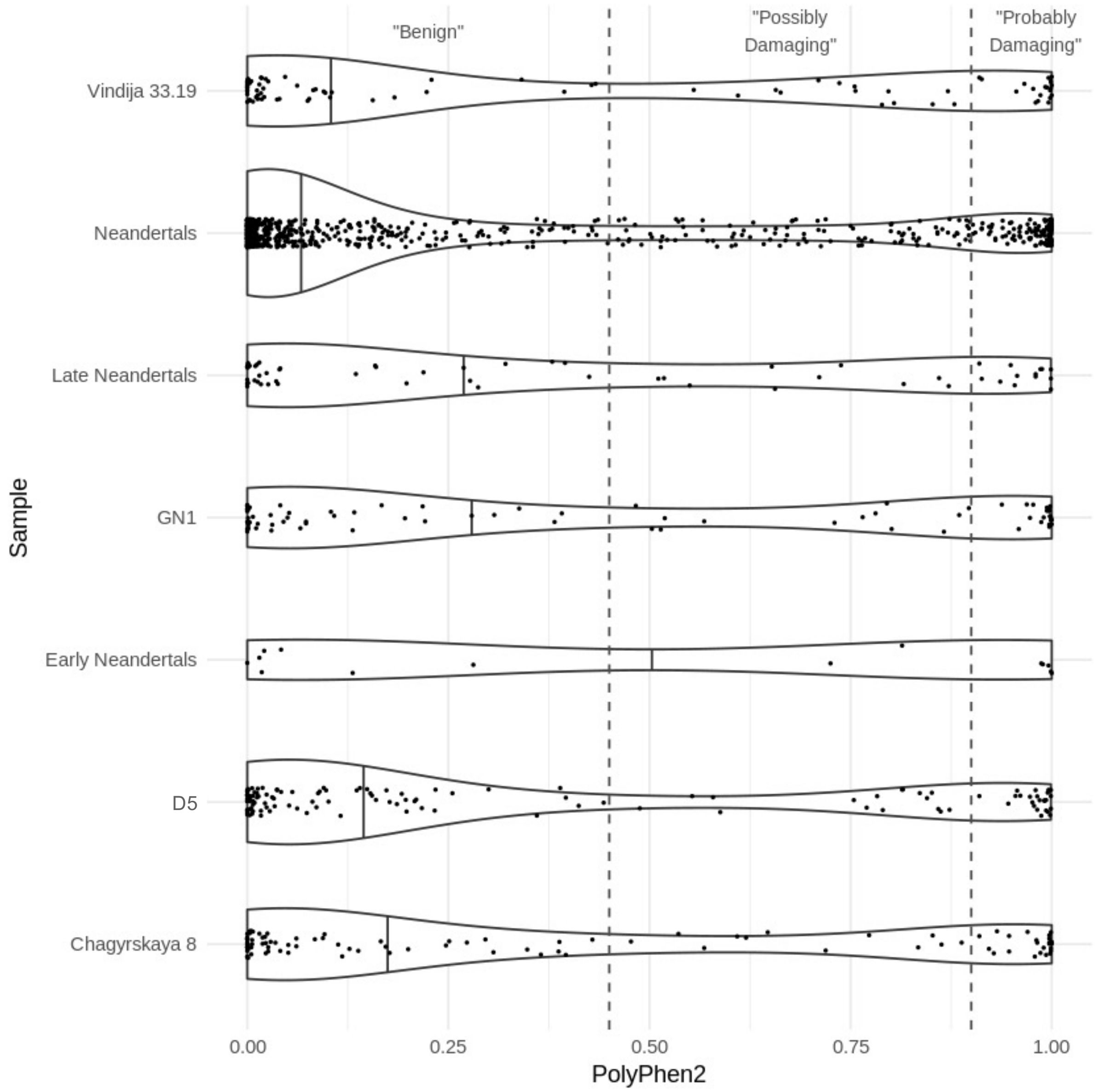

Supplementary Figure 9 PolyPhen2 scores of private and shared variants. Sites correspond to those in Supplementary Figure 6. Neandertals: Sites shared among all Neandertals. Early Neandertals: Sites shared between Denisova 5 and Chagyrskaya 8. Late Neandertals: Sites shared between Vindija 33.19 and Goyet GN1. The dashed lines show the boundaries between scores classified as "benign" (< 0.45), "possibly damaging" (< 0.9), and "probably damaging" (> 0.9).

To evaluate the functional impact of non-coding variants, we use phyloP[7]. PhyloP-scores using the 46-way placental mammal alignment were downloaded from the UCSC genome-browser (https://hgdownload.soe.ucsc.edu/goldenPath/hg19/phyloP46way/) . We then annotated the private and shared variants (see Supplementary Figure 10) with base-wise PhyloP-scores, which range from -8 (most accelerated) to +3 (most conserved). Overall, the distribution of PhyloP-scores are very similar

between all private and shared variants, and the difference between Early and Late Neandertals is not significant (t-test, t=0.42, p=0.65).

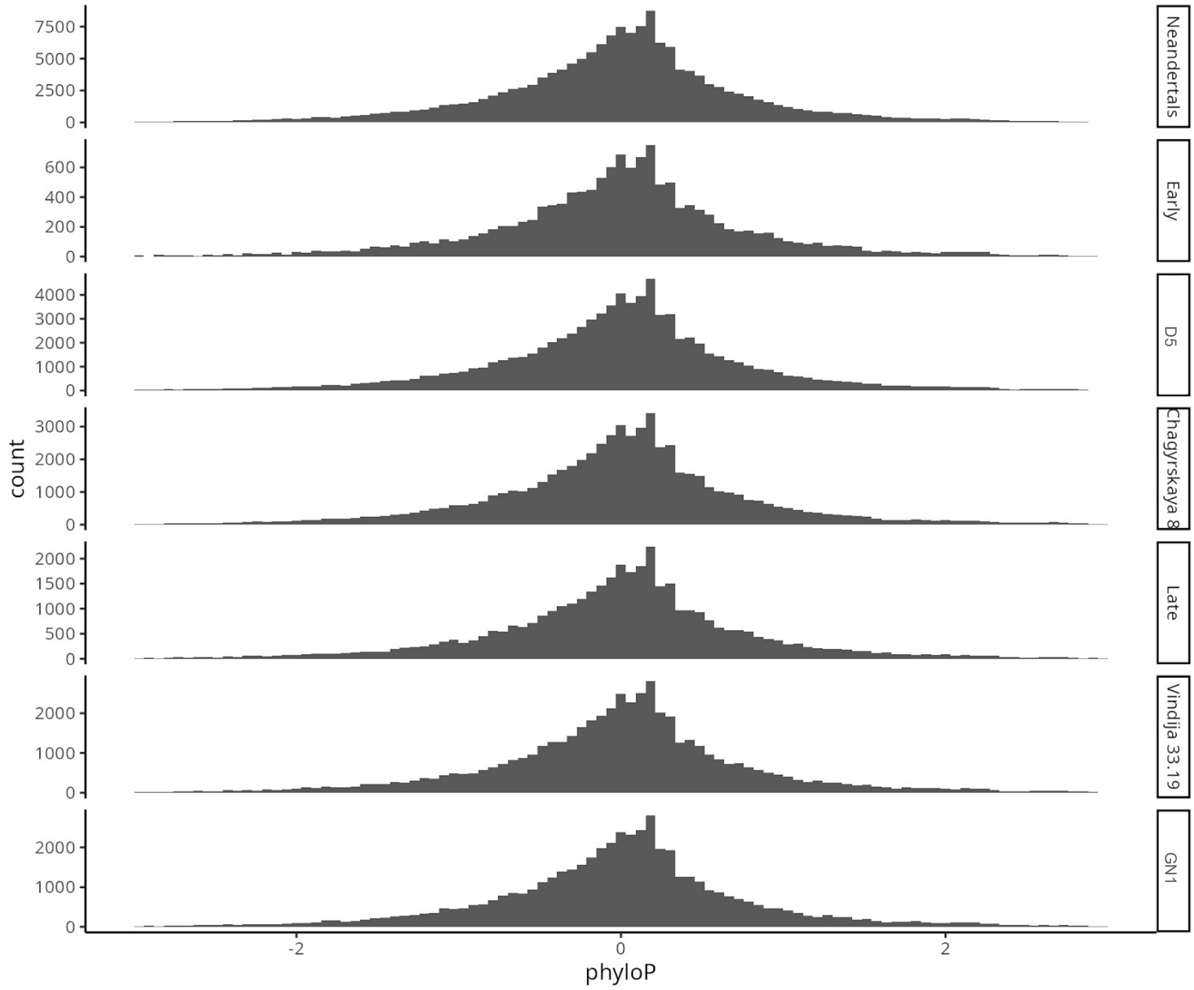

Supplementary Figure 10 PhyloP of Neandertal-private variants. Sites correspond to those in Supplementary Figure 6. Neandertals: Sites shared between Neandertals. Early: Sites shared between Neandertal D5 and Chagyrskaya 8. Late: Sites shared between Vindija 33.19 and GN1.

4.1 Protein changes: GO, HPO, ABA

For the following analyses, we used the R package GOfuncR (version 1.23.2, https://github.com/sgrote/GOfuncR) to perform over-representation analyses of nodes in the Gene Ontology (GO) and the Human Phenotype Ontology (HPO) in the set of genes affected by these protein-changing private mutations[6]. We also used the ABAEnrichment R package[7] for enrichment analyses of brain regions from the Allen Brain Atlas (http://www.brain-map.org). It contained three expression data sets: 1) adult individuals, 2) five different developmental stages (prenatal, infant, child, adolescent, adult), and 3) developmental effect scores measuring the age effect on expression per gene.

Both software packages use the ontology enrichment evaluation software FUNC[8]. We used the hypergeometric test feature of these packages to evaluate the enrichment of candidate genes compared to background genes for each ontology node or brain region. In the case of putative protein-altering mutations, we used all protein-coding genes from the *OrganismDb* R-package "Homo.sapiens" as a background set[9].

We first focused on the set of genes which had been annotated as affected by protein-changing Goyet-specific mutations ("missense variant" or more severe) for further analyses. Additionally, we required that the affected transcript had been verified by the Consensus CDS (CCDS) project[10]. With these criteria, we found 404 putative private mutations affecting 346 genes in GN1. GOfuncR as well as ABAEnrichment assign family-wise error rates (FWER) using random sets which are generated by repeated permutation of the candidate and background gene sets, scaling the error rates by the length of the gene. We neither found any significantly (FWER < 0.05) overrepresented category in the Gene Ontology, nor the phenotypes in the Human Phenotype Ontology nor the Allen Brain Atlas data.

4.2 Regulatory changes

Genomic regions with evidence for regulatory function are listed as "regulatory features" in the Ensembl data. However, the VEP annotations do not contain any mappings to genes that are influenced by the regulatory elements. Therefore, we mapped Ensembl regulatory features to genes if they overlapped with the transcription start sites of a gene to be able to perform enrichment analyses on the resulting list. This resulted in 10,008 putative private mutations overlapping 1,794 regulatory features, which were assigned to 2,074 different genes. We restricted the background gene set for the ontology enrichment test by only including genes which we were able to assign regulatory features to. GOfuncR as well as ABAEnrichment assign family-wise error rates (FWER) using random sets which are generated by repeated permutation of the candidate and background gene sets, scaling the error rates by the length of the "regulatory feature".

We did find five significantly overrepresented GO categories (Supplementary Table 13), but no enriched HPO category. Interestingly, among others, we see an overrepresentation of the GO categories "anatomical structure formation involved in morphogenesis" and "anatomical structure morphogenesis" within the list of genes putatively affected by regulatory changes private to GN1. This coincides with a visible increase in the gracility of later Neandertals[11].

Supplementary Table 13 List of the ten most overrepresented GO categories within the list of genes whose transcription start sites (TSS) overlap regulatory features that are affected by private variants in Goyet Q56-1, sorted by FWER.

**Ontology**	**Node ID**	**Node Name**	**FWER**
biological_process	GO:0030334	regulation of cell migration	0.015
biological_process	GO:0072359	circulatory system development	0.032
biological_process	GO:0048646	anatomical structure formation involved in morphogenesis	0.032
biological_process	GO:0009653	anatomical structure morphogenesis	0.039
biological_process	GO:0040012	regulation of locomotion	0.044
biological_process	GO:0051270	regulation of cellular component movement	0.050
biological_process	GO:2000145	regulation of cell motility	0.066
biological_process	GO:0016477	cell migration	0.093
molecular_function	GO:0005178	integrin binding	0.096
biological_process	GO:0032502	developmental process	0.125

4.3 EMBL GWAS catalog overlaps

We then listed overlaps of private, GN1-specific mutations with the Genome Wide Association Studies (GWAS) catalog (EMBL-EBI GWAS catalog, accessed on 28. October 2024, downloaded and lifted over to GRCh37 via the "gwascat" R package, version 2.38.0)[12]. We found two private variants that may impact human traits as identified by genome-wide association studies. These mutations might affect the traits “carotid artery thickness”, and “response to antipsychotic drug, high density lipoprotein cholesterol measurement” (Supplementary Table 14).

Supplementary Table 14 List of direct overlaps of private, GN1-specific mutations with the EMBL-EBI GWAS catalog.

Variant	Initial Sample Size	Mapped Gene	P-Value	GWAS Catalog Accession	Mapped Trait
7:144288768 rs145770279	3,963 Sub-Saharan African ancestry females	TPK1	6.00E-06	GCST90092503	carotid artery thickness
12:55425553 rs117513619	1,936 Han Chinese ancestry individuals	NEUROD4 - OR9K1P	1.00E-06	GCST90454531	response to antipsychotic drug, high density lipoprotein cholesterol measurement

References

1. Prüfer, K. *et al.* A high-coverage Neandertal genome from Vindija Cave in Croatia. *Science* **358**, 655–658 (2017).
2. Prüfer, K. *et al.* The complete genome sequence of a Neanderthal from the Altai Mountains. *Nature* **505**, 43–49 (2014).
3. Mafessoni, F. *et al.* A high-coverage Neandertal genome from Chagyrskaya Cave. *Proc. Natl. Acad. Sci.* **117**, 15132–15136 (2020).
4. Meyer, M. *et al.* A High-Coverage Genome Sequence from an Archaic Denisovan Individual. *Science* **338**, 222–226 (2012).
5. Chen, S. *et al.* A genomic mutational constraint map using variation in 76,156 human genomes. *Nature* **625**, 92–100 (2024).
6. Adzhubei, I. A. *et al.* A method and server for predicting damaging missense mutations. *Nat. Methods* **7**, 248–249 (2010).
7. Pollard, K. S., Hubisz, M. J., Rosenbloom, K. R. & Siepel, A. Detection of nonneutral substitution rates on mammalian phylogenies. *Genome Res.* **20**, 110–121 (2010).

8. Gargano, M. A. *et al.* The Human Phenotype Ontology in 2024: phenotypes around the world. *Nucleic Acids Res.* **52**, D1333–D1346 (2024).
9. Grote, S., Prüfer, K., Kelso, J. & Dannemann, M. ABAEnrichment: an R package to test for gene set expression enrichment in the adult and developing human brain. *Bioinformatics* **32**, 3201–3203 (2016).
10. Prüfer, K. *et al.* FUNC: a package for detecting significant associations between gene sets and ontological annotations. *BMC Bioinformatics* **8**, 41 (2007).
11. Marc Carlson, H. P. OrganismDbi. Bioconductor https://doi.org/10.18129/B9.BIOC.ORGANISMDBI (2017).
12. Pruitt, K. D. *et al.* The consensus coding sequence (CCDS) project: Identifying a common protein-coding gene set for the human and mouse genomes. *Genome Res.* **19**, 1316–1323 (2009).
13. Cosnefroy, Q. *et al.* Highly selective cannibalism in the Late Pleistocene of Northern Europe reveals Neandertals were targeted prey. *Sci. Rep.* **15**, 40741 (2025).
14. Carey, V. gwascat: representing and modeling data in the EMBL-EBI GWAS catalog. (2024).

5. Branch shortening

We calculated the genetic age of the GN1 through molecular "branch-shortening"[1]. Over time, mutations are gradually expected to accumulate, and thus indirectly measure time. Hence, for two contemporaneous individuals, we expect to observe a comparable number of derived variants on each of their branches. In contrast, an older genome has less time to accumulate mutations, and thus will have fewer derived variants specific to their lineage. Thus, we estimated a genetic age of an individual by scaling said difference assuming a known divergence times between two genomes.

We polarised the mutations in the hominin tree using the genotypes of four apes: a chimpanzee (panTro4, GCA_000001515.4), a bonobo (panPan1.1, AJFE00000000.2), a gorilla (gorGor3, GCA_000001515.4) and an orangutan (ponAbe2, GCA_000001545.3). For calibration, we focused on the chimpanzee as the outgroup to all hominins, assuming a divergence of 13 million years ago with a mutation rate of 0.5×10^{-9} per bp per year[1] We then counted the number of derived variants on each of the hominin branches of interest. Our point of reference for the present-day modern human branch was defined by an Mbuti individual (HGDP00982)[2]. As for the archaic humans, besides GN1, we also contextualised our results by calculating in parallel the ages of three other high-coverage Neandertals (Vindija 33.19[3], D5[1], Chagyrskaya 8[4], one Denisovan – Denisova 3[2]), and five ancient modern humans (Ust'-Ishim[5], Stuttgart or "LBK", Loschbour[6], Zlatý kůň and Ranis 13[7]). All archaic hominins and ancient modern humans were genotyped and filtered as described in Section 3, and then randomly sampled at each heterozygous position. To further mitigate the effect of ancient DNA damage, we restricted our branch shortening analyses to transversions.

We estimated the molecular ages of all archaic and ancient individuals by taking the difference between the number of derived sites on each archaic branch in respect to the Mbuti and scaling it by divergence time.

We estimated the weighted average of the genetic age estimates of GN1to be around 46,032 years BP (IQR 34,129 – 63,721 years BP as defined by the first and third quantiles), which overlaps with the estimates for the Vindija 33.19 Neandertal at around 47,056 years BP (IQR= 35,184 – 68,154 years BP, Supplementary Figure 11). While the branch shortening estimates can be quite noisy (as noted by Sümer et al.[7], both of the interquartile ranges overlap with the direct radiocarbon dates of these specimens, i.e., 42,450 years calBP (95.4% CI between 42,740 and 42,160 years calBP, IntCal20) for GN1[8] and 47,310 years calBP (95.4% CI between 49,930 and 44,690 years calBP, IntCal20) for Vindija 33.19[9]. Thus, we conclude that the Vindija 33.19 and GN1 Neandertals were most likely contemporaneous.

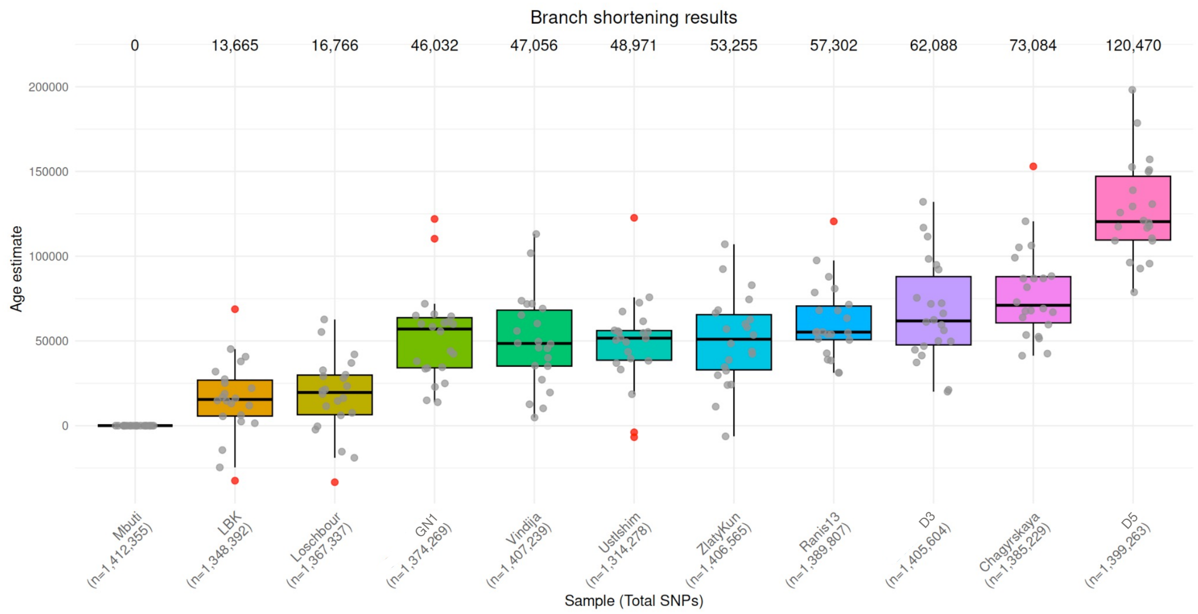

Supplementary Figure 11 Age estimates by branch shortening by chromosome in each of the selected high-coverage archaic hominins and ancient humans, taking a present-day Mbuti as a point of reference. On the X-axis, the total number of SNPs used appears at the bottom of each individual, and the weighted mean age average at the top. Outliers are represented as red dots and were excluded from the calculation of the average age. The lower and upper hinges of the boxplots represent the 25th and 75th percentiles, respectively, and the whiskers extend up to 1.5 times the interquartile range from the hinges.

References

1. Prüfer, K. *et al.* The complete genome sequence of a Neanderthal from the Altai Mountains. *Nature* **505**, 43–49 (2014).
2. Meyer, M. *et al.* A High-Coverage Genome Sequence from an Archaic Denisovan Individual. *Science* **338**, 222–226 (2012).
3. Prüfer, K. *et al.* A high-coverage Neandertal genome from Vindija Cave in Croatia. *Science* **358**, 655–658 (2017).
4. Mafessoni, F. *et al.* A high-coverage Neandertal genome from Chagyrskaya Cave. *Proc. Natl. Acad. Sci.* **117**, 15132–15136 (2020).
5. Fu, Q. *et al.* Genome sequence of a 45,000-year-old modern human from western Siberia. *Nature* **514**, 445–449 (2014).
6. Lazaridis, I. *et al.* Ancient human genomes suggest three ancestral populations for present-day Europeans. *Nature* **513**, 409–413 (2014).
7. Sümer, A. P. *et al.* Earliest modern human genomes constrain timing of Neanderthal admixture. *Nature* **638**, 711–717 (2025).
8. Rougier, H. *et al.* Neandertal cannibalism and Neandertal bones used as tools in Northern Europe. *Sci. Rep.* **6**, 29005 (2016).

9. Devièse, T. *et al.* Direct dating of Neanderthal remains from the site of Vindija Cave and implications for the Middle to Upper Paleolithic transition. *Proc. Natl. Acad. Sci.* **114**, 10606–10611 (2017).

6. Demographic history

Having a high-coverage genome of the GN1 enabled us to study her population demography, taking advantage of the Pairwise Sequentially Markovian Coalescent method (PSMC)[1]. Specifically, we employed PSMC version 0.6.5 to estimate how population size changed over time, as well as to estimate the sample's age, following the procedure described in Sümer et al.,[2], using the pipeline available at https://github.com/StephanePeyregne/calibratePSMC. As reference, we co-analysed a set of previously published high-coverage individuals: three Neandertals including Vindija 33.19[3], D5[4], and Chagyrskaya 8[5], and the Denisovan 3 (D3)[6].

6.1 Inference of population size over time

We generated the input files for running PSMC in a suitable "psmcfa" format. For this, we split each genome in windows of 100 base pairs. For each window we then assigned a "state" in function of heterozygosity: "state K" if it included at least one heterozygous site, "state T" if all sites called were homozygous, or "state N" if the amount of missing data was higher than 10%. We generated the states of heterozygosity in the high-coverage genomes using an in-house script (myvcf2fq.sh, also available at https://github.com/StephanePeyregne/calibratePSMC). We parallelised it by chromosome, excluding the sex chromosomes, and then concatenated all the files from the same individual.

After this, we used PSMC to get estimates of the demographic history of each genome, assuming a mutation rate of 1.45e-8 per year and a generation time of 29 years. We used the default 25 iterations, with specified time-steps of "4+25*2+4+6", which has been shown to be a good compromise between the resolution of the results at the cost of higher computational effort[5]. Importantly, PSMC is susceptible to biases created by missing data[3,5]. Considering that all the archaic genomes of interest had been extensively filtered using their sets of 'Manifesto' filters (see Section 3), it was not possible to directly rely on the unfiltered demographics (Supplementary Figure 12).

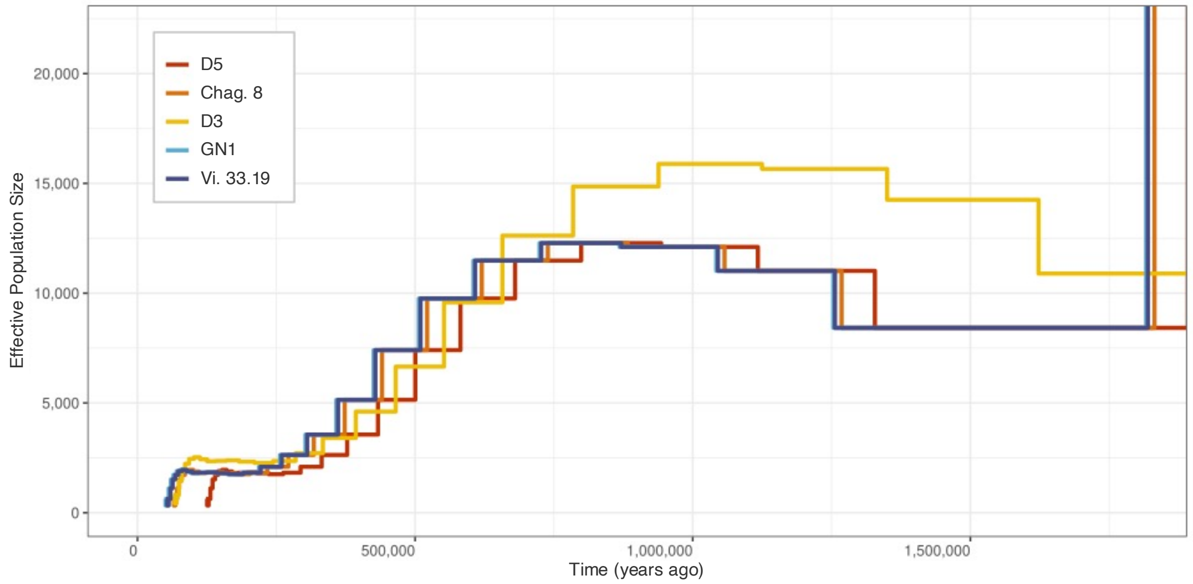

Supplementary Figure 12 Demography curves uncorrected for missing data, obtained by PSMC and assuming ages of 120,000 years for the D5l, 60,000 years for the Chagyrskaya 8 Neandertal and the Denisova 3 Denisovan, and 45,000 years for the Vindija 33.19 and GN1 Neandertals. Curves of Vindija 33.19 and GN1 plot on top of each other.

To correct for the missing data, we used *scrm* simulations to explore the effect of varying parameter combinations. We studied the correction factors for the parameters representing the recombination rate "r" and the mutational rate "q", both ranging from 0 to 2, in steps of 0.05. Each simulation was filtered in a way that mimicked that of each high-coverage genome, and used as input for PSMC. By using the demographic histories obtained from each simulation, in combination with the real data, we ran PSMC and got a likelihood for each pair of correction factors. We then selected the combination of correction factors with the highest likelihood, i.e., the one that fit better the demographic history of the read data (Supplementary Table 15, Supplementary Figure 13).

The obtained corrected demographies were in line with our expectations (Supplementary Figure 14): as a Late Neandertal, the population history of the GN1 is very similar to that of Vindija 33.19. This is consistent with the branch shortening estimates calculated in Section 5.

Supplementary Table 15 Correction factors of the θ and ρ parameters for the genomes included in this analysis.

**Specimen**	**f_Theta**	**f_Rho**	**Likelihood**
GN1	1.40	1.75	-1,153,632
Chagyrskaya 8	1.40	1.55	-1,052,341
D5	1.45	1.75	-1,155,454
Vindija 33.19	1.40	1.65	-1,255,969
D3	1.40	1.65	-1,416,704

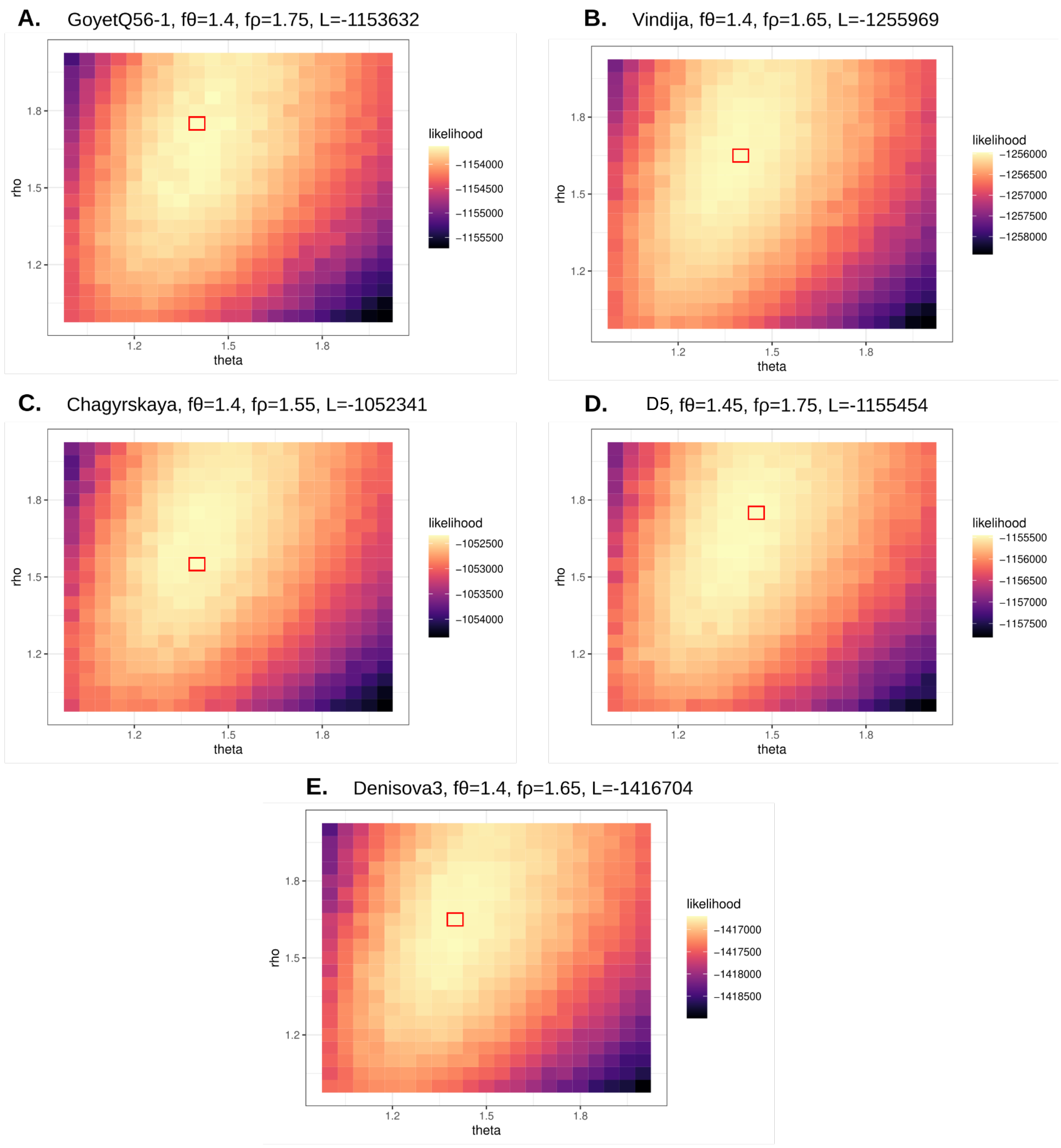

Supplementary Figure 13 Heatmaps representing the fit of the corrected demographic histories. The best fit with the used correction factors, which are also reported in Supplementary Table 16, is indicated with the red square for each high-coverage archaic genome.

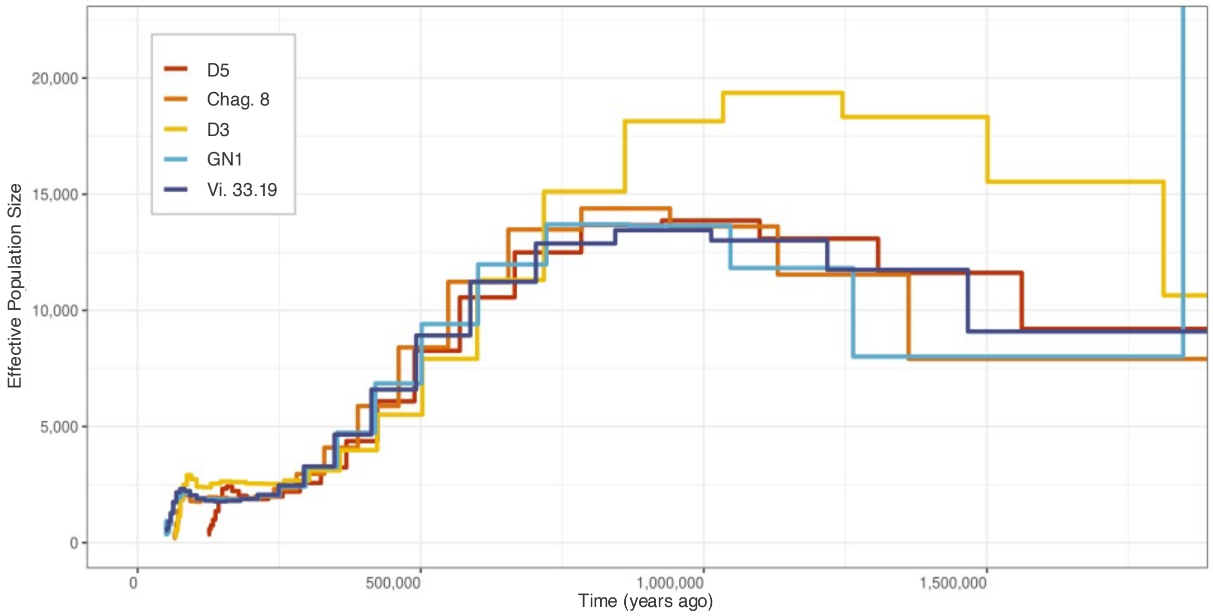

Supplementary Figure 14 Corrected demography curves obtained by PSMC assuming 120,000 years of age for the D5, 60,000 years for the Chagyrskaya 8 Neandertal and the Denisova 3 Denisovan, and 45,000 years for the Vindija 33.19 and GN1 Neandertals.

6.2 Age estimates using demographic histories

To obtain the age differences between the high-coverage Neandertals, complementary to the branch shortening estimates presented in Section 5, we followed the SI Appendix 6 from Mafessoni et al.[5]. We used the demographic histories inferred by PSMC in the previous section, and compared two genomes each time to estimate the difference in age between the two. We used only the high-coverage genomes of Neandertals in this analysis, as the demographic history of the Denisovan individual is too different from the Neandertal demographic histories to compare in this method. Following previous naming conventions[5], we called the older genome in the pairwise comparisons G*old*, and the younger genome as G. Using the *scrm* codes of the corrected demographies (Supplementary Table 17), we generated ten whole genome simulations for each of the four high-coverage Neandertal genomes.

Supplementary Table 16 Corrected scrm codes to produce simulations of the demographic histories plotted in Supplementary Figure 14.

**Genome**	**Simulation code**
GN1	scrm 2 1 -SC abs -p 10 -t 143626 -r 34739.6 2881033286 -en 0.0446 1 1.0000 -en 0.0741 1 0.5210 -en 0.1099 1 0.9818 -en 0.1531 1 1.7508 -en 0.2054 1 2.3841 -en 0.2687 1 2.7142 -en 0.3453 1 2.9833 -en 0.4379 1 3.0722 -en 0.5500 1 2.9591 -en 0.6856 1 2.8527 -en 0.8496 1 2.8713 -en 1.0481 1 2.8964 -en 1.2883 1 2.8314 -en 1.5788 1 2.7589 -en 1.9303 1 2.8657 -en 2.3556 1 3.2948 -en 2.8701 1 4.1386 -en 3.4926 1 5.6013 -en 4.2457 1 8.0906 -en 5.1569 1 11.6484 -en 6.2593 1 15.3469 -en 7.5930 1 18.0610 -en 9.2066 1 19.3159 -en 11.1589 1 19.0374 -en 13.5208 1 17.3276 -en 16.3785 1 13.2431 -en 24.0186 1 45.4743
Chagyrskaya 8	scrm 2 1 -SC abs -p 10 -t 833051 -r 173462 2881033286 -en 0.0068 1 0.0412 -en 0.0113 1 0.1138 -en 0.0168 1 0.2079 -en 0.0235 1 0.3076 -en 0.0316 1 0.4253 -en 0.0415 1 0.5332 -en 0.0535 1 0.5381 -en 0.0682 1 0.4930 -en 0.0860 1 0.4819 -en 0.1076 1 0.4937 -en 0.1339 1 0.4900 -en 0.1659 1 0.4774 -en 0.2048 1 0.4782 -en 0.2521 1 0.4943 -en 0.3096 1 0.5230 -en 0.3796 1 0.5778 -en 0.4647 1 0.6921 -en 0.5681 1 0.9413 -en 0.6939 1 1.4409 -en 0.8469 1 2.2048 -en 1.0330 1 2.9482 -en 1.2592 1 3.3335 -en 1.5343 1 3.3950 -en 1.8689 1 3.3206 -en 2.2757 1 3.0814 -en 2.7705 1 2.1683 -en 4.1038 1 8.3474
Vindija 33.19	scrm 2 1 -SC abs -p 10 -t 1.78171e+07 -r 3.96304e+06 2881033286 -en 0.0004 1 0.0058 -en 0.0007 1 0.0078 -en 0.0010 1 0.0127 -en 0.0014 1 0.0174 -en 0.0019 1 0.0227 -en 0.0024 1 0.0257 -en 0.0031 1 0.0259 -en 0.0039 1 0.0246 -en 0.0049 1 0.0236 -en 0.0062 1 0.0234 -en 0.0076 1 0.0226 -en 0.0094 1 0.0214 -en 0.0115 1 0.0213 -en 0.0141 1 0.0227 -en 0.0172 1 0.0256 -en 0.0210 1 0.0301 -en 0.0255 1 0.0379 -en 0.0310 1 0.0533 -en 0.0377 1 0.0795 -en 0.0457 1 0.1118 -en 0.0554 1 0.1382 -en 0.0671 1 0.1529 -en 0.0812 1 0.1585 -en 0.0983 1 0.1561 -en 0.1189 1 0.1426 -en 0.1438 1 0.1097 -en 0.2102 1 0.4048
D5	scrm 2 1 -SC abs -p 10 -t 108678 -r 25321.5 2881033286 -en 0.0631 1 0.7872 -en 0.1050 1 1.0666 -en 0.1558 1 1.5586 -en 0.2174 1 2.3170 -en 0.2920 1 3.1639 -en 0.3825 1 3.9307 -en 0.4922 1 4.4744 -en 0.6252 1 4.5890 -en 0.7864 1 4.3349 -en 0.9818 1 4.0577 -en 1.2188 1 3.8987 -en 1.5059 1 3.8966 -en 1.8541 1 4.0490 -en 2.2761 1 4.3184 -en 2.7877 1 4.7591 -en 3.4079 1 5.7627 -en 4.1597 1 8.0772 -en 5.0710 1 12.2885 -en 6.1758 1 17.7456 -en 7.5151 1 22.3110 -en 9.1386 1 24.7240 -en 11.1067 1 25.6403 -en 13.4924 1 25.7977 -en 16.3846 1 25.2012 -en 19.8906 1 23.5697 -en 24.1406 1 19.3198 -en 35.5383 1 65.3383

We filtered each of these genomes (G with the filters produced for G*old*) and ran PSMC on each simulated and filtered genome. We then used the command "psmc -N1 -i {input.demography} -o {output} {input.data}" to obtain the best fitting time difference between the ages of genomes in the pairswe were comparing. We plot the results from all simulations, and the report value for the peak

point of the distribution, with minimum and maximum values obtained from the 5% simulations with the highest likelihood (Supplementary Table 17, Supplementary Figure 15).

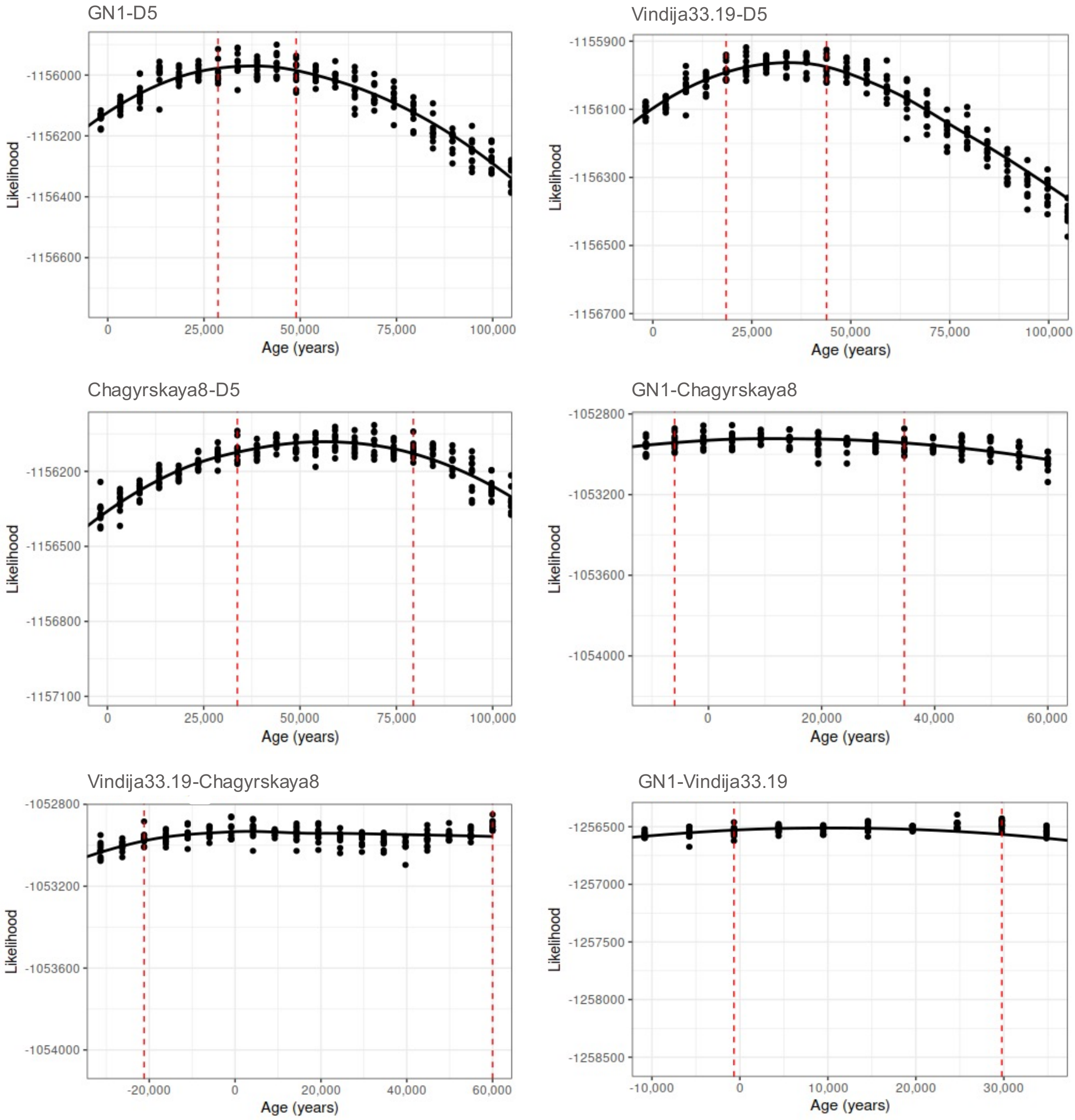

Supplementary Figure 15 Age estimates from ten generations using the indicated pairs. Ages for the younger genomes (G) estimated by assuming 120,000 years of age for D5, 60,000 years for Chagyrskaya 8, and 45,000 years for the Vindija 33.19 and GN1Neandertals. Red dashed lines represent the minimum and maximum values obtained from simulations with the best likelihood (top 5%).

Supplementary Table 17 Branch shortening estimates at the peak of the distribution for the genome in column named G and corresponding likelihood estimates. Minimum and maximum values correspond to the red dashed lines in Supplementary Figure 15, estimates from simulations with the best likelihood (top 5%).

**Genomes used**		**Estimated age for the focus genome (G)**			
**G*old***	**G**	**Peak of the distribution**	**Likelihood**	**Minimum**	**Maximum**
D5	GN1	37,772	-1,155,970	28,650	48,950
D5	Vindija 33.19	33,660	-1,155,963	18,500	43,875
D5	Chagyrskaya 8	56,273	-1,156,081	33,725	79,400
Chagyrskaya 8	GN1	10,663	-1,052,922	-5975	34,625
Chagyrskaya 8	Vindija 33.19	2,440	-1,052,932	-21,200	60,000
Vindija 33.19	GN1	10,053	-1,256,511	-675	29,775

We confirm the previous age difference estimates for the high-coverage Chagyrskaya 8 Neandertal using the demographic curves obtained for it and the D5 Neandertal, which we find to be around 60,000 years (Mafessoni et al., 2020). Similar ages were obtained for Vindija 33.19 and GN1 using the high-coverage D5 genome, with Vindija 33.19 having a slightly younger age estimate.

When using the Chagyrskaya 8 genome as G*old* to estimate the ages of the younger Neandertals, Vindija 33.19 and GN1, we observed a very flat distribution of the estimates from simulations, for all simulated times. This resulted in very large intervals of the top 5% with the highest likelihood, with lower estimates below zero. Similarly, when using the high-coverage genome of the Vindija 33.19 Neandertal as G*old* to estimate the age of GN1, we obtained a very large interval for the inferred ages, ranging from below zero to around 30,000 years, which is not plausible. The fact that the distribution is almost flat indicates resolution issues, most likely due to PSMC inherent limitations in inferring recent demography (i.e., the history close to the death of the individual whose genome is being analysed). For this reason, we are currently unable to estimate precise molecular dates for GN1 using PSMC. However, our results are consistent with it being a Late Neandertal and broadly contemporaneous with the Vindija 33.19 individual.

References

1. Li, H. & Durbin, R. Fast and accurate long-read alignment with Burrows–Wheeler transform. *Bioinformatics* **26**, 589–595 (2010).
2. Sümer, A. P. *et al.* Earliest modern human genomes constrain timing of Neanderthal admixture. *Nature* **638**, 711–717 (2025).
3. Prüfer, K. *et al.* A high-coverage Neandertal genome from Vindija Cave in Croatia. *Science* **358**, 655–658 (2017).
4. Prüfer, K. *et al.* The complete genome sequence of a Neanderthal from the Altai Mountains. *Nature* **505**, 43–49 (2014).
5. Mafessoni, F. *et al.* A high-coverage Neandertal genome from Chagyrskaya Cave. *Proc. Natl. Acad. Sci.* **117**, 15132–15136 (2020).
6. Meyer, M. *et al.* A High-Coverage Genome Sequence from an Archaic Denisovan Individual. *Science* **338**, 222–226 (2012).

7. Genetic diversity

7.1 Heterozygosity

We compared the heterozygosity of GN1 to those of the other high-coverage Neandertals. We used the filtered genotypes described in Section 3, as well as the filtered genotypes of three other high-coverage Neandertals: the ~120,000-year-old D5 Neandertal[1], the ~60,000-year-old Chagyrskaya 8[2], and ~45,000-year-old Vindija 33.19[3]; the Denisovan D3 individual[4]; and five modern humans: the ~45,000-year-old Zlatý kůň and Ranis 13[5], the ~44,000-year-old Ust'-Ishim[6], as well as the ~8,000-year-old individuals of Stuttgart (or "LBK") and Loschbour[7]. All of these individuals' genomes were genotyped and filtered in the same way as the GN1. To calculate the heterozygosity of each genome, we divided the number of observed heterozygous sites by the total number of sites. We repeated this calculation while also masking the regions inferred as homozygous by descent (HBD) in Section 7.2. In both cases the levels of heterozygosity observed in the GN1 genome fell within the Neandertal variation observed to date (Extended Data Fig. 3).

7.2 Inbreeding

We inferred HBD segments, i.e., regions of the genome with predominantly homozygous genotypes as the result of a recent shared ancestry, using a previously established method for both archaic and ancient humans[1,3,5]. This approach takes into account that such segments are not expected to be runs of homozygosity (ROH), due to genotyping errors or *de novo* mutations. The method includes a parameter π, reflecting the proportion of an HBD segment that is part of an ROH fragment(s). Following Sümer et al.[5], we defined HBD for a range of $\pi = \{0.8, 0.825, \ldots, 0.975, 0.99\}$, and selected the optimal value of π at which the proportion of the longer HBD fragments (≥10cM) is highest (Supplementary Figure 16). This strategy optimises the sensitivity for longer segments while reducing false positives for shorter segments (≥2.5cM and <10cM). Decreasing the parameter π would increase the likelihood of incorrect shorter segment identification. The inbreeding level was determined as the proportion of sites located in HBD segments, calculated by dividing the number of such sites by the total number of sites that passed our filtering criteria.

Since no direct recombination maps for archaic hominins are available, we used three strategies: a constant recombination rate of 1.3 cM/Mb, the African American or "AA" map[8], and the deCODE map[9]. All three maps yielded comparable results, with HBD estimates in the GN1 ranging from 5% to 25% of the genome, depending on π. This places GN1 within the known Neandertal variation, with levels comparable to the contemporaneous Vindija 33.19, and with lower inbreeding levels than Chagyrskaya 8 (Supplementary Figure 16).

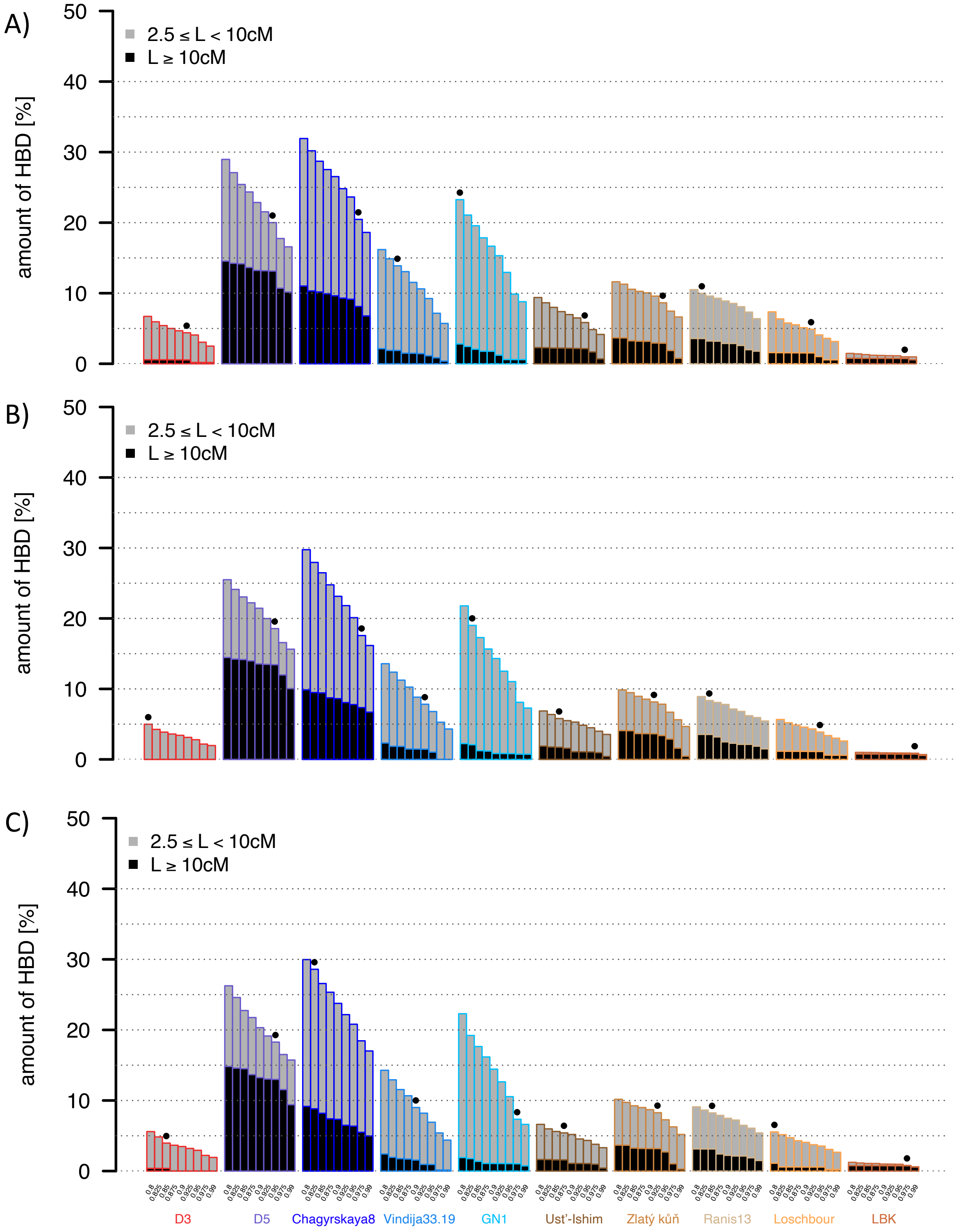

Supplementary Figure 16 Proportion of HBD over the genome of each archaic and ancient human individual, where each bar represents a different value for the parameter π, of which the optimal one is indicated with a dot on top. Black and gray bars indicate the inbreeding proportion in short and long HBD segments, respectively (see also Section 7.2). A) Constant recombination rate, B) "AA" map, and C) deCODE map.

References

1. Prüfer, K. *et al.* The complete genome sequence of a Neanderthal from the Altai Mountains. *Nature* **505**, 43–49 (2014).
2. Mafessoni, F. *et al.* A high-coverage Neandertal genome from Chagyrskaya Cave. *Proc. Natl. Acad. Sci.* **117**, 15132–15136 (2020).
3. Prüfer, K. *et al.* A high-coverage Neandertal genome from Vindija Cave in Croatia. *Science* **358**, 655–658 (2017).
4. Meyer, M. *et al.* A High-Coverage Genome Sequence from an Archaic Denisovan Individual. *Science* **338**, 222–226 (2012).
5. Sümer, A. P. *et al.* Earliest modern human genomes constrain timing of Neanderthal admixture. *Nature* **638**, 711–717 (2025).
6. Fu, Q. *et al.* Genome sequence of a 45,000-year-old modern human from western Siberia. *Nature* **514**, 445–449 (2014).
7. Lazaridis, I. *et al.* Ancient human genomes suggest three ancestral populations for present-day Europeans. *Nature* **513**, 409–413 (2014).
8. Hinch, A. G. *et al.* The landscape of recombination in African Americans. *Nature* **476**, 170–175 (2011).
9. Halldorsson, B. V. *et al.* Characterizing mutagenic effects of recombination through a sequence-level genetic map. *Science* **363**, eaau1043 (2019).

8. GN1 relation to modern humans

The peak of the last major event that contributed Neandertal ancestry to all present-day non-Africans took place ~47,000 years ago[1,2], resulting in putatively introgressed Neandertal segments in all modern human genomes from outside of sub-Saharan Africa sequenced to date[3–6]. Previous research identified the Vindija 33.19 Neandertal individual as the closest match for all of the Neandertal ancestry present in both ancient modern humans and present-day non-African populations[5,7]. However, in some ancient modern human genomes, such as the Initial Upper Palaeolithic individuals sequenced from Romania and Bulgaria[8,9], there is evidence supporting that part of their Neandertal ancestry derives from a different source populations that did not contribute to later modern human populations[1]. Considering that these modern humans overlapped in time with the GN1, we examined if the introgressed segments in modern human individuals – both ancient and present-day – are closer on average to Vindija 33.19, compared to GN1.

8.1 Archaic lineage assignment of introgressed segments

We examined the Neandertal introgressed segments in the autosomes of 59 ancient non-African modern human individuals and 231 non-African present-day individuals from the Simons Genome Diversity Project data set (SGDP)[10], as in Iasi et al.[1], with the addition of the high coverage genome of Zlatý kůň and Ranis 13[2]. We called the segments using *admixfrog* version 0.7.2[11]. We only retained segments longer than 0.2 cM for ancient and 0.05 cM for present-day individuals. We performed the archaic lineage assignment of these introgressed segments as described in Iasi et al.[1], relying on diagnostic sites that differed between six hominin reference individuals. The reference was composed of one present-day Mbuti individual (B_Mbuti-4)[10], four Neandertals – Neandertal D5 (ND5)[12], Chagyrskaya 8 (CHA)[6], Vindija 33.19 (VIN)[5] and the newly sequenced GN1 – as well as the Denisovan D3 (DEN)[13] genome. To assess that there are no biases when using different reference Neandertals when calling introgressed segments in *admixfrog*, we used two call sets: We compared the segments identified using present-day Sub-Saharan-Africans, the Denisovan and only the Neandertal D5 and Chagyrskaya Neandertals (ND5,CHA), to the segments identified using Neandertal D5, Chagyrskaya, Vindija and GN1 Neandertals (ND5,CHA,VIN,GN1) as a reference.

The set of diagnostic sites was constructed as follows. First, the genotypes for the archaic individuals were called using *snpAD* version 0.3.11[14]. For each individual, we removed tandem-repeats and indels. We only considered regions with uniquely mappable 35-mers and applied a GC-content conditioned coverage filter of 10x. After such filtering, we randomly sampled an allele from each individual for the sites where all individuals have non-missing genotypes. We used the chimpanzee (PanTro6), bonobo (PanPan1), gorilla (GorGor3) and orangutan (PanAbe3) reference genomes aligned to the hg19 human reference (downloaded from http://hgdownload.cse.ucsc.edu/goldenpath/hg19/), to determine the

ancestral alleles where at least three ape species had the same allele. We only retained sites where at least one of the reference hominins had a derived allele. Finally, we applied a last filter consisting of the mappability track map35_99 filter [12]. This resulted in 2,501,843 diagnostic positions.

We then performed random allele sampling at these diagnostic positions for our modern human test individuals. For ancient individuals, we required that the reads had a minimum mapping quality of 25 and a minimum length of 35 bp. For present-day individuals, we only sampled the genotypes with genotype quality score >= 1. From this, we computed the number of shared derived alleles matching a given hominin reference individual and the total number of derived sites across the Neandertal introgressed segments. This, in turn, allowed us to calculate the probability of matching said reference over all diagnostic positions ($\hat{p}$).

To this end we used a Bayesian approach whereby the number of derived genotypes (or in the case of ancient individuals, the number of reads) matching the reference is denoted as x_i and the total number of genotypes/reads of sites with at least one genotype/read being derived is k_i. The posterior distribution of x_i on an introgressed segment matching derived diagnostic positions of a reference Neandertal $\hat{p}$, is then given by using a binomial likelihood $x_i|p \sim Bin(x_i; k_i, p)$. Using a uniform prior for p yields the standard analytical solution for the posterior[15]:

$$\hat{p}|X \sim Beta(\sum x_i + 1, \sum k_i - \sum x_i + 1)$$

We took 2000 samples from the posterior. We calculated this both at the individual and at the population / continental level (as defined by the SGDP). In the latter case, we only consider non-overlapping segments. If there were multiple segments in a population overlapping a specific position, we only sampled one random individual for this position.

To compare if there is a difference in matching, we subtracted the samples of the posterior distribution of the matching to the reference Neandertal1 from the matching to the reference Neandertal2 (e.g. GN1 vs. Vindija 33.19). From this difference between these two posterior distributions, we compute the 95% highest posterior density intervals (HPDI) using the *rethinking* package in *R*. If the interval overlapped zero, we concluded that there was no significant difference between the matching to the two Neandertals. If it is positive, then reference Neandertal1 matches more closely the introgressing Neandertal population. And if it was negative, reference Neandertal2 matches more the introgressing Neandertal population.

Extended Data Fig. 9 and 10, left panel, show the mean difference between the posterior estimates for the matching of segments from the call set that called segments using only Neandertal D5 and

Chagyrskaya or all high coverage Neandertals on diagnostic positions for the 58 ancient individuals. We compared Vindija 33.19 vs. Neandertal D5, Vindija 33.19 vs. Chagyrskaya 8, GN1 vs. Chagyrskaya 8 and Vindija 33.19 vs. GN1. The error bars indicate the corresponding 95% HPDI. For the comparisons of Vindija 33.19 vs. Neandertral D5, Vindija 33.19 vs. Chagyrskaya 8 and GN1 vs. Chagyrskaya 8, the majority of the posterior matchings are significantly closer to Vindija 33.19 or GN1 for both call sets.

When comparing Vindija 33.19 to GN1 the differences in matching are very subtle, resulting from the observation that the two individuals stem from closely related Neandertal populations (Supplementary Section 6). For the comparison of Vindija 33.19 vs. GN1 we found 15 individuals closer to Vindija 33.19. For the remaining 43 ancient modern humans, the posterior estimates are not significantly different from one another. Since the majority of Neandertal ancestry is thought of stemming from one event with one group of Neandertals[1], we can pool all individuals together to get more sites and thus increase the power of our analyses. Doing that results in the posterior distribution of the introgressed segments matching closer to Vindija 33.19 when compared to GN1 for both *admixfrog* call sets. For present-day populations we find a similar pattern of a majority being closer to Vindija 33.19 or GN1 when compared to Neandertal D5 or Chagyrskaya 8, but mostly inconclusive when comparing Vindija 33.19 to GN1 (Supplementary Figures 17 and 18, left panel).

To gain power, we grouped the SGDP individuals into their continental superpopulations (Supplementary Figures 17 and 18, middle panel). Oceanians are the least closest to Vindija 33.19 versus GN1 compared to the other superpopulations (still with > 50% of the HPDI tending to Vindija 33.19). Misclassifications of Denisovan ancestry to Neandertal when classifying using *admixfrog*, which might obscure the signal (as seen in Iasi et al.[1]). The combined Neandertal segments in South-Asians, however, are significantly closer to Vindija 33.19. This could be an effect of having more power since they have the largest number of observations, possibly due to them harbouring more unique Neandertal ancestry[16]. Since the single extended period of Neandertal admixture was estimated to happen potentially over millenias, the Out-of-Africa population might have been structured. Hence, we can not exclude scenarios of additional minor contributions of Neandertal groups more distant from Vindija 33.19 and potentially closer to GN1 into the ancestors of West-Eurasians, Americans and East-Asians, while South Asians and potentially Oceanians retained more Vindija like ancestry[1]. Nevertheless, when pooling all individuals together, we find again that they are closer to Vindija 33.19 compared to GN1 (Supplementary Figures 17 and 18, right panel), indicating that the majority of Neandertal ancestry was contributed from a Vindija-like population.

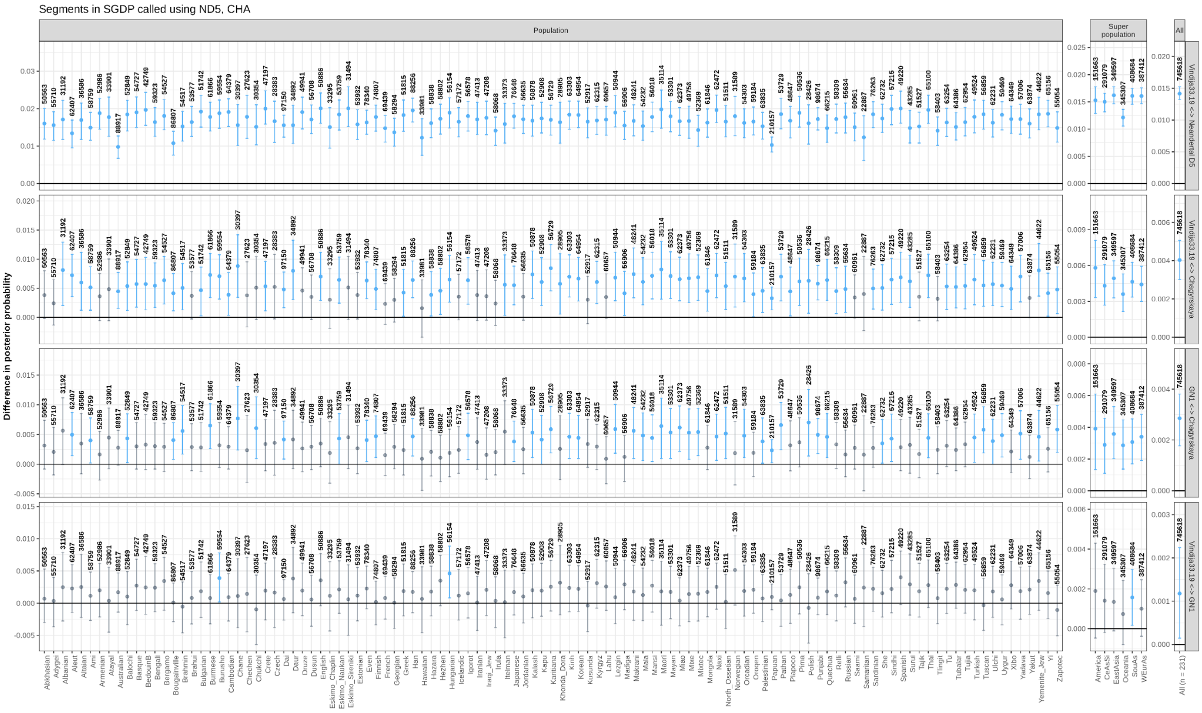

Supplementary Figure 17 Difference in posterior estimates of the probability of derived alleles on an introgressed segment matching the diagnostic positions of: Vindija 33.19 vs. Neandertal D5, Vindija 33.19 vs. Chagyrskaya 8, GN1 vs. Chagyrskaya 8 and Vindija 33.19 vs. GN1 Neandertals, for 231 diverse present-day non-African individuals from the SGDP data set clustered into populations, continental superpopulations (SouAs = South-Asia, WEurAs = West-Eurasia, CeAsSi = Central-Asia and Siberia) and all individuals pooled together. The segments were called using the Neandertal D5 and Chagyrskaya 8 Neandertals as reference for Neandertals. Points indicate the mean posterior difference and error bars indicate the 95% HPDI. Blue coloured populations' estimates indicate a significantly better match to one of the Neandertals. Numbers on top give the total amount of unique positions across all introgressed segments.

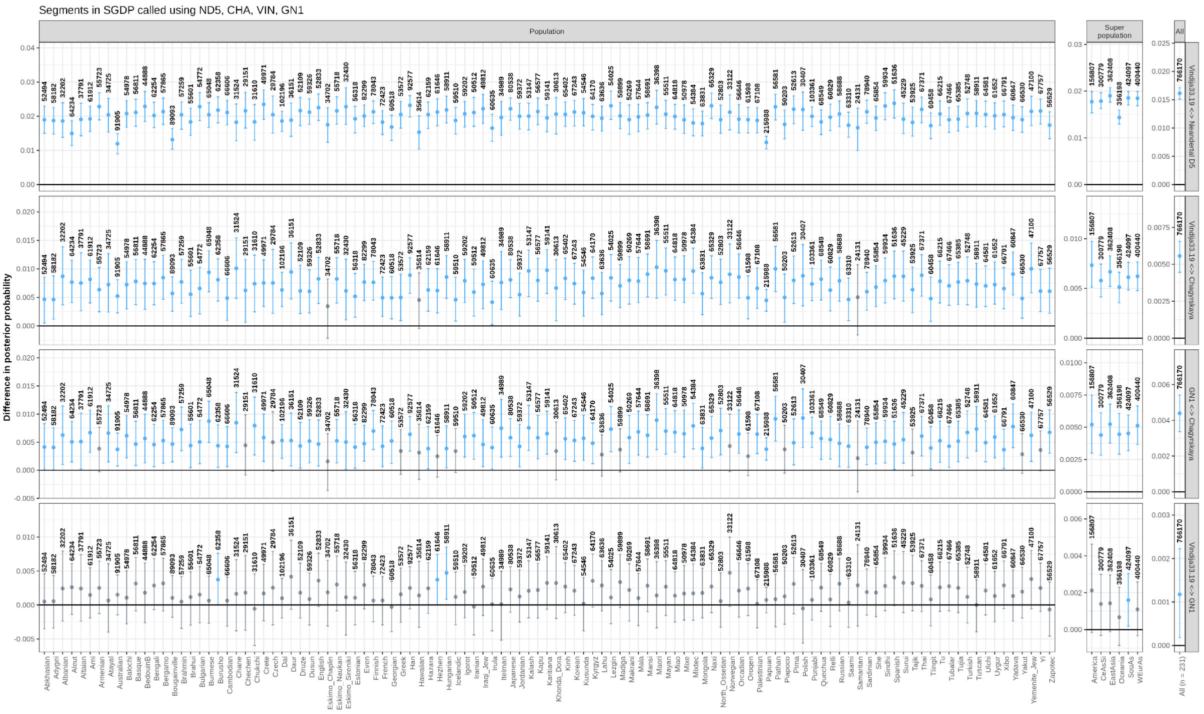

Supplementary Figure 18 Difference in posterior estimates of the probability of derived alleles on an introgressed segment matching the diagnostic positions of: Vindija 33.19 vs. Neandertal D5, Vindija 33.19 vs. Chagyrskaya 8, GN1 vs. Chagyrskaya 8 and Vindija 33.19 vs. GN1 Neandertals, for 231 diverse present-day non-African individuals from the SGDP data set clustered into populations, continental superpopulations (SouAs = South-Asia, WEurAs = West-Eurasia, CeAsSi = Central-Asia and Siberia) and all individuals pooled together. The segments were called using the Neandertal D5, Chagyrskaya 8, Vindija 33.19 and GN1 Neandertals as reference for Neandertals. Points indicate the mean posterior difference and error bars indicate the 95% HPDI. Blue coloured populations' estimates indicate a significantly better match to one of the Neandertals. Numbers on top give the total amount of unique positions across all introgressed segments.

8.2 Stratified D-statistics

We further investigated the relationship of introgressed Neandertal segments in present-day humans to the high coverage Neandertals using the genome-wide ABBA-BABA or D-statistics, measuring the amount of imbalance in non-canonical gene trees between 4 population (i.e., trees not following the overall phylogeny)[17,18]. We used the *snpAD* calls for the Neandertal D5, Vindija 33.19, Chagyrskaya 8 and GN1 Neandertals together with all individuals from the 1000 Genomes project (1KG).

[19]. The panTro4 Chimpanzee reference genome was used as an outgroup. We followed [6] and calculated D(A,B,C,O) as:

$$D = \frac{\sum \quad (p(ABBA) - p(BABA))}{\sum \quad (p(BABA) + p(ABBA))}$$

with:

$$p(ABBA) = (1 - f_A)f_B f_C(1 - f_O) + f_A(1 - f_C)f_O$$
$$p(BABA) = f_A(1 - f_B)f_C(1 - f_O) + (1 - f_A)f_B(1 - f_C)f_O$$

With f_x being the allele frequency of the population at a given position. We calculated standard errors (*SE*) using a weighted block jackknife approach in windows of 5 Mb[20], with *Z* scores calculated as $Z = D / SE$. We restricted the analysis to transversions only.

We calculated the D-statistics to test which Neandertal is closer to the introgressing Neandertal population in the form of A = Neandertal1, B = Neandertal2, C = 1KG superpopulation and O = Chimp. We also pooled all non-African individuals (all individuals from AMR, EAS, EUR and SAS) into one group (all_nonAFR) and we excluded African-Americans (ASW) and Afro-Caribbeans (ACB) groups from the AFR superpopulation (AFRred).

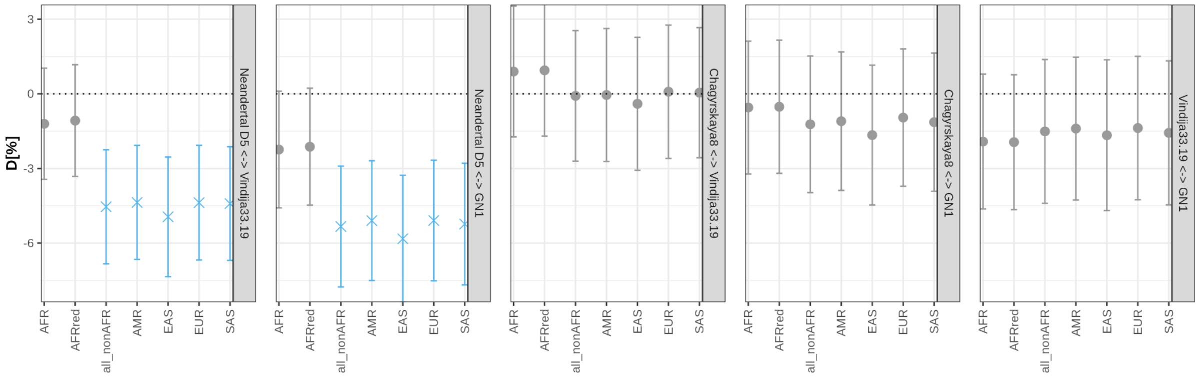

Supplementary Figure 19 Genome wide D-statistic as a relative sharing of alleles of 1000 Genomes superpopulations (x-axis) with: Vindija 33.19 vs. Neandertal D5, GN1 vs. Neandertal D5, Vindija 33.19 vs. Chagyrskaya 8, GN1 vs. Chagyrskaya 8 and Vindija 33.19 vs. GN1. Super-populations are: Africans (AFR), Africans excluding African-Americans and Afro-Caribbeans (AFRred), all non-Africans (all_nonAFR), Americans (AMR), East Asians (EAS), Europeans (EUR) and South Asians (SAS) and all AMR, EAS, EUR and SAS into all non-African (all_nonAFR). D-statistics are in the form D(Neanderthal 1, Neanderthal 2, 1KG superpopulation, Chimp). Blue coloured estimates indicate a significant deviation from 0 (Z > 2, X shaped points indicate Z > 3. Error bars indicate 2 standard errors.

In Supplementary Figure 19 we observe a clear signal that Vindija 33.19 and GN1 are both closer to the Introgressing Neandertal than Neandertal D5. However, there are no significant differences between the other comparisons. Since introgressed alleles are usually in low frequency, we investigated the D-statistics by stratifying the derived allele frequency in the present-day human population into windows of 0.05 for the comparisons of Vindija 33.19 and GN1 to Chagyrskaya 8 and to each other. We determined the derived status of an allele using the Chimp.

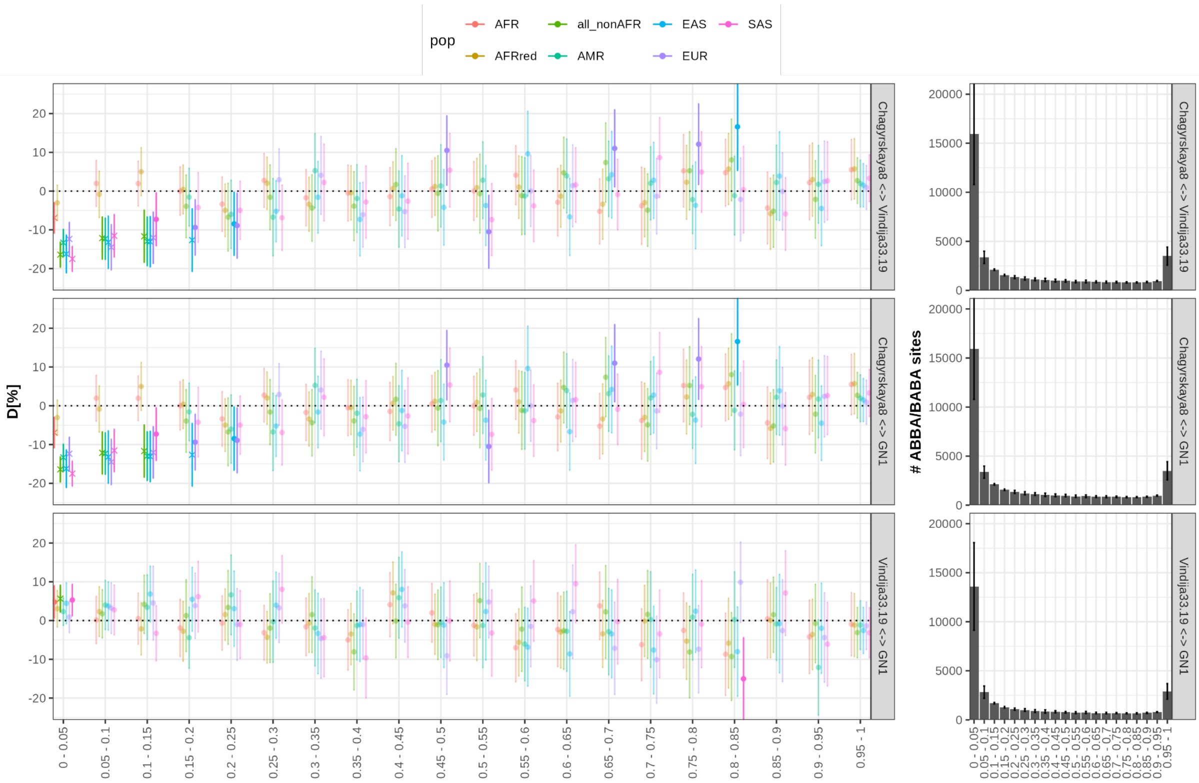

Supplementary Figure 20 Stratified D-statistic for 1000 Genomes super-populations with: Vindija 33.19 vs. Chagyrskaya, GN1 vs. Chagyrskaya and Vindija 33.19 vs. GN1 (left panel). Right panel indicates the number of ABBA and/or BABA sites per derived allele frequency window. Super-populations are: Africans (AFR), Africans excluding African-Americans and Afro-Caribbeans (AFRred), all non-Africans (all_nonAFR), Americans (AMR), East Asians (EAS), Europeans (EUR) and South Asians (SAS) and all AMR, EAS, EUR and SAS into all non-African (all_nonAFR). D-statistics are in the form D(Neandertal 1, Neandertal 2; 1KG superpopulation, Chimp). Full coloured populations' estimates indicate a significant deviation from 0 ($Z > 2$). X shaped points indicate $Z > 3$. Error bars indicate 2 standard errors.

We replicated the findings from Mafessioni et al. 2020 showing that in lower allele frequency windows, Vindija 33.19 is closer to the introgressing Neandertal than Chagyrskaya 8 is. Similarly, we find that GN1 is also closer to the introgressing Neandertal population than Chagyrskaya 8 is (Supplementary Figure 20). When restricting the D-statistic to sites with derived allele frequency less than 10% we can conclude that Vindija 33.19 and GN1 are closest to the introgressing Neandertals (Supplementary Figure 21). In line with the results from the matching of the introgressed segments, South Asians are closer to Vindija 33.19 ($Z > 2$), and the same is true when pooling all non-African individuals.

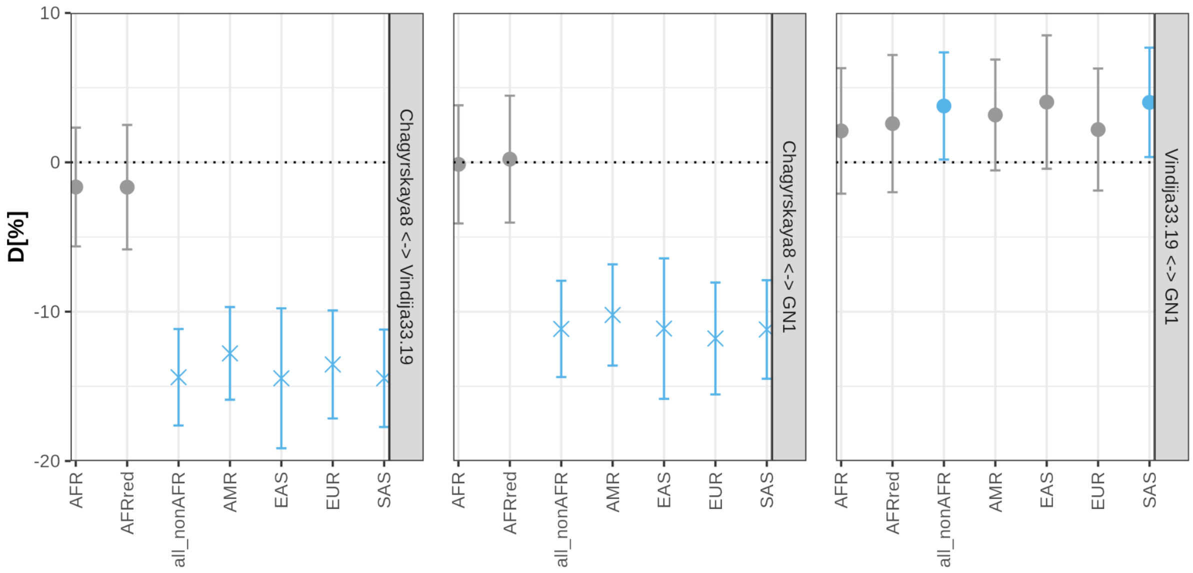

Supplementary Figure 21 D-statistic for alleles with derived frequency < 10 % as a relative sharing of alleles of 1000 Genomes super-populations with: Vindija 33.19 vs. Chagyrskaya 8, GN1 vs. Chagyrskaya 8 and Vindija 33.19 vs. GN1. Super-populations are: Africans (AFR), Africans excluding African-Americans and Afro-Caribbeans (AFRred), all non-Africans (all_nonAFR), Americans (AMR), East Asians (EAS), Europeans (EUR) and South Asians (SAS) and all AMR, EAS, EUR and SAS into all non-African (all_nonAFR). D-statistics are in the form D(Neandertal 1, Neandertal 2; 1KG superpopulation, Chimp). Blue coloured estimates indicate a significant deviation from 0 (Z > 2). X shaped points indicate Z > 3. Error bars indicate 2 standard errors.

References

1. Iasi, L. N. M. *et al.* Neanderthal ancestry through time: Insights from genomes of ancient and present-day humans. *Science* **386**, eadq3010 (2024).
2. Sümer, A. P. *et al.* Earliest modern human genomes constrain timing of Neanderthal admixture. *Nature* **638**, 711–717 (2025).
3. Vernot, B. & Akey, J. M. Resurrecting Surviving Neandertal Lineages from Modern human Genomes. *Science* **343**, 1017–1021 (2014).
4. Sankararaman, S. *et al.* The genomic landscape of Neanderthal ancestry in present-day humans. *Nature* **507**, 354–357 (2014).
5. Prüfer, K. *et al.* A high-coverage Neandertal genome from Vindija Cave in Croatia. *Science* **358**, 655–658 (2017).
6. Mafessoni, F. *et al.* A high-coverage Neandertal genome from Chagyrskaya Cave. *Proc. Natl. Acad. Sci. U.S.A.* **117**, 15132–15136 (2020).
7. Mafessoni, F. *et al.* A high-coverage Neandertal genome from Chagyrskaya Cave. *PNAS* (2020) doi:10.1073/pnas.2004944117.

8. Fu, Q. *et al.* An early modern human from Romania with a recent Neanderthal ancestor. *Nature* **524**, 216–219 (2015).
9. Hajdinjak, M. *et al.* Initial Upper Palaeolithic humans in Europe had recent Neanderthal ancestry. *Nature* **592**, 253–257 (2021).
10. Mallick, S. *et al.* The Simons Genome Diversity Project: 300 genomes from 142 diverse populations. *Nature* **538**, 201–206 (2016).
11. Peter, B. M. 100,000 years of gene flow between Neandertals and Denisovans in the Altai mountains. 2020.03.13.990523 Preprint at https://doi.org/10.1101/2020.03.13.990523 (2020).
12. Prüfer, K. *et al.* The complete genome sequence of a Neanderthal from the Altai Mountains. *Nature* **505**, 43–49 (2014).
13. Meyer, M. *et al.* A High-Coverage Genome Sequence from an Archaic Denisovan Individual. *Science* **338**, 222–226 (2012).
14. Prüfer, K. snpAD: an ancient DNA genotype caller. *Bioinformatics* **34**, 4165–4171 (2018).
15. Lee, P. M. *Bayesian Statistics: An Introduction*. (Arnold, London, 1997).
16. Witt, K. E., Villanea, F., Loughran, E., Zhang, X. & Huerta-Sanchez, E. Apportioning archaic variants among modern populations. *Philos Trans R Soc Lond B Biol Sci* **377**, 20200411 (2022).
17. Patterson, N. *et al.* Ancient Admixture in Human History. *Genetics* **192**, 1065–1093 (2012).
18. Green, R. E. *et al.* A Draft Sequence of the Neandertal Genome. *Science* **328**, 710 (2010).
19. The 1000 Genomes Project Consortium *et al.* A global reference for human genetic variation. *Nature* **526**, 68 (2015).
20. Busing, F. M. T. A., Meijer, E. & Leeden, R. V. D. Delete-m jackknife for unequal m. *Statistics and Computing* **9**, 3–8 (1999).

9. Genetic screening

9.1 Sampling, DNA extraction and library preparation

All Neandertal remains were sampled in dedicated clean room facilities. The majority of the remains from archaeological sites in Wallonia (Belgium) were sampled in the Royal Belgian Institute of Natural Sciences in Brussels (Belgium), including Première Caverne du Bay Bonnet (or **Fonds-de-Forêt**) (Trooz, Liège Province), Troisième caverne of **Goyet** (Gesves, Namur Province) and **Spy** Cave (Jemeppe-sur-Sambre, Namur Province). Two of the specimens were sampled at the University of Tübingen (Germany): Goyet Q305-1 from Goyet, and TM2422-36 from **Trou Magrite** (Pont-à-Lesse, Namur Province). Specimens from Trou de l'Abîme (**Couvin**, Namur Province), Schmerling Cave or Deuxième Caverne of **Engis** (Flémalle, Liège Province) and **Walou** Cave (Trooz, Liège Province), as well as a subset of the remains from Goyet, and all of the specimens from the French archaeological sites of Grotte du Renne (**Arcy-sur-Cure**, Yonne), La Roche-à-Pierrot (**Saint-Césaire**, Charente-Maritime) and **Les Cottés** (Saint-Pierre-de-Maillé, Vienne) were sampled at the Max Planck Institute for Evolutionary Anthropology (Leipzig, Germany).

In total, this screening effort comprised 35 different skeletal elements (Supplementary Data Table 1.1). We used a sterile dentistry drill to remove a thickness of ~1 mm of material over a surface area of approximately (or up to) 2 × 2 mm, and then collected between 1 and 57.1 mg of skeletal powder by drilling deeper into the bone. In order to maximise the chances of obtaining an optimal sample, and given the heterogeneity of aDNA preservation within bones and teeth[1,2], we used a micro-sampling approach. This consisted in taking multiple small samples of ~10 mg at different locations over surface areas of usually circa 2 x 2 mm across the skeletal element, instead of taking a larger sample from a single spot. The rationale behind this strategy was to minimise the risk of diluting DNA-rich areas with bone powder from regions of lower quality or with higher levels of present-day human contamination. As a result, an average of four micro-samples were extracted from each skeletal element.

Over a third of the powdered samples underwent a pre-treatment step prior to DNA extraction, exposing the powder to either 0.5% hypochlorite solution or a phosphate buffer[3] (Supplementary Data Table 1.1). These pre-treatments have been shown to effectively reduce exogenous DNA contamination, at the cost of at times also decreasing the levels of endogenous DNA recovered. For the rest of the samples, the bone or tooth powder was lysed directly without any pre-treatment.

We used DNA extraction and purification steps tailored for the recovery of highly degraded DNA[4]. In detail, buffer "D" was used either in a manual spin column purification or using automated purification

with silica-coated paramagnetic beads on an Agilent Technologies Bravo NGS workstation B. This resulted in a total of 134 different extracts.

Relying on a combination of manual and automatised protocols, these extracts were then converted into single-stranded DNA libraries [5,6]. We used between 5 µl and 30 µl of extract as input for the library preparation (Supplementary Data Table 1.1), resulting in 189 libraries. A subset of the earliest libraries (27 out of 189) underwent a modified version of a single-stranded DNA library-preparation protocol, employing partial uracil–DNA–glycosylase (UDG) and Endonuclease VIII (Endo VIII) treatment (i.e., USER treatment), which reduced the abundance of Uracil bases within ancient sequences while preserving partial deamination signals at the sequence alignment ends[5].

The number of unique sequences in each library was quantified using quantitative PCR (qPCR) or digital-droplet PCR (ddPCR). Moreover, since a control oligonucleotide had been spiked into each reaction, it was also possible to infer the efficiency of library preparation[7]. From this point onwards, for the majority of the samples these procedures were automated on an Agilent Bravo NGS Workstation, unless it is specified in Supplementary Data Table 1.1 that they were done manually. To allow for multiplex sequencing, libraries were amplified and barcoded using unique combinations of indices of 7 or 8 base pairs (bp). Therefore, after purifying[8], it was possible to sequence pooled libraries together[9,10]. This also included extraction and library negative controls, which had been processed alongside the samples in all of the preceding steps to monitor the levels of possible modern DNA contamination.

9.2 Sequencing and bioinformatic analysis

To evaluate levels of endogenous DNA preservation in each library, we used shallow shotgun sequencing on Illumina MiSeq and HiSeq platforms, using double-index pair-end configurations. The bioinformatic processing of these data follows[11]. First, bases were called using Illumina's software *Bustard*. Next, *leeHom* was used for trimming the adaptors and merging overlapping pair-end reads into single sequences[12]. These sequences were then aligned to the GRCh37 (hg19) human reference genome[13] using the Burrows-Wheeler Aligner version 0.5.10[14] with parameters adjusted for ancient DNA[15]. For de-multiplexing of each sequencing run, we used an in-house script that separated libraries based on the expected index combinations, and removed PCR duplicates using bam-rmdup (https://github.com/mpieva/biohazard-tools). The final quality-control filters included a minimum sequence length of 35 bp and a minimum mapping quality of 25. An overview of the summary statistics for shotgun data can be found in Supplementary Data Table 1.1.

We investigated the presence or absence of authentic ancient DNA (aDNA) by analysing the damage patterns at terminal positions of the alignment, in the form of an increase of 'C-to-T' substitutions (or

‘G-to-A’ in the case of the reverse strand) at the 5’ and 3’ ends of the sequences. If more than 10% of the terminal bases at either end of the read showed evidence of deamination, we considered this as evidence that aDNA was preserved. Out of the 189 prepared libraries, 175 were shotgun sequenced, and 112 showed evidence of aDNA preservation.

It is possible to surmise the contamination levels in a given library by evaluating how the deamination values at the ends of the sequence alignments change after conditioning for deamination at one of the ends. As described in[16], if the conditional deamination differs notably from the initial deamination levels, this suggests a mixture of ancient and modern molecules. Moreover, the complexity of each library was estimated by calculating the product of the number of mappable fragments with the average fragment length. All these factors were taken into account when deciding which libraries would make candidates for downstream nuclear DNA captures. All of the constructed DNA libraries were further enriched for the human mitochondrial DNA, even if the shotgun data indicated poor preservation of ancient nuclear DNA and/or high levels of present-day DNA contamination, in order to at minimum determine the hominin group the skeletal remains belong to.

References

1. Hajdinjak, M. *et al.* Reconstructing the genetic history of late Neanderthals. *Nature* **555**, 652–656 (2018).
2. Prüfer, K. *et al.* A high-coverage Neandertal genome from Vindija Cave in Croatia. *Science* **358**, 655–658 (2017).
3. Korlević, P. & Meyer, M. Pretreatment: Removing DNA Contamination from Ancient Bones and Teeth Using Sodium Hypochlorite and Phosphate. in *Ancient DNA: Methods and Protocols* 15–19 (Springer New York, New York, NY, 2019). doi:10.1007/978-1-4939-9176-1.
4. Rohland, N., Glocke, I., Aximu-Petri, A. & Meyer, M. Extraction of highly degraded DNA from ancient bones, teeth and sediments for high-throughput sequencing. *Nat. Protoc.* **13**, 2447–2461 (2018).
5. Gansauge, M.-T. & Meyer, M. Single-stranded DNA library preparation for the sequencing of ancient or damaged DNA. *Nat. Protoc.* **8**, 737–748 (2013).
6. Gansauge, M.-T., Aximu-Petri, A., Nagel, S. & Meyer, M. Manual and automated preparation of single-stranded DNA libraries for the sequencing of DNA from ancient biological remains and other sources of highly degraded DNA. *Nat. Protoc.* **15**, 2279–2300 (2020).
7. Glocke, I. & Meyer, M. Extending the spectrum of DNA sequences retrieved from ancient bones and teeth. *Genome Res.* **27**, 1230–1237 (2017).
8. Slon, V. *et al.* Neandertal and Denisovan DNA from Pleistocene sediments. *Science* **356**, 605–608 (2017).

9. Kircher, M., Sawyer, S. & Meyer, M. Double indexing overcomes inaccuracies in multiplex sequencing on the Illumina platform. *Nucleic Acids Res.* **40**, e3 (2012).
10. Zavala, E. I. *et al.* Quantifying and reducing cross-contamination in single- and multiplex hybridization capture of ancient DNA. *Mol. Ecol. Resour.* **22**, 2196–2207 (2022).
11. Skov, L. *et al.* Genetic insights into the social organization of Neanderthals. *Nature* **610**, 519–525 (2022).
12. Renaud, G., Stenzel, U. & Kelso, J. leeHom: adaptor trimming and merging for Illumina sequencing reads. *Nucleic Acids Res.* **42**, e141 (2014).
13. Church, D. M. *et al.* Modernizing Reference Genome Assemblies. *PLoS Biol.* **9**, e1001091 (2011).
14. Li, H. & Durbin, R. Fast and accurate long-read alignment with Burrows–Wheeler transform. *Bioinformatics* **26**, 589–595 (2010).
15. Meyer, M. *et al.* A High-Coverage Genome Sequence from an Archaic Denisovan Individual. *Science* **338**, 222–226 (2012).
16. Meyer, M. *et al.* Nuclear DNA sequences from the Middle Pleistocene Sima de los Huesos hominins. *Nature* **531**, 504–507 (2016).

10. Mitochondrial captures

10.1 Mitochondrial DNA enrichment and sequencing

We enriched a total of 180 sample libraries from 31 skeletal elements and 156 control libraries for human mitochondrial DNA (mtDNA) following published protocols[1,2]. We used two rounds of in-solution hybridisation capture and a probe-set that tiled the revised Cambridge Reference Sequence (rCRS,NC_01290)[3]. We captured the library A57813 from Goyet Q54-4 using a probe-set that included mitochondrial genomes of 241 mammalian species in addition to the rCRS[4]. The results presented in Section 10.3 indicated that the obtained data were comparable to the rest of the human mitochondrial captures.

All mtDNA enriched libraries were sequenced on Illumina MiSeq or HiSeq platforms. Raw reads were merged and trimmed using leeHom[5]. The generated sequences were mapped to both the rCRS and to the Vindija 33.16 Neandertal mitochondrial genome[6] using BWA version 0.5.10[7] with ancient DNA parameters[8]. Since the mitochondrial genome is circular, bases near the beginning and the end of the linear reference are harder to map and analyse. To overcome this, we re-mapped sequences to an elongated version of Vindija 33.16[9]. We used unique 8-base pair combinations of the index sequences for demultiplexing. We removed PCR duplicates using *bam-rmdup* (https://github.com/mpieva/biohazard-tools), and filtered for unique sequences with a minimum length of 35 bp and a mapping quality of 25.

10.2 Mitochondrial DNA data evaluation

We used the same criteria as for the shallow-shotgun data to evaluate aDNA preservation in each library, i.e., we used the presence of at least 10% deamination at both ends of the sequences to be evidence for the presence of authentic aDNA preservation (Supplementary Data Table 2). A total of 143 out of 180 libraries showed evidence of ancient mtDNA preservation, and none of the negative controls passed that threshold.

To determine whether specimens carried mtDNA sequences of Neandertal or modern human type, and subsequently to estimate to what extent specimens were contaminated with present-day human DNA, we relied on a set of "lineage-diagnostic positions" previously described[10]. We counted the number of fragments supporting the Neandertal state as defined by ten Neandertals, and the number of fragments supporting the modern human state as defined by 311 modern humans from across the world with a minimum allele frequency of 99%. The results of this analysis can be found in Supplementary Data Table 2.

We evaluated two different thresholds to determine how much of present-day human DNA contamination can be tolerated without substantially affecting downstream analyses. We used a maximum of 10% and a maximum of 25% of the sequences assigned to modern humans, i.e., putatively stemming from present-day contamination. Next, we estimated contamination twice for each library: once using all data, and once after filtering for sequences with evidence of aDNA damage, i.e., inferred deamination within the first three and last three terminal bases of each sequence. This resulted in evaluating four different criteria for curating suitable data for downstream analyses: (1) a maximum contamination of 25%, while using all sequences, (2) a maximum contamination of 25%, while using deaminated sequences only, (3) a maximum contamination of 10%, while using all sequences, and (4) a maximum contamination of 10%, while using deaminated sequences only.

To be more conservative, we estimated the contamination for each library using the sequences aligned to the rCRS, which retained more contaminant sequences than Neandertal-reference alignments (Supplementary Data Table 2.1). While most libraries followed this pattern, there were three exceptions. The Walou premolar and the AR-14 skull fragment from Arcy-sur-Cure showed higher contamination estimates for the Vindija 33.16 alignment than the rCRS alignment. While using the rCRS alignment, the libraries from these two specimens passed the contamination filters with levels under 25%, while with the Vindija 33.16 alignment we estimated present day human contamination of ~30%. Similarly, we estimated 9.8% present day human DNA contamination when aligned to rCRS, and 16.5% when aligned to Vindija 33.16 (Supplementary Data Table 2.2) for the Couvin molar. We therefore interpret results from these libraries with caution.

Based on these criteria, we merged libraries that showed acceptable levels of contamination and excluded the rest of the libraries from downstream analysis. Specifically, 95 libraries passed criteria (1), 137 passed criteria (2), 65 passed criteria (3), and 121 passed criteria (4). The complete summary statistics are reported in (Supplementary Data Tables 2.3 and 2.4), and an overview of the contamination estimates and coverages of the used data after merging by specimen are reported in Supplementary Tables 18 and 19.

Supplementary Table 18 Summary statistics after merging libraries aligned to the Vindija 33.16 mitochondrial genome with less than 25% present day human DNA contamination. Cells with "-" indicate that no libraries met the required thresholds for that specimen. Values in red indicate higher contamination levels in comparison to the values obtained with the rCRS alignment. CI - confidence intervals

**Samples**	All sequences		Deaminated sequences	
	**Average coverage**	**Contamination (%) [95% binomial CI]**	**Average coverage**	**Contamination (%) [95% binomial CI]**
AR-14	-	-	15.4	28.11 [25.33 - 31.02]
AR-30	116.5	2.64 [2.30 - 3.02]	46.1	4.29 [3.60 - 5.07]
Goyet Q305-7	1,370.5	6.6 [6.44 - 6.77]	332.7	2.95 [2.72 - 3.20]
Goyet Q56-1	1,290.6	8.35 [8.17 - 8.53]	186.0	2.7 [2.42 - 3.01]
Goyet Q374a-1	3,713.5	3.65 [3.58 - 3.73]	1,044.5	2.67 [2.55 - 2.80]
Spy 94a	37.8	5.28 [4.39 - 6.29]	13.3	10.02 [8.04 - 12.31]
Spy 8	202.2	18.3 [17.64 - 18.97]	45.3	5.46 [4.65 - 6.37]
Spy 572a	-	-	3.4	11.47 [7.56 - 16.46]
Goyet Q54-4	466.0	5.71 [5.46 - 5.97]	121.0	2.49 [2.16 - 2.85]
Goyet C5-1	138.5	6.63 [6.15 - 7.15]	48.6	5.5 [4.74 - 6.35]
Goyet Q376-9	61.6	3.44 [2.92 - 4.03]	19.3	7.86 [6.45 - 9.47]
Goyet Q57-1	304.3	9.42 [9.01 - 9.84]	70.3	3.26 [2.73 - 3.86]
Goyet Q57-2	4,224.9	5.79 [5.70 - 5.88]	1,010.5	2.76 [2.63 - 2.89]
Goyet Q57-3	2,509.0	11.99 [11.83 - 12.14]	513.1	2.65 [2.48 - 2.83]
Goyet 1424-3D	66.8	14.84 [13.79 - 15.94]	18.5	3.64 [2.61 - 4.92]
Goyet Q376-25	60.3	18.11 [16.98 - 19.30]	17.9	8.96 [7.41 - 10.70]
Fonds-de-Forêt 1	643.2	4.78 [4.57 - 4.98]	153.0	2.02 [1.75 - 2.32]
Goyet Q55-4	202.7	11.19 [10.67 - 11.73]	87.2	4.91 [4.35 - 5.51]

Goyet Q305-4	1,044.7	6.36 [6.18 - 6.54]	225.4	3.43 [3.14 - 3.74]
Goyet Q119-2	118.4	6.21 [5.70 - 6.75]	35.6	4.16 [3.40 - 5.04]
Walou	-	-	18.1	31.4 [28.68 - 34.21]
Couvin	-	-	17.4	16.48 [14.39 - 18.74]
Saint-Césaire 1	-	-	23.4	14.33 [12.67 - 16.12]

Supplementary Table 19 Summary statistics after merging libraries aligned to the Vindija 33.16 with less than 10% present day human DNA contamination. Cells with "-" indicate that no libraries met the required thresholds for that specimen. Values in red indicate higher contamination levels in comparison to the values obtained with the rCRS alignment. CI - confidence intervals

Samples	All sequences		Deaminated sequences	
	Average coverage	Contamination [%] [95% binomial CI]	Average coverage	Contamination [%] [95% binomial CI]
AR-14	-	-	-	-
AR-30	116.5	2.64 [2.30 - 3.02]	35.3	1.13 [0.74 - 1.64]
Goyet Q305-7	982.6	2.57 [2.45 - 2.70]	332.7	2.95 [2.72 - 3.20]
Goyet Q56-1	850.1	4.23 [4.07 - 4.40]	186.0	2.7 [2.42 - 3.01]
Goyet Q374a-1	3,713.5	3.65 [3.58 - 3.73]	1,044.5	2.67 [2.55 - 2.80]
Spy 94a	37.8	5.28 [4.39 - 6.29]	10.5	2.84 [1.66 - 4.51]
Spy 8	-	-	45.3	5.46 [4.65 - 6.37]
Spy 572a	-	-	3.8	0 [0.00 - 13.23]
Goyet Q54-4	466.0	5.71 [5.46 - 5.97]	121.0	2.49 [2.16 - 2.85]
Goyet C5-1	82.9	2.48 [2.09 - 2.91]	48.6	5.5 [4.74 - 6.35]
Goyet Q376-9	61.6	3.44 [2.92 - 4.03]	11.1	1.63 [0.84 - 2.82]
Goyet Q57-1	117.0	3.85 [3.42 - 4.31]	70.3	3.26 [2.73 - 3.86]
Goyet Q57-2	3,576.7	3.64 [3.56 - 3.71]	997.0	2.62 [2.49 - 2.75]
Goyet Q57-3	455.5	7.65 [7.36 - 7.96]	513.1	2.65 [2.48 - 2.83]

Goyet 1424-3D	-	-	18.5	3.64 [2.61 - 4.92]
Goyet Q376-25	-	-	10.6	3.74 [2.48 - 5.39]
Fonds-de-Forêt 1	643.2	4.78 [4.57 - 4.98]	153.0	2.02 [1.75 - 2.32]
Goyet Q55-4	42.8	7.2 [6.27 - 8.22]	87.2	4.91 [4.35 - 5.51]
Goyet Q305-4	583.9	4.03 [3.83 - 4.22]	225.4	3.43 [3.14 - 3.74]
Goyet Q119-2	77.3	1.83 [1.49 - 2.23]	35.6	4.16 [3.40 - 5.04]
Walou	-	-	-	-
Couvin	-	-	17.4	16.48 [14.39 - 18.74]
Saint-Césaire 1	-	-	15.3	7.76 [6.22 - 9.54]

10.3 Mitochondrial genome reconstruction

In order to reconstruct the mitochondrial genomes, we called consensus bases with enough support and a minimum coverage and base quality, while masking transitions at the ends of the sequences to mitigate missteps caused by ancient DNA damage[11]. We explored the robustness to present day human DNA contamination using two different thresholds: i) 80% of sequences supporting a called consensus base, at least 4x coverage, and a minimum base quality of 20; and ii) 66% of the sequences supporting a called consensus base, at least 3x coverage, and a minimum base quality of 20[11].

We compared all of the possible reconstructed consensus sequences by using all sequences versus deaminated-only sequences, with higher and lower contamination thresholds as detailed above, and with stricter and more lenient consensus-calling requirements. Whenever possible, we contrasted our results to the already published mitochondrial consensus genomes[12–14]. The comparisons were performed by first estimating the pairwise-differences among consensus sequences using MEGA version 10.1.7[15] and then using *samtools* to manually inspect the aligned reads at sites with differing calls[16]. All the reconstructed consensus genome sequences stemming from the same specimen and using different criteria were consistent with each other, with no bases being called differently regardless of the filtering criteria selected (Supplementary Data Table 3). The only exception was a discrepancy between the consensus sequence of Spy 94a using deaminated sequences only and a minimum coverage of 3-fold (Supplementary Table 20).

Supplementary Table 20 Pairwise differences between different consensus calls for the Spy 94a mitochondrial genome. For each pair, all ambiguous positions were omitted (pairwise deletion option). A total of 16,604 positions were analysed.

Spy 94a consensus	1	2	3	4	5	6	7	8	9
1 AllSequences MaxCont25 Cov3Support66	-								
2 AllSequences MaxCont25 Cov4Support80	0	-							
3 AllSequences MaxCont10 Cov3Support66	0	0	-						
4 AllSequences MaxCont10 Cov4Support80	0	0	0	-					
**5 DeamSequences_MaxCont25_Cov3Support66**	**5**	**1**	**5**	**1**	-				
6 DeamSequences MaxCont25 Cov4Support80	0	0	0	0	0	-			
**7 DeamSequences_MaxCont10_Cov3Support66**	**6**	**3**	**6**	**3**	**3**	**1**	-		
8 DeamSequences MaxCont10 Cov4Support80	0	0	0	0	0	0	0	-	
9 Published in Hajdinjak et al. 2018	0	0	0	0	**5**	0	**6**	0	-

After visually examining the aligned sequences, we concluded that the differences in the reconstructed consensus sequences likely stem from ancient DNA damage (for an example, see Supplementary Figure 22). All other conflicting bases in Spy 94a were resolved in a comparable manner, and manually annotated in the curated consensus.

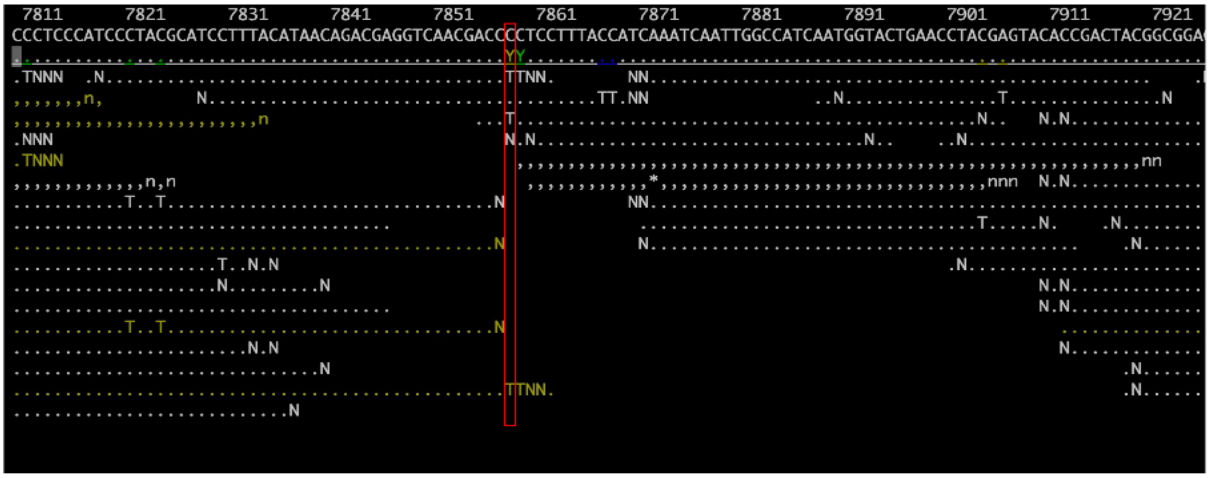

Supplementary Figure 22 An example of a base called inconsistently in different consensus mitochondrial genomes of Spy 94a (in red). Visualising the rCRS alignment with *samtools tview* highlights how the C-to-T substitutions stemming from aDNA damage patterns can interfere with a correct consensus calling.

We examined the consensus support across the mitochondrial genome of Goyet Q54-4 as an additional quality check (Supplementary Figure 23) since its library was captured with an array that also included other mammalian mitochondrial genomes[4]. All but two sites could be recovered with high support.

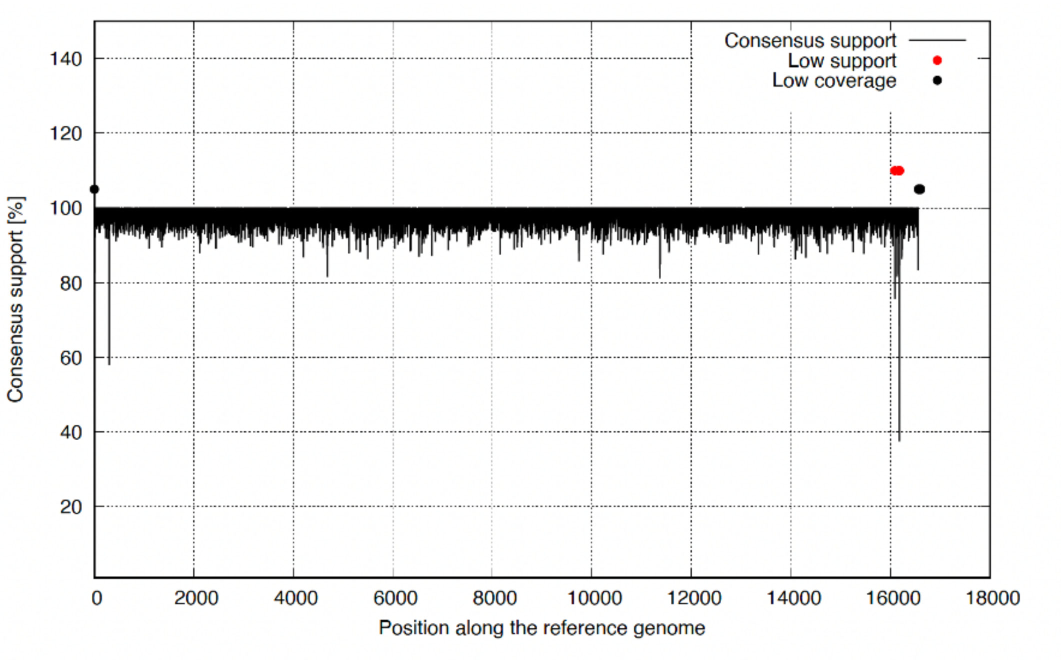

Supplementary Figure 23 Distribution of coverage and a consensus support for the Goyet Q54-4 mitochondrial genome.

We reconstructed 15 new Neandertal mitochondrial genomes, and improved versions of seven Goyet mitochondrial genomes previously reported[12]. For most genomes, the exact quality-control thresholds used did not impact the analysis. Their exact completeness, and comparisons with the published sequences, can be found in Supplementary Table 21.

Supplementary Table 21 Overview of the number of called and uncalled bases in the final consensus sequence for each of the analysed Neandertal specimens.

Sample	Number of called bases	Number of uncalled bases	% coding region called	Previous publications	Previously called bases	Previously uncalled bases
AR-14	15,746	819	94.5	new	-	-
AR-30	16,527	38	99.7	new	-	-
Goyet Q305-7	16,565	0	100.0	Rougier et al. 2016	16,561	2
Goyet Q56-1	16,565	0	100.0	Hajdinjak et al. 2018	16,565	0
Goyet Q374a-1	16,565	0	100.0	Rougier et al. 2016	16,562	3
Spy 94a	16,565	0	100.0	Hajdinjak et al. 2018	16,565	0
Spy 8	16,551	14	99.9	new	-	-
Spy 16	6,533	10,032	37.3	new	-	-
Spy 572a	676	15,926	4.35	new	-	-
Goyet Q54-4	16,562	4	100.0	new	-	-
Goyet C5-1	16,543	22	100.0	new	-	-
Goyet Q376-9	16,411	154	98.7	new	-	-
Goyet Q57-1	16,562	3	100.0	Rougier et al. 2016	16,257	309
Goyet Q57-2	16,564	1	100.0	Rougier et al. 2016	16,564	2
Goyet Q57-3	16,564	1	100.0	Rougier et al. 2016	16,538	27
Goyet 1424-3D	16,215	350	98.8	new	-	-
Goyet Q376-25	16,470	95	99.4	new	-	-
Fonds-de-Forêt 1	16,564	1	100.0	Devièse et al. 2021	16,564	1
Goyet Q55-4	16,554	11	100.0	new	-	-
Goyet Q305-4	16,565	0	100.0	Rougier et al. 2016	16,565	0
Goyet Q119-2	16,542	23	99.9	new	-	-

Sample	Number of called bases	Number of uncalled bases	% coding region called	Previous publications	Previously called bases	Previously uncalled bases
Walou	16,063	502	97.8	new	-	-
Couvin	16,125	440	97.2	new	-	-
Saint-Césaire 1	16,445	120	99.6	new	-	-
Les Cottés Z4-1514	16,565	0	100.0	Hajdinjak et al. 2018	16,565	0

10.4 Heteroplasmies

Unresolved bases in the mtDNA consensus sequences may be the result of heteroplasmies, i. e., sites where the mitochondrial genomes of an individual are polymorphic. Because heteroplasmies are usually short-lived, shared heteroplasmies may pinpoint towards closely-related individuals[17,18]. We visually examined all data for possible heteroplasmies, focusing primarily on sites that had sufficient coverage for calling the consensus, and at the same time, insufficient support.

The results are summarised in Supplementary Table 22. Out of 29 candidate positions for shared heteroplasmies between Goyet specimens, only one could not be explained by either ancient DNA damage, present-day contamination, or a combination of both.

Supplementary Table 22 Candidate positions for shared heteroplasmies between Goyet specimens. We evaluated the data using the allele in rCRS as a proxy for contamination, and the orientation of the reads.

Position (Vindija 33.16)	Shared between…	V1/V2	Variant in rCRS	Are the transitions mostly in one read orientation?	Conclusion
146	Goyet 1424-3D, Goyet Q119-2, Goyet 376-25, Goyet Q376-9, Goyet Q55-4, Goyet Q57-1	T/C	T	-	Contamination
150	Goyet 1424-3D, Goyet Q119-2, Goyet 376-25, Goyet Q376-9, Goyet Q55-4, Goyet Q57-1	T/C	C	-	Contamination
152	Goyet 1424-3D, Goyet C5-1, Goyet Q119-2, Goyet Q305-4, Goyet Q376-25, Goyet Q376-9, Goyet Q55-4, Goyet Q57-1	C/T	T	No	Contamination

189	Goyet C5-1, Goyet Q119-2, Goyet Q376-25, Goyet Q376-9, Goyet Q55-4	G/A	A	Yes	Contamination and damage
200	Goyet Q119-2, Goyet Q55-4	G/A	A	Yes	Contamination and damage
243	Goyet C5-1, Goyet Q55-4	G/A	A	Yes	Contamination and damage
245	Goyet C5-1, Goyet Q376-9	C/T	T	Yes	Contamination and damage
310	Goyet 1424-3D, Goyet C5-1, Goyet Q119-2, Goyet Q305-4, Goyet Q376-25, Goyet Q376-9, Goyet Q55-4, Goyet Q57-1, Goyet Q57-3	C/T/-	T	Yes	Poli-C stretch, unresolved
3,079	**Goyet C5-1, Goyet Q119-2**	**T/C**	**T**	**-**	**Unclear**
4,935	Goyet Q376-25, Goyet Q376-9	T/C	C	-	Contamination
8,450	Goyet Q376-25,Goyet 1424-3D	T/C	C	-	Contamination
8,463	Goyet Q376-25, Goyet Q376-9	T/C	C	-	Contamination
10,319	Goyet C5-1, Goyet Q376-9	C/T	T	Yes	Contamination and damage
10,368	Goyet Q376-25, Goyet Q376-9	A/G	G	-	Contamination
10,960	Goyet Q376-25, Goyet Q376-9	C/T	C	Yes	Damage
11,359	Goyet 1424-3D, Goyet Q376-9	C/T	C	Yes	Damage
14,048	Goyet Q376-25, Goyet Q376-9	G/A	A	No	Contamination

15,624	Goyet1424-3D, Goyet Q55-4	C/T	T	Yes	Damage
15,662	Goyet Q376-25, Goyet Q376-9	T/C	C	-	Contamination
15,666	Goyet Q376-25, Goyet Q376-9	G/A	C	Yes	Damage
16,088	Goyet C5-1, Goyet Q376-25, Goyet Q376-9, Goyet Q54-4	C/T	T	No	Contamination
16,124	Goyet C5-1, Goyet Q376-25	A/G	G	-	Contamination
16,134	Goyet C5-1, Goyet Q376-25	T/A	A	-	Contamination
16,135	Goyet C5-1, Goyet Q376-25	A/T	T	-	Contamination
16,143	Goyet C5-1, Goyet Q376-25	T/C	C	-	Contamination
16,177	Goyet 1424-3D, Goyet C5-1, Goyet Q119-2, Goyet Q305-4, Goyet Q305-7, Goyet Q374a-1, Goyet Q376-25, Goyet Q376-9, Goyet Q54-4, Goyet Q57-1, Goyet Q57-2, Goyet Q57-3	A/C/T/-	A	-	Poli-C stretch, unresolved
16,178	Goyet C5-1, Goyet Q54-4	A/C/T/-	A	-	Poli-C stretch, unresolved
16,307	Goyet Q119-2, Goyet Q376-25, Goyet Q55-4	C/T	T	No	Contamination and damage
16,316	Goyet C5-1, Goyet Q119-2, Goyet Q376-25, Goyet Q376-9, Goyet Q55-4	T/C	C	-	Contamination

The most complex is the position 3,079 in the Vindija 33.16 genome coordinate system. At this site, we find sequences with both “T” and “C” alleles in libraries from Goyet C5-1 and Goyet Q119-2, which could originate from cytosine deamination. Similarly, since the rCRS has a “T” in this position, this allele might result from human DNA contamination. However, because the “T”-alleles in the examined

libraries are always the most abundant allele (with a frequency of at least 70%), and are supported in roughly equal amounts by sequences in both orientations (i.e. the allele is not only present in a forward orientation), we cannot assign this polymorphism to contamination or deamination as in previous cases. At the same time, one of the quality checks applied previously to verify ancient heteroplasmies, i.e., that the polymorphism frequencies should be roughly the same before and after filtering for deaminated sequences[18], does not hold true for Goyet Q119-2 (Supplementary Table 23). Since the reconstructed mitochondrial genomes of Goyet C5-1 and Goyet Q119-2 have eight differences, this is unlikely to be a shared heteroplasmy (Supplementary Figure 25).

Supplementary Table 23 Coverage and consensus information for the heteroplasmy position candidate that could not be explained by contamination or ancient DNA damage (Deam = deaminated).

Position (Vindija 33.16)	Sample	Sequences	Depth of coverage at that position	% of alternative allele (C)
3,079	Goyet C5-1	all	150 x	25.00
		Deam-only	81 x	23.45
	Goyet Q119-2	all	151 x	5.29
		Deam-only	43 x	32.55

Therefore, we conclude that no shared heteroplasmies could be confidently identified between the analysed genomes.

10.5 Mitochondrial phylogeny

We next placed the reconstructed consensus mtDNA sequences in the broader context of Neandertal mitochondrial variation. Taking into consideration the kinship results described in Supplementary Section 14, we excluded redundant mitochondrial genomes recovered from different bones that we found to have originated from the same individual. In those cases, we used the most complete mitochondrial genome for downstream analyses. For the GN1 individual we used the mtDNA genome recovered from the Goyet Q56-1 (and omitted Goyet Q374a-1 and Goyet Q305-7), for the GN2 individual we used the mtDNA genome from the Goyet Q57-2 (and omitted Goyet Q57-1, Goyet Q57-3 and Goyet Q54-4), and for Spy 94a_8 we used the mtDNA genome from Spy 94a (and omitted Spy 8).

We then aligned our set of 17 reconstructed Neandertal mitochondrial genomes to a mitochondrial dataset of 55 present-day modern humans[3,19], ten ancient modern humans[20–22,1,23,11,24] and 23 Neandertals[6,25,9,26,27,12,28,13,29,14,30,18,31,32]. We added the mitochondrial genome from the Middle Pleistocene hominin from Sima de los Huesos[11] and four Denisovans[33–36] as outgroups. If multiple archaics had identical mtDNA sequences, we only included one in the reconstructed tree.

The alignment was performed with *mafft* with 1,000 iterations (version 7.453)[37], defining the coordinates with rCRS. We built a Maximum Likelihood (ML) tree using 100 bootstrap iterations using the software MEGA[38]. The ML tree is represented in Supplementary Figure 24.

All the newly reconstructed mitochondrial genomes from Goyet fall within the previously described 'haplogroups'[12], which is also reflected in their number of pairwise differences (Supplementary Figure 25). The Saint-Césaire 1 mitochondrial genome falls close to the mtDNA genomes of the Spy, Goyet and Broion Neandertals. In contrast, the Couvin mitochondrial genome falls within the Stajnia S5000[39] and Mezmaiskaya 1 clade[25], together with the recently published mtDNA genome of the Thorin Neandertal[32]. Even though the Couvin specimen was unearthed from a layer dated with AMS to 44,500 +1,100/-800 years 14C BP (GrA-40444)[40] or 49,795 - 44,955 years cal BP with 95.4% probability, calibrated with IntCal20 using Oxcal 4.4.4[41,42], it clusters with far older Neandertal individuals with dates of over 80,000 years BP[39].

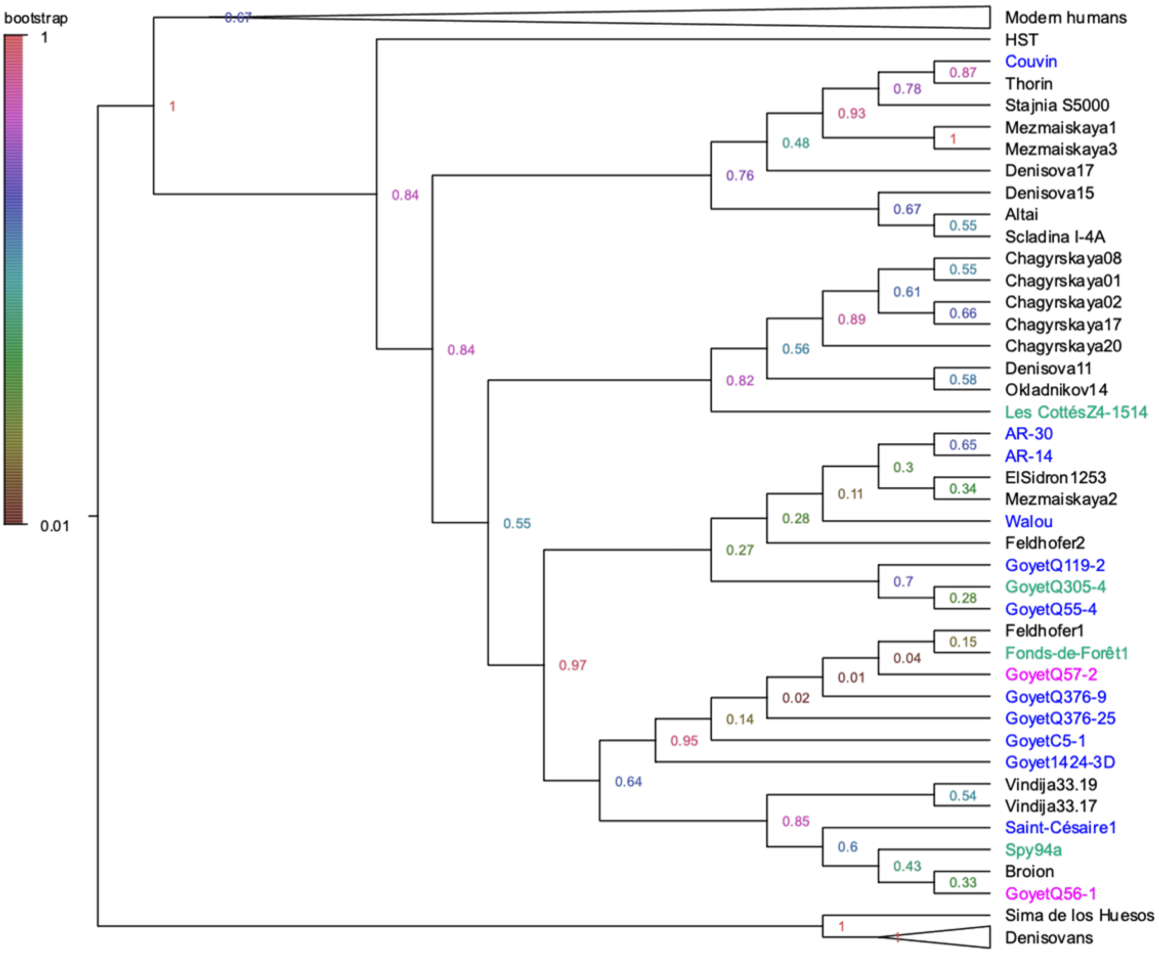

Supplementary Figure 24 Maximum likelihood tree of the mitochondrial genomes. New consensus sequences are coloured in dark blue, improved versions of previously published sequences appear in purple, and unmodified published sequences that are of special relevance for this study are highlighted in turquoise. The values in the nodes indicate the bootstrap support for that branching (100 replicates). A total of 14,739 positions were used to calculate the tree using MEGA.

		1	2	3	4	5	6	7	8	9	10	11	12	13	14	15	16	17	18	19	20	21	22
1	**rCRS_NC_012920**																						
2	**AR-14**	72																					
3	**AR-30**	193	0																				
4	**Goyet Q305-7**	208	9	13																			
5	**Goyet Q56-1**	208	9	13	0																		
6	**Goyet Q374a-1**	208	9	13	0	0																	
7	**Spy 94a**	207	8	12	1	1	1																
8	**Spy 8**	200	8	12	1	1	1	0															
9	**Goyet C5-1**	188	6	9	11	11	11	10	10														
10	**Goyet Q376-9**	176	6	9	12	12	12	11	11	0													
11	**Goyet Q57-1**	203	7	10	13	13	13	12	12	0	0												
12	**Goyet Q57-2**	205	8	11	14	14	14	13	13	0	0	0											
13	**Goyet Q57-3**	205	8	11	14	14	14	13	13	0	0	0	0										
14	**Goyet Q54-4**	203	7	10	13	13	13	12	12	0	0	0	0	0									
15	**Goyet 1424-3D**	177	7	8	11	11	11	10	10	0	0	0	0	0	0								
16	**Goyet Q376-25**	171	6	9	11	11	11	10	10	0	0	0	0	0	0	0							
17	**Fonds-de-Forêt 1**	205	8	11	14	14	14	13	13	1	2	1	2	2	2	1	1						
18	**Goyet Q55-4**	191	2	2	13	13	13	12	12	8	8	9	10	10	9	8	8	10					
19	**Goyet Q305-4**	201	2	2	13	13	13	12	12	9	9	10	11	11	10	8	9	11	0				
20	**Goyet Q119-2**	190	2	2	13	13	13	12	12	8	8	9	10	10	9	8	8	10	0	0			
21	**Couvin**	124	15	27	37	37	37	36	34	29	31	35	36	36	35	29	30	36	29	30	28		
22	**Saint-Césaire 1**	135	6	11	3	3	3	2	2	9	10	11	12	12	11	9	9	12	11	11	11	27	
23	**Les Cottés Z4-1514**	210	26	39	46	46	46	45	45	39	40	42	43	43	42	36	39	43	37	39	37	42	39

Supplementary Figure 25 Pairwise differences of the reconstructed consensus sequences. The three mitochondrial groups of Goyet Neandertals first described in Rougier et al., 2016 are highlighted in red, blue and green, respectively. Other pairs of identical genomes are highlighted in grey and yellow.

To verify the result for the mitochondrial genomes with more than 500 uncalled bases (i.e., AR-14, Spy 16, Spy 572a, and Walou), as well as to try to place the samples for which it was not possible to reconstruct a complete consensus sequence, we explored their genetic affinities using Kallisto[43]. This strategy has previously been employed for sedimentary DNA samples[44], which are typically of lower coverage, to evaluate with which set of known reference samples they match the most. For this analysis, we used the same multiple sequence alignment as described above. One caveat of this approach are technical errors when the set of references contain identical sequences, with the number of potential matches evenly split between identical sequences. Therefore, we removed all identical sequences from the reference set of Kallisto, including Feldhofer 1. This mitochondrial genome has just one difference to Goyet Q57-2, which was causing the same technical error as identical sequences, since they are virtually indistinguishable for the method. The selected reference sequences were split on k-mers of 21 base pairs as described priorly[44].

The Kallisto results were in accordance with the reconstructed ML phylogenies (Supplementary Figure 26). Both Walou and AR-14 best match the mtDNA genome of Feldhofer 2. Similarly, the best Neandertal match for Spy 16 is Spy 94a, another Neandertal from the same archaeological site. For the Spy 572a the results remain inconclusive. We detected present day human contamination in all samples using Kallisto, as supported by the high estimated counts on the modern human branch.

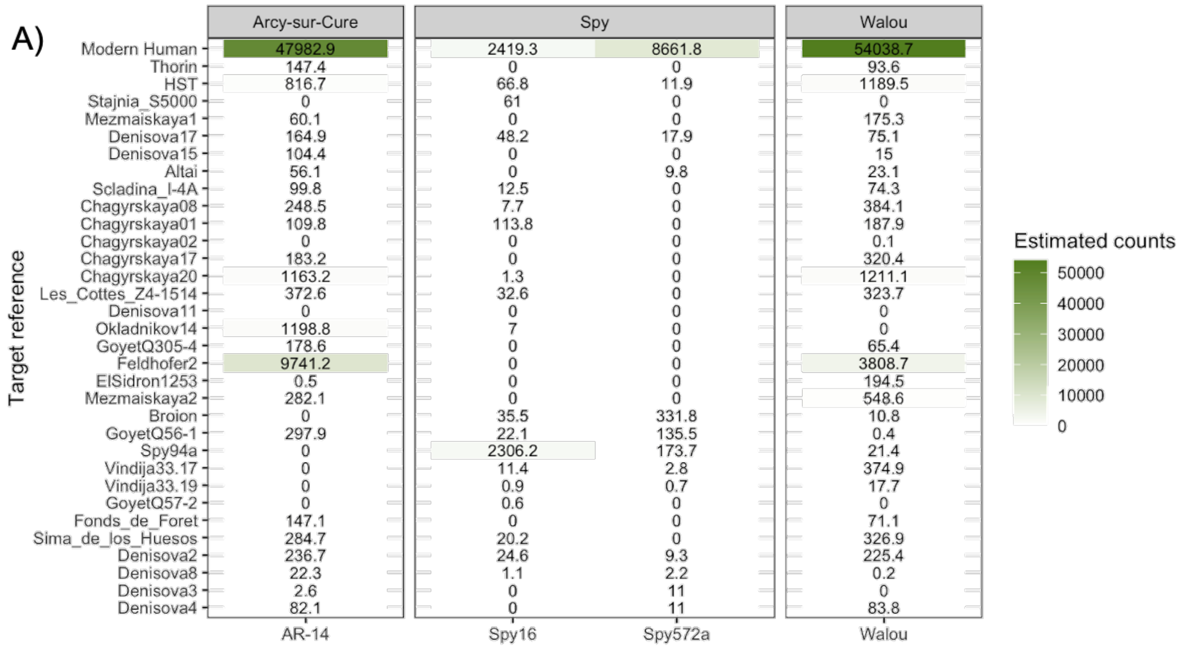

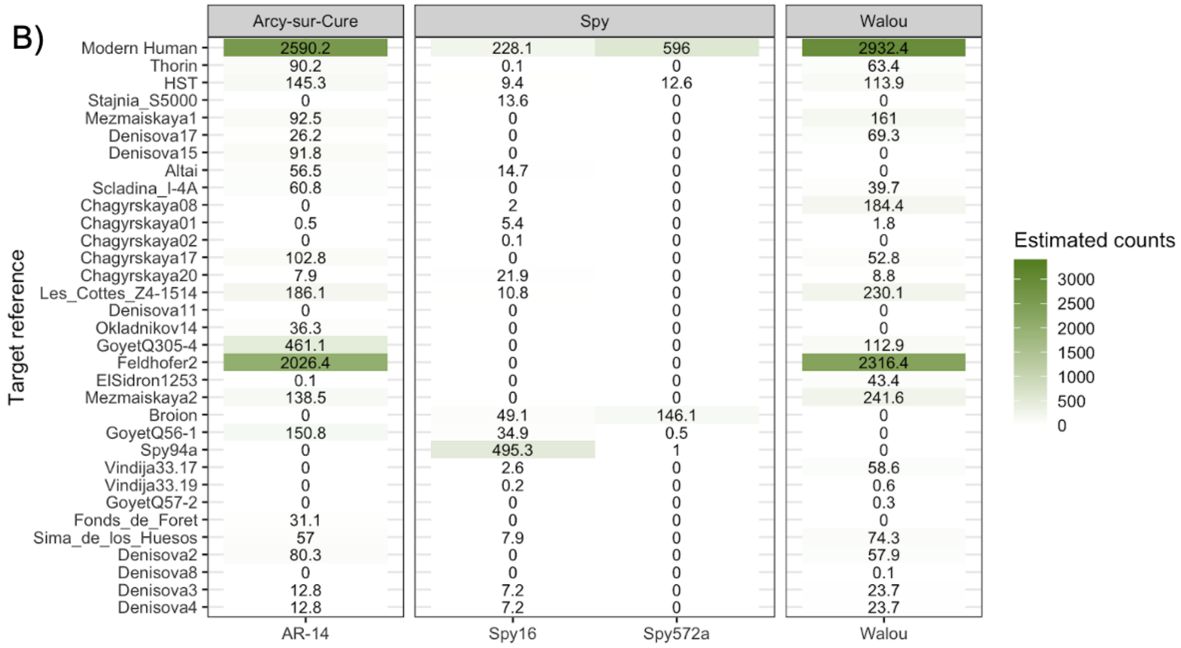

Supplementary Figure 26 Kallisto mitochondrial DNA abundances for AR-14, Spy 572a, Spy 572a and Walou relative to a reference of hominin mitochondrial genomes, using A) all sequences and B) deaminated sequences only.

10.6 Bayesian mitochondrial phylogeny

We performed a Bayesian phylogenetic analysis of the reconstructed mtDNA genomes using BEAST2 v.2.6.1[45], which allowed us to estimate the genetic dates of analysed genomes.

We prepared our input files using seaview v5.0[46] and beauti2[45]. The dataset for this analysis resembled the one described in Section 10.5 for the maximum likelihood phylogeny. In BEAST2, we repeated our analyses twice: once by making use of the complete mitochondrial genome and a mutation rate of 2.53×10^{-8} substitutions per site per year[47], and once using only the region that falls outside of the hypervariable region (from 577 bp to 16,023 bp[23] and a mutation rate of 1.57×10^{-8} substitutions per site per year.

The dates used as BEAST2 priors are indicated in Supplementary Tables 24, 25 and 26, with their respective references. All radiocarbon dates were calibrated with the IntCal20 curve, using OxCal 4.4.4, rounded to the nearest 10 years[41,42]. A few instances required a more thorough examination. Firstly, some specimens that we identified as genetically identical (see Supplementary Section 14), namely Goyet Q57-1, Goyet Q57-2 and Goyet Q57-3 comprising individual GN2, had non-overlapping radiocarbon dates (both before and after calibration).

We tested the congruence of their radiocarbon dates using a Chi-squared test, as described[48], with a significance threshold of 5%. The three dates failed to pass the test, thus confirming that they could not be combined using the OxCal "R Combine function", which is the common procedure for multiple measurements from the same individual (Supplementary Figure 27).

Name	Unmodelled (BP)	
	from_95.4	to_95.4
R_Date GrA-46173	44876	43161
R_Date GrA-60019	42663	42089
R_Date GrA-54024	41996	41109
R_Combine GoyetQ57-combined	42668	42271
	Warning! X-Test fails at 5% - GoyetQ57-combined X2-Test: df=2 T=73.695(5% 6.0)	

Supplementary Figure 27 OxCal 4.4.4 R-combine output, indicating that the three radiocarbon dates from Goyet Q57-1, Goyet Q57-2 and Goyet Q57-3 could not be combined.

Considering that two of these bone fragments belong to the left and right tibias of the same individual – Goyet Q57-1 and Goyet Q57-3, respectively – we can rule out to a certain extent differences caused by dietary factors[49,50] or intra-skeletal variation[51]. However, this does not apply to Goyet Q57-2, which is a femur.

The most parsimonious explanation is the presence of residual contamination from present-day carbon. Samples from this time period, at the edge of the radiocarbon dating limits, are especially sensitive to small traces of present-day carbon[52]. Even though the C/N atomic ratios obtained from these samples fell within the acceptable range of variation[12,53], it is still possible that small traces of undetected contamination could greatly bias the final radiocarbon results. Since these three fragments were all isolated from the unidentified fauna collection that did not undergo consolidating treatment – unlike the hominin remains – contamination originating from animal varnish is unlikely. However, the samples could have been contaminated in the laboratory between the stable isotopic and the radiocarbon measurements.

Thus, we considered the oldest date to be the most representative. Since we cannot be certain which of the dates is the most accurate, we also explored the effects of using the youngest dates. Thus, we ran BEAST2 twice: once with the oldest calibrated date as defined by Goyet Q57-1 (with a 95.4 % range of 44,880 – 43,160 years cal BP), and once with the youngest calibrated date as defined by Goyet Q57-2 (with a 95.4 % range of 42,000 – 41,100 years cal BP).

The second pair of instances that required a more thorough examination concerned the priors of Couvin and Thorin – a Neandertal from Mandrin, France[32]. Both of these Neandertals stem from an archaeological context supporting their classification as Late Neandertals, but represent an early-splitting branch of the Neandertal mitochondrial tree (as seen in Section 10.5). The Couvin molar was found at the base of Layer II of Trou de l'Abîme. Its relative dating is based on the AMS radiocarbon ages of 44,500 +1,100/−800 BP (GrA-40444), 43,600 ± 1,900 (OxA-34120) and 43,400 ± 1,800 (OxA-34121); respectively calibrated to 47,200 cal BP (95.4% probability range: 49,800–44,950 cal BP), 47,300 cal BP (95.4 % probability range: 52,480–43,160 cal BP), and 47,000 cal BP(95.4% probability range: 51,950–43,180 cal BP); all obtained from faunal remains recovered from the same layer[40]. Unlike the case of Goyet Q57-2 described above, statistically we found no evidence of significant differences between the dates of the faunal remains (R_combine X2-Test: df=2, T=0.3 (5% 6.0); Combine X2-Test: df=2 T=0.339(5% 5.991)), and therefore we decided to combine these dates so that the Couvin prior was 46,670 cal BP(95.4% probability range: 49,660–44,170 cal BP). As for the Thorin Neandertal, he has been dated by Uranium series of one of his premolars with a minimum age of 43,500 ± 4,100 years BP, as well as by radiocarbon dating of the hydroxyproline (HYP) of three hominin fragments identified through Zooarchaeology by Mass Spectrometry, with a combined age of 53,000 - 48,000 years cal BP with a 95.4% confidence interval[32].

Since Slimak et al.[32] reported that the genetic dates of Thorin can vary by up to ~50 ky based on whether the HYP radiocarbon dates were used as priors or not, we tested different scenarios. We ran BEAST2 once by following the Slimak et al. approach, with a prior for Thorin ranging from 45,000 to 50,000 years BP, or with a flat prior of 30,000 to 200,000 years BP. Similarly, we also estimated the genetic

dates of Couvin by using their relative radiocarbon dates as priors ranging from 49,660 to 44,170 years calBP, as well as by specifying the same flat prior of 30,000 to 200,000 years BP. We grouped our BEAST2 analyses in three groups: without any priors for either Thorin or Couvin being constrained by the archaeological data, with radiocarbon dates as priors only for Couvin, and with radiocarbon dates as priors only for Thorin.

Regarding the rest of the samples in our analyses, in the cases where no radiocarbon dates were available, we either based our priors on previous genetic dating, or used a flat prior of 30,000 to 200,000 years BP. Additionally, all present-day humans were set to an age of 0 years BP. In the case of "group priors" (e.g., all Neandertals), we explicitly required BEAST2 to consider such groups as monophyletic.

We used jModelTest2 (version 2.11), which takes into consideration the Bayesian information criterion (BIC), the decision theory measure (DT) and the corrected Akaike information criterion (AICc), to find the best substitution model (Supplementary Table 27). The results indicated that the best substitution model explaining our data was TrN[54], with a proportion of invariant sites of 0.8. We specified these parameters in BEAST2, allowing for the re-estimate of the proportion of invariable sites.

Supplementary Table 24 Priors for the tip dates analysed in BEAST2 based on radiocarbon dates, rounded to a precision of 10 years. Samples of special interest appear at the end of the table, highlighted in bold letters. All present-day human genomes were set with a prior age of 0 years BP. The special cases described in the supplementary chapter are highlighted in grey.

Group	Individual	Radiocarbon date ID	Raw radio-carbon date	Publication of the date	Initial tip date (prior)	Calibrated Date OxCal 4.4.4 IntCal20 in BP 95.4 % lower	Calibrated Date OxCal 4.4.4 IntCal20 in BP 95.4 % higher
**Modern human**	Ust'-Ishim 1	OxA-25516; OxA-30190	41,400 ± 1,300; 41,400 ± 1,400	Fu et al., 2014	44,370	45,930	42,900
	Boshan 11	MAMS-13530	7,368 ± 34	Fu et al., 2013	8,170	8,320	8,030
	Loschbour	OxA-7338	7,205 ± 50	Higham et al., 2007; Toussaint et al., 2010; Delsate et al., 2011	8,050	8,170	7,930

Group	Individual	Radiocarbon date ID	Raw radio-carbon date	Publication of the date	Initial tip date (prior)	Calibrated Date OxCal 4.4.4 IntCal20 in BP 95.4 % lower	Calibrated Date OxCal 4.4.4 IntCal20 in BP 95.4 % higher
	Tianyuan 1	BA-03222	34,430 ± 510	Fu et al., 2013	39,460	40,850	38,070
	Kostenki 14	OxA-X-2395-15 (corrected by HPLC background)	33,900 ± 550	Marom et al., 2012	38,770	40,240	37,210
	Eskimo Saqqaq	OxA-20656	4,044 ± 31	Rasmussen et al., 2010	4,600	4,790	4,410
	Oberkassel 998	OxA-4790	11,570 ± 100	Fu et al., 2013	13,450	13,730	13,180
	Iceman	OxA-37371; OxA-37372; OxA-37373; OxA-37374; OxA-37375; OxA-37376; OxA-3419; OxA-3420; OxA-3421	4,660 ± 55; 4,565 ± 60; 4,550 ± 70; 4,660 ± 80; 4,530 ± 70; 4,450 ± 80; 4,540 ± 55; 4,530 ± 70; 4,480 ± 55	Bonani et al., 1992, 1994; Hedges et al., 1992	5,180	5,320	5,050
	Dolní Věstonice 14	Aix-12028	26,760 ± 100	Fewlass et al., 2019	31,000	31,150	30,840
	Dolní Věstonice 13	Aix-12027	27,040 ± 100	Fewlass et al., 2019	31,140	31,250	31,030
**Neandertal**	Feldhofer 1	ETH-20981	39,900 ± 620	Schmitz et al., 2002	43,410	44,290	42,530
	Feldhofer 2	ETH-19660	39,240 ± 670	Schmitz et al., 2002	43,140	44,040	42,250
	Vindija 33.19	OxA-X-2717-11	44,300 ± 1,200	Devièse et al., 2017	47,310	49,930	44,690
	El Sidrón 1253	OxA-21776	48,400 ± 3,200	Wood et al., 2012	53,480	60,000	46,970
	Mezmaiskaya 2	OxA-21839	39,700 ± 1,100	Pinhasi et al., 2011	43,560	44,970	42,150
	**AR-14**	**MAMS-25149**	**36,840 ±660**	**Welker et al., 2016**	**41,570**	**42,370**	**40,770**

Group	Individual	Radiocarbon date ID	Raw radio-carbon date	Publication of the date	Initial tip date (prior)	Calibrated Date OxCal 4.4.4 IntCal20 in BP 95.4 % lower	Calibrated Date OxCal 4.4.4 IntCal20 in BP 95.4 % higher
**Neandertal**	**Goyet Q305-4**	**GrA-46176**	**40,690 +480, −400**	**Rougier et al., 2016**	**43,730**	**44,490**	**42,980**
	**Goyet Q56-1**	**GrA-46170**	**38,440 +340, −300**	**Rougier et al., 2016**	**42,450**	**42,740**	**42,160**
	**Goyet Q57-1**	**GrA-46173**	**41,200 +500, −410**	**Rougier et al., 2016**	**44,020**	**44,880**	**43,160**
	**Goyet Q57-2**	**GrA-54024**	**36,590 +300, −270**	**Rougier et al., 2016**	**41,550**	**42,000**	**41,100**
	**Goyet Q57-3**	**GrA-60019**	**38,260 +350, −310**	**Rougier et al., 2016**	**42,370**	**42,670**	**42,080**
	**Les Cottés Z4-1514**	**MAMS-26196**	**39,485 ± 271**	**Hajdinjak et al., 2018**	**42,840**	**43,150**	**42,540**
	**Spy 94a**	**OxA-X-2762-21**	**41,500 ± 1,800**	**Devièse et al., 2021**	**46,030**	**49,740**	**42,330**
	**Fonds-de-Forêt 1**	**OxA-38322**	**39,500 ± 1,100**	**Devièse et al., 2021**	**43,460**	**44,860**	**42,070**
	**Couvin**	**GrA-40444; OxA-34120; OxA-34121**	**44,500 ± 1,100; 43,600 ± 1,900; 43,400 ± 1,800**	**Toussaint et al., 2010; Abrams et al., 2024; Devièse et al., 2021**	**46,670**	**49,660**	**44,170**
	**Thorin**	**OxA-37787; OxA-38388; OxA-38389**	**45,800 ± 2,100; 45,100 ± 0; 45,400 ± 0**	**Slimak et al., 2024**	**48,000**	**45,000**	**50,000**

Supplementary Table 25 Priors for the tip dates analysed in BEAST2 based on archaeological or genetic lines of evidence. Samples of special interest appear at the end of the table, highlighted in bold letters. All present-day human genomes were set with a prior age of 0 years BP. The special cases described in the supplementary chapter are highlighted in grey.

Group	Individual	Archaeological or genetic evidence informing the prior	Initial tip date (prior)	Lower prior bound	Upper prior bound
**Denisovan**	Denisova 2	No radiocarbon date available, used flat prior	50,000	300,000	30,000
	Denisova 4		50,000	300,000	30,000
	Denisova 8		50,000	300,000	30,000
	Denisova 3	No radiocarbon date available, used prior based on Prüfer et al., 2017	50,000	100,000	30,000
**Neandertal**	Mezmaiskaya 1	No radiocarbon date available, used prior based on Hajdinjak et al., 2018	60,000	200,000	30,000
	Vindija 33.17	No radiocarbon date available, used flat prior	50,000	200,000	30,000
	Mezmaiskaya 3		50,000	200,000	30,000
	Okladnikov 14		50,000	200,000	30,000
	HST		50,000	200,000	30,000
	Denisova 11		50,000	200,000	30,000
	Broion		50,000	200,000	30,000
	Denisova 15		50,000	200,000	30,000
	Stajnia S5000		50,000	200,000	30,000
	Saint Césaire 1	Available radiocarbon date (OxA-18099) had a collagen yield < 1% and %C = 27	50,000	200,000	30,000
	Chagyrskaya 01	No radiocarbon date available, used prior based on Skov et al.,	70,000	120,000	40,000

Group	Individual	Archaeological or genetic evidence informing the prior	Initial tip date (prior)	Lower prior bound	Upper prior bound
**Neandertal**	Chagyrskaya 02	2022	70,000	120,000	40,000
	Chagyrskaya 08		70,000	120,000	40,000
	Chagyrskaya 17		70,000	120,000	40,000
	Chagyrskaya 20		70,000	120,000	40,000
**Neandertal**	Denisova 17	No radiocarbon date available, used prior based on Brown et al., 2022	100,000	200,000	30,000
	Scladina I-4A	No radiocarbon date available, used prior based on Peyrégne et al., 2019	100,000	200,000	30,000
	Altai	No radiocarbon date available, used prior based on Prüfer et al., 2014	120,000	200,000	30,000
	Thorin	Radiocarbon dates available, but used flat prior for exploratory analyses	50,000	200,000	30,000
	**Couvin**		**50,000**	**200,000**	**30,000**
	**Walou**	**No radiocarbon date available, used flat prior**	**50,000**	**200,000**	**30,000**
	**Goyet 1424-3D**		**50,000**	**200,000**	**30,000**
	**Goyet C5-1**		**50,000**	**200,000**	**30,000**
	**Goyet Q119-2**		**50,000**	**200,000**	**30,000**
	**Goyet Q54-4**		**50,000**	**200,000**	**30,000**
	**Goyet Q55-4**		**50,000**	**200,000**	**30,000**
	**Goyet Q376-25**		**50,000**	**200,000**	**30,000**

Group	Individual	Archaeological or genetic evidence informing the prior	Initial tip date (prior)	Lower prior bound	Upper prior bound
	**Goyet Q376-9**		**50,000**	**200,000**	**30,000**
	**AR-30**		**50,000**	**200,000**	**30,000**
**Sima de los Huesos**	**SH Femur XIII**	No radiocarbon date available, used prior based on Arsuaga et al., 2014	300,000	780,000	260,000

Supplementary Table 26 Priors for the node ages of monophyletic groups indicated in BEAST2.

Group	Genomes included in each group	Lower limit date in BP	Upper limit data in BP
Denisovans (no Sima)	Denisova 2, Denisova 3, Denisova 4, Denisova 8	Inf	100,000
Denisovans (with Sima)	Denisova 2, Denisova 3, Denisova 4, Denisova 8, Sima de los Huesos	Inf	400,000
Neandertals	Altai, AR-14, AR-30, Broion, Thorin, Chagyrskaya 01, Chagyrskaya 02, Chagyrskaya 08, Chagyrskaya 17, Chagyrskaya 20, Couvin, Denisova 11, Denisova 15, Denisova 17, El Sidrón 1253, Feldhofer 1, Feldhofer 2, Fonds-de-Forêt 1, Goyet 1424-3D, Goyet C5-1, Goyet Q119-2, Goyet Q305-4, Goyet Q376-25, Goyet Q376-9, Goyet Q55-4, Goyet Q56-1, Goyet Q57-2, HST, Les Cottés Z4-1514, Mezmaiskaya 1, Mezmaiskaya 2, Mezmaiskaya 3, Okladnikov 14, Saint-Césaire 1, Scladina I-4A, Spy 8, Spy 94a, Stajnia S5000, Vindija 33.17, Vindija 33.19, Walou	Inf	200,000
Modern humans (MH)	AF346966, AF346973, AF346975, AF346977, AF346981, AF346990, AF346995, AF346999, AF347008, AF347010, AF347014, AF347015, AF381981, AF381984, AF381988, AF381997, AF381998, AF381999, AF382000, AY195748, AY195754, AY195756, AY195757, AY195759, AY195766, AY195773, AY195774, AY195787, AY289059, AY289082, AY289085, AY289094, AY289097, AY289101, AY882380, AY882382, AY882386, AY882388, AY882389, AY882390, AY882391, AY882392, AY882393, AY882403, AY882412, AY882416, AY950293, AY950300, AY963572, AY963573, AY963586, Boshan 11, Dolní Věstonice 13, Dolní Věstonice 14, DQ137410, DQ137411, Eskimo Saqqaq, Iceman, Kostenki 14, Loschbour, Oberkassel 998, rCRS_NC_012920, Tianyuan 1, Ust'-Ishim 1	Inf	100,000
MH + Neandertals	All Neandertals + All Modern humans (see two previous rows)	Inf	200,000
All Hominins	All samples	Inf	600,000

Supplementary Table 27 Comparison of substitution models using jModelTest2, for both the complete and the coding portion of the mitochondrial genome. Abbreviations: Bayesian information criterion (BIC), corrected Akaike information criterion (AICc) and decision theory (DT).

Genome	Measure	Model	Log-likelihood	Proportion invariable sites
**Complete mitochondria**	BIC	TrN+I+G	-35,528.92	0.81
	AICc	GTR+I+G	-35,519.09	0.82
	DT	TrN+I+G	-35,528.92	0.81
**Coding region of the mitochondria**	BIC	TrN+I+G	-30,441.36	0.69
	AICc	TrN+I+G	-30,441.36	0.69
	DT	TrN+I+G	-30,441.36	0.69

For each BEAST2 analysis, we tested four different models with different clock and tree models. We compared (1) a strict clock and constant population size, (2) a "relaxed" clock (i.e., an uncorrelated normal clock with freedom for each branch) and constant population size, (3) a strict clock and a Bayesian Skyline population model[55], and (4) a relaxed clock and a Bayesian Skyline population model.

For each model, we used a chain length of 75,000,000, with a burn-in of 10,000,000, storing every 5,000 steps. The subsequent model selection was performed using a path sampling approach[45] of 40 path steps. We used BIC to compare the different likelihoods of each model, and found that the best models always favoured a Bayesian Skyline model, with a consistent preference for strict mutational clocks (Supplementary Data Table 6; Supplementary Tables 26 - 29), both for the complete mitochondrial sequence and the coding regions-only. We also tested a relaxed mutational clock, despite it not being the best-supported model, to align our comparisons as closely as possible with the analyses described in Slimak et al.[32].

Supplementary Table 28 Marginal likelihoods resulting from the path sampling of different clock and tree models, for both the complete and the coding portion of the mitochondrial genome, using the oldest dates of Goyet Q57-2 as prior, and with flat priors for Thorin and Couvin. The best model is highlighted in blue.

Genome	Selected clock	Selected tree	Marginal log-likelihood for that model
**Complete mitochondria**	Strict	Coalescent Constant size	-36,421.74
	Uncorrelated normal	Coalescent Constant size	-36,409.30
	Strict	Bayesian Skyline	-36,371.63
	Uncorrelated normal	Bayesian Skyline	-36,376.69
**Coding Region of the mitochondria**	Strict	Coalescent Constant size	-31,076.24
	Uncorrelated normal	Coalescent Constant size	-31,118.29
	Strict	Bayesian Skyline	-31,016.87
	Uncorrelated normal	Bayesian Skyline	-31,053.56

Supplementary Table 29 Marginal likelihoods resulting from the path sampling of different clock and tree models, for both the complete and the coding portion of the mitochondrial genome, using the oldest dates of Goyet Q57-2 as prior, with the Thorin prior informed by the associated radiocarbon dates. The best model is highlighted in blue.

Genome	Selected clock	Selected tree	Marginal log-likelihood for that model
**Complete mitochondria**	Strict	Coalescent Constant size	-36,429.87
	Uncorrelated normal	Coalescent Constant size	-36,412.50
	Strict	Bayesian Skyline	-36,373.22
	Uncorrelated normal	Bayesian Skyline	-36,392.28
**Coding Region of the mitochondria**	Strict	Coalescent Constant size	-31,084.80
	Uncorrelated normal	Coalescent Constant size	-31,104.54
	Strict	Bayesian Skyline	-31,026.04
	Uncorrelated normal	Bayesian Skyline	-31,054.28

Supplementary Table 30 Marginal likelihoods resulting from the path sampling of different clock and tree models, for both the complete and the coding portion of the mitochondrial genome, using the oldest dates of Goyet Q57-2 as prior, with the Couvin prior informed by the associated radiocarbon dates. The best model is highlighted in blue.

Genome	Selected clock	Selected tree	Marginal log-likelihood for that model
**Complete mitochondria**	Strict	Coalescent Constant size	-36,427.51
	Uncorrelated normal	Coalescent Constant size	-36,417.53
	Strict	Bayesian Skyline	-36,377.03
	Uncorrelated normal	Bayesian Skyline	-36,380.56
**Coding Region of the mitochondria**	Strict	Coalescent Constant size	-31,079.81
	Uncorrelated normal	Coalescent Constant size	-31,107.62
	Strict	Bayesian Skyline	-31,027.54
	Uncorrelated normal	Bayesian Skyline	-31,062.78

We generated three independent BEAST2 runs using the best models, with the same MCMC parameters as for the model selection. We combined the results using logcombiner2 version 2.7.5[45] and used FigTree v1.4.4 (http://tree.bio.ed.ac.uk/software/figtree/) to visualise and annotate the final trees shown in Supplementary Figures 28 and 29. The 95% highest posterior density intervals (HPDI) can be found in Supplementary Data Table 6.

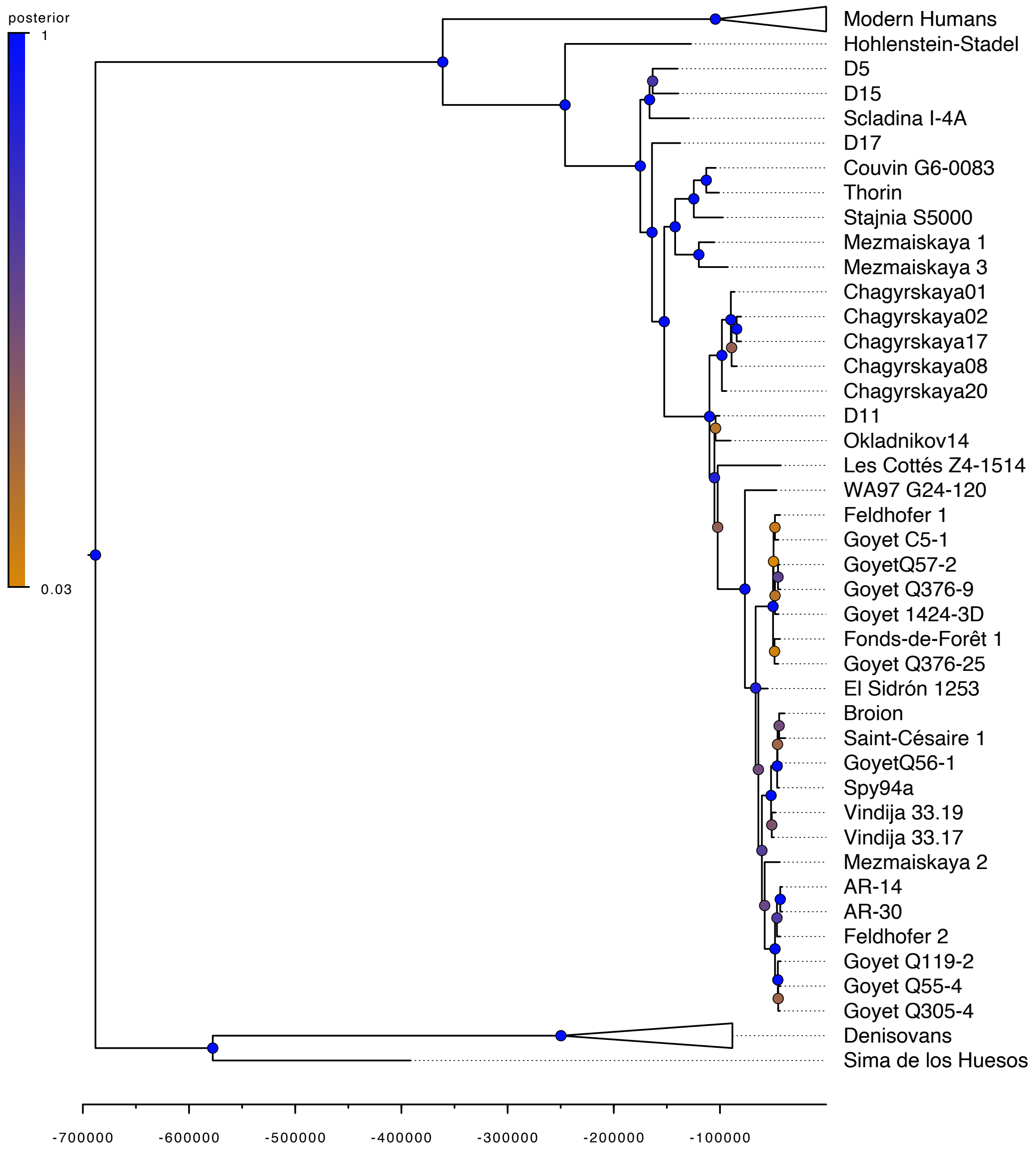

Supplementary Figure 28 Hominin mtDNA tree generated with BEAST2 using the complete mitochondrial genome, the oldest dates of Goyet Q57-2 as prior, and flat priors for Thorin and Couvin. Each node is coloured based on the posterior probability of the branch and the x-axis represents the time in years before present.

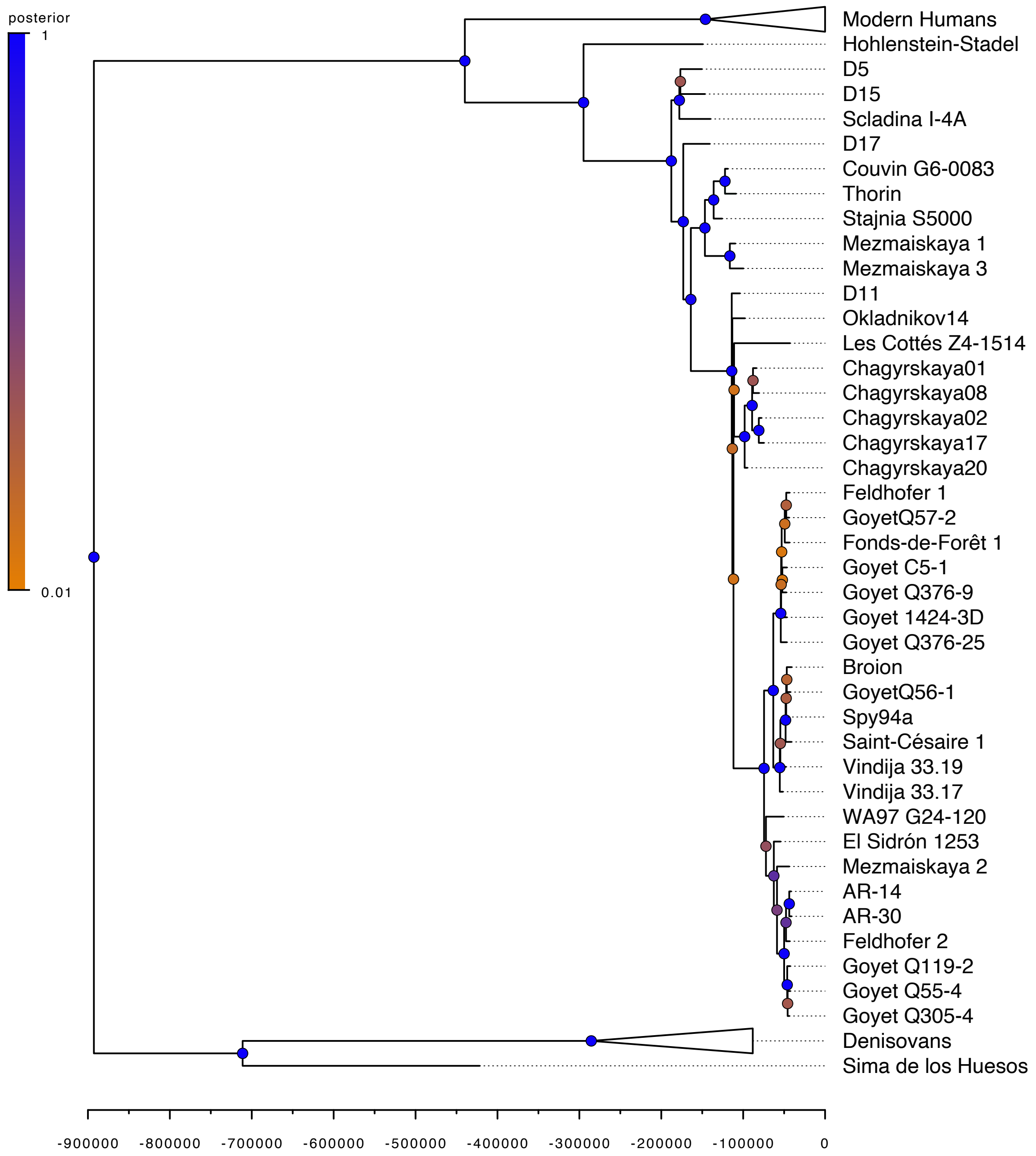

Supplementary Figure 29 Hominin mtDNA tree generated with BEAST2 using the coding region of the mitochondrial genome, the oldest dates of Goyet Q57-2 as prior, and flat priors for Thorin and Couvin. Each node is coloured based on the posterior probability of the branch and the x-axis represents the time in years before present.

We assessed the effect that prior choice for Goyet Q57-2 had on all the resulting genetic dates. For this, we compared the probability distributions on the ages of the focus Neandertals, while using either the older or the younger radiocarbon dates available for Goyet Q57-2, and both the complete mitochondrial genome and only the coding region (Supplementary Figure 30). Beyond making the genetic date of Goyet Q57-2 about 2,000 years older or younger (with the younger and older prior, respectively), none of the other results were significatively affected. We report more extensively (i.e., in all of the previously cited tables) the values for the older prior, as the older dates are more likely to represent the true age of the individual, assuming that the discrepancies in radiocarbon dates arose due to contamination with modern carbon.

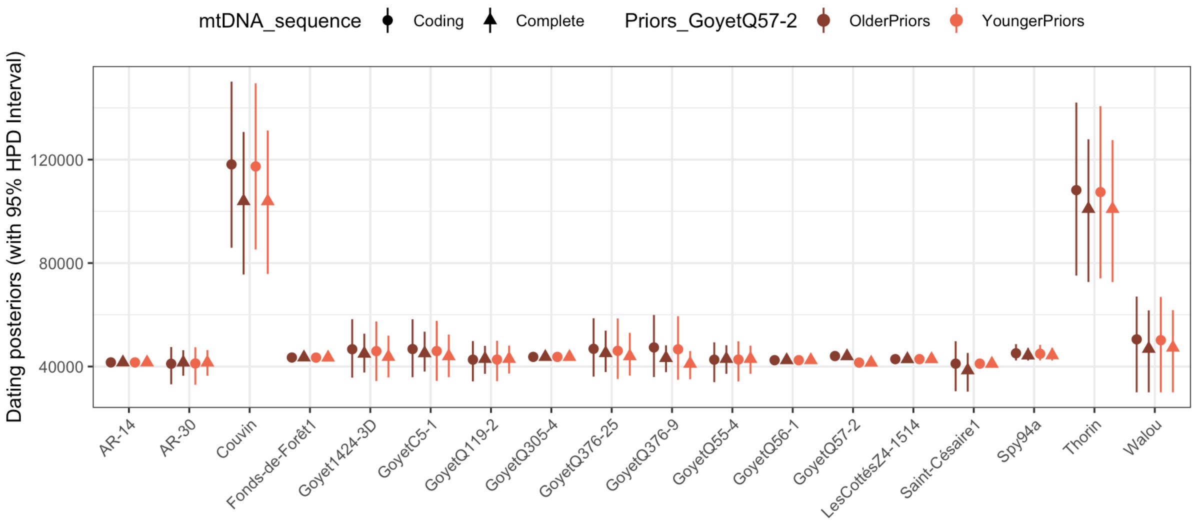

Supplementary Figure 30 Estimated genetic dates (with their respective 95% HPDI) for all the focus Neandertals. The colours indicate whether the prior used for Goyet Q57-2 was 44,880 - 43,160 calBP (i.e., older date) or 42,000 - 41,100 calBP (i.e., younger date). The shapes indicate which portion of the mitochondrial genome was analysed.

In contrast, the effects of the priors of Couvin and Thorin on their resulting genetic dates are much larger (Extended Data Fig. 2). When we specify a flat prior between 30,000 and 200,000 years BP, and thus the ages are largely unconstrained, the dates of these two Neandertals exceed 100,000 years BP. However, when we use the ~48,000 years BP prior for Thorin, the molecular age of Couvin is lowered from 118,140 years BP (150,170 - 85,906 years BP, 95% HPDI) to 69,052 years BP (89,680 - 50,445 years BP, 95% HPDI). The reverse is also true, and to a greater extent as well: when we use the ~46,000 years BP prior for Couvin, the molecular age of Thorin is lowered from 108,210 (142,070 - 75, 155 years BP, 95% HPDI) to 49,137 years BP (66,431 - 30,066 years BP, 95% HPDI).

The age estimates of other Neandertals in the same clade, such as Mezmaiskaya 1 and Stajnia S5000, are also affected by these prior modifications. They become younger when the radiocarbon dates of either Couvin or Thorin are used to inform the prior by up to 50,000 years (albeit not overlapping the age ranges of Late Neandertals). The molecular dates of Mezmaiskaya 1, Mezmaiskaya 3 and Stajnia S5000 are 107,840 years BP (142,330 - 75,819 years BP, 95% HPDI), 97,817 years BP (132,480 - 64,610 years BP, 95% HPDI) and 124,160 years BP (156,570 - 93,421 years BP, 95% HPDI), respectively, when we use flat priors. In contrast, when we use the Thorin radiocarbon date priors, these change to 81,021 years BP (108,840 - 56,959 years BP, 95% HPDI), 72,035 years BP (100,280 - 48,423 years BP, 95% HPDI) and 86,310 years BP (112,640 - 63,080 years BP, 95% HPDI), in that order. Similarly, when we use the Couvin radiocarbon date priors, their respective molecular ages are lowered to 76,837 years BP (104,340 - 53,101 years BP, 95% HPDI), 68,169 years BP (95,666 - 44,966 years BP, 95% HPDI) and 78,132 years BP (104,490 - 54,018 years BP, 95% HPDI). These results are consistent between all population size and mutational clock models we evaluated.

In conclusion, with the exception of Couvin, all the newly reported individuals here fall inside the variation of, and date-range of Late Neandertals. Couvin falls on the same mitochondrial branch as Thorin, and the two Neandertals are most likely of a similar age. However, the exact results differ starkly based on the assumptions and priors used. Unconstrained genetic dating puts both Couvin and Thorin as much older Neandertals, whereas adding the dating from either of the two specimens to the model pulls them towards a much younger date.

References

1. Fu, Q. *et al.* DNA analysis of an early modern human from Tianyuan Cave, China. *Proc. Natl. Acad. Sci.* **110**, 2223–2227 (2013).
2. Slon, V. *et al.* Neandertal and Denisovan DNA from Pleistocene sediments. *Science* **356**, 605–608 (2017).
3. Andrews, R. M. *et al.* Reanalysis and revision of the Cambridge reference sequence for human mitochondrial DNA. *Nat. Genet.* **23**, 147–147 (1999).
4. Slon, V. *et al.* Mammalian mitochondrial capture, a tool for rapid screening of DNA preservation in faunal and undiagnostic remains, and its application to Middle Pleistocene specimens from Qesem Cave (Israel). *Quat. Int.* **398**, 210–218 (2016).
5. Renaud, G., Stenzel, U. & Kelso, J. leeHom: adaptor trimming and merging for Illumina sequencing reads. *Nucleic Acids Res.* **42**, e141 (2014).
6. Green, R. E. *et al.* A Complete Neandertal Mitochondrial Genome Sequence Determined by High-Throughput Sequencing. *Cell* **134**, 416–426 (2008).
7. Li, H. & Durbin, R. Inference of human population history from individual whole-genome sequences. *Nature* **475**, 493–496 (2011).

8. Meyer, M. *et al.* A High-Coverage Genome Sequence from an Archaic Denisovan Individual. *Science* **338**, 222–226 (2012).
9. Prüfer, K. *et al.* The complete genome sequence of a Neanderthal from the Altai Mountains. *Nature* **505**, 43–49 (2014).
10. Meyer, M. *et al.* Nuclear DNA sequences from the Middle Pleistocene Sima de los Huesos hominins. *Nature* **531**, 504–507 (2016).
11. Meyer, M. *et al.* A mitochondrial genome sequence of a hominin from Sima de los Huesos. *Nature* **505**, 403–406 (2014).
12. Rougier, H. *et al.* Neandertal cannibalism and Neandertal bones used as tools in Northern Europe. *Sci. Rep.* **6**, 29005 (2016).
13. Hajdinjak, M. *et al.* Reconstructing the genetic history of late Neanderthals. *Nature* **555**, 652–656 (2018).
14. Devièse, T. *et al.* Reevaluating the timing of Neanderthal disappearance in Northwest Europe. *Proc. Natl. Acad. Sci.* **118**, e2022466118 (2021).
15. Kumar, S., Stecher, G., Li, M., Knyaz, C. & Tamura, K. MEGA X: Molecular Evolutionary Genetics Analysis across Computing Platforms. *Mol. Biol. Evol.* **35**, 1547–1549 (2018).
16. Li, H. *et al.* The Sequence Alignment/Map format and SAMtools. *Bioinformatics* **25**, 2078–2079 (2009).
17. Liu, C. *et al.* Presence and transmission of mitochondrial heteroplasmic mutations in human populations of European and African ancestry. *Mitochondrion* **60**, 33–42 (2021).
18. Skov, L. *et al.* Genetic insights into the social organization of Neanderthals. *Nature* **610**, 519–525 (2022).
19. Ingman, M., Kaessmann, H., Pääbo, S. & Gyllensten, U. Mitochondrial genome variation and the origin of modern humans. *Nature* **408**, 708–713 (2000).
20. Ermini, L. *et al.* Complete Mitochondrial Genome Sequence of the Tyrolean Iceman. *Curr. Biol.* **18**, 1687–1693 (2008).
21. Gilbert, M. T. P. *et al.* Paleo-Eskimo mtDNA Genome Reveals Matrilineal Discontinuity in Greenland. *Science* **320**, 1787–1789 (2008).
22. Krause, J. *et al.* A Complete mtDNA Genome of an Early Modern human from Kostenki, Russia. *Curr. Biol.* **20**, 231–236 (2010).
23. Fu, Q. *et al.* A Revised Timescale for Human Evolution Based on Ancient Mitochondrial Genomes. *Curr. Biol.* **23**, 553–559 (2013).
24. Mittnik, A. & Krause, J. Genetic analysis of the Dolní Věstonice human remains. in *Dolní Věstonice II: Chronostratigraphy, Paleoethnology, Paleoanthropology* 377–384 (Academy of Sciences of the Czech Republic, Institute of Archeology, Brno, 2016).
25. Briggs, A. W. *et al.* Targeted Retrieval and Analysis of Five Neandertal mtDNA Genomes. *Science* **325**, 318–321 (2009).

26. Skoglund, P. *et al.* Separating endogenous ancient DNA from modern day contamination in a Siberian Neandertal. *Proc. Natl. Acad. Sci.* **111**, 2229–2234 (2014).
27. Brown, S. *et al.* Identification of a new hominin bone from Denisova Cave, Siberia using collagen fingerprinting and mitochondrial DNA analysis. *Sci. Rep.* **6**, 23559 (2016).
28. Posth, C. *et al.* Deeply divergent archaic mitochondrial genome provides lower time boundary for African gene flow into Neanderthals. *Nat. Commun.* **8**, 16046 (2017).
29. Peyrégne, S. *et al.* Nuclear DNA from two early Neandertals reveals 80,000 years of genetic continuity in Europe. *Sci. Adv.* **5**, eaaw5873 (2019).
30. Brown, S. *et al.* The earliest Denisovans and their cultural adaptation. *Nat. Ecol. Evol.* **6**, 28–35 (2021).
31. Andreeva, T. V. *et al.* Genomic analysis of a novel Neanderthal from Mezmaiskaya Cave provides insights into the genetic relationships of Middle Palaeolithic populations. *Sci. Rep.* **12**, 13016 (2022).
32. Slimak, L. *et al.* Long genetic and social isolation in Neanderthals before their extinction. *Cell Genomics* **4**, 100593 (2024).
33. Krause, J. *et al.* The complete mitochondrial DNA genome of an unknown hominin from southern Siberia. *Nature* **464**, 894–897 (2010).
34. Reich, D. *et al.* Genetic history of an archaic hominin group from Denisova Cave in Siberia. *Nature* **468**, 1053–1060 (2010).
35. Sawyer, S. *et al.* Nuclear and mitochondrial DNA sequences from two Denisovan individuals. *Proc. Natl. Acad. Sci.* **112**, 15696–15700 (2015).
36. Slon, V. *et al.* A fourth Denisovan individual. *Sci. Adv.* **3**, e1700186 (2017).
37. Katoh, K. & Standley, D. M. MAFFT Multiple Sequence Alignment Software Version 7: Improvements in Performance and Usability. *Mol. Biol. Evol.* **30**, 772–780 (2013).
38. Stecher, G., Tamura, K. & Kumar, S. Molecular Evolutionary Genetics Analysis (MEGA) for macOS. *Mol. Biol. Evol.* **37**, 1237–1239 (2020).
39. Picin, A. *et al.* New perspectives on Neanderthal dispersal and turnover from Stajnia Cave (Poland). *Sci. Rep.* **10**, 14778 (2020).
40. Toussaint, M. *et al.* The Neandertal lower right deciduous second molar from Trou de l'Abîme at Couvin, Belgium. *J. Hum. Evol.* **58**, 56–67 (2010).
41. Ramsey, C. B. Methods for Summarizing Radiocarbon Datasets. *Radiocarbon* **59**, 1809–1833 (2017).
42. Reimer, P. J. *et al.* The IntCal20 Northern Hemisphere Radiocarbon Age Calibration Curve (0–55 cal kBP). *Radiocarbon* **62**, 725–757 (2020).
43. Bray, N. L., Pimentel, H., Melsted, P. & Pachter, L. Near-optimal probabilistic RNA-seq quantification. *Nat. Biotechnol.* **34**, 525–527 (2016).

44. Vernot, B. *et al.* Unearthing Neanderthal population history using nuclear and mitochondrial DNA from cave sediments. *Science* **372**, eabf1667 (2021).
45. Bouckaert, R. *et al.* BEAST 2: A Software Platform for Bayesian Evolutionary Analysis. *PLoS Comput. Biol.* **10**, e1003537 (2014).
46. Gouy, M., Guindon, S. & Gascuel, O. SeaView Version 4: A Multiplatform Graphical User Interface for Sequence Alignment and Phylogenetic Tree Building. *Mol. Biol. Evol.* **27**, 221–224 (2010).
47. Fu, Q. *et al.* Genome sequence of a 45,000-year-old modern human from western Siberia. *Nature* **514**, 445–449 (2014).
48. Ward, G. K. & Wilson, S. R. Prodecures for comparing and combining radiocarbon age determinations: a critique. *Archaeometry* **20**, 19–31 (1978).
49. Ascough, P., Cook, G. & Dugmore, A. Methodological approaches to determining the marine radiocarbon reservoir effect. *Prog. Phys. Geogr. Earth Environ.* **29**, 532–547 (2005).
50. Keaveney, E. M. & Reimer, P. J. Understanding the variability in freshwater radiocarbon reservoir offsets: a cautionary tale. *J. Archaeol. Sci.* **39**, 1306–1316 (2012).
51. Johnstone-Belford, E., Fallon, S. J., Dipnall, J. F. & Blau, S. The importance of bone sample selection when using radiocarbon analysis in cases of unidentified human remains. *Forensic Sci. Int.* **341**, 111480 (2022).
52. Hublin, J.-J. The last Neanderthal. *Proc. Natl. Acad. Sci.* **114**, 10520–10522 (2017).
53. DeNiro, M. J. Postmortem preservation and alteration of in vivo bone collagen isotope ratios in relation to paleodietary reconstruction. *Nature* 806–809 (1985).
54. Tamura & Nei, Masatoshi. Estimation of the number of nucleotide substitutions in the control region of mitochondrial DNA in humans and chimpanzees. *Mol. Biol. Evol.* (1993) doi:10.1093/oxfordjournals.molbev.a040023.
55. Drummond, A. J. Bayesian Coalescent Inference of Past Population Dynamics from Molecular Sequences. *Mol. Biol. Evol.* **22**, 1185–1192 (2005).

11. Y chromosome captures

11.1 Y chromosome enrichment and sequencing

To date, Spy 94a is the only male Neandertal from Northwestern Europe that has been genetically characterised[1]. Here, we genetically sexed four additional individuals as males: Goyet 1424-3D, Goyet Q305-1, Trou Magrite 2422-36 and Fonds-de-Forêt 1. Unfortunately, the low coverage and high-contamination of Goyet 1424-3D precluded us from including this individual's Y chromosome sequences in our analyses. We performed targeted enrichment of five libraries of the remaining three individuals, alongside three negative controls, for the 6.91 MB of the Y chromosome using the same capture array and laboratory protocols as in prior publications[1].

We sequenced these libraries using Illumina's HiSeq 4000 and NextSeq platforms. We processed the data as described in Section 10, except that we mapped the sequences to the hg19 reference genome[2] and restricted our analyses to only "on-target" positions (i.e., to the 6.91 MB of the Y chromosome targeted by the capture array).

Summary statistics for the sequenced libraries are reported in Supplementary Data Table 3.1. The C-to-T frequencies at the ends of the sequences indicated that ancient DNA was present for all five Neandertal libraries and for none of the controls. After merging all the data for each individual, on-target sites were covered on average 1.75 times in Fonds-de-Forêt 1, 8.19 times in Goyet Q305-1, and 3.78 times in Trou Magrite 2422-36. Based on the AuthentiCT estimates of autosomal contamination (Section 13), we expect ~6% of contaminant sequences for Fonds-de-Forêt 1 and Goyet Q305-1, and ~15% for Trou Magrite 2422-36. As a quality control, we repeated all of our analyses by filtering for deaminated sequences only. After this filtering, the average coverage decreased to 0.27 fold for Fonds-de-Forêt 1, 2.45 fold for Goyet Q305-1 and 1.10 fold for Trou Magrite 2422-36.

11.2 Y chromosome lineage assignment

We used the same set of diagnostic sites that had been used previously[3] for the lineage assignment of the recovered Y chromosome sequences (Supplementary Figure 31). In short, these had been defined using four modern humans[4–6], two Denisovans, and eleven Neandertals: Mezmaiskaya 2, Spy 94a, El Sidrón 1253[1], Chagyrskaya A, Chagyrskaya B, Chagyrskaya C, Chagyrskaya D, Chagyrskaya E, Chagyrskaya G, and Chagyrskaya K[3]. Following the approach described[3], we used the derived sites specific for each branch of the Y chromosome tree to count the proportion of derived variants that were present in each of our samples. We polarised the SNPs by using the chimpanzee reference genome (panTro4) as the ancestral state. Given that transversions could have arisen from deaminated reads, we

excluded C-to-T mismatches on reads mapping to the forward and G-to-A mismatches on reads mapping to the reverse strand, respectively.

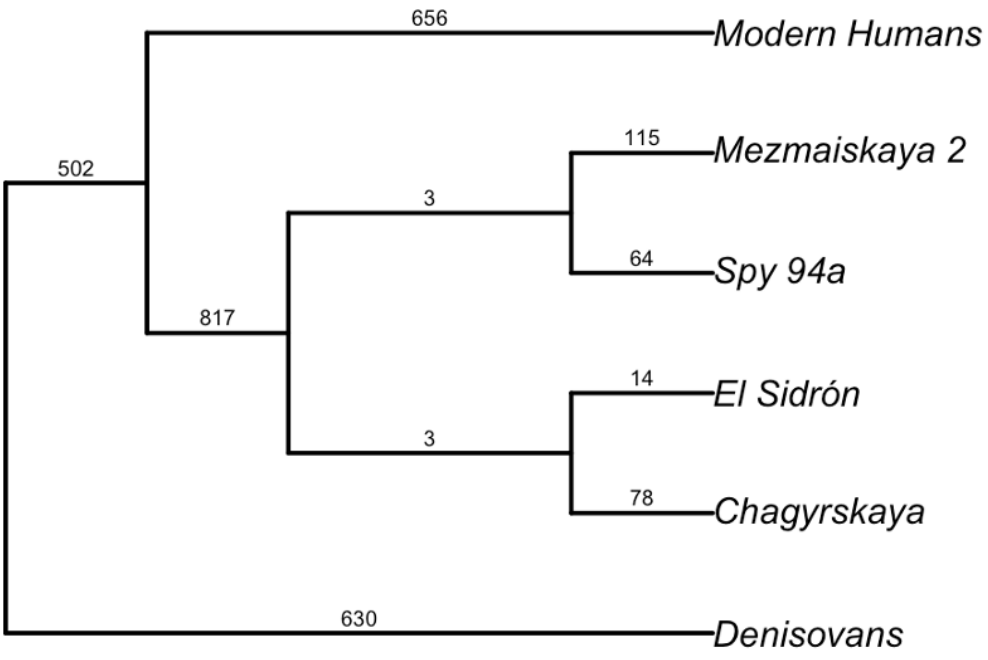

Supplementary Figure 31 Number of lineage diagnostic positions used for each branch of the Y chromosome tree of archaic hominins.

The lineage assignment results are summarised in Supplementary Tables 31 and 32; and Supplementary Figure 32. The proportion of sequences that fall on the modern human branch provides an estimate of the present-day human DNA contamination levels. These estimates were slightly higher than the autosomal estimates (Section 13): ~8% each for Fonds-de-Forêt 1 and Goyet Q305-1, and ~16% for Trou Magrite 2422-36. When repeating this analysis using deaminated sequences only, we obtained contamination estimates of 3.73%, 0.41% and 2.13%, respectively.

The lineage assignment analysis also provided insights about which previously sequenced Neandertal Y chromosomes were most similar to our samples. Surprisingly, both Fonds-de-Forêt 1 and Trou Magrite 2422-36 show highest support for the Chagyrskaya-specific Y chromosome sites, with 34.96% (95% CI: 27.1-43.73%) and 16.13% (95% CI: 7.09-32.63%) of all sequences matching Chagyrskaya Y chromosome derived sites, respectively. This proportion increases to 38.89% (95% CI: 20.31- 61.38%) and 40% (95% CI: 11.76-76.93%) when restricting the analyses to deaminated fragments only, albeit with a much smaller number of sequences overlapping the diagnostic sites. In contrast, Y chromosome sequences of Goyet Q305-1 show highest support for the Mezmaiskaya 2-specific Y chromosome sites,

with 40.42% (95% CI: 36.98-43.96%) using all sequences, and 44.81% (95% CI: 38.27-51.54%) using deaminated sequences only.

Supplementary Table 31 Support for each lineage in the Y chromosome hominin tree using all sequences. CI - confidence intervals

	Fonds-de-Forêt 1			Goyet Q305-1			Trou Magrite 2422-36		
Lineage	Number of reads (total)	% Derived	% Derived (95% CI)	Number of reads (total)	% Derived	% Derived (95% CI)	Number of reads (total)	% Derived	% Derived (95% CI)
Denisova	1297	0.85	[0.47-1.51]	6457	0.09	[0.04-0.2]	281	0	[0.0-1.35]
Modern human /Neandertal	1057	98.39	[97.44-98.99]	5407	98.24	[97.86-98.56]	274	97.81	[95.31-98.99]
Modern human	1012	8.4	[6.84-10.27]	3365	8.23	[7.35-9.21]	172	16.28	[11.51-22.52]
Neandertal	1272	89.54	[87.74-91.11]	4233	86.16	[85.08-87.16]	217	82.03	[76.38-86.57]
El Sidrón /Chagyrskaya	5	60	[23.07-88.24]	26	0	[0.0-12.87]	0	NA	NA
El Sidrón	21	4.76	[0.85-22.67]	170	0	[0.0-2.21]	7	0	[0.0-35.43]
Chagyrskaya	**123**	**34.96**	**[27.1-43.73]**	644	0	[0.0-0.59]	**31**	**16.13**	**[7.09-32.63]**
Mezmaiskaya /Spy	6	0	[0.0-39.03]	6	83.33	[43.65-96.99]	4	0	[0.0-48.99]
Mezmaiskaya 2	249	0	[0.0-1.52]	**757**	**40.42**	**[36.98-43.96]**	43	0	[0.0-8.2]
Spy 94a	152	0	[0.0-2.46]	691	0.72	[0.31-1.68]	32	0	[0.0-10.72]

Supplementary Table 32 Support for each lineage in the Y chromosome hominin tree using deaminated sequences only. CI - confidence intervals

	Fonds-de-Forêt 1			Goyet Q305-1			Trou Magrite 2422-36		
Lineage	Number of reads (total)	% Derived	% Derived (95% CI)	Number of reads (total)	% Derived	% Derived (95% CI)	Number of reads (total)	% Derived	% Derived (95% CI)
Denisova	199	1.01	[0.28-3.59]	1907	0.05	[0.01-0.3]	75	0.00	[-0.0-4.87]
Modern human /Neandertal	189	95.77	[91.87-97.84]	1637	97.01	[96.06-97.73]	70	97.14	[90.17-99.21]
Modern human	161	3.73	[1.72-7.89]	969	0.41	[0.16-1.06]	47	2.13	[0.38-11.11]
Neandertal	193	92.75	[88.19-95.63]	1188	92.26	[90.6-93.64]	53	92.45	[82.14-97.03]
El Sidrón /Chagyrskaya	1	100.00	[20.65-100.0]	7	0.00	[0.0-35.43]	0	0.00	[NA-NA]
El Sidrón	4	0.00	[0.0-48.99]	43	0.00	[0.0-8.2]	1	0.00	[0.0-79.35]
Chagyrskaya	**18**	**38.89**	**[20.31-61.38]**	186	0.00	[0.0-2.02]	**5**	**40.00**	**[11.76-76.93]**
Mezmaiskaya /Spy	1	0.00	[0.0-79.35]	2	100.00	[34.24-100.0]	1	0.00	[0.0-79.35]
Mezmaiskaya 2	31	0.00	[0.0-11.03]	**212**	**44.81**	**[38.27-51.54]**	9	0.00	[0.0-29.91]
Spy 94a	24	0.00	[0.0-13.8]	208	0.96	[0.26-3.44]	11	0.00	[0.0-25.88]

A) Fonds-de-Forêt 1

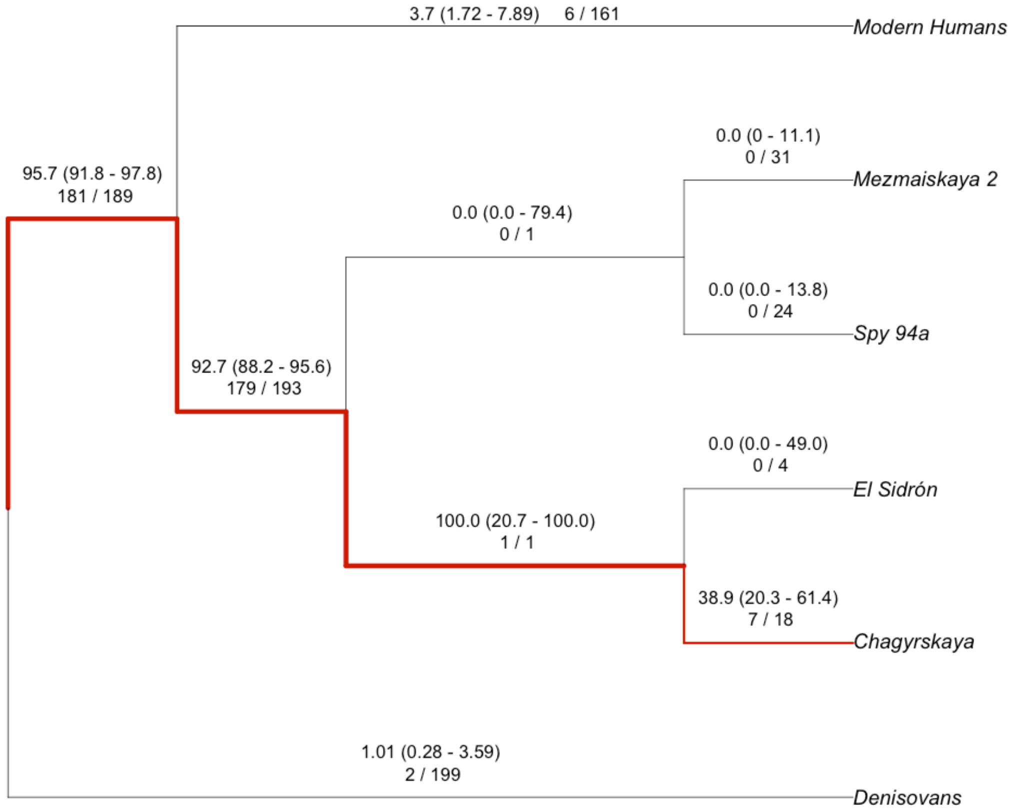

B) Goyet Q305-1

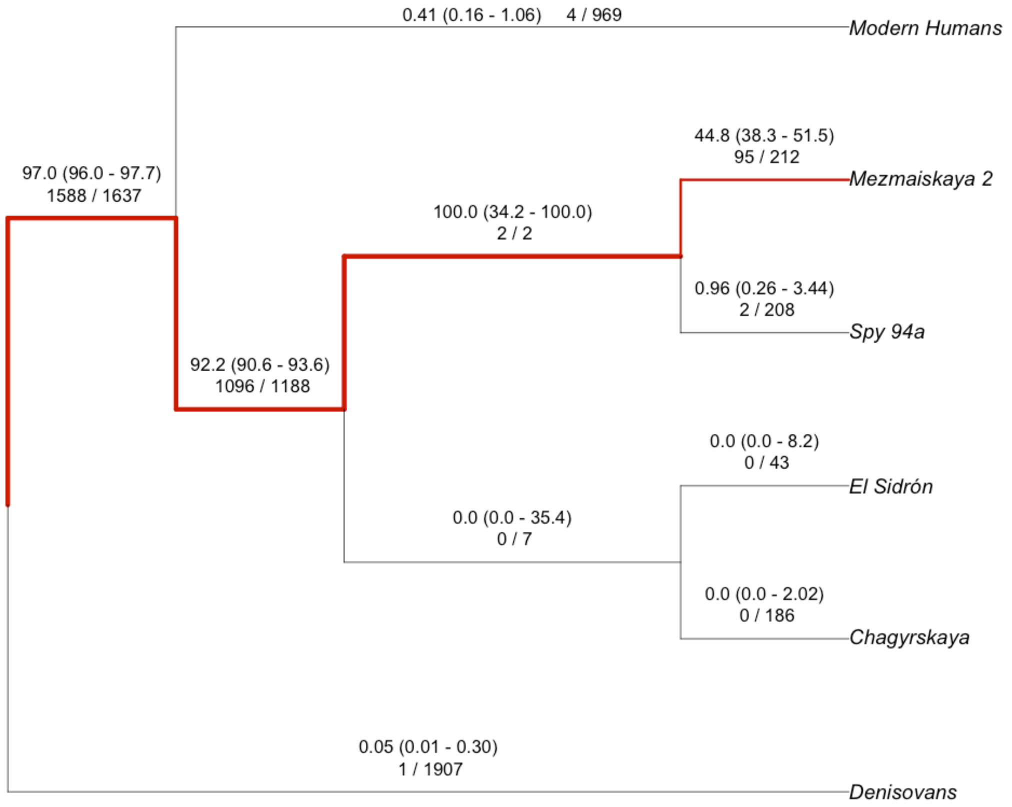

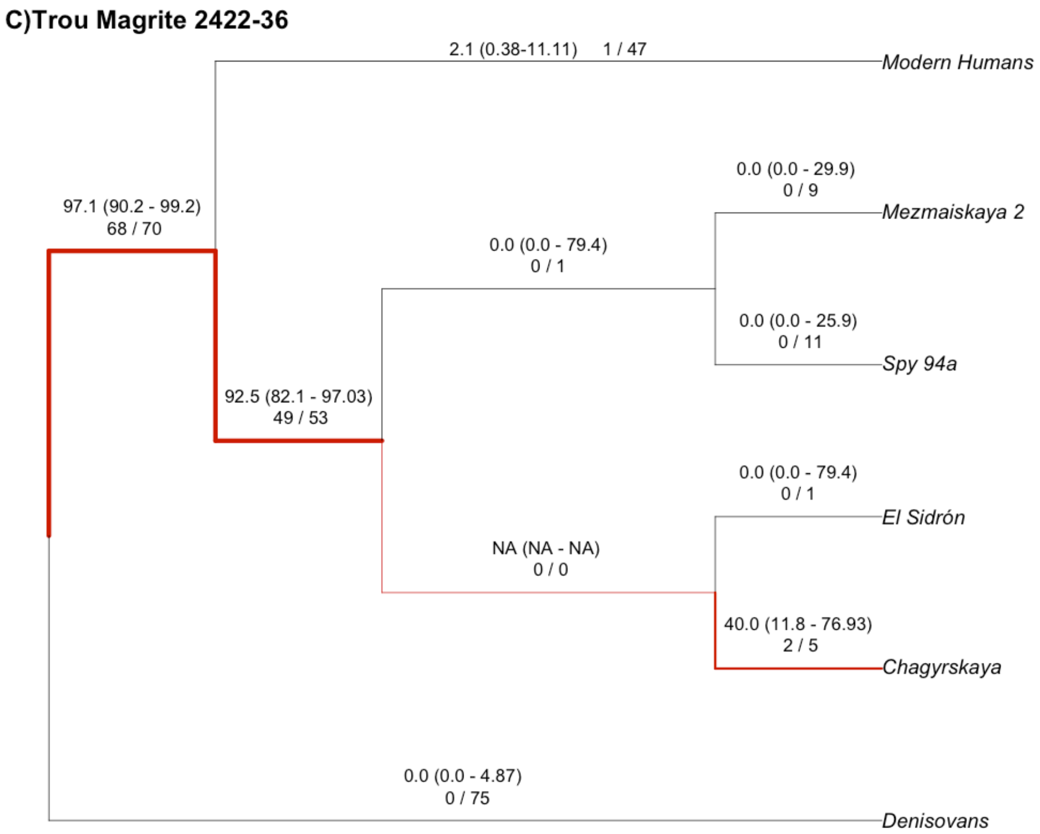

Supplementary Figure 32 Lineage support as point estimate (with associated 95% confidence intervals) for the Y chromosomes of A) Fonds-de-Forêt 1, B) Goyet Q305-1, and C) Trou Magrite 2422-36 after filtering for deaminated-reads only. The number of reads with the derived alleles / total number of reads is shown below. The branch with the highest genetic affinity with each sample is highlighted in red, its width proportional to the support.

11.4 Variant calling

We called the variants on the Y chromosomes of the three male Neandertals following Skov et al.[3], with a similar approach to Petr et al.[1]. Due to the modern human contamination detected in the previous section, we only performed the variant calling after subsetting the sequences to the ones with terminal C-to-T substitutions. We called a base at a given site if that base was supported by at least three reads and if 90% of reads agreed on a consensus base. In addition, we applied the criteria of minimum of one T on the reverse strand if the mutation was a C-to-T, or minimum one A on the forward strand if the mutation was G-to-A. After this filtering, we identified 17 variants in Fonds-de-Forêt 1, one in Trou Magrite 2422-36 and 754 in Goyet Q305-1.

Next, we used Variant Effect Predictor (VEP)[7] with the GRCh37.p13 assembly and the "Ensembl/GENCODE transcripts" as the transcript database with the default parameters to annotate these variants (Supplementary Data Tables 4.1, 4.2 and 4.3). Based on this, all variants annotated for

Fonds-de-Forêt 1 were classified as "modifier impact variants", in other words, variants occurring in non-coding regions of the genome or affecting non-coding genes, which challenges the prediction of its impact or lacks evidence of impact. Four out of the 17 modifier impact variants overlapped with known genes. However, they were either intron variants or associated with processed pseudogenes (Supplementary Data Tables 4.4, 4.5 and 4.6). One of them was Ubiquitin Specific Peptidase 9 Y-Linked (*USP9Y,* ENSG00000114374, a protein coding gene expressed in the male germ line stem cell), which was previously identified[8,3] and known to be related to spermatogenic failure.

Similarly to Fonds-de-Forêt 1, the majority of the variants on the Y chromosome of Goyet Q305-1 were intron or intergenic variants, with modifier impact. Only three exceptions were classified as "low" impact, i.e., variants presumed to be mostly benign or very unlikely to affect protein function. These were found in association with genes *ARSDP1* (ENSG00000225117, an unprocessed pseudogene), *BCORP1* (ENSG00000215580, a processed transcript or pseudogene) and *USP9Y*. As mentioned previously, the latter gene had also been annotated in Fonds-de-Forêt 1, albeit without an effect on gene function. This particular variant associated with this gene in Goyet Q305-1 could not be called either in Fonds-de-Forêt 1 or TM2422-36, due to low coverage. Finally, due to the low coverage we could only identify a single variant in TM2422-36, which was a transversion.

To evaluate the possible impact of ancient DNA damage on variant calling, we estimated the transversion/transition (TS/TV) ratio among the called variants. The expected value for Neandertal Y chromosomes was previously reported to be ~1.64[1]. We found that in Fonds-de-Forêt 1, nine of the variants were transitions and eight were transversions, resulting in a TS/TV ratio of 1.13. For Goyet Q305-1, this ratio was 1.75 (480 transitions and 274 transversions), which is close to the previous findings[1]. It was not possible to calculate the values for TM2422-36.

References

1. Petr, M. *et al.* The evolutionary history of Neanderthal and Denisovan Y chromosomes. *Science* **369**, 1653–1656 (2020).
2. Church, D. M. *et al.* Modernizing Reference Genome Assemblies. *PLoS Biol.* **9**, e1001091 (2011).
3. Skov, L. *et al.* Genetic insights into the social organization of Neanderthals. *Nature* **610**, 519–525 (2022).
4. Mendez, F. L. *et al.* An African American Paternal Lineage Adds an Extremely Ancient Root to the Human Y Chromosome Phylogenetic Tree. *Am. J. Hum. Genet.* **92**, 454–459 (2013).
5. Karmin, M. *et al.* A recent bottleneck of Y chromosome diversity coincides with a global change in culture. *Genome Res.* **25**, 459–466 (2015).

6. Mallick, S. *et al.* The Simons Genome Diversity Project: 300 genomes from 142 diverse populations. *Nature* **538**, 201–206 (2016).
7. McLaren, W. *et al.* The Ensembl Variant Effect Predictor. *Genome Biol.* **17**, 122 (2016).
8. Mendez, F. L., Poznik, G. D., Castellano, S. & Bustamante, C. D. The Divergence of Neandertal and Modern human Y Chromosomes. *Am. J. Hum. Genet.* **98**, 728–734 (2016).

12. ArchaicPlus nuclear capture ascertainment

We designed the "ArchaicPlus" capture array with two main goals: (1) to improve the analysis of archaic samples by capturing a larger number of nuclear SNPs informative for Neandertal and Denisovan variation and (2) to provide accurate estimates of present-day human contamination. Broadly, the design relied on the following genomes:

- Three high coverage Neandertal genomes from Vindija 33.19[1], D5[2] and Chagyrskaya 8[3];
- One high coverage Denisovan genome from Denisova 3[4];
- 504 present-day genomes from Africa from the 1000 Genomes phase 3 project[5];
- Two great ape genomes, used as outgroups, from a chimpanzee (panTro4, GCA_000001515.4) and a gorilla (gorGor3, GCA_000151905.1).

Each of the high-coverage genomes was filtered according to the quality-control filters described in their respective publications. These included the removal of tandem-repeats and insertions-deletions, filtering for coverage stratified by GC-content with a minimum of a 10-fold coverage, restricting to the mappable regions of the genome with unique 35mers and a minimum mapping quality of 25. After applying these filters, we combined the resulting genotypes into a single file using bcftools version 1.4[6]. Regions that did not pass the filters were set as missing.

The "ArchaicPlus" ascertainment is composed of five main types of sites:

1. **Sites that are variable in the archaic high-coverage genomes**. We included polymorphic sites, i.e., sites with allelic frequencies between 0.1 and 0.9. As long as both the reference and the alternative alleles were present in at least one archaic individual, even if all the other genomes had missing data, or sites which were filtered out due to the criteria described above, we included that site. In contrast to the previous nuclear capture array[7], this included both transversions and transitions. This resulted in a total of 2,659,117 sites.

2. **Sites that are fixed derived in Neandertals, and ancestral in Sub-Saharan Africans**. The polarisation here relied on the chimpanzee and gorilla genomes which were used as outgroups. For this category of sites, no missing data was allowed (i.e., all genomes must have a called genotype in each position). This restriction can severely limit the number of sites, as the missingness in the gorGor3 alignment to the human reference is notable. This resulted in a total of 57,691 sites.

3. **Sites that are mostly derived in Sub-Saharan modern humans, and mostly ancestral in Archaics.** This set of sites was designed to enable estimating present-day human contamination levels through the linear combination approach. We selected SNPs with large allele frequency differences between Archaics and present-day Sub-Saharan Africans, so that most Sub-Saharan Africans had the derived allele, and most Archaic hominins had the ancestral allele. The advantage of using non-fixed differences (in contrast to categories 2 and 4) is that these sites are more abundant, which increases the power of the linear combination approach for estimating levels of present day human contamination.

   After pseudo-haploidising the genomes of three Archaic individuals (Denisova 3, D5 and Vindija 33.19), as well as the Mbuti individual HGDP0456, we selected SNPs where the three former genomes carried the ancestral allele and the latter carried the derived allele. Because the linear combination approach needs a reference of the expected frequency in an independent archaic genome, we intentionally omitted Chagyrskaya 8 from this part of the array design (for more information, see Section 13). We used the chimpanzee (panTro4) and gorilla (gorGor3) genomes as outgroups. We allowed no missing data. The total equaled 776,710 sites.

4. **Sites that are fixed derived in Africans, and ancestral in Neandertals**. As with category 2, the polarisation relied on the chimpanzee (panTro4) and gorilla (gorGor3) genomes as outgroups, to define the ancestral alleles. We allowed no missing data. This filter resulted in a total of 15,554 sites.

5. **Y chromosome sites.** The goal of this category was to help with the genetic sexing of samples (together with the sites on the X chromosome from the previous four categories), as well as to provide basic phylogenetic information. Skov et al.[7], had defined 5,416 sites informative in the archaic hominin Y chromosome tree. We based our design on those, removing 104 putative recurrent mutations, and restricting the positions to biallelic variants only. This resulted in a total of 5,074 sites.

Overall, categories 1 and 5 were intended to accomplish the first objective of capturing as much of the known archaic variation as possible. The remaining categories were geared towards obtaining reliable contamination estimates as well as detecting possible modern human introgression. Sites from categories 2 and 4 are lineage-diagnostic, representing those sites that have been found to differ between archaic hominins and modern humans, with the downside that there are fewer of them. For this reason, and for the contamination estimate purposes, we mostly relied on category 3, that is, sites that are not completely fixed different between archaic hominins and modern humans. In the past, this approach

has proven useful to estimate contamination estimates based on a linear combination of the frequencies of derived alleles in both lineages[7,8].

Because some of the sites from the different categories overlap, the initial stages of array design resulted in a total of 3,486,778 sites. We next applied a set of stringent filters to improve the performance:

- **We removed Nuclear Mitochondrial DNA (NUMTs)** as defined previously[9]. This removed a total of 561 SNPs.
- **We removed probes with unexpected bases.** We filtered out all probes that contained bases with IUPAC nucleotide codes other than “A”, “C”, “T” or “G” (e. g. “M”, standing for “A or C”). This only had minor effects, as it removed only four SNPs.
- **We defined a minimum distance between targeted sites**, so as to avoid probe overlap and the ensuing reference-bias that this would cause. Considering that the probe length was 60 bp, and no flanking probes were used, the minimum distance to prevent probe-overlap was set to 61 bp. This filter removed a total of 850,885 SNPs.
- **We filtered for GC content between 25% and 75%.** The GC content of each probe was estimated based on a 60 bp probe design. The thresholds were based on previous capture experiments, in which probes with higher and lower %GC were rarely captured. This filter removed 212,160 SNPs.
- **We applied a repeat masker,** removing the probes that overlapped in any base with the UCSC hg19 annotated genome database’s track for simple repeats (http://hgdownload.soe.ucsc.edu/goldenPath/hg19/database/simpleRepeat.txt.gz). This removed 23,219 SNPs.
- **We applied DUST** to remove low-complexity probes[10,11]. A cut-off of 20 k-mers was selected, after exploring different alternatives (cut-offs of 10, 15, 20, 25 and 30). This removed 89,764 SNPs.
- **We removed tandem repeats** of mononucleotides (N=10) or dinucleotides (NN=5). Moreover, probes containing the sequence “CATACCGC” were also removed, because this 8 bp appeared in unexplained artifacts during the testing of previous versions of ArchaicPlus. This removed further 6,512 SNPs, resulting in a final total of 2,303,673 informative SNPs.

The probes were designed centered around a given SNP, with a total length of 60 bp taking hg19 as reference[12]. The original idea was to make full use of these 60 bp (i.e. 29 bp + SNP + 30 bp), but this was later changed to the in-house format of 52bp + adaptor sequence to enable probe slicing and amplification (i.e. 26 bp + SNP + 25 bp + 8bp adaptor). To further minimize potential reference-bias, we used a third allele rather than the reference or the alternative allele at the relevant SNP position.

We classified the resulting probes as “SR_only” or “SR+AllRep”, depending on whether they overlapped with the UCSC repeat tracks (http://hgdownload.soe.ucsc.edu/goldenPath/hg19/database/rmsk.txt.gz) or not. In contrast to the simple repeat USCS track used for our stringent filtering detailed above, these annotations go beyond simple repeats and also include transposable elements, regions of low complexity, satellites, etc. This decision was anticipating possible troubleshooting avenues in case the array did not perform as expected. In practice, it had no impact on the overall ascertainment, as we ended up using all sites (“SR_only” and “SR+AllRep”).

Finally, because this custom-array design was produced through Agilent Technologies, whose probe-sets have a maximum size of 974,016 sites, and we wanted to keep the “SR_only” and “SR+AllRep” sites separately, we faced the problem that the “SR_only” sites were slightly over the limit with 993,236 sites. To overcome this, we decided to remove 19,220 positions at random, taking care that none of them was from category 5, as Y chromosome sites are already scarce, so that the array could fit in three probe-sets, i.e., two probe-sets for the “SR+AllRep” sites and one for “SR_only” sites.
The final ascertainment contained 2,284,453 SNPs (Supplementary Figure 33, Supplementary Table 33).

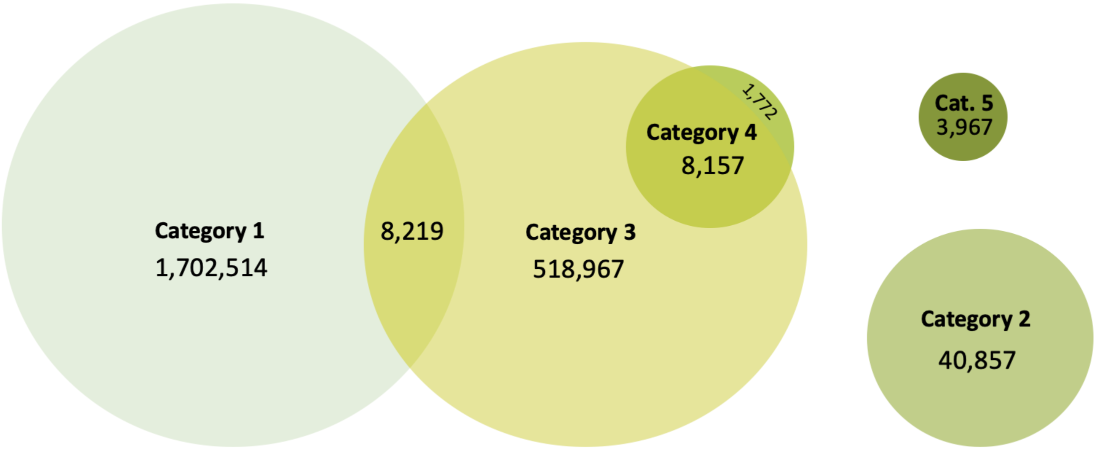

Supplementary Figure 33 Venn diagram of the number of sites for each of the five categories of the ArchaicPlus array after filtering.

Supplementary Table 33 Number of sites for each category of the ArchaicPlus array, before and after filtering.

	ArchaicPlus Ascertainment	# sites	%per array	# sites (accumulative)
Pre-filter	1 - Archaic variation	2659117	75.67	2659117
	2 - Fixed between Archaic - Africans (Archaic derived)	57691	1.64	2716808
	3 - Informative sites, human branch	776710	22.10	3493518
	4 - Fixed between Archaic - Africans (Africans derived)	15554	0.44	3509072
	5 - Y chromosome	5074	0.14	3514146
Total unique sites pre-filtering:				3486778
Filtering	NUMTS	-27929		3486217
	OVERLAP	-850889		2635328
	GC	-212160		2423168
	DUST + TRF	-112983		2310185
	remove CATACCGC	-311		2309874
	no N10, NN5	-6201		2303673
Subset to fit Agilent probe-sets (487.008 each)	Randomly removed	19220	0.83	
	Selected for array	2284453	99.17	2284453

References

1. Prüfer, K. *et al.* A high-coverage Neandertal genome from Vindija Cave in Croatia. *Science* **358**, 655–658 (2017).
2. Prüfer, K. *et al.* The complete genome sequence of a Neanderthal from the Altai Mountains. *Nature* **505**, 43–49 (2014).
3. Mafessoni, F. *et al.* A high-coverage Neandertal genome from Chagyrskaya Cave. *Proc. Natl. Acad. Sci.* **117**, 15132–15136 (2020).
4. Meyer, M. *et al.* A High-Coverage Genome Sequence from an Archaic Denisovan Individual. *Science* **338**, 222–226 (2012).
5. The 1000 Genomes Project Consortium *et al.* A global reference for human genetic variation. *Nature* **526**, 68–74 (2015).
6. Li, H. A statistical framework for SNP calling, mutation discovery, association mapping and population genetical parameter estimation from sequencing data. *Bioinformatics* **27**, 2987–2993 (2011).
7. Skov, L. *et al.* Genetic insights into the social organization of Neanderthals. *Nature* **610**, 519–525 (2022).
8. Peyrégne, S. *et al.* Nuclear DNA from two early Neandertals reveals 80,000 years of genetic continuity in Europe. *Sci. Adv.* **5**, eaaw5873 (2019).
9. Calabrese, F. M., Simone, D. & Attimonelli, M. Primates and mouse NumtS in the UCSC Genome Browser. *BMC Bioinformatics* **13**, S15 (2012).
10. Altschul, S. F., Gish, W., Miller, W., Myers, E. W. & Lipman, D. J. Basic Local Alignment Search Tool. *J. Mol. Biol.* **215**, 403–410 (1990).

11. Morgulis, A., Gertz, E. M., Schäffer, A. A. & Agarwala, R. A Fast and Symmetric DUST Implementation to Mask Low-Complexity DNA Sequences. *J. Comput. Biol.* **13**, 1028–1040 (2006).
12. Church, D. M. *et al.* Modernizing Reference Genome Assemblies. *PLoS Biol.* **9**, e1001091 (2011).

13. ArchaicPlus captures

13.1 Data generation and processing

We selected a total of 43 libraries from 19 skeletal elements for nuclear DNA enrichment through hybridisation captures. The laboratory workflow was the same as the one described in Supplementary Sections 10 and 11, except for the probes, for which we employed the nuclear capture array described in Section 12. The enriched libraries were sequenced on Illumina's HiSeq4000 and NextSeq platforms.

We de-multiplexed libraries by identifying perfect matches of the expected pairs of indices for each library, and filtered the data for a minimum read-length of 35 bp and mapping quality of 25, restricting our analysis to the 2,284,453 target sites (Supplementary Data Table 5.1). To prevent reference bias at the target sites, we aligned the sequenced fragments to two different versions of the human genome. First, we aligned the data to the human reference genome GRCh37 or hg19[1]. All reads mapping these data were then further aligned to a modified version that included the alternative archaic allele, as described in [2], and only reads aligning to both reference genomes in the same location were retained. Summary statistics of the resulting data can be found in Supplementary Data Table 5.2.

13.2 Data curation

We used two different methods for estimating present-day human DNA contamination. The first approach relied on modeling the frequency of observed derived alleles as a linear combination of the proportion of endogenous and exogenous sequences. Knowing the expected values of the derived allele frequency in modern humans and for Neandertals in a given ascertainment, it is possible to infer the proportion of contaminant sequences[2,3]. We used a present-day European (S_French-1) from the Simons Genome Diversity Project[4] and the Chagyrskaya 8 Neandertal[5], with an average derived frequency of 37.7% and 0.05%, respectively. Then, assuming a binomial distribution, we obtained the 95% binomial confidence intervals.

We further used AuthentiCT, a Hidden Markov Model based on the deamination patterns of the sequences as signals of ancient damage, as a second contamination estimate for all non-UDG treated libraries [6].

We focused on the data mapped to the hg19 reference genome to obtain present-day contamination estimates. Since the expectation is that more contaminant reads will align to the hg19 rather than to the modified reference genome, this analysis is more conservative. The estimates obtained by the two contamination estimation methods were for the most part consistent with each other, as reported in

Supplementary Figure 34 and Supplementary Data Tables 5.3 and 5.4. A total of 40 out of the 43 libraries had more than 2% present-day human DNA contamination, a conservative cut-off for ancient DNA studies [7,8]. Since this represents the majority of the libraries, we decided to limit our analyses to deaminated sequences only.

We filtered for deaminated sequences by only retaining sequences with at least one C-to-T substitution within the three terminal bases from each side of the sequence alignment. In the filtered dataset, 36 out of 43 libraries passed the 2% contamination threshold using the linear combination test. Of the seven libraries that did not pass our 2% threshold, we fully excluded Lib.I.1004 and Lib.I.1625 from downstream analyses, since we had less-contaminated data from other libraries from the same specimens. The remaining five libraries were from specimens that would otherwise not be represented at all in downstream analyses, and we thus retained them despite their high levels of present-day human DNA contamination. These are the libraries from the specimens Goyet C5-1 (library Lib.I.1291 with 7.8% contamination), Goyet Q119-2 (Lib.I.1292 with 6.3% contamination), Goyet Q376-25 (Lib.L.9393 with 9.3% contamination), Goyet 1424-3D (Lib.I.1611 with 20.2% contamination), and Goyet Q55-4 (Lib.I.1607 with 6.7% contamination).

In summary, as a result of the selection criteria described above, 41 libraries were included in the downstream nuclear capture analysis. Libraries originating from the same skeletal remains were further combined using *samtools merge* version 1.3.1[9] Supplementary Data Tables 5.5 and 5.6[9].

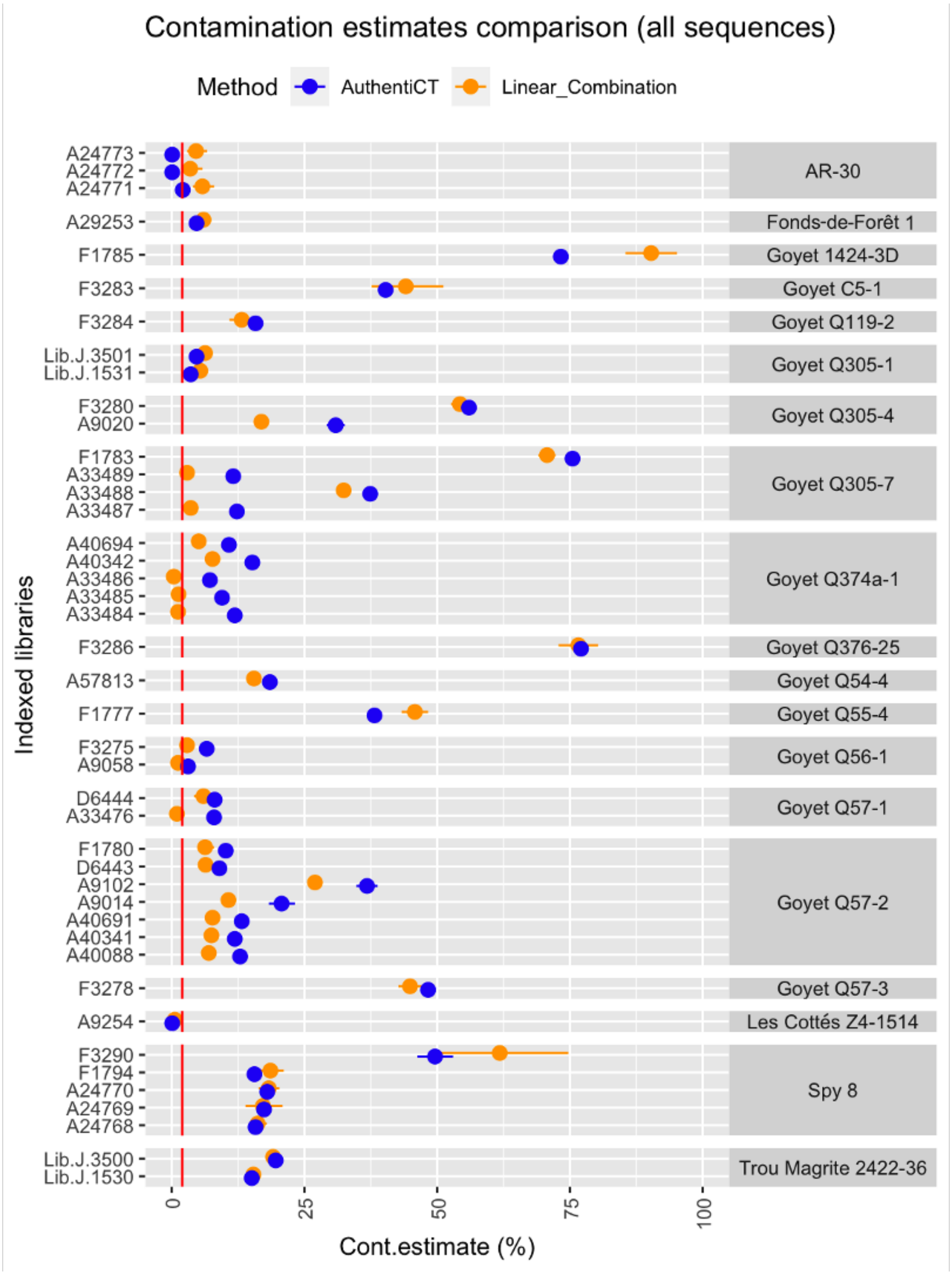

Supplementary Figure 34 Comparison of contamination estimates for each library. The red line indicates the 2% contamination threshold cut-off.

13.3 Genetic sexing

The ratio between the observed coverage on the sex chromosomes versus the autosomes is informative about the genetic sex of ancient individuals[10], including in capture data[11,3,12]. For the capture data, it is necessary to take into account the number of relevant target sites. For the ArchaicPlus array, there are 79,487 targeted SNPs on the X chromosome, 3,967 targeted SNPs on the Y chromosome and 2,200,999 targeted SNPs on the autosomes. By calculating the number of targets of sites on the sex chromosomes versus the autosomes, we estimated an expected "X-rate" of 0.0361 (79,487 / 2,200,999) and an expected "Y-rate" of 0.0018 (3,967 / 2,200,999). We used these expected rates to normalise the observed coverage rates for each sample. Samples with final X-ratios close to 1 were assigned as "females", while those close to 0.5 as "males". Similarly, samples with final Y-ratios close to 0 were assigned as "females", while those close to 0.5 as "males".

The results can be found in Extended Data Fig. 1. The majority of the analysed samples stem from female individuals, except for the bones of Fonds-de-Forêt 1, Spy 8, Spy 94a, Goyet Q305-1, Goyet 1424-3D and Trou Magrite 2422-36, which originate from male individuals. We note that Goyet Q56-1, Les Cottés Z4-1514 and Spy 94a had previously been genetically sexed as two females and one male, respectively[13], and we obtained consistent results. As a result of their low coverage and their high-contamination levels, we could not assign a genetic sex to Goyet Q119-2 and Goyet C5-1 (indicated in red in Extended Data Fig. 1).

References

1. Church, D. M. *et al.* Modernizing Reference Genome Assemblies. *PLoS Biol.* **9**, e1001091 (2011).
2. Peyrégne, S. *et al.* Nuclear DNA from two early Neandertals reveals 80,000 years of genetic continuity in Europe. *Sci. Adv.* **5**, eaaw5873 (2019).
3. Skov, L. *et al.* Genetic insights into the social organization of Neanderthals. *Nature* **610**, 519–525 (2022).
4. Mallick, S. *et al.* The Simons Genome Diversity Project: 300 genomes from 142 diverse populations. *Nature* **538**, 201–206 (2016).
5. Mafessoni, F. *et al.* A high-coverage Neandertal genome from Chagyrskaya Cave. *Proc. Natl. Acad. Sci.* **117**, 15132–15136 (2020).
6. Peyrégne, S. & Peter, B. M. AuthentiCT: a model of ancient DNA damage to estimate the proportion of present-day DNA contamination. *Genome Biol.* **21**, 246 (2020).
7. Allentoft, M. E. *et al.* Population genomics of Bronze Age Eurasia. *Nature* **522**, 167–172 (2015).
8. Nakatsuka, N. *et al.* ContamLD: estimation of ancient nuclear DNA contamination using breakdown of linkage disequilibrium. *Genome Biol.* **21**, 199 (2020).

9. Li, H. *et al.* The Sequence Alignment/Map format and SAMtools. *Bioinformatics* **25**, 2078–2079 (2009).
10. Skoglund, P., Storå, J., Götherström, A. & Jakobsson, M. Accurate sex identification of ancient human remains using DNA shotgun sequencing. *J. Archaeol. Sci.* **40**, 4477–4482 (2013).
11. Fu, Q. *et al.* The genetic history of Ice Age Europe. *Nature* **534**, 200–205 (2016).
12. Essel, E. *et al.* Ancient human DNA recovered from a Palaeolithic pendant. *Nature* **618**, 328–332 (2023).
13. Hajdinjak, M. *et al.* Reconstructing the genetic history of late Neanderthals. *Nature* **555**, 652–656 (2018).

14. Genetic kinship

A common obstacle in Palaeolithic archaeology is to determine which skeletal remains might stem from the same individual. This is especially challenging for the specimens from the Troisième caverne of Goyet, as the recovered bones are highly fragmentary due to anthropogenic activities[1]. Approximately half of these fragments could be refitted to each other based on morphological analyses. Here, we demonstrate how ancient DNA can provide a complementary approach in identifying bones from the same individual, and potentially also identify genetically closely related individuals.

We used KIN, a genetic-relatedness inference method bespoke for ancient DNA[2] that is able to detect up to third-degree relatives. The main advantages of KIN are that it is robust to genetic ascertainment, explicitly models present-day DNA contamination, and is tailored towards low-coverage ancient DNA. We used the curated capture dataset described in Supplementary Section 13 as input, consisting of libraries filtered to include deaminated reads only. Since typical ancient DNA damage could affect the results, we applied strand-specific filtering to our data. For C/T variants, only reverse-strand reads are considered, similarly, for G/A variants, only forward-strand reads are retained. After this strand-filtering, we re-estimated the contamination estimates using the linear combination method described in Supplementary Section 13, and used the resulting point estimates of contamination to run KIN.

KIN first estimates the average pairwise difference between unrelated individuals from the population (p_0), which is used to calibrate the analysis. Following prior recommendations[3], we estimated p_0 from the higher-quality samples, i.e., those with contamination levels under 1% and with at least 260,000 SNPs covered. This yielded an estimate of $p_0 = 0.0554$. Windows that were too noisy or otherwise low quality in this analysis were also discarded from further analysis.

Having defined these case-specific parameters, we then ran KIN with the rest of the samples that met KIN's model assumption of scarce contamination (i.e., below 5%). This meant that for five samples with contamination rates above 5% (Goyet C5-1, Goyet 1424-3D, Goyet Q376-25, Goyet Q55-4 and Goyet Q119-2) it was not possible to gain any insights with similar KIN analyses, as they violate basic assumptions from the model. We applied a log likelihood ratio of 1.0 as a threshold for significance. Besides captured data, we also included low-coverage shotgun data for Les Cottés Z4-1514 and Spy 94a, as well as high-coverage data for Goyet Q56-1. These genomes were all ascertained to the capture target sites. The results for this analysis are reported in the Supplementary Figure 35. Using this approach, we identified three clusters of skeletal remains belonging to three distinct individuals: (1) Spy 8 and Spy 94a, assigned to Spy 94a_8 (2) Goyet Q56-1, Goyet Q374a-1 and Goyet Q305-7,

assigned to GN1, and (3) Goyet Q57-1, Goyet Q57-2 and Goyet Q57-3, assigned to GN2. These three individuals were not related to each other, to the limits of our resolution.

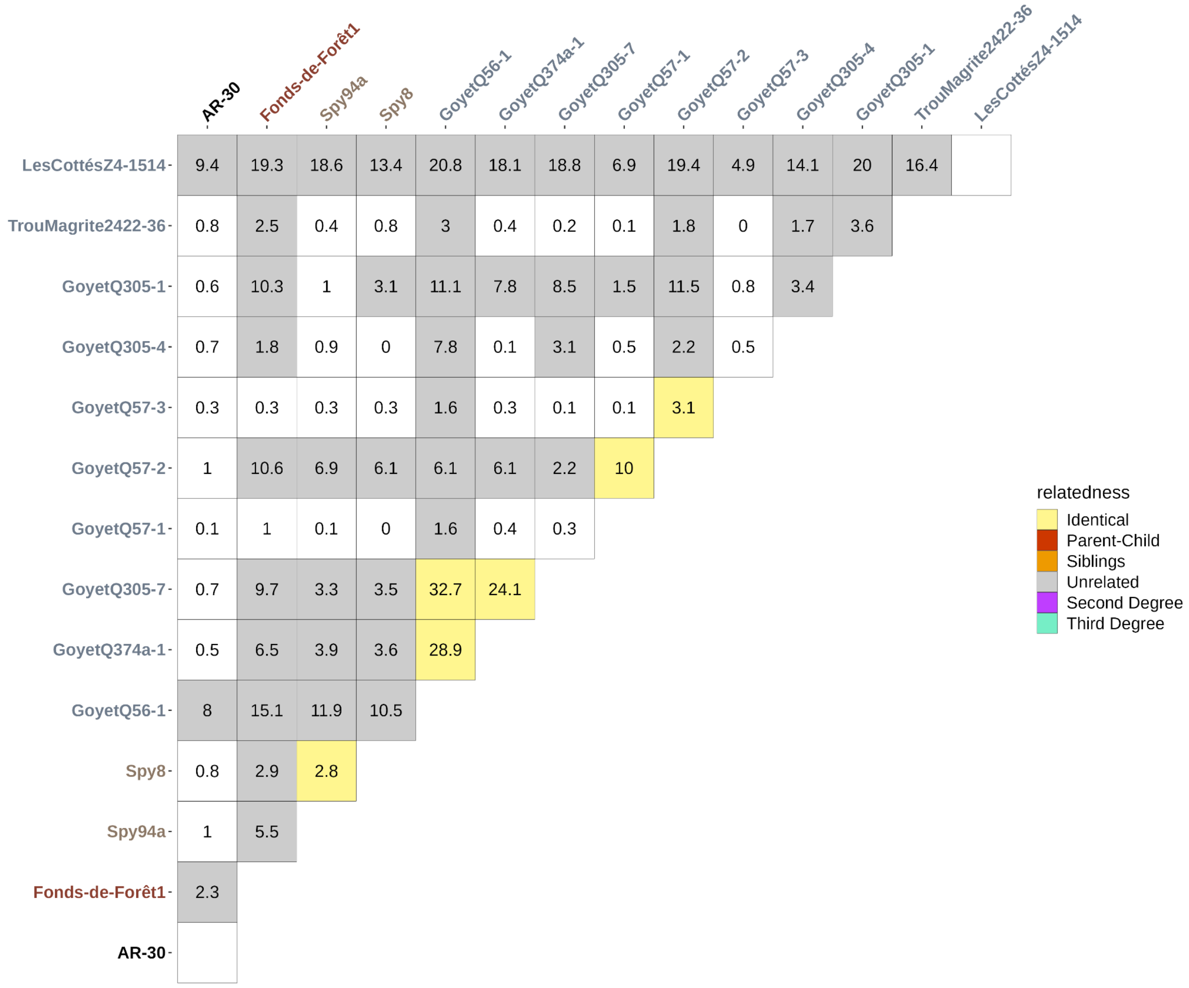

Supplementary Figure 35 Kinship estimates by KIN for the samples with <5% contamination. Coloured cells indicate the degree of relatedness. Uncertain kinships with log likelihoods lower than 1 are depicted in white. Sample labels are coloured by archaeological sites.

The fact that Goyet Q305-7 and Goyet Q374a-1 were identified as identical to each other was initially unexpected. It implied that the bones either originated from the same individual, or from monozygotic twins. Given that these samples had been described as two right tibias that overlap in the area of the soleal line (Tibia III and Tibia V, respectively, as described in Rougier et al.[1]), the genetic results would thus indicate the presence of monozygotic twins in this archaeological assemblage.

To validate these results, a thorough re-examination of the re-fitting was conducted. This analysis identified that the fragment Goyet Q305-7, which was originally sampled as representative of Tibia III, had been wrongly re-fitted to that element. To explore this possibility further, we took a new sample

from Tibia III, this time from its lowermost part of the fragment Goyet Q54-4 (Supplementary Figure 36).

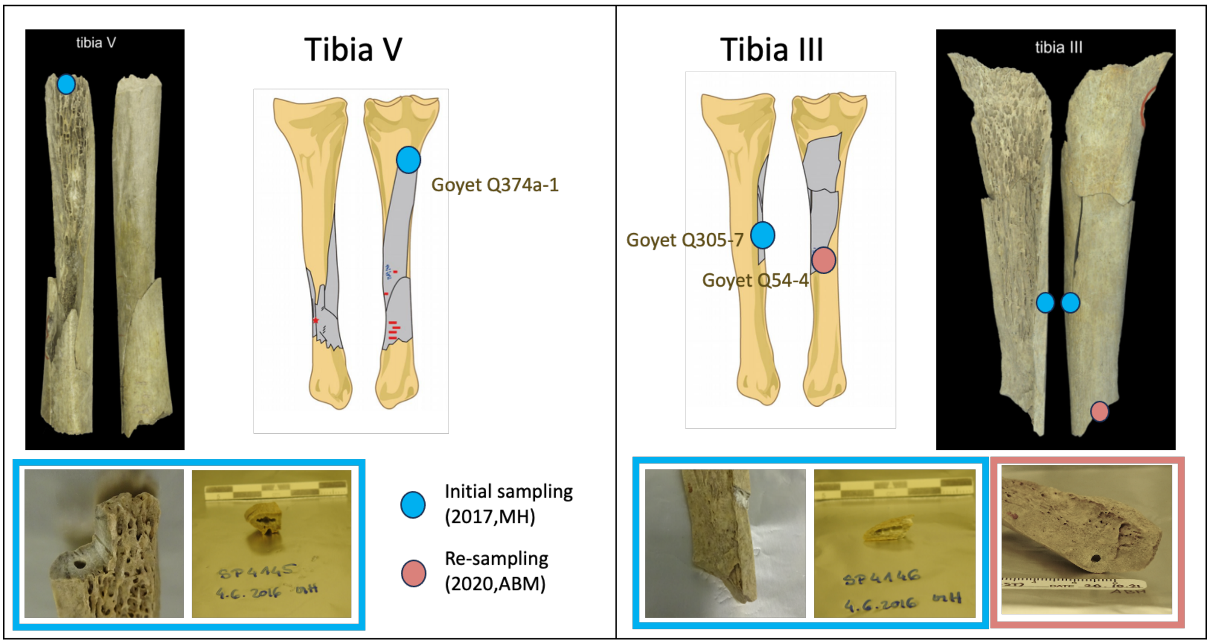

Supplementary Figure 36 Sampling locations on Tibia V and Tibia III. The colours indicate the sampling rounds. Diagrams and refitting pictures modified from Rougier et al., 2016, originally taken by E. De Wamme (Copyright RBINS).

By incorporating the genetic data of Goyet Q54-4 into the KIN analyses, we were able to verify that Goyet Q374a-1 and Goyet Q54-4 indeed belonged to genetically distinct individuals (Supplementary Figure 37). In fact, Goyet Q54-4 (a fragment of right Tibia III) was identical to Goyet Q57-1 (left Tibia II), Goyet Q57-2 (right Femur II), and by extension, to Goyet Q57-3 (which could be identified as identical to Goyet Q57-2 but not to the rest of the remains due to the low coverage of this sample). Specifically, Goyet Q57-3 represents a fragment of right tibia (initially numbered Tibia VI). However, the recovered parts of right Tibias III and VI do not overlap, so it is most parsimonious to propose that they belong to the same individual, and Goyet Q57-3 has been re-assigned to Tibia III along with Goyet Q54-4. In summary, the genetic and morphological results both support that Goyet Q57-1, Goyet Q57-2, Goyet Q57-3 and Goyet Q54-4 all originate from the same individual, named GN2.

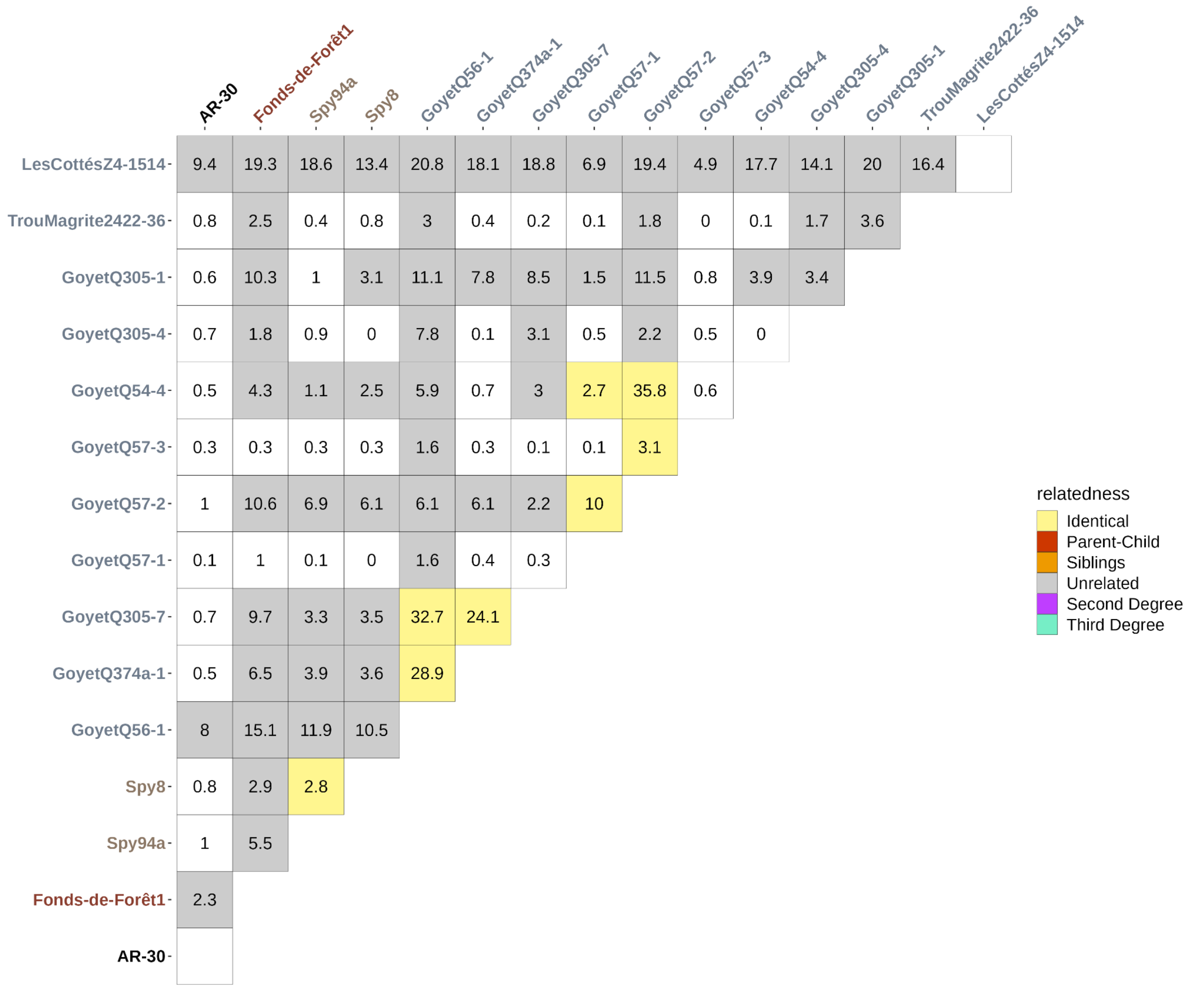

Supplementary Figure 37 Kinship estimated by KIN for the samples with <5% contamination, including Goyet Q54-4. Coloured cells indicate the degree of relatedness. Uncertain kinships with log likelihoods lower than 1 are depicted in white. Sample labels are coloured by archaeological sites.

Given these results, we merged the data from identical individuals and repeated the KIN analyses. This further confirmed that all the individuals identified to date do not appear to be closely related (Supplementary Figure 38).

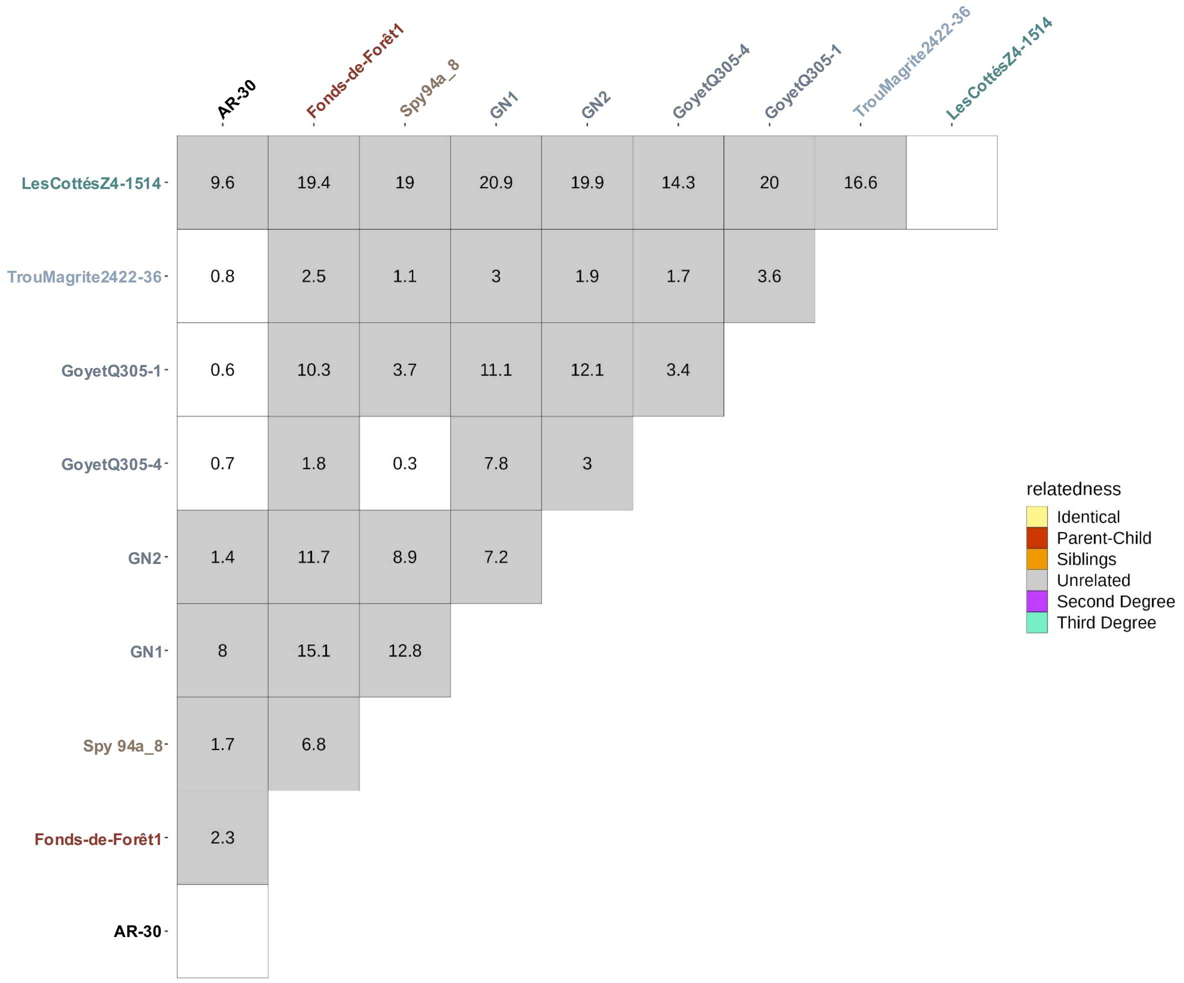

Supplementary Figure 38 Kinship estimated by KIN for the samples with <5% contamination, after merging data from identical individuals. Coloured cells indicate the degree of relatedness. Uncertain kinships with log likelihoods lower than 1 are depicted in white. Sample labels are coloured by archaeological sites.

Based on the morphological analyses of the bones and the mtDNA results, a minimum of five Neandertal individuals had previously been identified in the Goyet collection[1]. In this study, we increased this number to a minimum of six individuals. The first two are defined by their relative age-at-death, i.e., the incisor of a child Goyet 1424-3D and a neonate femur Goyet Q305-1. They represent the only males that we were able to genetically identify in the collection. Besides these, the nuclear kinship analyses confirmed that the three right tibias that overlap in the area of the soleal line and have different mitochondrial haplogroups (Tibia V, represented here by Goyet Q374a-1; Tibia III, represented here by Goyet Q54-4; and Tibia IV, represented here by Goyet Q55-4) all belong to distinct, unrelated individuals. Goyet Q305-4, Tibia I, represents the final distinct individual.

Taking into consideration that the individuals under study originate from localities in very close geographical proximity and broadly contemporaneous contexts, especially for those from the Mosan Basin (i.e., Goyet, Spy, Trou Magrite and Fonds-de-Forêt), it would have been conceivable to anticipate close genetic connections amongst the inhabitants of the region. However, our findings indicate that this is not the case for at least up to a third-degree genetic kinship.

References

1. Rougier, H. *et al.* Neandertal cannibalism and Neandertal bones used as tools in Northern Europe. *Sci. Rep.* **6**, 29005 (2016).
2. Popli, D., Peyrégne, S. & Peter, B. M. KIN: a method to infer relatedness from low-coverage ancient DNA. *Genome Biol.* **24**, 10 (2023).
3. Sümer, A. P. *et al.* Earliest modern human genomes constrain timing of Neanderthal admixture. *Nature* **638**, 711–717 (2025).

15. Population affinities

15.1 Relationships with other Neandertals

We placed the studied Neandertals from Arcy-sur-Cure, Les Cottés, Fonds-de-Forêt, Goyet, Spy and Trou Magrite within the known archaic variation. Since their genetic data were generated using a capture array ascertained on the Denisovan D3[1] and three high coverage Neandertals – D5 Neandertal[2], Chagyrskaya 8[3] and Vindija 33.19[4] – we evaluated how the ascertainment bias might affect our downstream results[5].

To address this, we performed a series of quality controls using low-coverage and ascertainment-free data. Specifically, we analysed the shotgun data of five Late Neandertals and Mezmaiskaya 1[4,6], as well as the whole-genome capture data from the "Thorin" Neandertal from Mandrin[7]. We further downsampled these genomes to the autosomal positions defined in the ArchaicPlus ascertainment as informative of archaic variation only, i.e., excluding sets of sites used for estimating present day contamination and positions on the sex chromosomes (N (final set of sites) = 1,652,202 SNPs, see Section 12). In this way, we could benchmark with the unfiltered data, and evaluate how the ArchaicPlus ascertainment of sites might affect downstream population genetic results, before extending the analyses to our set of captured samples.

D-statistics with ascertained samples

We explored the relationship between the archaic populations quantitatively, through a range of D-statistics[8]. Firstly, we focused on D-statistics that included the high-coverage individuals used to define the ArchaicPlus ascertainment. We evaluated *D(X1, X2; Y, Mbuti)*, where *X1* and *X2* were unique combinations of Denisova 3, D5, Chagyrskaya 8 and Vindija 33.19 (i.e., the archaic individuals used for ascertainment), *Y* was one of the low-coverage Neandertal genomes, and *Mbuti* was a present-day Mbuti individual from the Human Genome Diversity Project (HGDP00982)[1].

We genotyped all of the high-coverage genomes using snpAD (version 3.11)[9], combined them into a single vcf file using bcftools (version 1.4), and converted it to the eigenstrat format using an in-house perl script. For the low-coverage Neandertals, to make their data more comparable to the newly generated data in this study, we used only deaminated sequences and applied strand-filtering to limit the effect of post-mortem damage. We performed random-read sampling to call their genotypes, and relied on another in-house script to transform it to eigenstrat format. We computed all of the D-statistics using *admixr* (version 0.9.1)[10], which relies on ADMIXTOOLS version 7.0[11]. We gauged significance

based on the absolute Z-score values, using the conservative threshold of Z-score values larger than 3[12–14].

We find the results of unascertained and ascertained data to be highly consistent with each other, with an overall correlation of 0.999 between the D-statistics calculated on the two data sets (Supplementary Figure 39).

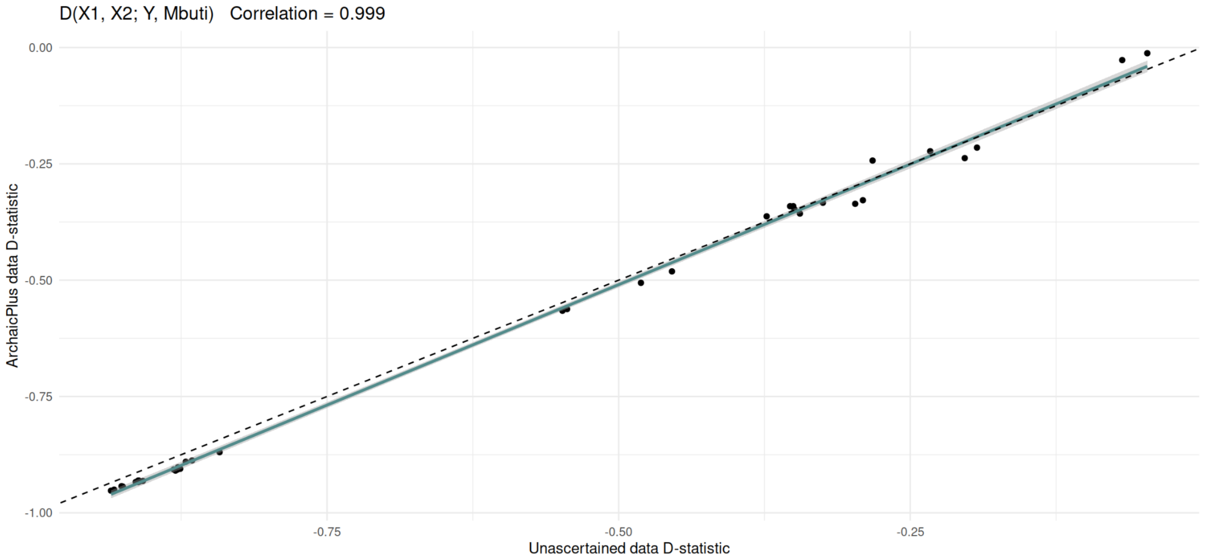

Supplementary Figure 39 Correlation of the values of *D(X1, X2; Y, Mbuti)*, where *X1* and *X2* represent a unique combination of Denisova 3, D5, Chagyrskaya 8 and Vindija 33.19, *Y* is a low-coverage Neandertal genome, and *Mbuti* is the present-day individual used as an outgroup. The dotted line denotes a perfect correspondence between the two, and the full line shows the fitted correlation with uncertainty.

Since we found no evidence for ascertainment bias in these D-statistics where we compare the low-coverage genomes "*Y*" to the ascertainment references, we performed this analysis using the newly generated ArchaicPlus data. We used the data described in Section 14, i.e., curated bam files that had been strand-filtered and merged according to the distinct individuals identified during the kinship analysis.

All the following results presented in this section have been compiled in Supplementary Data Table 7. We find that all low-coverage Neandertals are closer to the Vindija 33.19 Neandertal than the ~120,000-year-old D5 Neandertal, and only Thorin, Mezmaiskaya 1 and Goyet C5-1 are not significantly closer to the ~45,000-year-old Vindija 33.19 than they are to the ~70,000-year-old Chagyrskaya 8 (Supplementary Figure 40). While the first two cases are consistent with the previous results obtained

for these Neandertals [3,7], the latter result is likely affected by the high contamination levels and low coverage of Goyet C5-1, as detailed in Section 13.

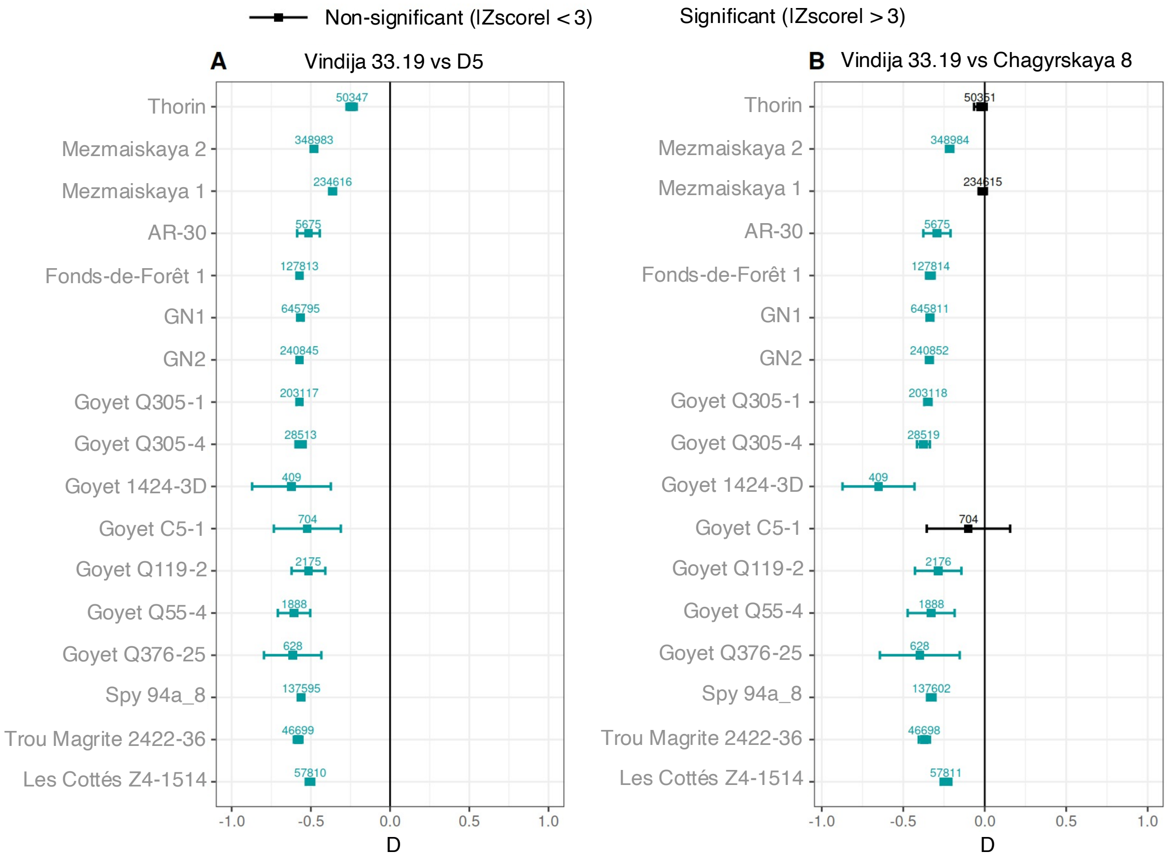

Supplementary Figure 40 D-statistics of the form *D(X, Vindija 33.19; Y, Mbuti)*, where "*X*" represents either the D5 Neandertal (**A**) or Chagyrskaya 8 (**B**), and "*Y*" represents the Neandertals on the Y-axis. The errors around the mean (± standard error) of the D-statistics are indicated, coloured depending on whether the associated Z-score reached the absolute threshold of 3 for significance. The numbers above the error bars indicate the number of SNPs used for each specific comparison (N = 1,652,202 SNPs).

Therefore, we conclude that all the newly analysed Neandertals in this paper appear to be closest to the Vindija 33.19 Neandertal.

Principal component analysis with ascertained samples

We also explored the genetic affinities of the samples of interest relative to other archaic humans through a Principal Component Analysis (PCA)[15,16]. We used the dataset as for the D-statistics described above. First, we defined the variation focusing on the genomes of Denisova 3, a present-day Mbuti individual from the Human Genome Diversity Project (HGDP00982)[1] and the three high-coverage Neandertal genomes from Vindija 33.19, D5 and Chagyrskaya 8. We projected the low-coverage

samples onto the principal components generated from the reference individuals. All PCA analyses were performed using smartpca[15].

As expected from the close correspondence between PCA and D-statistics[17], the results mirror those of the D-statistics, in that most of the focus samples of our study are all closer to Vindija 33.19 (Supplementary Figure 41). The low-coverage samples cluster closely with Neandertals, but we observe a gradient towards Mbuti-related ancestry that is concordant with the levels of present-day human contamination we found in the samples. For example, the most-contaminated samples, Goyet C5-1, Goyet 1424-3D and Goyet Q119-2, appear closest to Mbuti on both principal components 1 and 2.

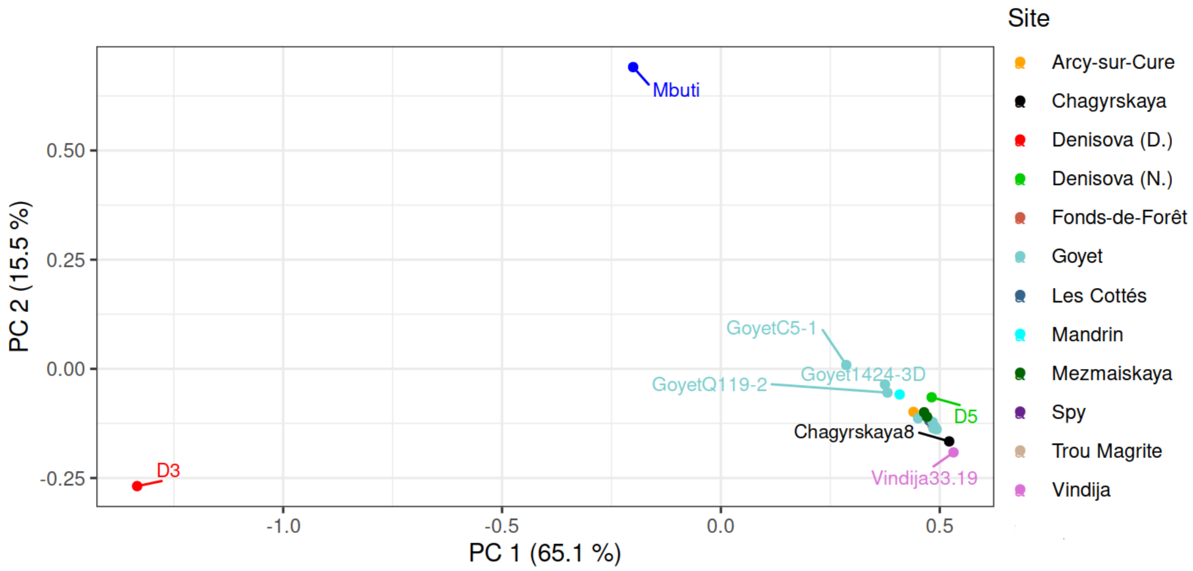

Supplementary Figure 41 PCA defined by the variation between Denisovans, Neandertals and modern humans (N = 1,652,202 SNPs).

Based on these results, as well as the ones detailed in the previous section, we proceeded to use the D5 Neandertal as an outgroup for all our downstream analyses to reduce noise[18].

D-statistics with unascertained individuals

We further zoomed in and explored the variation and relationships within Late Neandertals. Specifically, we wanted to determine whether the individuals reported in our study are more closely related to the Vindija 33.19 or the GN1 Neandertals. This requires computing D-statistics that compare Neandertals from the ascertainment panel (Vindija 33.19) with newly sequenced Neandertals that were not included in the ascertainment (GN1). This differential relationship to the ascertained sites will result in an ascertainment bias if not handled carefully, and thus we prefaced our analysis with a number of validation steps.

We compared unascertained whole-genome Neandertal data to a subset restricted to the ArchaicPlus positions only. Compared to unascertained data, all of the individuals showed a biased attraction towards Vindija 33.19 after restricting the analyses to the ArchaicPlus positions only, shifting the observed population affinities from the unascertained data significantly (Supplementary Figure 42).

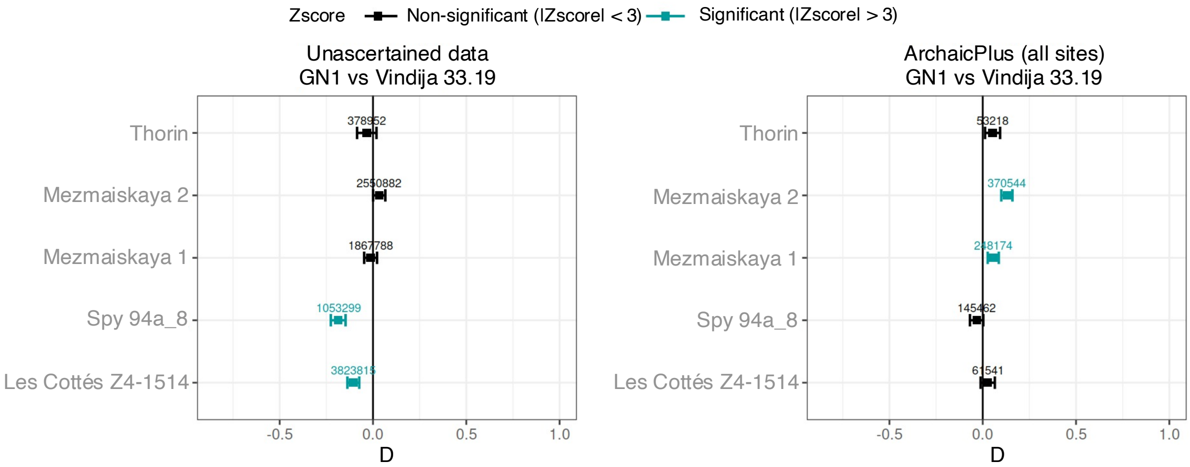

Supplementary Figure 42 D-statistics of the form *D(Vindija 33.19, GN1; Y, D5)*, where "*Y*" represents the Neandertals on the Y-axis. The errors around the mean (± standard error) of the D-statistics are indicated, coloured depending on whether the associated Z-score reached the absolute threshold of 3 for significance. The numbers above the error bars indicate the number of SNPs used for each specific comparison (unascertained set on the left: N = 16,715,517 SNPs; ArchaicPlus ascertained set on the right: N = 1,652,202 SNPs).

We found that this bias can be corrected by removing sites that were private to the Vindija 33.19 Neandertal from the ArchaicPlus ascertainment ("noVind" to abbreviate, N = 1,485,363 SNPs, Supplementary Figure 43A).

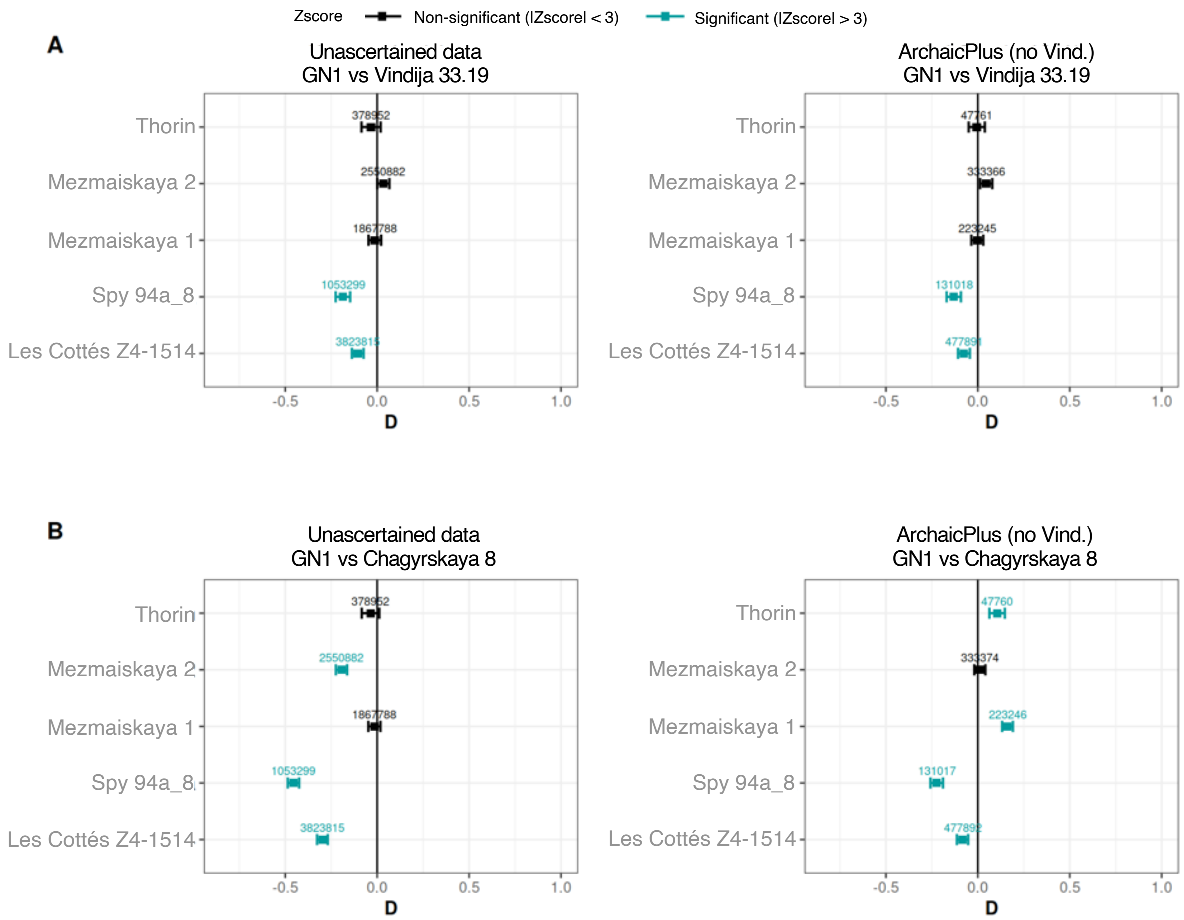

Supplementary Figure 43 D-statistics of the form *D(X, GN1; Y, D5)*, where "*X*" represents either Vindija 33.19 (facet A, on top) or Chagyrskaya 8 (facet B, on the bottom), and "*Y*" represents the Neandertals on the Y-axis. The plots on the left represent unascertained data, and on the right ArchaicPlus ascertained data without Vindija private sites. The errors around the mean (± standard error) of the D-statistics are indicated, coloured depending on whether the associated Z-score reached the absolute threshold of 3 for significance. The numbers above the error bars indicate the number of SNPs used for each specific comparison (N = 1,485,363 SNPs).

Furthermore, we found the same issue for other Neandertals, such as Chagyrskaya 8. When comparing the genetic affinity of ascertained individuals to the Chagyrskaya 8 Neandertal, we found an attraction to Chagyrskaya 8 (Supplementary Figure 43B), unless we remove Chagyrskaya-private sites. Since we also want to incorporate the Chagyrskaya 8 Neandertal into further analyses, we decided to revise the ascertainment bias correction to also exclude all sites derived in the Vindija 33.19 and/or Chagyrskaya 8 branch ("noChagVind" to abbreviate, N = 1,310,536 SNPs). This allowed us to effectively replicate the shotgun results regarding the relationships of Late Neandertals and Mezmaiskaya 1 with GN1, Vindija 33.19 and Chagyrskaya 8 while using D5 as an outgroup (Supplementary Figure 44).

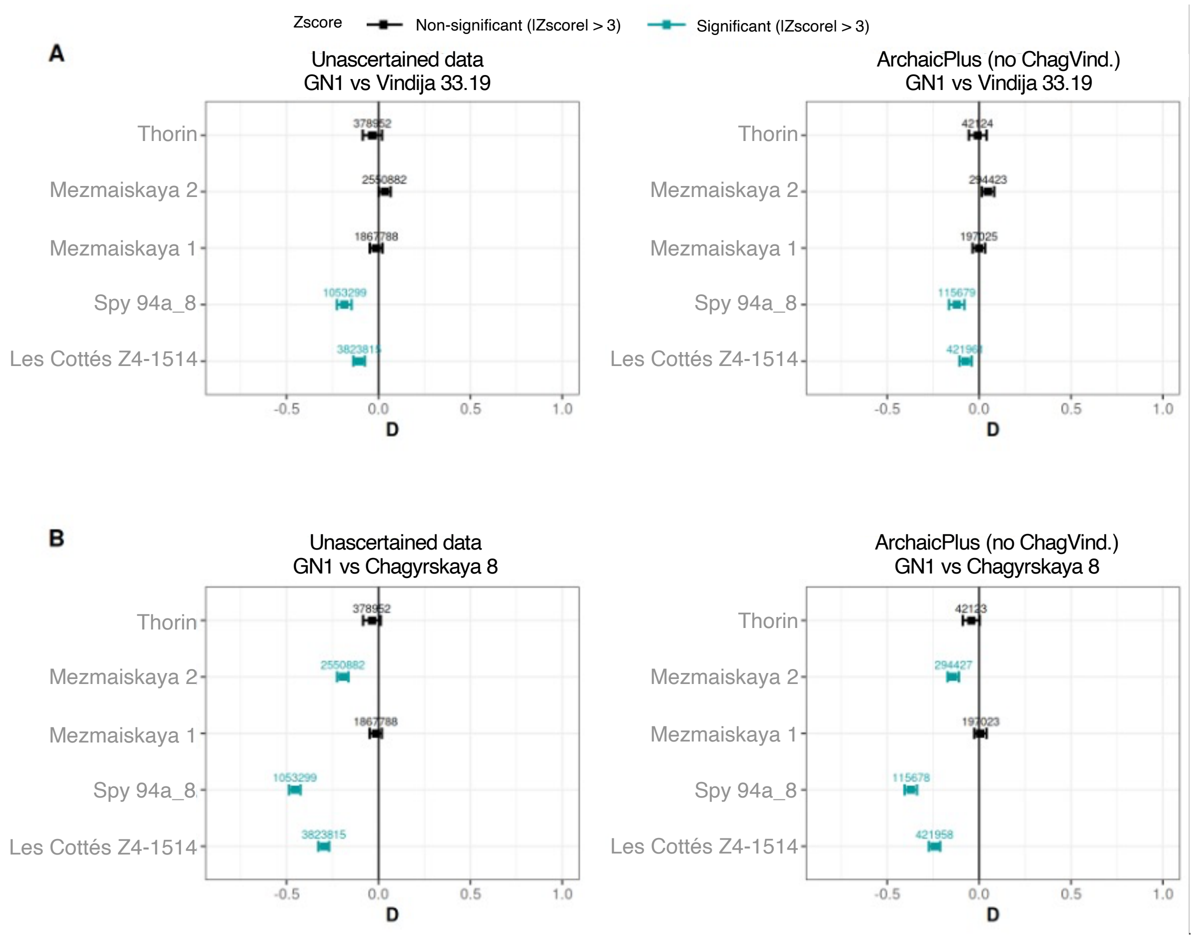

Supplementary Figure 44 D-statistics of the form *D(X, GN1; Y, D5)*, where "*X*" represents either Vindija 33.19 (facet A) or Chagyrskaya 8 (facet B), and "*Y*" represents the Neandertals on the Y-axis. The plots on the left represent unascertained data, and on the right ArchaicPlus ascertained data without Vindija and/or Chagyrskaya private sites. The errors around the mean (± standard error) of the D-statistics are indicated, coloured depending on whether the associated Z-score reached the absolute threshold of 3 for significance. The numbers above the error bars indicate the number of SNPs used for each specific comparison (N = 1,310,536 SNPs).

Since we found that removing private sites for calculating D-statistics is sufficient to control for ascertainment bias, we used these ascertainments to study the relationship of the Late Neandertals to the high-coverage genomes (Section 14). We found that most Late Neandertals from Belgium and France are closer to GN1 than to Vindija 33.19. This includes the samples from Trou Magrite, Les Cottés, Fonds-de-Forêt, and most of the individuals from Goyet. Both Mezmaiskaya 1 and 2, as well as Thorin, do not show an affinity to either Neandertal, consistent with their previous placement within Neandertals[6,7]. Goyet 1424-3D, Goyet C5-1, Goyet Q119-2, Goyet Q55-4, Goyet 376-25, AR-30 and Goyet Q305-4 show no significant difference in their allele sharing with Vindija 33.19 and GN1 (Extended Data Fig. 4). These samples are all low coverage and/or have high levels of present-day

human DNA contamination (see Supplementary Section 13). In particular, for Goyet Q305-4, the Z-score for *D(Vindija 33.19, GN1; Goyet Q305-4, D5)* is -2.97, just under the significance threshold of |Z-score| > 3, and suggesting an affinity towards GN1. In contrast, the Z-score for *D(Vindija 33.19, GN1; AR-30, D5)* is -1.19, which would not pass even less stringent criteria such as |Z-score| > 2. Our interpretation is that her relation to other Late Neandertals might be more nuanced, but more data will be needed to resolve this. Overall, we conclude that the majority of the individuals reported in this study are significantly more closely related to GN1.

Principal component analysis with unascertained individuals

We performed a PCA in the same way described previously, with an important difference. We used the genomes of Chagyrskaya 8, Vindija 33.19, and GN1 to define the underlying variation, and because GN1 is not included in the ArchaicPlus design, this forced us to restrict the analysis to the "noChagVind" set of sites (N = 1,310,536 SNPs) to avoid the ascertainment bias.

The results are summarised in Extended Data Fig. 5. The first principal component separates the D5 Neandertal from the rest. Similarly, the second component separates Eastern and Western Neandertals, and the third separates Vindija 33.19 and GN1. Based on this, we note that all of the focus samples cluster close to the high-coverage Late Neandertals like Vindija 33.19 and GN1. All of the samples fall closer to the latter, except for the two previously published low-coverage genomes from Mezmaiskaya which appear closer to Vindija 33.19, consistent with the results of Hajdinjak et al.[6]. Thorin, as reported by Slimak et al.[7] is an outgroup to both, and therefore shows no differential affinity towards either Vindija 33.19 or GN1.

15.2 Relationships within the focal Neandertals

F3-statistics

We used outgroup f3-statistics to gain insights into possible genetic clustering within the analysed individuals, which we calculated using the *qp3Pop* function in admixtools (version 2.0.4, https://uqrmaie1.github.io/admixtools/index.html). We used the D5 Neandertal as the outgroup, and restricted our analyses to 1,652,202 SNPs informative positions from the ArchaicPlus array that reduce the ascertainment bias as described above. In the case of the high-coverage genomes, we first downsampled them to an average coverage of ~1x to avoid any additional biases introduced due to differences in data quality. Moreover, to rule out potential ascertainment biases, we used the same strategy as in the previous sections and compared the values of *f3("X", "Y"; D5)* for the unique X/Y

Neandertal combinations using shotgun data before and after ascertaining to the ArchaicPlus sites only (Extended Data Fig. 6).

After verifying that these two quality control cases were comparable, we decided to calculate the outgroup f3-statistics on our dataset. For this analysis, we required at least 5,000 positions to be covered, which excluded AR-30, Goyet 1424-3D, Goyet C5-1, Goyet Q119-2, Goyet Q55-4, and Goyet 376-25 (Supplementary Table 34). Similarly, we removed pairwise estimates with less than a thousand overlapping SNPs.

Supplementary Table 34 Breadth of called positions for each ancient genome. Samples shaded in gray do not pass the required threshold of 5,000 SNPs covered.

Sample	SNP covered	Sample	SNP covered
Goyet 1424-3D	353	Spy 94a_8	138,758
Goyet Q376-25	557	Goyet Q305-1	172,939
Goyet C5-1	583	Mezmaiskaya 1	197,067
Goyet Q55-4	1,619	GN-2	204,984
Goyet Q119-2	1,792	Mezmaiskaya 2	294,486
AR-30	4,744	Les Cottés Z4-1514	422,022
Goyet Q305-4	19,763	Chagyrskaya 8	487,371
Trou Magrite 2422-36	39,782	GN-1	543,641
Thorin	42,133	Vindija 33.19	571,112
Fonds-de-Forêt 1	108,291		

The results are reported in Supplementary Data Table 7 and Supplementary Figure 45. As reported in Slimak et al., 2024, the Thorin Neandertal represents an outgroup to the rest of the Late Neandertals. Then, consistent with previous findings[6], Mezmaiskaya 1 is an outgroup for most Late Neandertals, together with Chagyrskaya 8.

Notably, all of the individuals from Goyet, Trou Magrite, Spy and Fonds-de-Forêt shared the higher proportion of drift among each other, especially regarding the two latter individuals. However, when considering the variation associated with those estimates, Spy 94a and Fonds-de-Forêt 1 were not significantly closer to each other than they were to other individuals (Supplementary Figure 46). Similarly, while Goyet Q305-4 had the highest f3-statistic with the Les Cottés Z4-1514 Neandertal, the difference with other samples was not significant (Supplementary Figure 47), and spurious results could

further be exacerbated by the fact that Goyet Q305-4 is the sample with the lowest coverage (see Supplementary Table 34).

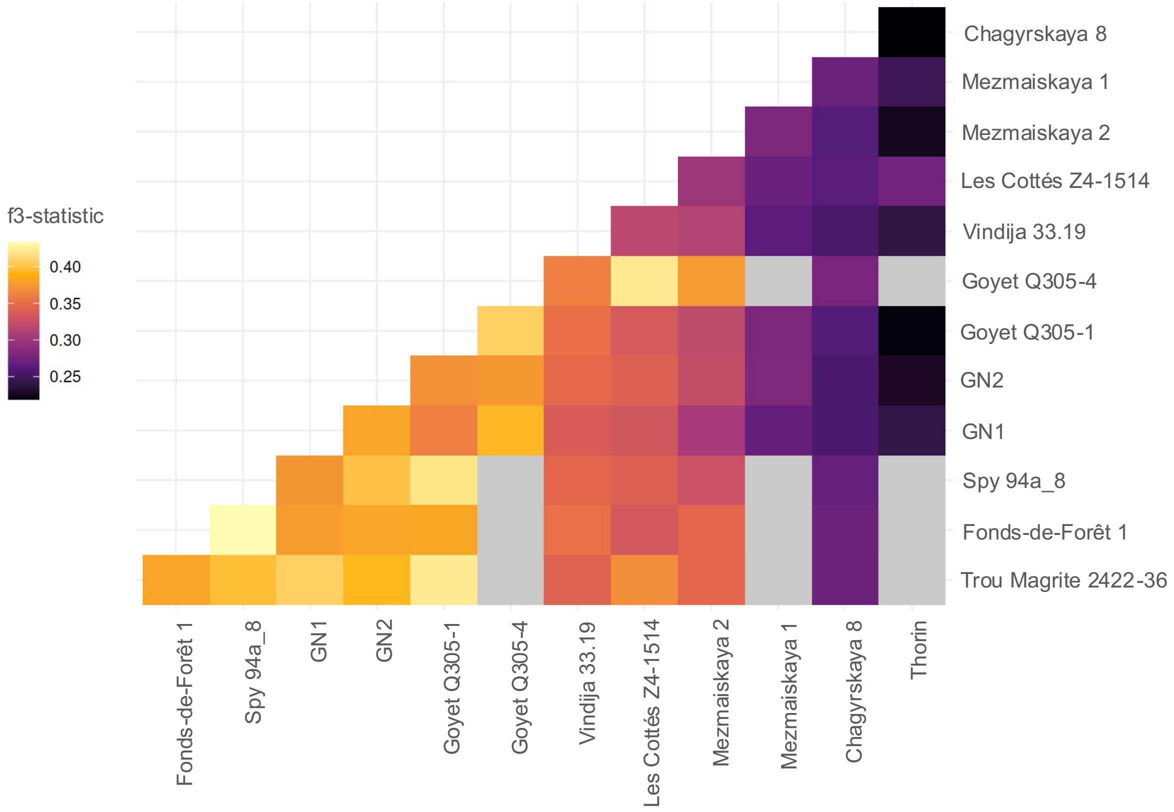

Supplementary Figure 45 Outgroup f3-statistics of the form *f3(X, Y; D5)*, where "*X*" represents the Neandertal on the X-axis and "*Y*" represents the Neandertal on the Y-axis. Statistics with fewer than 1,000 overlapping sites appear in grey.

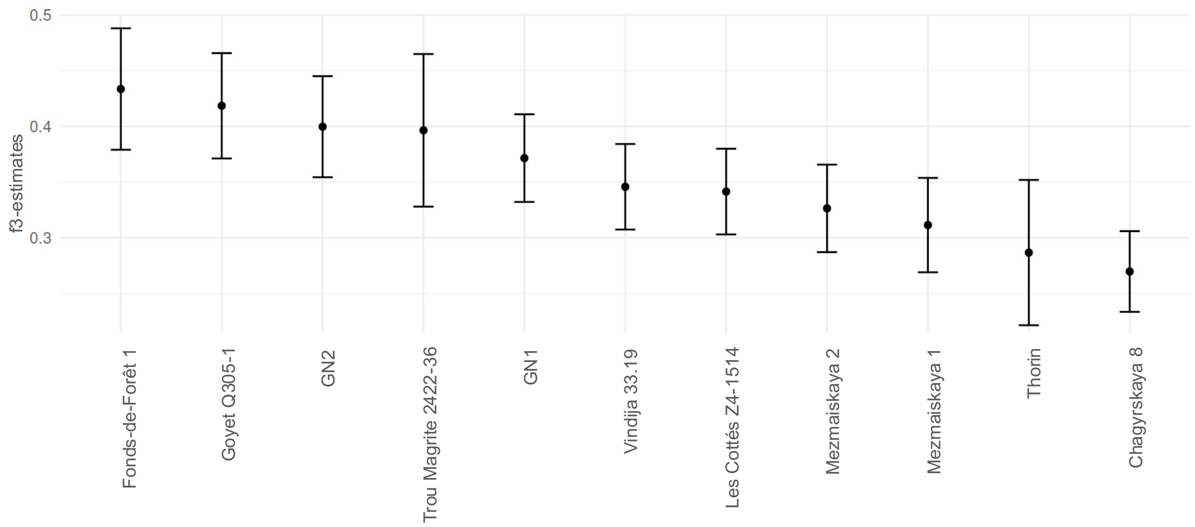

Supplementary Figure 46 Outgroup f3-statistics of the form *f3(X, Spy 94a_8; D5)*, where "*X*" represents the Neandertal on the X-axis, with their 95% confidence intervals.

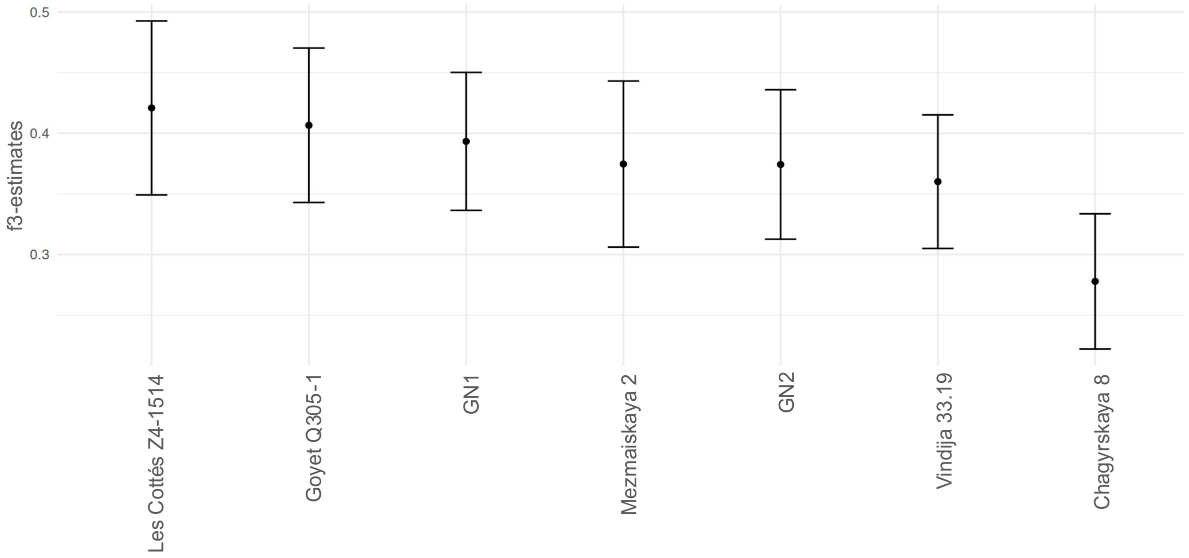

Supplementary Figure 47 Outgroup f3-statistics of the form *f3(X, Goyet Q305-4; D5)*, where "*X*" represents the Neandertal on the X-axis, with their 95% confidence intervals.

In summary, the f3-analysis supports the finding of the D-statistics that the Belgian Neandertals from Fonds-de-Forêt, Spy and Goyet are most closely related to each other, with the exception of Goyet Q305-4, for which we do not have sufficient data for a confident assignment.

D-statistics

To further study the relationship among the focal Neandertals of this study, we employed the statistic *D(X1, X2; X3, D5)*, where *X1, X2,* and *X3* represent three different Neandertals, using the same dataset as for the **f3-statistics section**, with the same threshold of at least 1,000 overlapping sites. We restricted analyses to combinations with at least 5 non-zero ABBA/BABA estimates (the minimum required in *admixtools*).

We used the same strategy as in the above sections to evaluate potential ascertainment biases, and by comparing the values of *D(X1, X2; X3, D5)* for the unique combinations, for the shotgun data before and after ascertaining to the ArchaicPlus sites only. The overall correlation is 0.834 between the D-statistics calculated on the two data sets (Supplementary Figure 48), with ascertained data resulting in overall lower absolute value D-statistics, as evidenced by the slope being less than one.

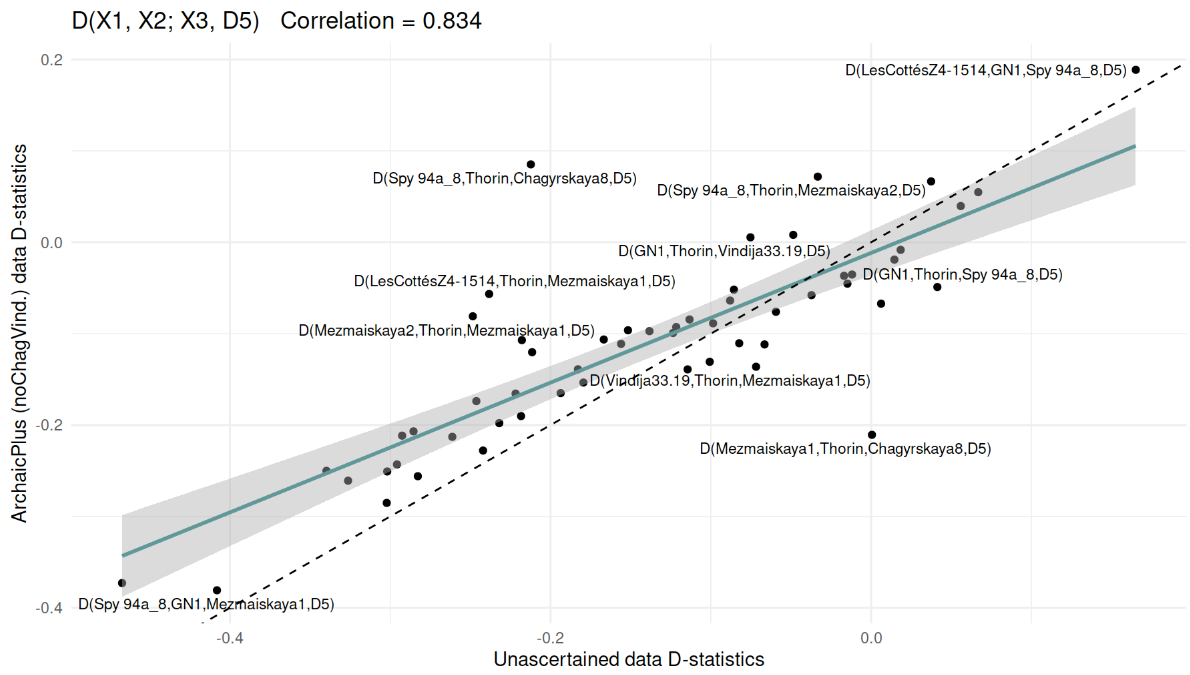

Supplementary Figure 48 Correlation of the values of *D(X1, X2; X3, D5)*, where *X1*, *X2* and *X3* represent a unique combination of Chagyrskaya 8, Thorin, Vindija 33.19, GN1, Spy 94a_8, Les Cottés Z4-1514, Mezmaiskaya 2 and Mezmaiskaya 1. The dashed line denotes a perfect correspondence between the two, and the full line shows the fitted correlation with uncertainty. The top 10 outliers are labelled.

We noted that Thorin was consistently an outlier, possibly due to the lower coverage relative to the rest of the samples, both in the ascertained and unascertained datasets (Supplementary Table 35).

Supplementary Table 35 Breadth of called positions for each ancient genome.

Sample	SNP covered (Unascertained)	SNP covered (Ascertained)
Thorin	378,952	42,133
Spy 94a_8	1,053,299	138,758
Mezmaiskaya 1	1,867,788	197,067
Mezmaiskaya 2	2,550,882	294,486
Les Cottés Z4-1514	3,823,815	422,022
Chagyrskaya 8	4,080,173	487,371
GN1	4,831,629	543,641
Vindija 33.19	5,151,671	571,112

For an analysis such as this, when three low-coverage samples are compared, it is important to take into account data quality because if a pair of samples have much higher coverage than the third sample, comparisons may be skewed (i.e., long-branch attraction). For this reason, we removed Thorin from these D-statistics, which as expected improved the correspondence between the ascertained and unascertained quality-control results (Supplementary Figure 49), and interpreted with special caution significant results between samples of differing quality.

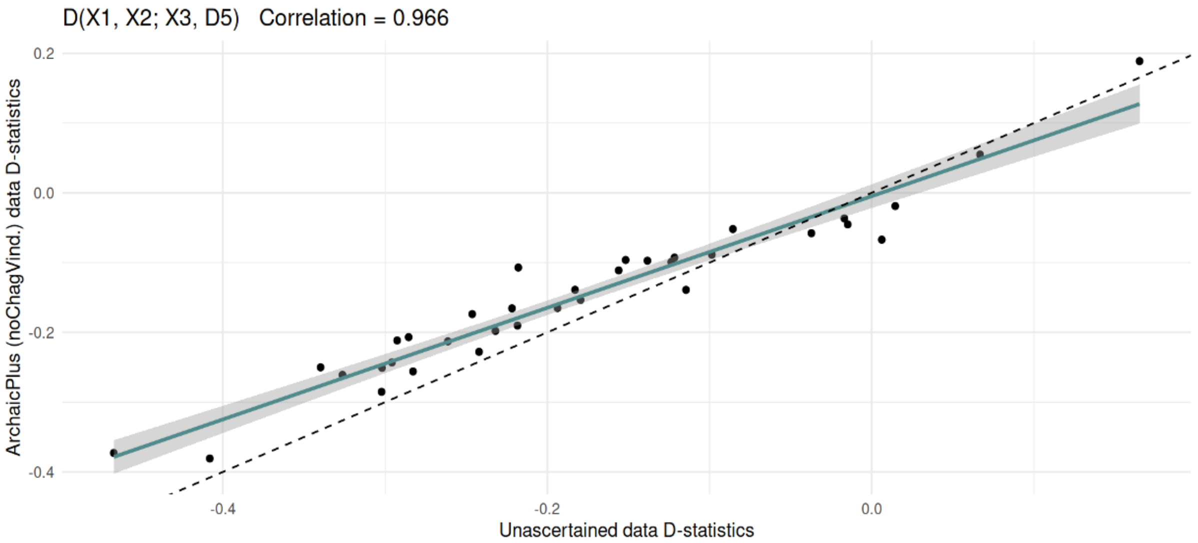

Supplementary Figure 49 Correlation of the values of *D(X1, X2; X3, D5)*, where *X1*, *X2* and *X3* represent a unique combination of Chagyrskaya 8, Vindija 33.19, GN1, Spy 94a_8, Les Cottés Z4-1514, Mezmaiskaya 2 and Mezmaiskaya 1. The dotted line denotes a perfect correspondence between the two, and the full line shows the fitted correlation with uncertainty.

In general, the results are consistent with the findings of the outgroup f3-statistics: Mezmaiskaya 1 is an outgroup to most Late Neandertals (Supplementary Figure 50). In turn, all the samples from Fonds-de-Forêt, Spy, Trou Magrite and Goyet appear closer to each other than they are to the Mezmaiskaya 2, Vindija 33.19 or Les Cottés Z4-1514 Neandertals (Supplementary Figures 51 to 60).

Beyond that, there is tentative evidence for differential relationships among the newly sequenced individuals, although statistical power remains too low for most comparisons. For example, GN2 shares significantly more alleles with GN1 than with Spy 94a_8 (Supplementary Figure 58). At first glance, Goyet Q305-4 also appears to share more alleles with the Fonds-de-Forêt Neandertal than with GN2 (Supplementary Figure 60). However, this pattern is likely driven by low coverage. A more detailed analysis of the relationships among these three individuals reveals conflicting signals, with some support instead for Fonds-de-Forêt 1 being closer to GN2 than to Goyet Q305-4 (Supplementary Figure 58). Specifically, among the 1,769 sites informative for the D-statistic *D(Goyet Q305-4, GN2; Fonds-*

de-Forêt 1, D5), 11 sites are shared between the Goyet individuals but differ in Fonds-de-Forêt 1 and D5 (AABB), 23 sites are shared between Fonds-de-Forêt 1 and GN2 (ABBA), and 24 sites between Fonds-de-Forêt 1 and Goyet Q305-4 (BABA). The similar numbers of ABBA and BABA sites, combined with a relatively low AABB count, suggest no meaningful biological signal and are best interpreted as noise. Therefore, the most parsimonious explanation is that the Goyet individuals were more closely related to each other than to Neandertals from other caves, such as Spy. However, additional data will be necessary to clarify these patterns.

Finally, we examined the relationships of AR-30 to the other Neandertals. Beyond comparisons to the high coverage individuals, the relationship to other Neandertals should be interpreted with caution since most of the direct comparisons in the form of D(W, X; AR-30, D5) and D(AR-30, X; Y, D5) rely on less than 1,000 overlapping positions. When compared to GN1 and Les Cottés Z4-1514, the AR-30 seems to be cladal with Les Cottés (Supplementary Figure 56). However, at the same time the AR-30 shares significantly more alleles with GN1 than with Les Cottés (Supplementary Figure 50), suggesting some level of gene-flow with GN1.

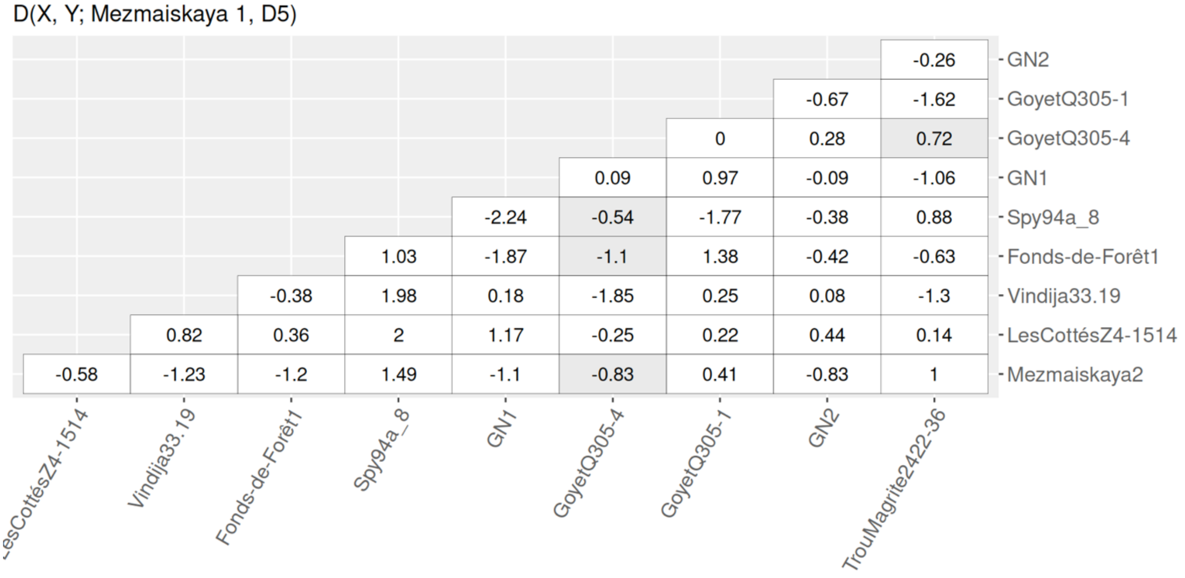

Supplementary Figure 50 D-statistics of the form *D("X", "Y"; Mezmaiskaya 1, D5)*, where "*X*" and "*Y*" represent the focus Neandertals on the X-axis and Y-axis respectively, coloured in function of their significance (Z-score > 3 in yellow, Z-score < -3 in blue, non-significant remains uncoloured, comparisons based on less than 1,000 overlapping positions coloured in grey).

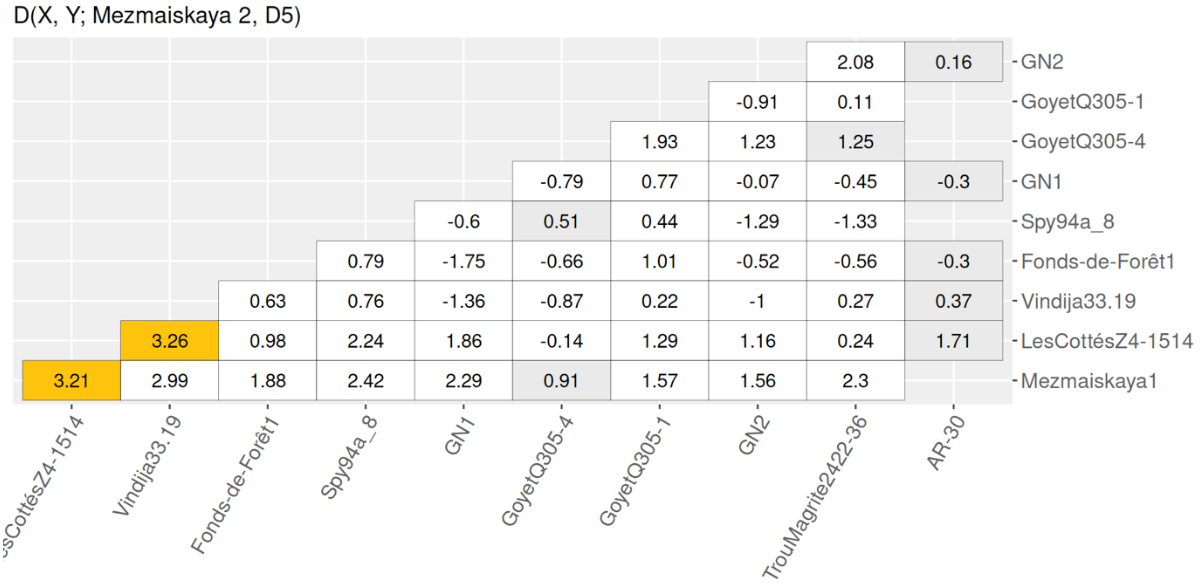

Supplementary Figure 51 D-statistics of the form *D("X", "Y"; Mezmaiskaya 2, D5)*, where "*X*" and "*Y*" represent the focus Neandertals on the X-axis and Y-axis respectively, coloured in function of their significance (Z-score > 3 in yellow, Z-score < -3 in blue, non-significant remains uncoloured, comparisons based on less than 1,000 overlapping positions coloured in grey).

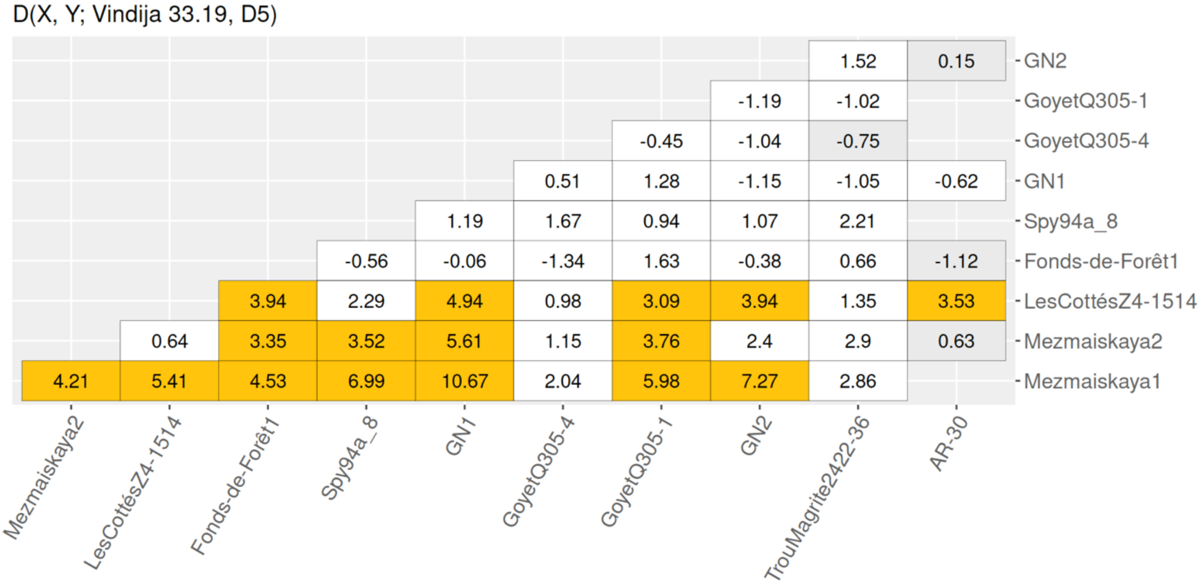

Supplementary Figure 52 D-statistics of the form *D("X", "Y"; Vindija 33.19, D5)*, where "*X*" and "*Y*" represent the focus Neandertals on the X-axis and Y-axis respectively, coloured in function of their significance (Z-score > 3 in yellow, Z-score < -3 in blue, non-significant remains uncoloured, comparisons based on less than 1,000 overlapping positions coloured in grey).

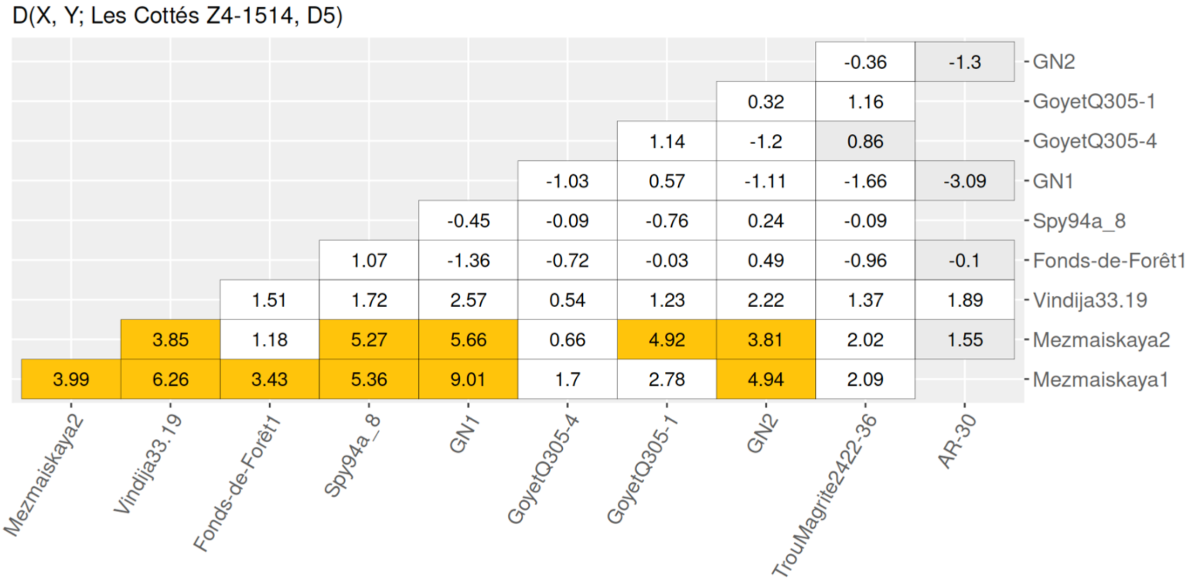

Supplementary Figure 53 D-statistics of the form *D("X", "Y"; Les Cottés Z4-1514, D5)*, where "*X*" and "*Y*" represent the focus Neandertals on the X-axis and Y-axis respectively, coloured in function of their significance (Z-score > 3 in yellow, Z-score < -3 in blue, non-significant remains uncoloured, comparisons based on less than 1,000 overlapping positions coloured in grey).

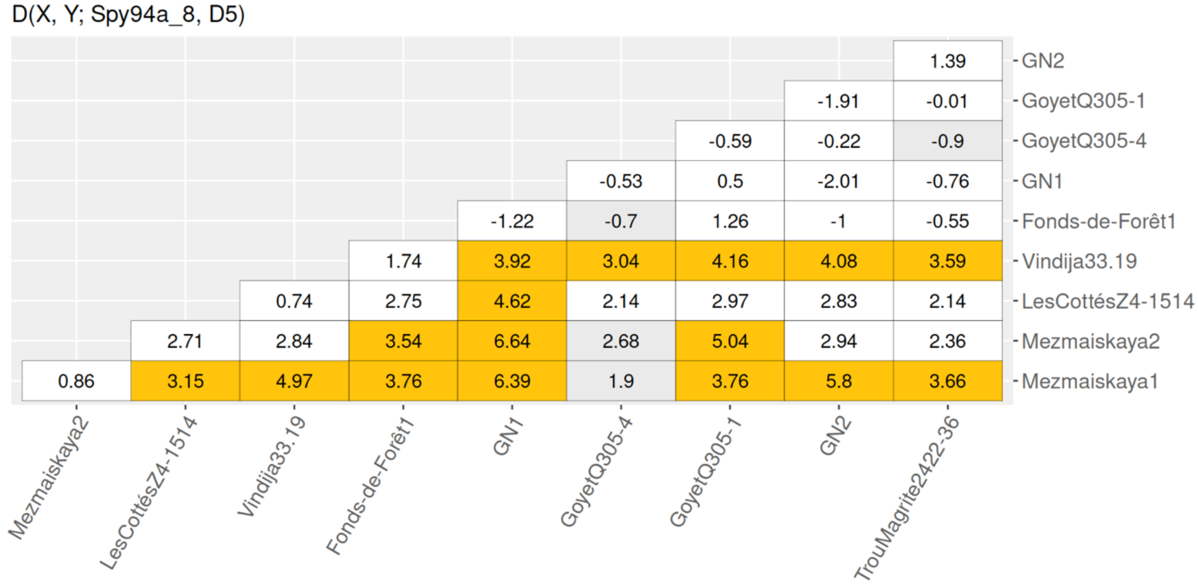

Supplementary Figure 54 D-statistics of the form *D("X", "Y"; Spy 94a_8, D5)*, where "*X*" and "*Y*" represent the focus Neandertals on the X-axis and Y-axis respectively, coloured in function of their significance (Z-score > 3 in yellow, Z-score < -3 in blue, non-significant remains uncoloured, comparisons based on less than 1,000 overlapping positions coloured in grey).

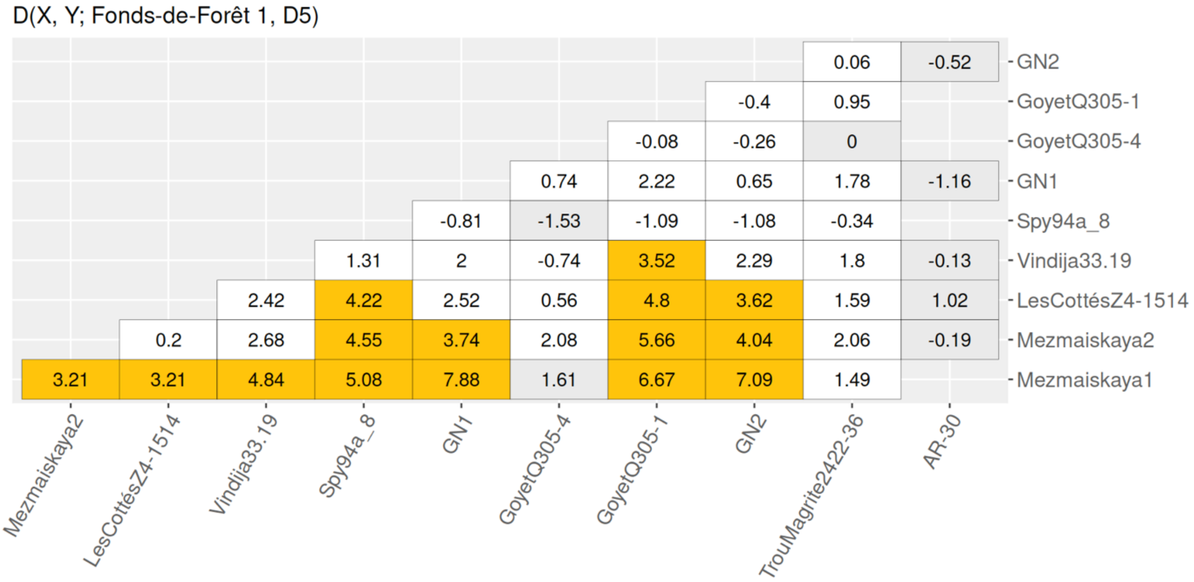

Supplementary Figure 55 D-statistics of the form *D("X", "Y"; Fonds-de-Forêt 1, D5)*, where "*X*" and "*Y*" represent the focus Neandertals on the X-axis and Y-axis respectively, coloured in function of their significance (Z-score > 3 in yellow, Z-score < -3 in blue, non-significant remains uncoloured, comparisons based on less than 1,000 overlapping positions coloured in grey).

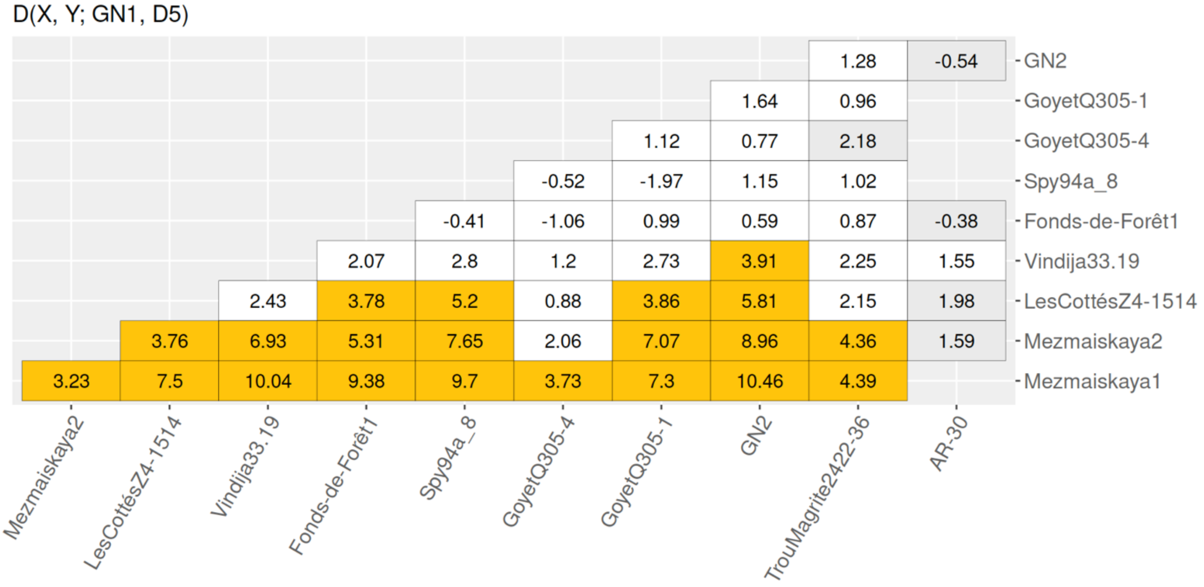

Supplementary Figure 56 D-statistics of the form *D("X", "Y"; GN1, D5)*, where "*X*" and "*Y*" represent the focus Neandertals on the X-axis and Y-axis respectively, coloured in function of their significance (Z-score > 3 in yellow, Z-score < -3 in blue, non-significant remains uncoloured, comparisons based on less than 1,000 overlapping positions coloured in grey).

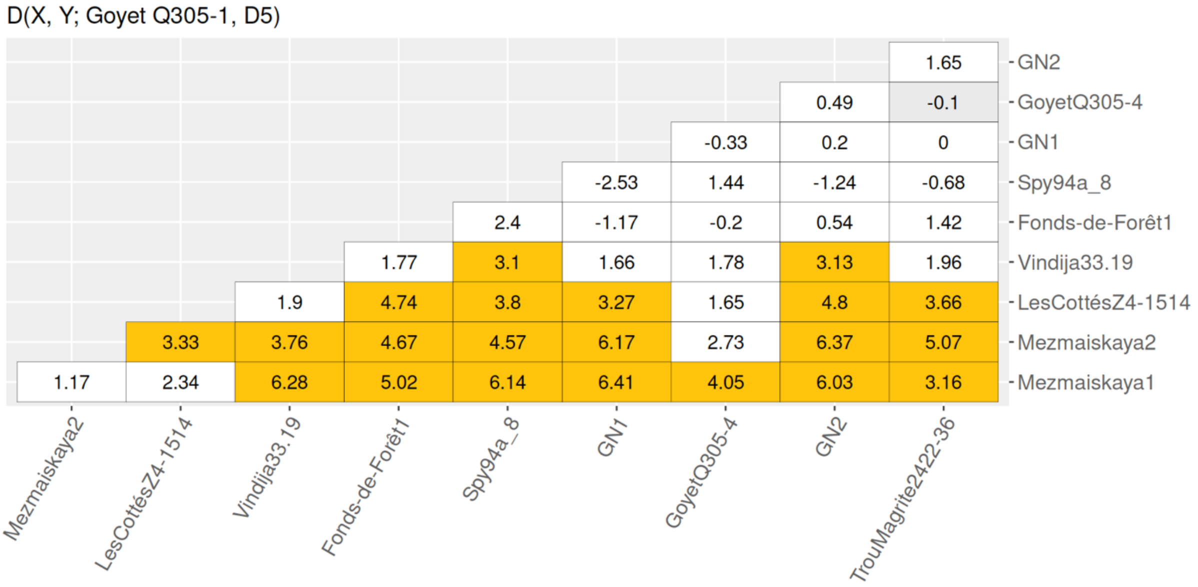

Supplementary Figure 57 D-statistics of the form *D(“X”, ”Y”; Goyet Q305-1, D5)*, where ”*X*” and “*Y*” represent the focus Neandertals on the X-axis and Y-axis respectively, coloured in function of their significance (Z-score > 3 in yellow, Z-score < -3 in blue, non-significant remains uncoloured, comparisons based on less than 1,000 overlapping positions coloured in grey).

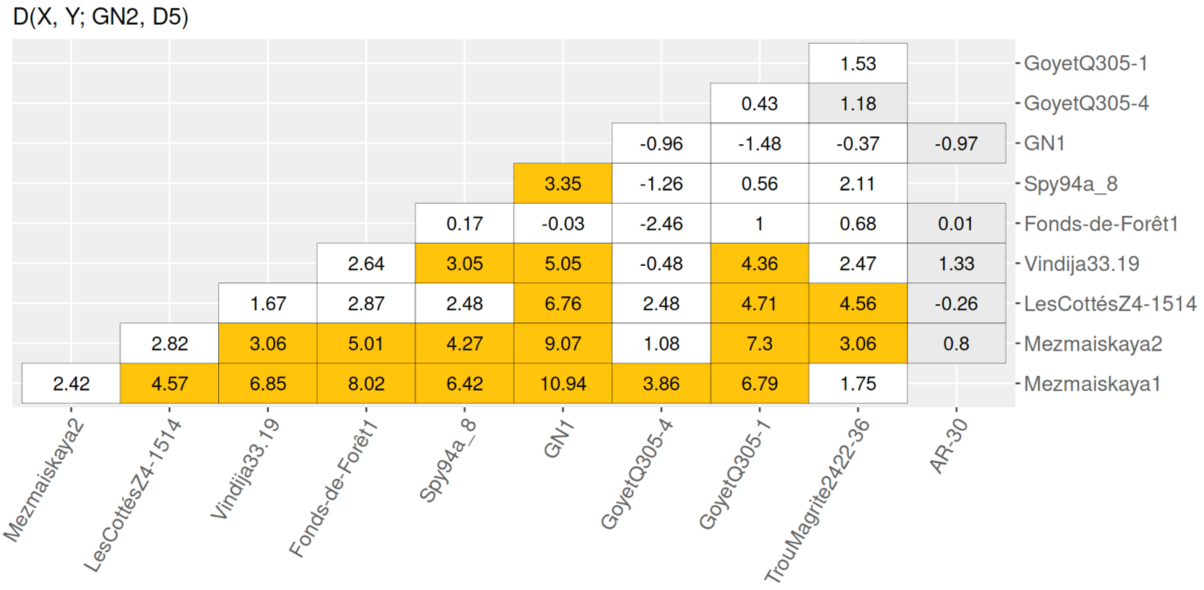

Supplementary Figure 58 D-statistics of the form *D(“X”, ”Y”; GN2, D5)*, where ”*X*” and “*Y*” represent the focus Neandertals on the X-axis and Y-axis respectively, coloured in function of their significance (Z-score > 3 in yellow, Z-score < -3 in blue, non-significant remains uncoloured, comparisons based on less than 1,000 overlapping positions coloured in grey).

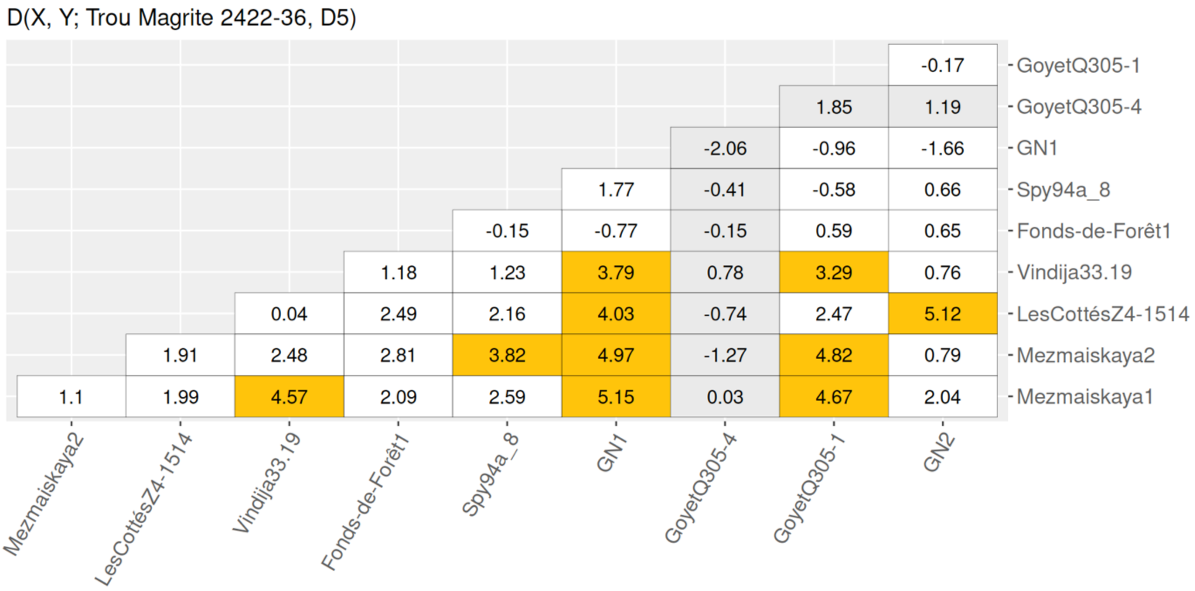

Supplementary Figure 59 D-statistics of the form *D("X", "Y"; Trou Magrite 2422-36, D5)*, where "*X*" and "*Y*" represent the focus Neandertals on the X-axis and Y-axis respectively, coloured in function of their significance (Z-score > 3 in yellow, Z-score < -3 in blue, non-significant remains uncoloured, comparisons based on less than 1,000 overlapping positions coloured in grey).

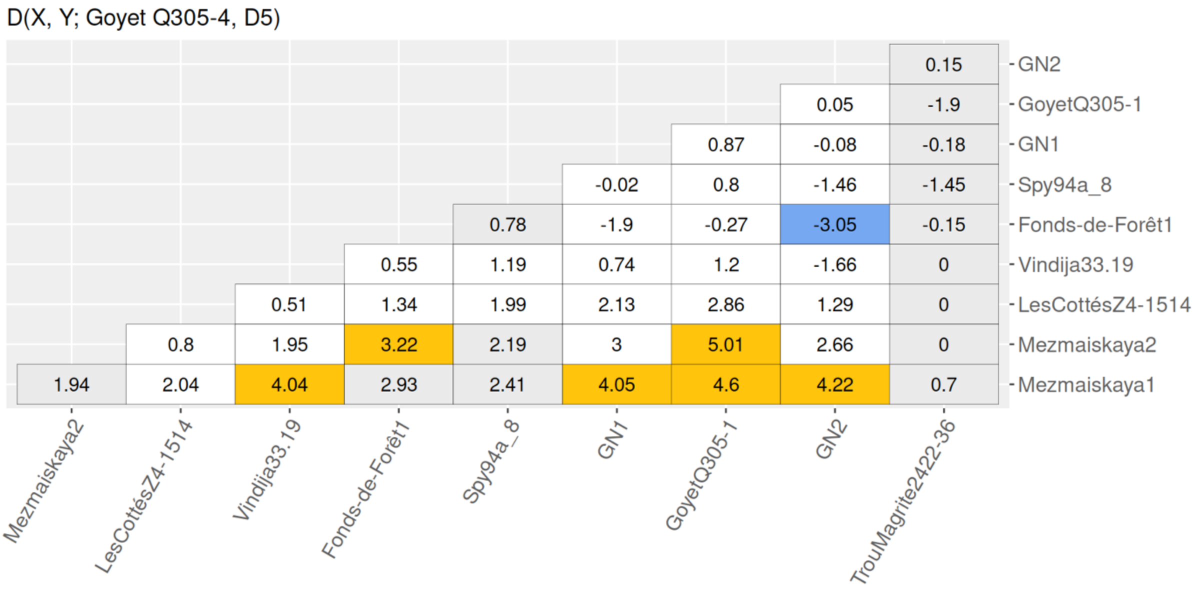

Supplementary Figure 60 D-statistics of the form *D("X", "Y"; Goyet Q305-4, D5)*, where "*X*" and "*Y*" represent the focus Neandertals on the X-axis and Y-axis respectively, coloured in function of their significance (Z-score > 3 in yellow, Z-score < -3 in blue, non-significant remains uncoloured, comparisons based on less than 1,000 overlapping positions coloured in grey).

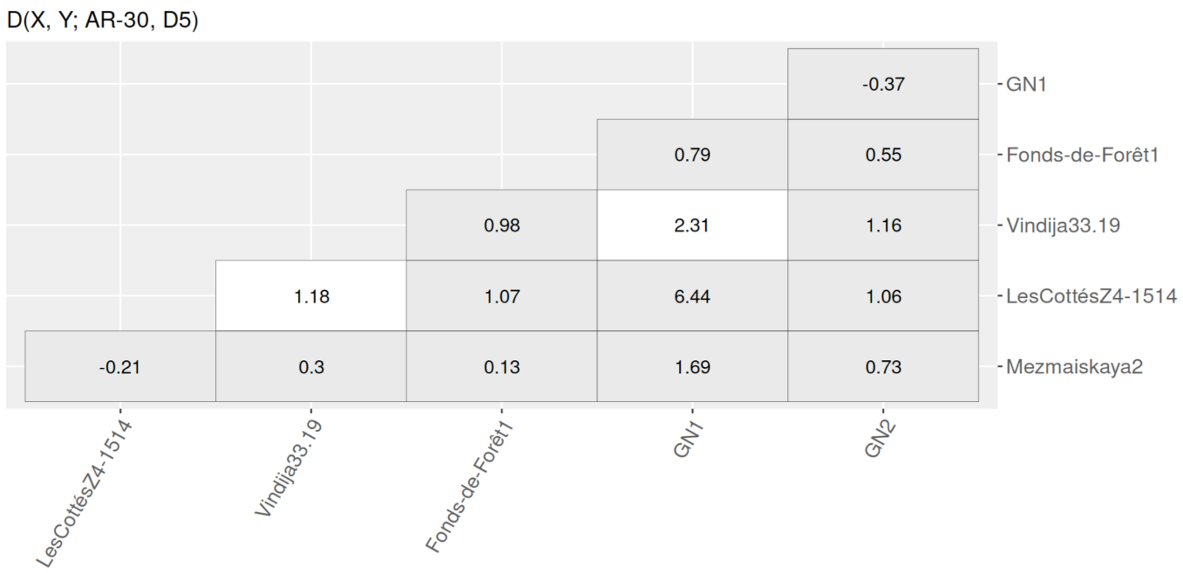

Supplementary Figure 61 D-statistics of the form *D("X", "Y"; AR-30, D5)*, where "*X*" and "*Y*" represent the focus Neandertals on the X-axis and Y-axis respectively, coloured in function of their significance (Z-score > 3 in yellow, Z-score < -3 in blue, non-significant remains uncoloured, comparisons based on less than 1,000 overlapping positions coloured in grey).

Summary

With both the D-statistics and outgroup-f3-analyses we find that the individuals from Fonds-de-Forêt, Spy and Trou Magrite are closest to the GN1 high coverage Neandertal, indicating that they form a distinct clade of Neandertals, consistent with their geographic location. The low-coverage samples from Goyet also mostly fall in this group, although for some individuals the data is too sparse for statistically significant conclusions. Overall, we find some evidence for structure within the newly sequenced Neandertals from Belgium and France, but more data would be needed to clarify the finer scale relationships among them.

References

1. Meyer, M. *et al.* A High-Coverage Genome Sequence from an Archaic Denisovan Individual. *Science* **338**, 222–226 (2012).
2. Prüfer, K. *et al.* The complete genome sequence of a Neanderthal from the Altai Mountains. *Nature* **505**, 43–49 (2014).
3. Mafessoni, F. *et al.* A high-coverage Neandertal genome from Chagyrskaya Cave. *Proc. Natl. Acad. Sci.* **117**, 15132–15136 (2020).
4. Prüfer, K. *et al.* A high-coverage Neandertal genome from Vindija Cave in Croatia. *Science* **358**, 655–658 (2017).

5. Lachance, J. & Tishkoff, S. A. SNP ascertainment bias in population genetic analyses: Why it is important, and how to correct it. *BioEssays* **35**, 780–786 (2013).
6. Hajdinjak, M. *et al.* Reconstructing the genetic history of late Neanderthals. *Nature* **555**, 652–656 (2018).
7. Slimak, L. *et al.* Long genetic and social isolation in Neanderthals before their extinction. *Cell Genomics* **4**, 100593 (2024).
8. Green, R. E. *et al.* A Draft Sequence of the Neandertal Genome. *Science* **328**, 710–722 (2010).
9. Prüfer, K. snpAD: an ancient DNA genotype caller. *Bioinformatics* **34**, 4165–4171 (2018).
10. Petr, M., Vernot, B. & Kelso, J. admixr —R package for reproducible analyses using ADMIXTOOLS. *Bioinformatics* **35**, 3194–3195 (2019).
11. Patterson, N. *et al.* Ancient Admixture in Human History. *Genetics* **192**, 1065–1093 (2012).
12. Bokelmann, L. *et al.* A genetic analysis of the Gibraltar Neanderthals. *Proc. Natl. Acad. Sci.* **116**, 15610–15615 (2019).
13. Pugach, I. *et al.* Ancient DNA from Guam and the peopling of the Pacific. *Proc. Natl. Acad. Sci.* **118**, e2022112118 (2021).
14. Wang, K. *et al.* 4000-year-old hair from the Middle Nile highlights unusual ancient DNA degradation pattern and a potential source of early eastern Africa pastoralists. *Sci. Rep.* **12**, 20939 (2022).
15. Patterson, N., Price, A. L. & Reich, D. Population Structure and Eigenanalysis. *PLoS Genet.* **2**, e190 (2006).
16. Price, A. L. *et al.* Principal components analysis corrects for stratification in genome-wide association studies. *Nat. Genet.* **38**, 904–909 (2006).
17. Peter, B. M. A geometric relationship of F_2, F_3 and F_4-statistics with principal component analysis. *Philos. Trans. R. Soc. B Biol. Sci.* **377**, 20200413 (2022).
18. Bhatia, G., Patterson, N., Sankararaman, S. & Price, A. L. Estimating and interpreting F_{ST}: The impact of rare variants. *Genome Res.* **23**, 1514–1521 (2013).

16. Split times

To estimate the split times between our set of Neandertals and other archaic human groups, we calculated *f(A|B)* statistics[1–3]. The *f(A|B)* statistic calculates the proportion of shared derived alleles between two genomes (hereby called "A" and "B"), after restricting the calculation to heterozygous positions in "B", and pseudo-haploidising "A". For closely related individuals *A, B, f(A|B)* ≈ 0.5, since the lineage from A is equally likely to coalesce first with either allele in B. The more distant the relationship, the more likely the A-lineage coalesces with the ancestral allele, and *f(A|B)* will be lower. To get the *f(A|B)* estimations, we followed an existing workflow[4], briefly summarized here.

As genome "A", we used the newly generated data from the individuals from Arcy-sur-Cure, Les Cottés, Fonds-de-Forêt, Goyet, Spy and Trou Magrite, genotyped and pseudo-haploidised as described in Section 15. As genome "B", we included the high-coverage archaic genomes of Denisova 3[5], Vindija 33.19[6], D5[1], Chagyrskaya 8[3]. We defined if their alleles were ancestral or derived using four great ape genomes as outgroups: a chimpanzee (panTro4, GCA_000001515.4), a bonobo (panPan1.1, AJFE00000000.2), a gorilla (gorGor3, GCA_000001515.4) and an orangutan (ponAbe2, GCA_000001545.3) – requiring that they all carried the ancestral allele. We used a block-jackknife with 5Mb windows to estimate the uncertainty of the *f(A|B)*.

As the split-time estimates obtained from *f(A|B)* are in terms of coalescent probabilities, they are not easily interpretable. We thus used calibration simulations to translate *f(A|B)* into split times in years, using a specific calibration curve for each genome "B". We generated calibration curves using simulations of the demographic history of genome B (obtained in Section 8), sampling at different time points and computing the *f(A|B)* that one would expect to obtain for each split time. This required a set of assumptions relating to the generation time (29 years) and mutation rate of ($1.45\text{x}10^{-8}$ per base pair per generation). We show an example of this for the GN1 high-coverage genome in Supplementary Figure 62, and for the low-coverage Goyet Q305-1 capture data in Supplementary Figure 63.

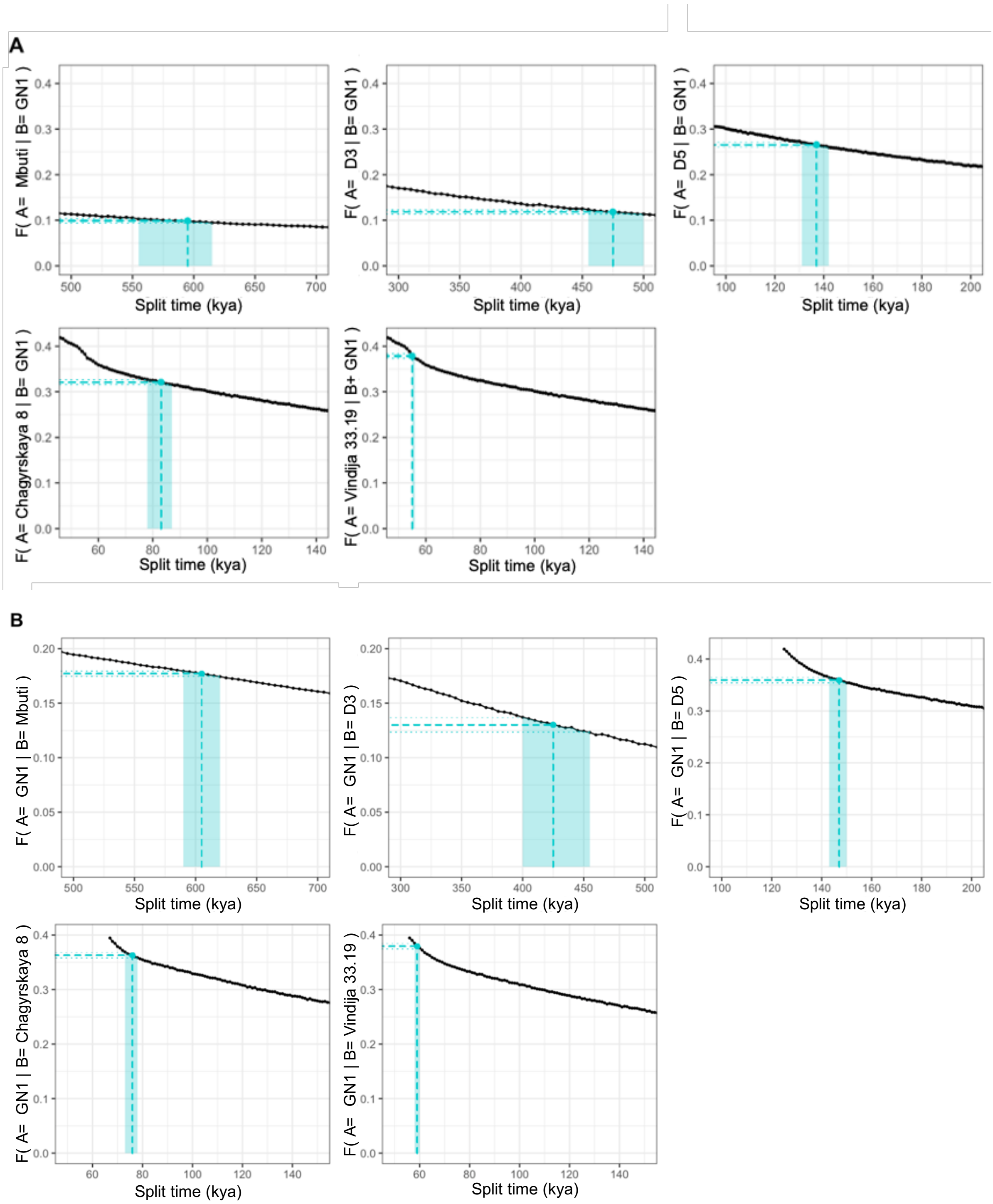

Supplementary Figure 62 Calibration curves of expected *f(A|B)* values at different split times for each "B" genome, in black, and the observed *f(A|B)* values with their 95% confidence interval (CI), in cyan. A) GN1 as genome "B", B) GN1 as genome "A".

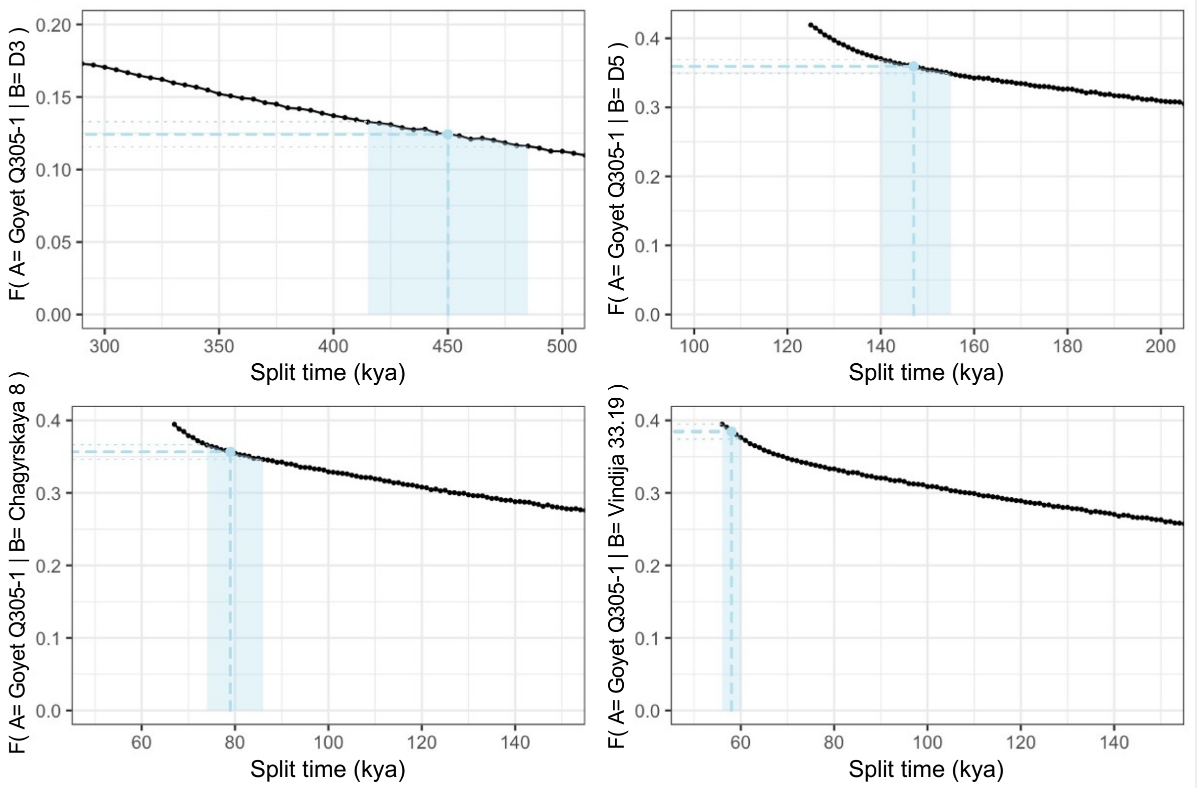

Supplementary Figure 63 Calibration curves of expected *f(A|B)* values at different split times for each "B" genome, in black, and the observed *f(A|B)* values with their 95% confidence interval (CI), in blue, for Goyet Q305-1 as genome "A".

Importantly, for capture data – such as the example of Goyet Q305-1 detailed above – not all split times could be estimated. This is because the array ascertainment limits the number of suitable "B" genomes. The "ArchaicPlus" array was designed with the goal of maximising the amount of archaic variation captured, based on the high coverage archaic genomes of Denisova 3, D5, Chagyrskaya 8 and Vindija 33.19 (Section 12). Therefore, while we are confident that all the heterozygous positions from these genomes are part of the array, this is not the case for other non-represented groups, such as the younger Neandertal population to which GN1 belonged. Using capture data we can only analyse heterozygous positions that were already present in the known lineages (e.g. Vindija 33.19), but not the private variants from GN1, which would bias the results. Consequently, we refrained including the GN1 high-coverage genome as population B, except when we exclusively compare high-coverage genomes (Supplementary Figure 62).

We found that the split times of all the captured Neandertals in respect to modern humans (represented by Mbuti) were unexpectedly recent: ~350 kya instead of the ~600 kya that would be expected [1,6,3], as reported in Supplementary Figure 64. Given that the "ArchaicPlus" array focused on Neandertal and

Denisovan variation, and did not include most alleles informative of modern human population history, the ascertainment likely also explains these observations.

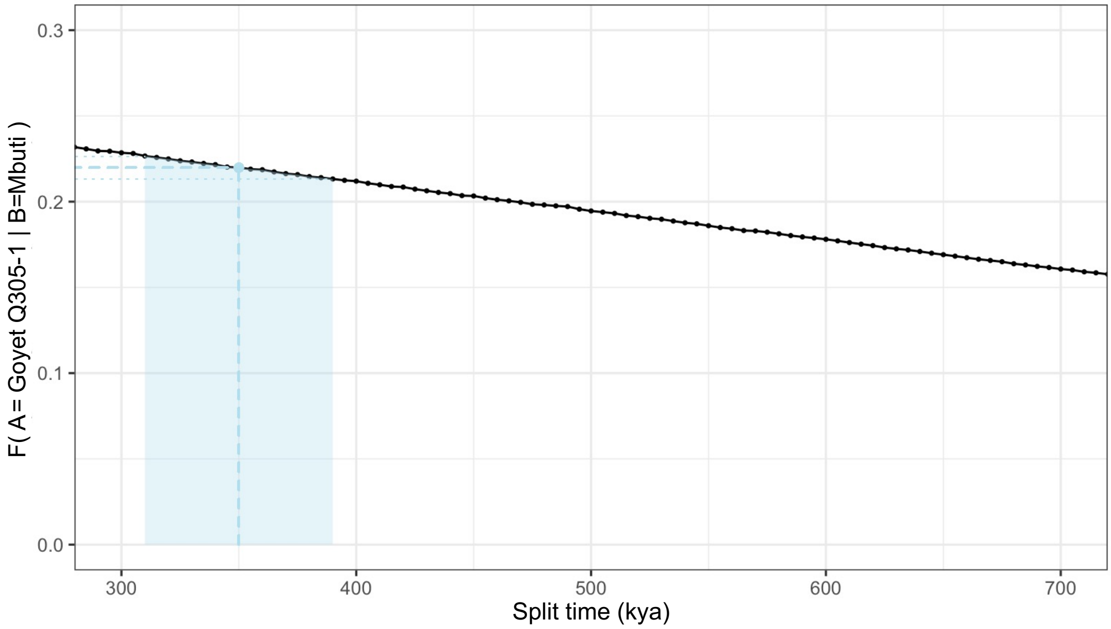

Supplementary Figure 64 Mbuti calibration curves of expected *f(A|B)* values at different split times, in black, and the intersecting observed *f(A|B)* values with their 95% confidence interval (CI) for Goyet Q305-1, in blue.

To test this hypothesis, we ascertained the high-coverage GN1 genome to the "ArchaicPlus" target sites, and re-estimated the *f(A|B)* and split times as before. The results, represented in Supplementary Figure 65, confirmed our hypothesis, showing how the split time *f(A = GN1 | B = Mbuti)* shifted from 605 kya (620 - 590 kya) to 345 kya (375 - 320 kya). This ascertainment bias prevents us from estimating the split time between present-day modern humans and the Neandertals captured with the "ArchaicPlus" array.

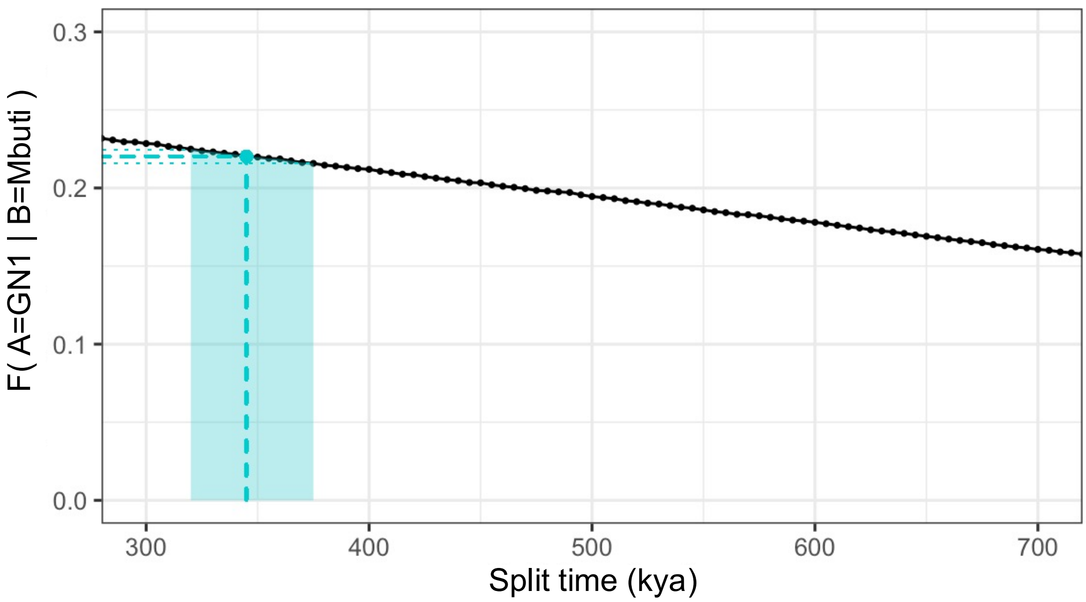

Supplementary Figure 65 Mbuti calibration curves of expected *f(A|B)* values at different split times, in black, and the intersecting observed *f(A|B)* values with their 95% confidence interval (CI) for GN1 ascertained to the "ArchaicPlus" sites, in cyan.

Therefore, given the "ArchaicPlus" ascertainment, we limited our calculations to the *f(A = Sample | B = Denisova 3, D5, Chagyrskaya 8 or Vindija 33.19)*. As reported in Section 13, we interpreted the results of Goyet 1424-3D, Goyet C5-1, Goyet Q119-2, Goyet Q376-25 and Goyet Q55-4 with caution. These Neandertals all have wide confidence intervals due to the high contamination levels and low coverage.

Focusing on the rest of the low-coverage samples from present-day Belgium and France, we found that their split time estimates with respect to Denisova 3, D5 and Chagyrskaya 8 (Supplementary Figures 66-68; Supplementary Data Table 8) all overlap within their 95% confidence intervals, showing no evidence of population structure at this level.

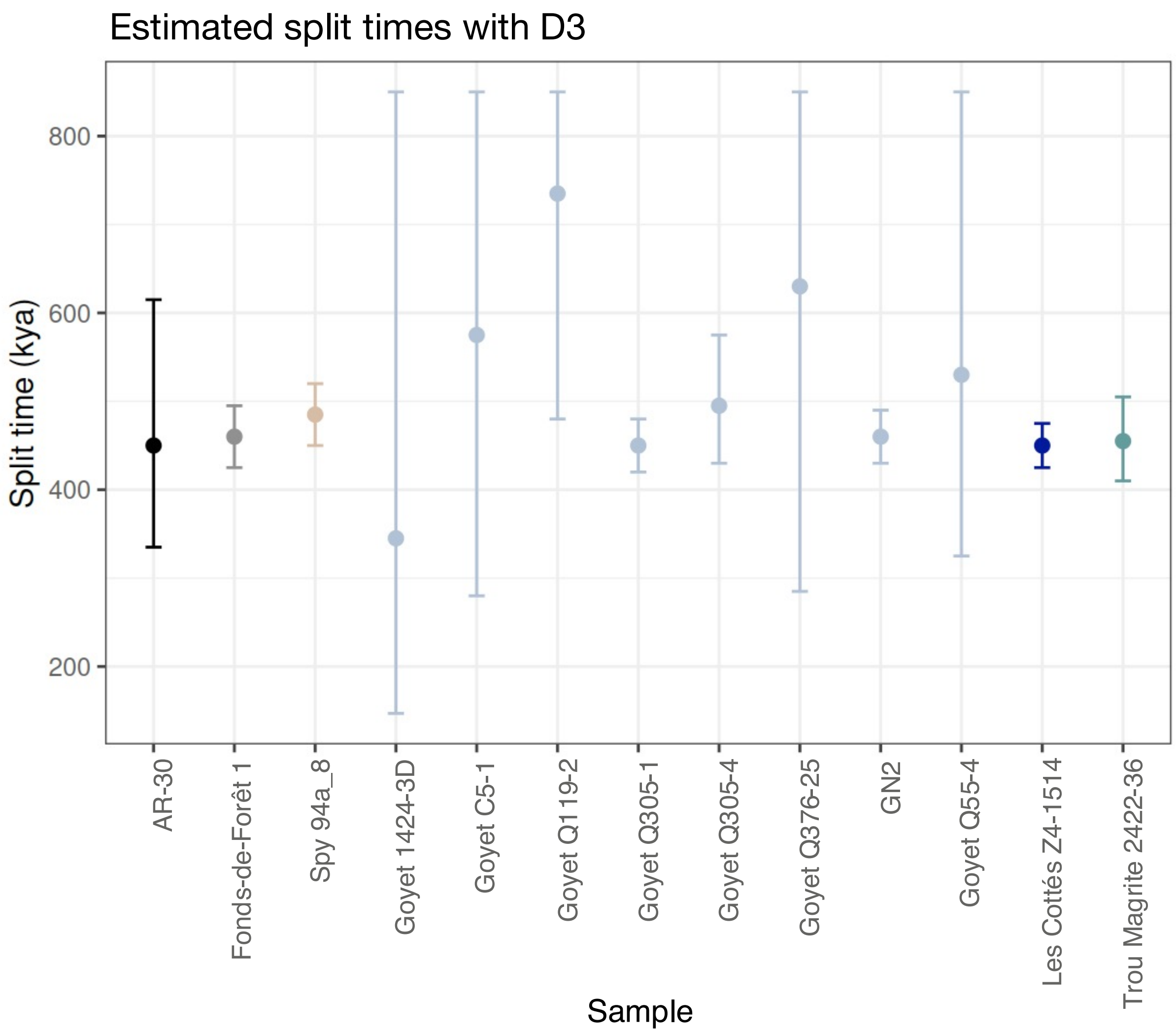

Supplementary Figure 66 Split time estimates with D3 (Denisovan). Point estimates and 95% confidence intervals are given for each Neandertal sample, coloured by site.

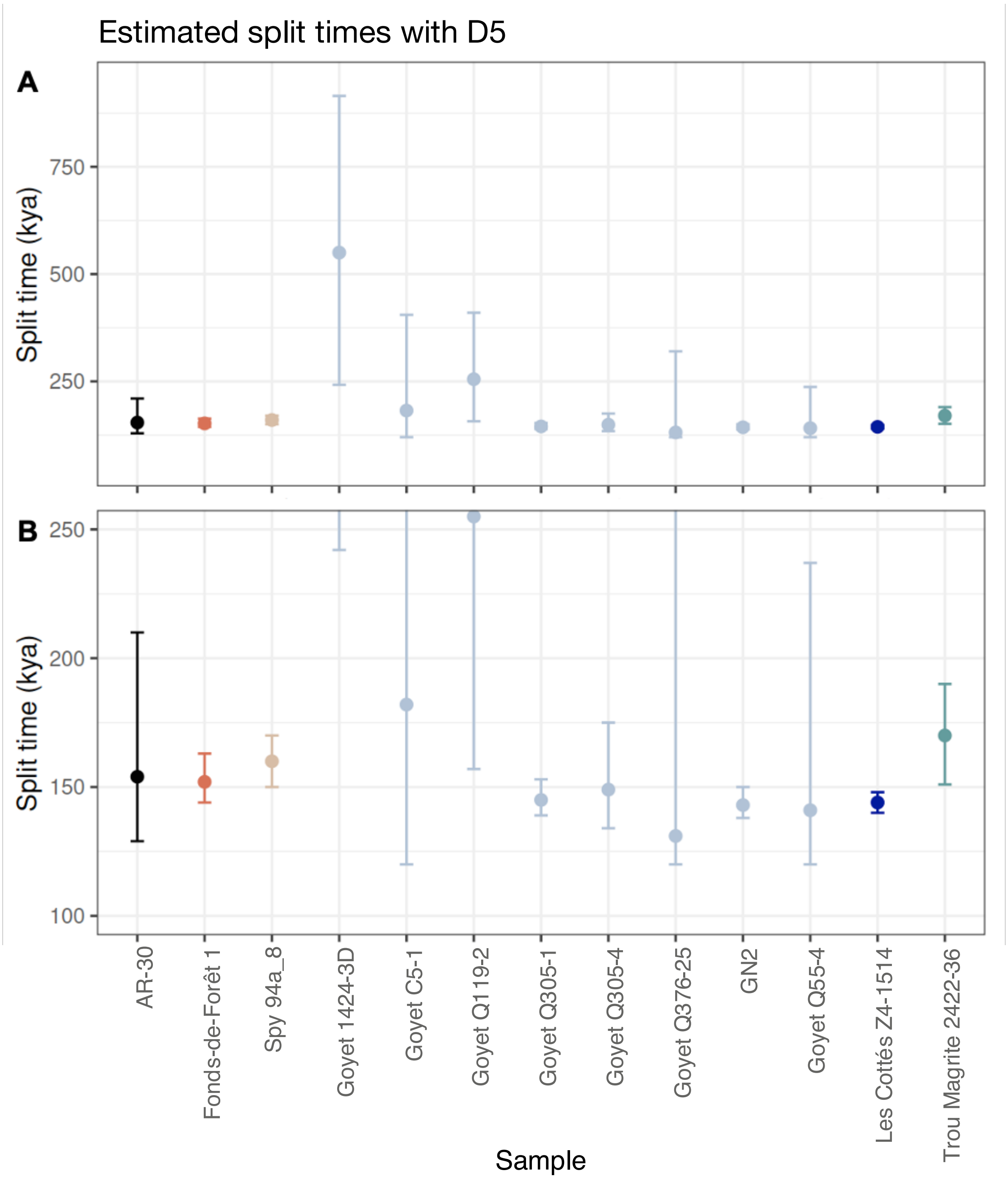

Supplementary Figure 67 Split time estimates with D5 (the "Altai" Neandertal). Point estimates and 95% confidence intervals are given for each Neandertal sample, coloured by site. A) All estimates, B) zoomed-in estimates between split times of 250 kya and 100 kya.

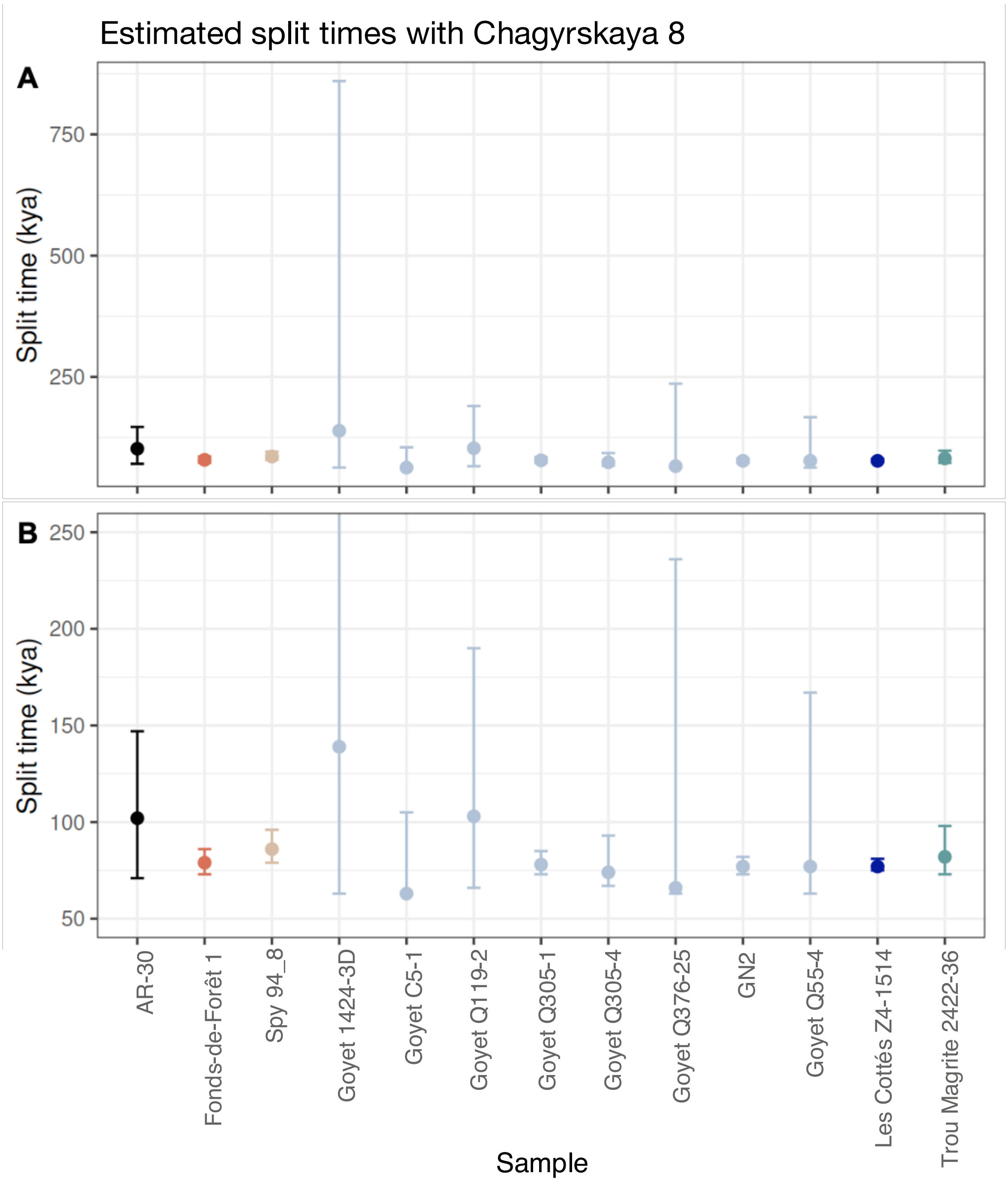

Supplementary Figure 68 Split time estimates with Chagyrskaya 8. Point estimates and 95% confidence intervals are given for each Neandertal sample, coloured by site. A) All estimates, B) zoomed-in estimates between split times of 250 kya and 50 kya.

This changes when calculating *f(A = Sample | B = Vindija 33.19)*: While the majority of the samples have a median split time around 54 kya (with 95% CI ranging from 58 kya to 51 kya), the ancestors of AR-30 split from the Vindija population much earlier, at ~131 kya (95% CI: 195 - 82 kya) (Supplementary Figure 69).

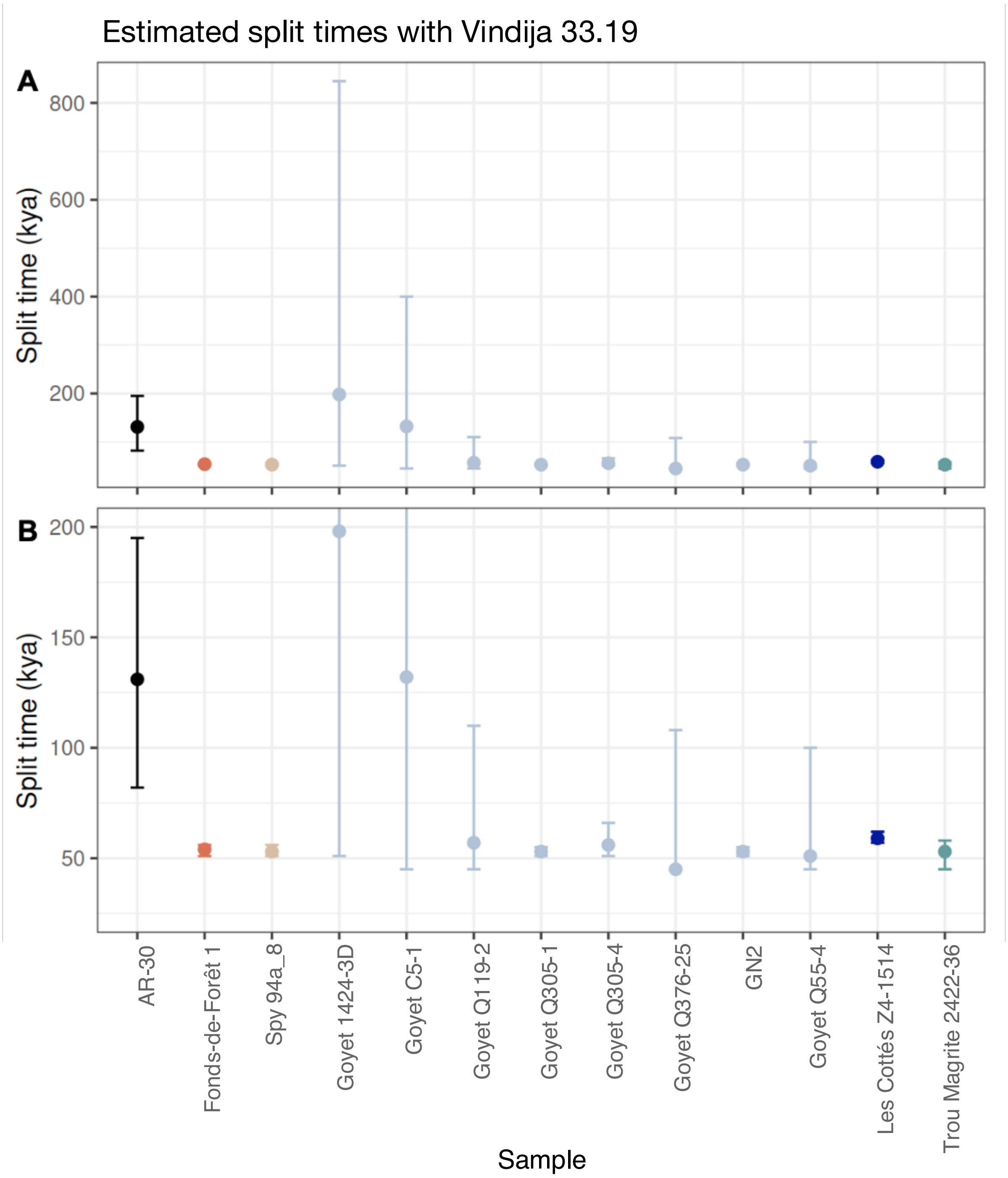

Supplementary Figure 69 Split time estimates with Vindija 33.19. Point estimates and 95% confidence intervals are given for each Neandertal sample, coloured by site. A) All estimates, B) zoomed-in estimates between split times of 200 kya and 50 kya.

The deeper split time of AR-30 is unexpected considering that the population genetic analyses presented in Section 15 showed no indication of a differential relationship with the other high-coverage genomes. Specifically, AR-30 belonged to the phylogenetic branch defined by the Late Neandertal Vindija 33.19 and GN1, sharing more affinity with them than with Chagyrskaya 8, just like the rest of the studied

Neandertals. Yet we find that AR-30 has a lower proportion of variants derived in Vindija 33.19 and/or Chagyrskaya 8 (Extended Data Fig. 7).

This excess of ancestral alleles in AR-30 could be explained by several scenarios. For instance, it could be due to the unaccounted presence of contaminant DNA, either faunal or present-day human. We ruled out the former option given the high proportion of derived alleles specific to archaic hominins (97.57% support for that branch, with 95% CI of 96.15% - 98.58%) and the fact that metagenomic pipelines such as quicksand (version 2.3)[7] only assign the reads to the *Hominidae* family, and no other organism. As for present-day human contamination, we used the linear combination method[8] to calculate the proportion of present-day human DNA that would be necessary to explain these results:

$$\mathrm{P} = c \times pc + (1 - c) \times pe$$

where "P" is the proportion of derived alleles observed, pe is the expectation of that value for a non-contaminated Neandertal (we used GN1 as a proxy), and pc is the expectation of that value for a contaminant present-day human (we used a French individual as a proxy, HGDP00521[5]. Specifically, in our case, P = 0.61 (95% CI of 0.53 - 0.69), pe = 0.74 and pc = 0.06. As a result, we obtain that the contamination levels would need to be at least 19.48% (95% CI of 8.33% - 21.24 %) to justify our observations, which is significantly higher than the contamination estimates that we obtained for that specimen at 1.31 % (95% CI of 0.26% - 2.77 %, Supplementary Data Table 5).

Besides contamination, another plausible scenario that would explain this excess of ancestral alleles in AR-30 would be gene flow from a deeper Neandertal lineage. However, due to the low amount of data and ascertainment bias of the array we cannot corroborate this hypothesis.

References

1. Prüfer, K. *et al.* The complete genome sequence of a Neanderthal from the Altai Mountains. *Nature* **505**, 43–49 (2014).
2. Hajdinjak, M. *et al.* Reconstructing the genetic history of late Neanderthals. *Nature* **555**, 652–656 (2018).
3. Mafessoni, F. *et al.* A high-coverage Neandertal genome from Chagyrskaya Cave. *Proc. Natl. Acad. Sci.* **117**, 15132–15136 (2020).
4. Sümer, A. P. *et al.* Earliest modern human genomes constrain timing of Neanderthal admixture. *Nature* **638**, 711–717 (2025).
5. Meyer, M. *et al.* A High-Coverage Genome Sequence from an Archaic Denisovan Individual. *Science* **338**, 222–226 (2012).

6. Prüfer, K. *et al.* A high-coverage Neandertal genome from Vindija Cave in Croatia. *Science* **358**, 655–658 (2017).
7. Szymanski, M., Visagie, J., Romagne, F., Meyer, M. & Kelso, J. quick analysis of sedimentary ancient DNA using quicksand. Preprint at https://doi.org/10.1101/2025.08.01.668088 (2025).
8. Peyrégne, S. *et al.* Nuclear DNA from two early Neandertals reveals 80,000 years of genetic continuity in Europe. *Sci. Adv.* **5**, eaaw5873 (2019).

17. Local ancestry inference

The Neandertals in this study are all close in time to the main pulse of the modern human-Neandertal admixture, dated to around 47,000 years ago[1,2] and are contemporaneous with the first Upper Palaeolithic humans in Europe[3,4,2]. To date, the available evidence supports that the latest gene flow events between these two groups were asymmetrical, i.e., from Neandertals to modern humans and not vice versa. Here, we attempt to identify modern human introgressed segments in the genomes of these Neandertal individuals.

Given the ages of the studied Neandertals (49,738 calBP to 39,960 calBP, see Supplementary Table 24), if there was gene flow in the opposite direction, we would expect this gene flow to be very recent, i.e., in the range of no more than 15,000 years or $t = 500$ generations. Assuming the segment length to be exponentially distributed with the rate parameter = $1/t$, we expect the length of introgressed segments to have a mean length of 0.2 cM, and 1% of the segments to be longer than 1 cM. Given a callable genome of 2,750 cM and an adxmiture proportion of 3%, we would expect ~8 segments longer than 1cM per individual[5].

We used *admixfrog* (version 0.7.2)[6], for detecting introgressed segments. This program, through a hidden Markov model (HMM), bins diploid genomes in small windows, and probabilistically assigns them to the best-matching ancestry from a given set of high-coverage reference genomes. To account for the challenges that low-coverage ancient samples pose, *admixfrog* incorporates an explicit model of present-day human contamination, which allows it to co-estimate the genotype likelihoods and contamination levels. The results are local ancestry segments, which can be inferred as either homozygous or heterozygous.

We used the genotypes, generated using a DNA damage aware genotyper *snpAD*[7], of the ~45,000-year-old Vindija 33.19[8], ~120,000-year-old D5[9] and ~60,000-year-old Chagyrskaya 8[10] Neandertals to define the Neandertal state (NEA). We chose individuals from the Sub-Saharan Esan, Luhya, Mandinka, Mende and Yoruba populations of the 1000 Genomes project[11] to define the modern human state (AFR). To account for the possible Denisovan ancestry as reported in Skov et al., 2021, we used the snpAD genotypes of the ~50,000-year-old Denisova 3 individual[12] as a reference for the Denisovan ancestry (DEN). Alleles were further polarised using the chimpanzee genotypes (panTro4, GCA_000001515.4), resulting in a total of 2,249,493 informative sites of the joint variation of these genomes.

We parsed the bam files of our focus Neandertals, using the following command:

```
admixfrog-bam --bam ${sample}.bam --ref ${ref}.csv.xz --out ${sample}.in.xz --length-bin-size 10
```

Because *admixfrog* can model contamination, we used all sequences in this analysis, unlike in previous sections where we used solely sequences with ancient DNA damage, i.e., only the deaminated fraction of the data. We then ran *admixfrog* following[1], calling the segments with the parameters detailed below:

```
admixfrog --infile ${sample}.in.xz --ref ${ref}.csv.xz --out ${sample}_AFK_NEA_DEN --states AFR NEA=CHA+ALT+VIN DEN --cont-id AFR --ll-tol 0.01 --bin-size 5000 --est-F --est-tau --freq-F 3 --freq-contamination 3 --e0 0.01 --est-error --ancestral PAN --run-penalty 0.25 --max-iter 250 --n-post-replicates 200 --filter-pos 50 --filter-map 0.000
```

We used a cut-off of 0.2 cM to call ancestry segments with a low false-positive rate[6]. The data from Goyet 1424-3D, Goyet C5-1, Goyet Q119-2 and Goyet Q376-25 had coverages that were below the recommended 0.02x threshold for reliably running *admixfrog*. We still ran *admixfrog,* but these runs resulted in inconclusive posteriors, where not even the base population could be confidently identified as Neandertal. Therefore, we excluded them from further downstream analyses. We display the posteriors of the remaining Neandertals in Supplementary Figures 70 - 79, finding overall little evidence of modern human or Denisovan admixture.

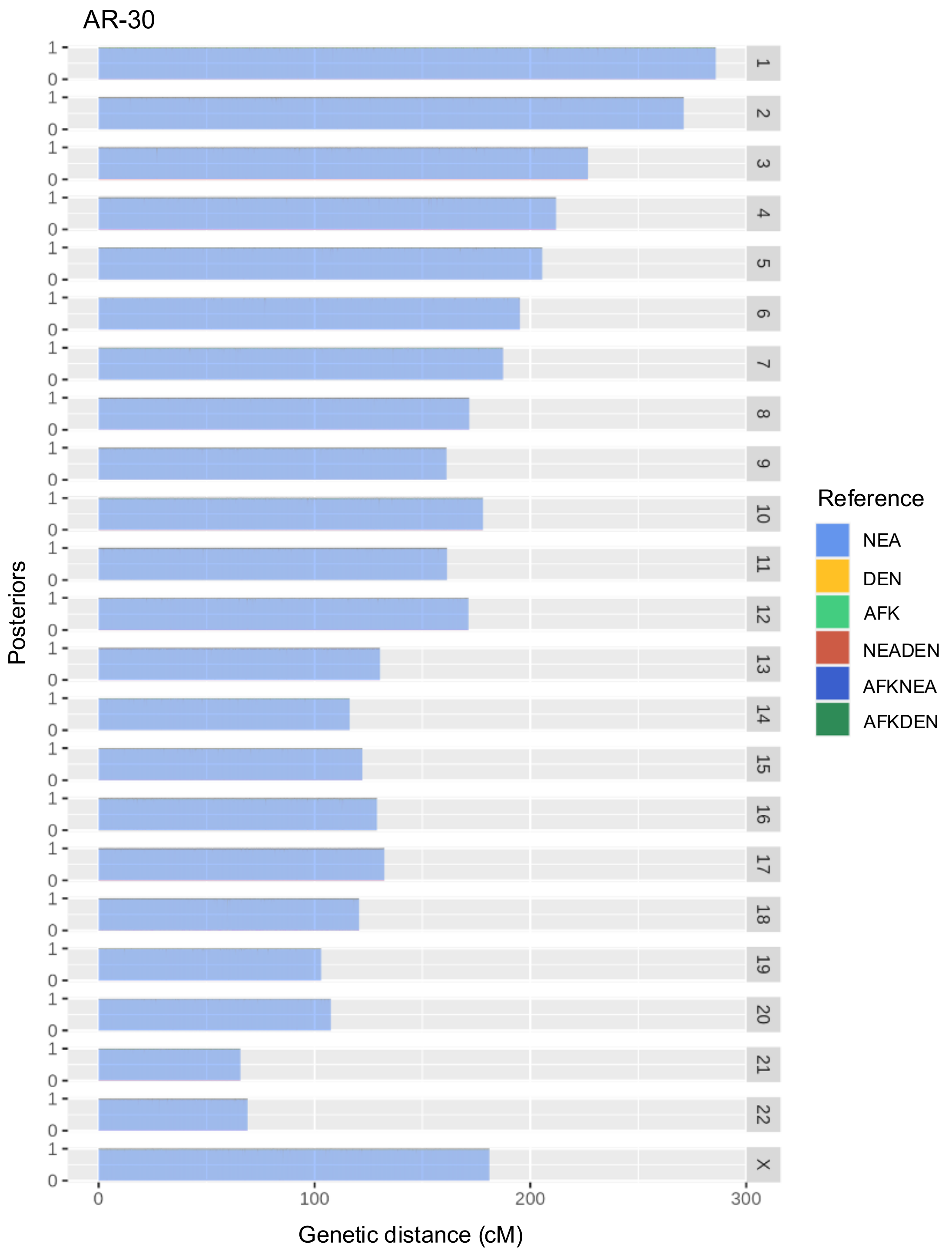

Supplementary Figure 70 Inferred local ancestry of AR-30, using *admixfrog*. The Y-axis indicates the posterior probability in each 10 bp bin, and the X-axis the genomic position in genetic distance (cM) across each chromosome. The coloured reference ancestries correspond to NEA = Neandertal, AFR = African, DEN = Denisovan, and combinations of the three in case of heterozygous fragments.

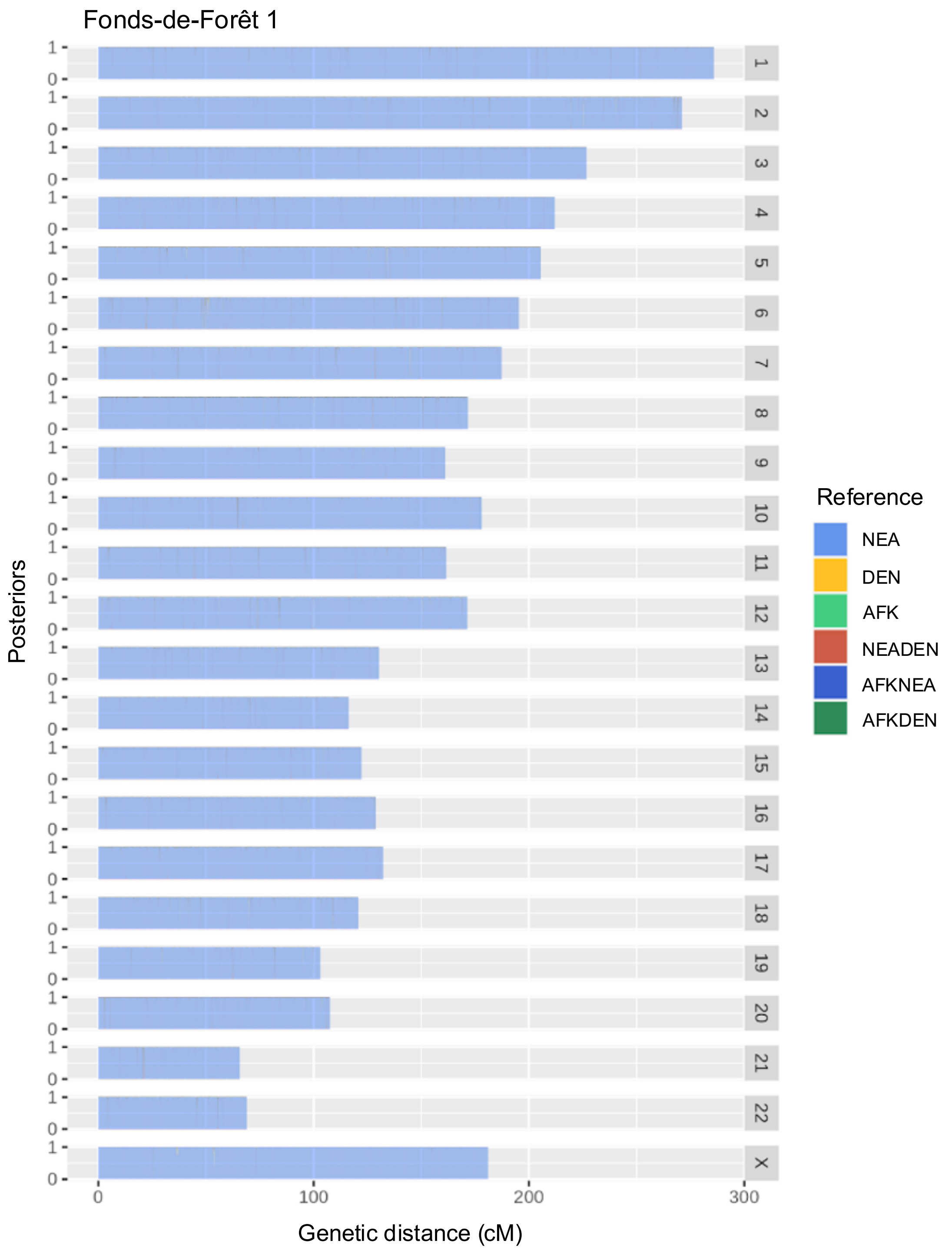

Supplementary Figure 71 Inferred local ancestry of Fonds-de-Forêt 1, using *admixfrog*. The Y-axis indicates the posterior probability in each 10 bp bin, and the X-axis the genomic position in genetic distance (cM) across each chromosome. The coloured reference ancestries correspond to NEA = Neandertal, AFR = African, DEN = Denisovan, and combinations of the three in case of heterozygous fragments.

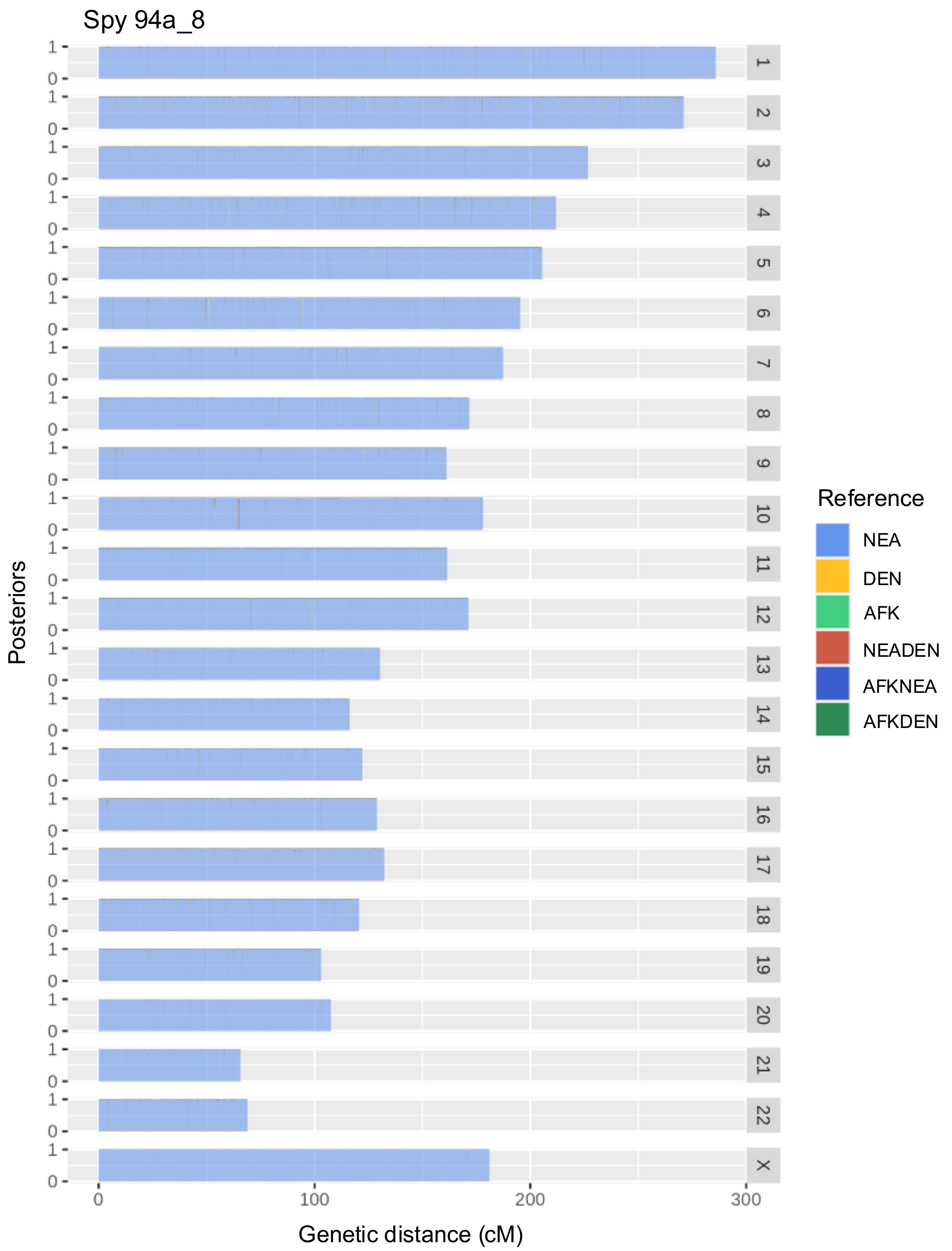

Supplementary Figure 72 Inferred local ancestry of Spy 94a_8, using *admixfrog*. The Y-axis indicates the posterior probability in each 10 bp bin, and the X-axis the genomic position in genetic distance (cM) across each chromosome. The coloured reference ancestries correspond to NEA = Neandertal, AFR = African, DEN = Denisovan, and combinations of the three in case of heterozygous fragments.

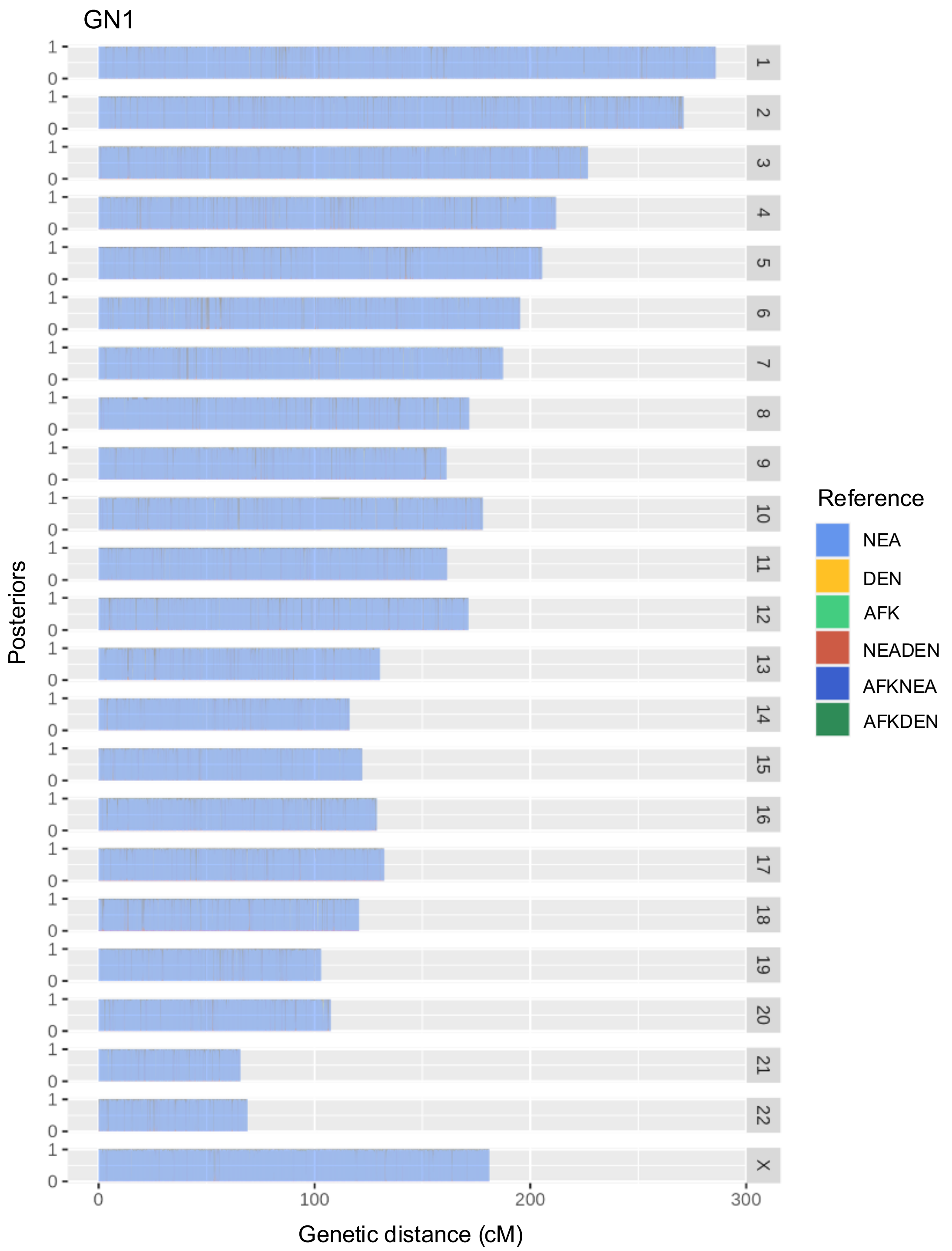

Supplementary Figure 73 Inferred local ancestry of GN1, using *admixfrog*. The Y-axis indicates the posterior probability in each 10 bp bin, and the X-axis the genomic position in genetic distance (cM) across each chromosome. The coloured reference ancestries correspond to NEA = Neandertal, AFR = African, DEN = Denisovan, and combinations of the three in case of heterozygous fragments.

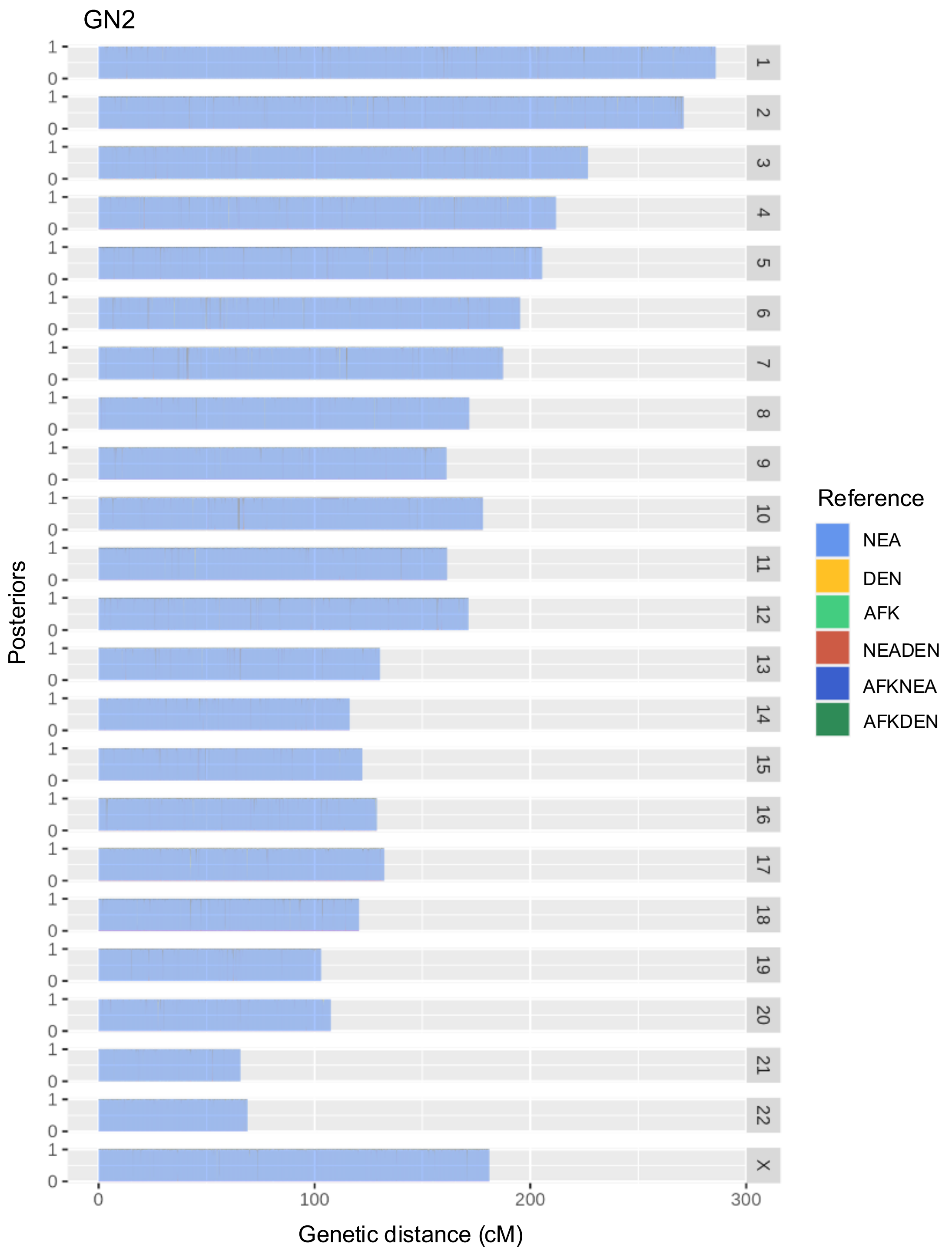

Supplementary Figure 74 Inferred local ancestry of GN2, using *admixfrog*. The Y-axis indicates the posterior probability in each 10 bp bin, and the X-axis the genomic position in genetic distance (cM) across each chromosome. The coloured reference ancestries correspond to NEA = Neandertal, AFR = African, DEN = Denisovan, and combinations of the three in case of heterozygous fragments.

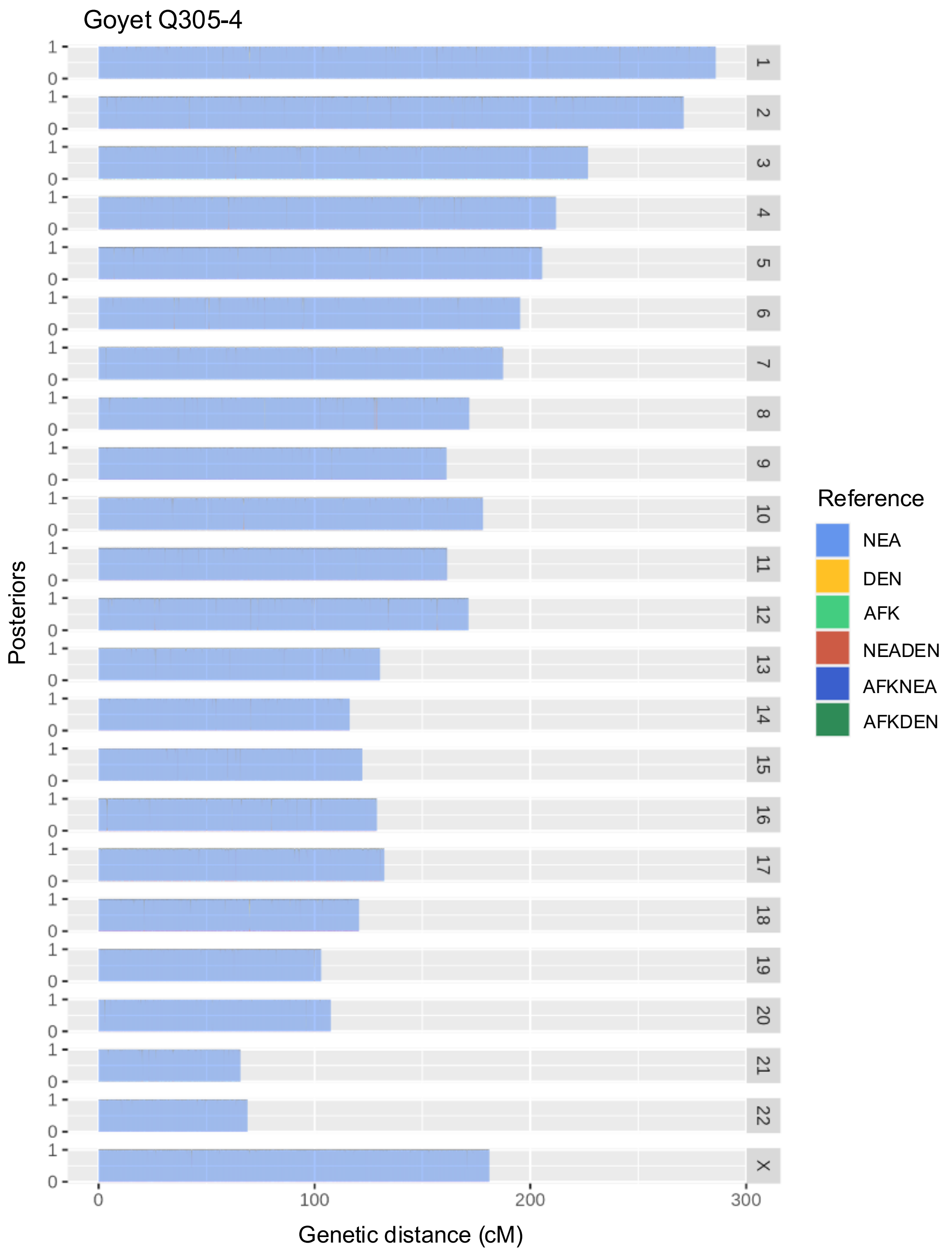

Supplementary Figure 75 Inferred local ancestry of Goyet Q305-4, using *admixfrog*. The Y-axis indicates the posterior probability in each 10 bp bin, and the X-axis the genomic position in genetic distance (cM) across each chromosome. The coloured reference ancestries correspond to NEA = Neandertal, AFR = African, DEN = Denisovan, and combinations of the three in case of heterozygous fragments.

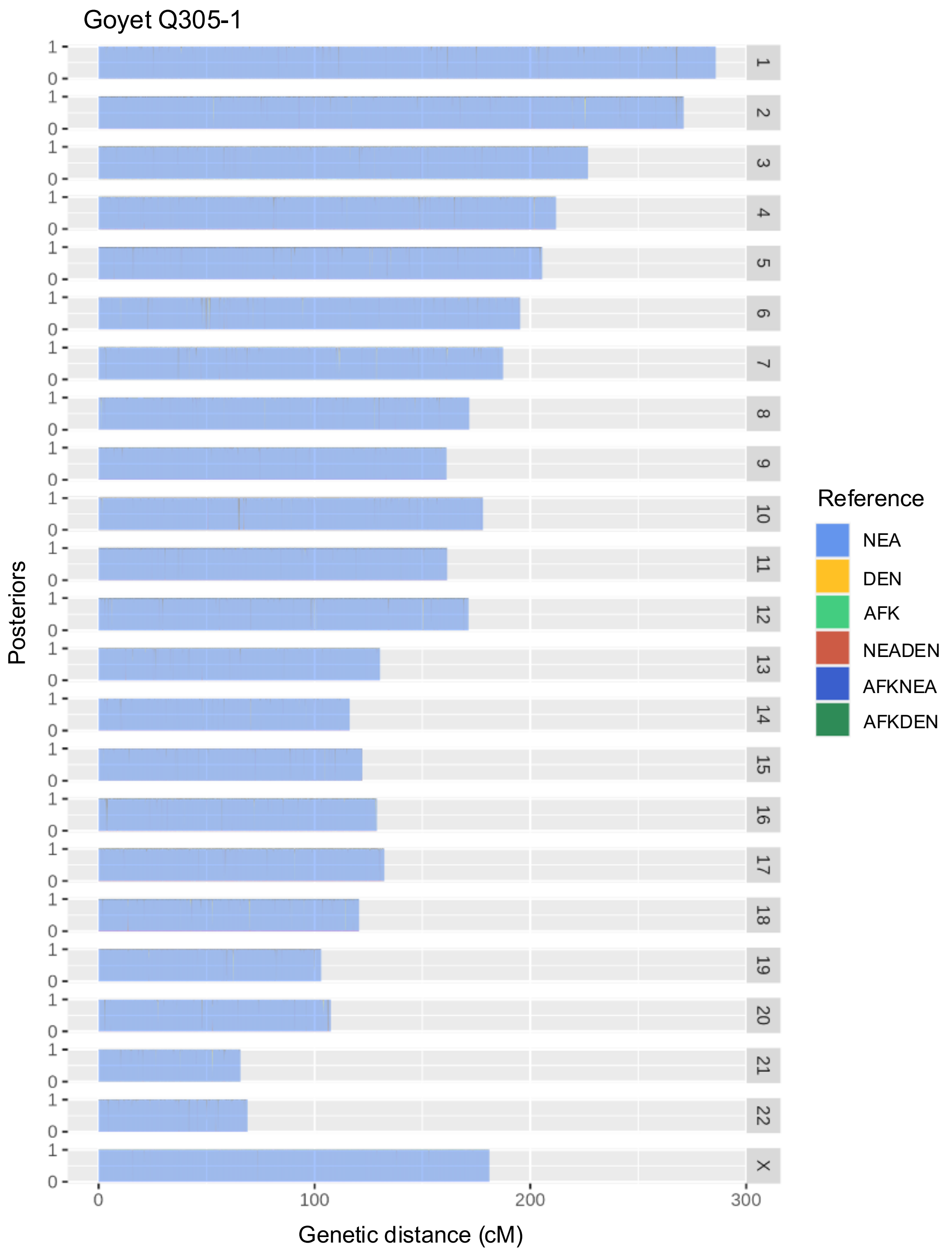

Supplementary Figure 76 Inferred local ancestry of Goyet Q305-1, using *admixfrog*. The Y-axis indicates the posterior probability in each 10 bp bin, and the X-axis the genomic position in genetic distance (cM) across each chromosome. The coloured reference ancestries correspond to NEA = Neandertal, AFR = African, DEN = Denisovan, and combinations of the three in case of heterozygous fragments.

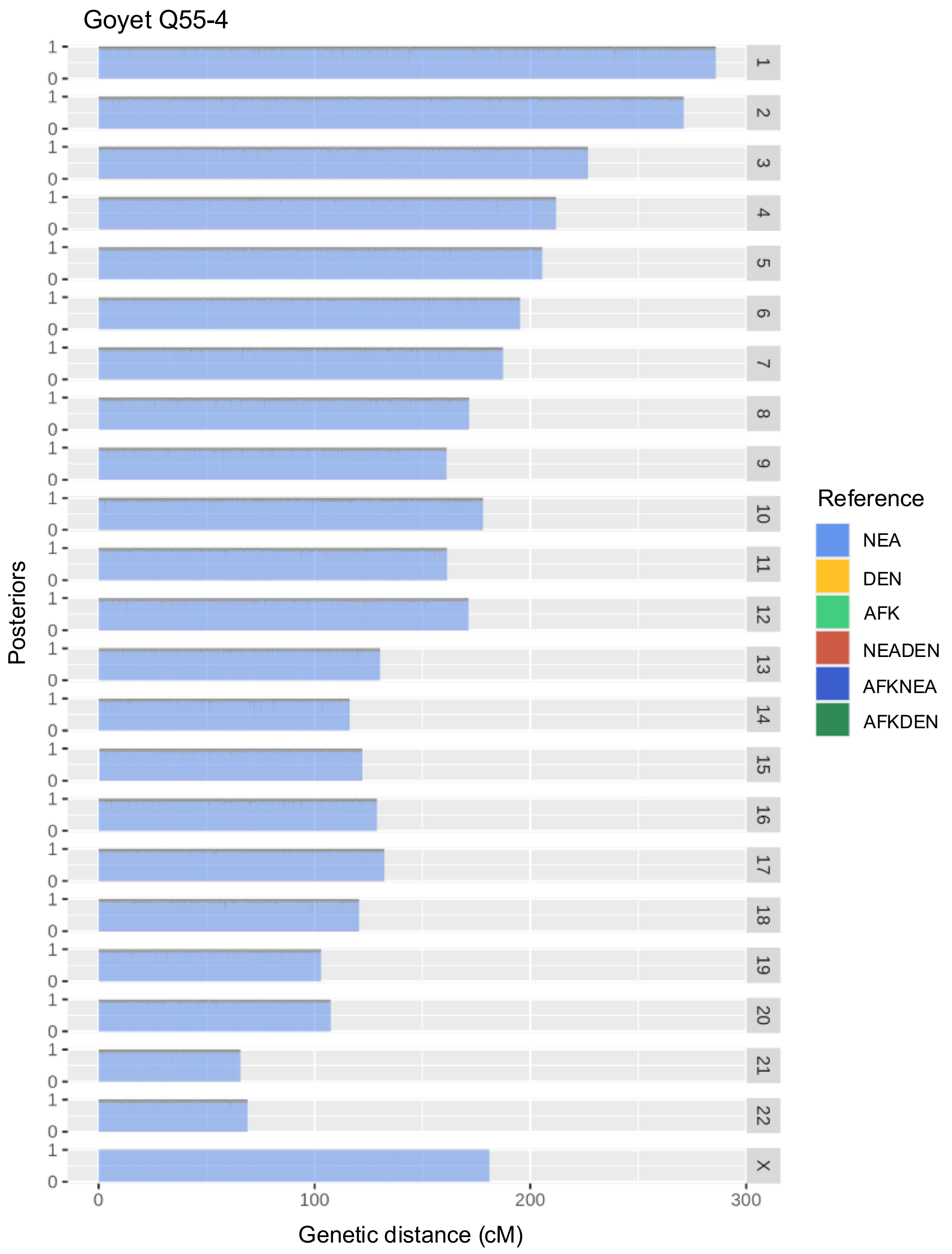

Supplementary Figure 77 Inferred local ancestry of Goyet Q55-4, using *admixfrog*. The Y-axis indicates the posterior probability in each 10 bp bin, and the X-axis the genomic position in genetic distance (cM) across each chromosome. The coloured reference ancestries correspond to NEA = Neandertal, AFR = African, DEN = Denisovan, and combinations of the three in case of heterozygous fragments.

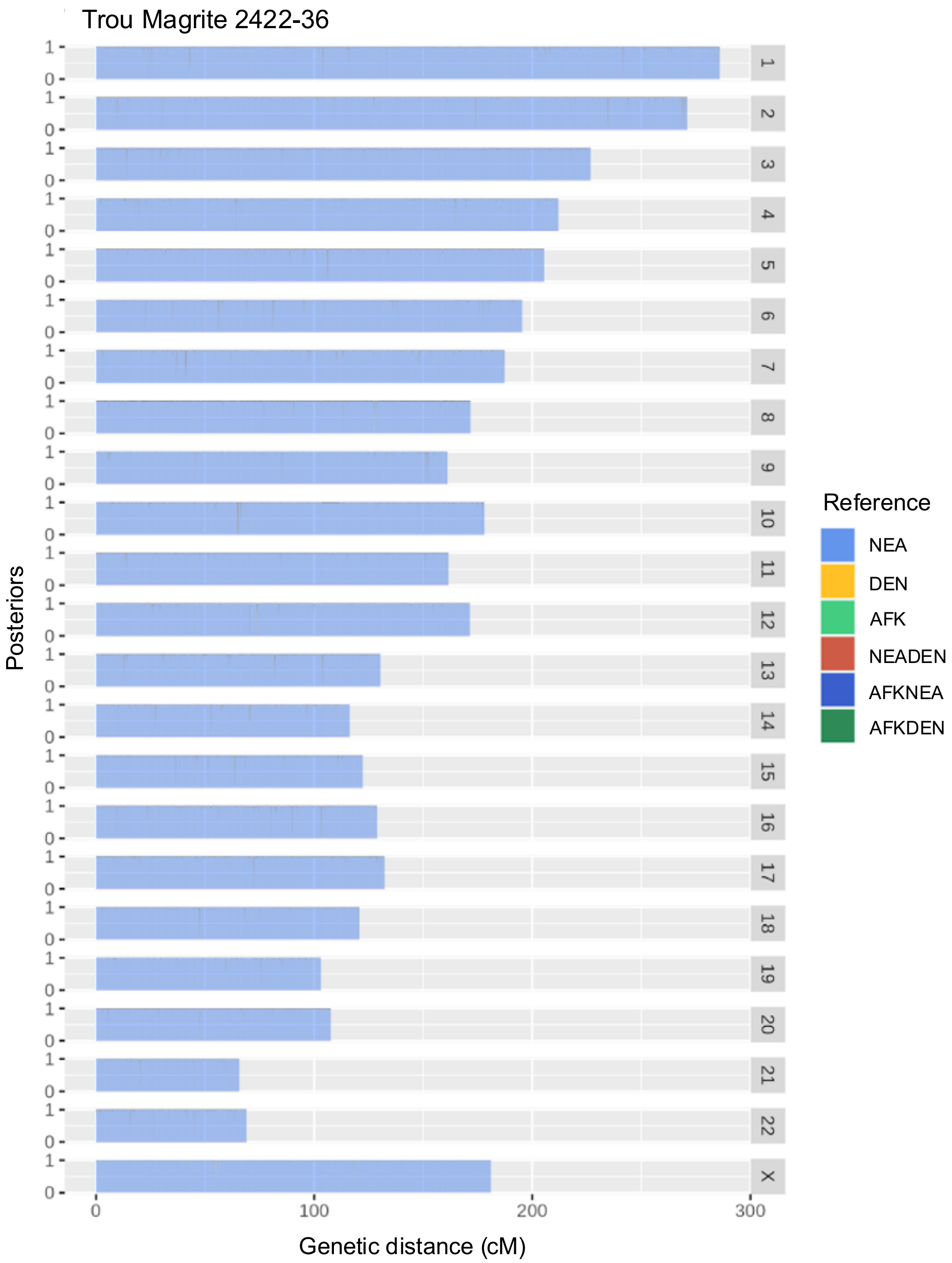

Supplementary Figure 78 Inferred local ancestry of Trou Magrite 2422-36, using *admixfrog*. The Y-axis indicates the posterior probability in each 10 bp bin, and the X-axis the genomic position in genetic distance (cM) across each chromosome. The coloured reference ancestries correspond to NEA = Neandertal, AFR = African, DEN = Denisovan, and combinations of the three in case of heterozygous fragments.

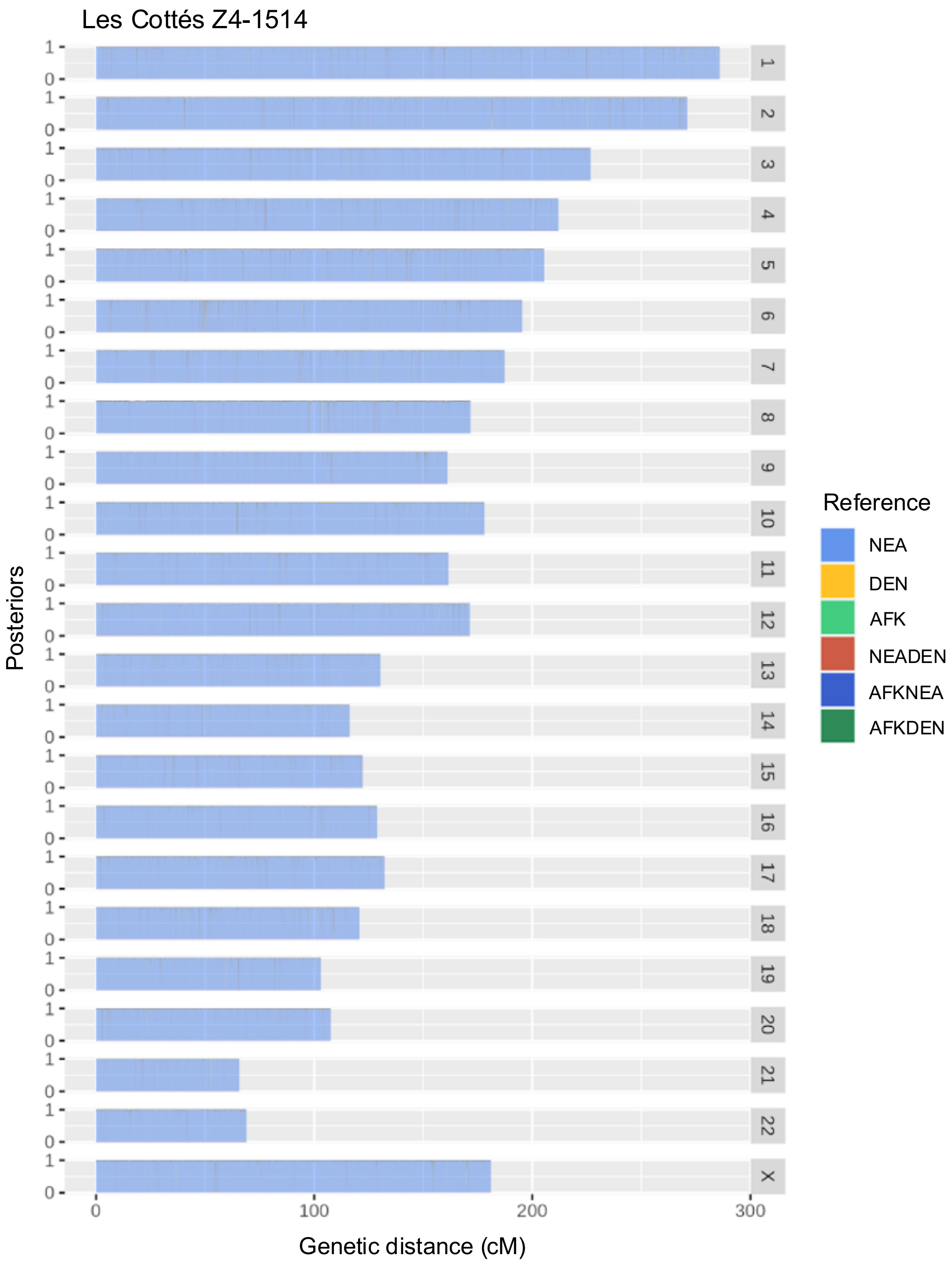

Supplementary Figure 79 Inferred local ancestry of Les Cottés Z4-1514, using *admixfrog*. The Y-axis indicates the posterior probability in each 10 bp bin, and the X-axis the genomic position in genetic distance (cM) across each chromosome. The coloured reference ancestries correspond to NEA = Neandertal, AFR = African, DEN = Denisovan, and combinations of the three in case of heterozygous fragments.

There were few instances of modern human segments longer than 0.2 cM that did not overlap the centromeres (https://hgdownload.soe.ucsc.edu/goldenPath/hg19/database/gap.txt.gz). The majority were found in the GN1 high-coverage genome, and sporadic cases in Goyet Q305-1, GN2 and Les Cottés Z4-1514 (Supplementary Table 36).

Supplementary Table 36: Modern human fragments called in the studied Neandertal genomes. Chr. – chromosome; bp – base pair; cM – centimorgan.

Sample	Chr.	Ancestry	Type	Physical coordinates (bp)	Map coordinates (cM)	Fragment length (cM)
GN1	10	AFR	Homozygous	36,889,240 - 38,324,913	64.61 - 65.02	0.41
GN1	2	AFR	Heterozygous	240,388,008 - 240,471,598	268.86 - 269.14	0.28
GN1	4	AFR	Heterozygous	166,621,042 - 166,734,132	172.81 - 173.03	0.22
GN1	5	AFR	Heterozygous	136,377,306 - 136,446,833	142.14 - 142.36	0.22
GN1	6	AFR	Heterozygous	32,968,017 - 33,246,770	50.73 - 51.05	0.32
GN1	7	AFR	Homozygous	22,526,627 - 22,779,032	36.79 - 37	0.21
GN1	7	AFR	Heterozygous	26,164,494 - 26,438,925	41.25 - 41.49	0.24
GN1	12	AFR	Heterozygous	77,803,586 - 77,853,029	93.93 - 94.2	0.27
GN1	17	AFR	Heterozygous	50,954,244 - 51,203,009	78.1 - 78.31	0.21
GN1	6	AFR	Heterozygous	24,251,391 - 24,627,544	47.43 - 47.75	0.32
Goyet Q305-1	10	AFR	Heterozygous	4,3224,839 - 43,752,672	65.16 - 65.37	0.2
GN2	6	AFR	Homozygous	3,7599,390 - 37,708,624	56.16 - 56.48	0.31
Les Cottés Z4-1514	1	AFR	Heterozygous	209,587,774 - 209,692,610	224.79 - 225.01	0.22
Les Cottés Z4-1514	10	AFR	Homozygous	36,889,240 - 38,372,047	64.61 - 65.03	0.41
Les Cottés Z4-1514	4	AFR	Heterozygous	64,128,302 - 64,743,952	77.52 - 77.85	0.33
Les Cottés Z4-1514	7	AFR	Heterozygous	79,226,679 - 79,687,884	93.08 - 93.29	0.21

We first checked whether the called modern human segment had been identified in all bones that we previously determined to belong to that same individual (Supplementary Section 14). This was not the case for GN2, where it had only been called in Goyet Q57-2, but not in Goyet Q57-1 (which has higher coverage) and Goyet Q57-3 (Supplementary Figures 80 - 81). Similarly, Goyet Q374a-1 and Goyet Q305-7 did not possess the putative introgressed modern human segments we found in the higher-coverage Goyet Q56-1 genome from the same individual, except for a fragment on chromosome 10 (Supplementary Figures 82 – 83).

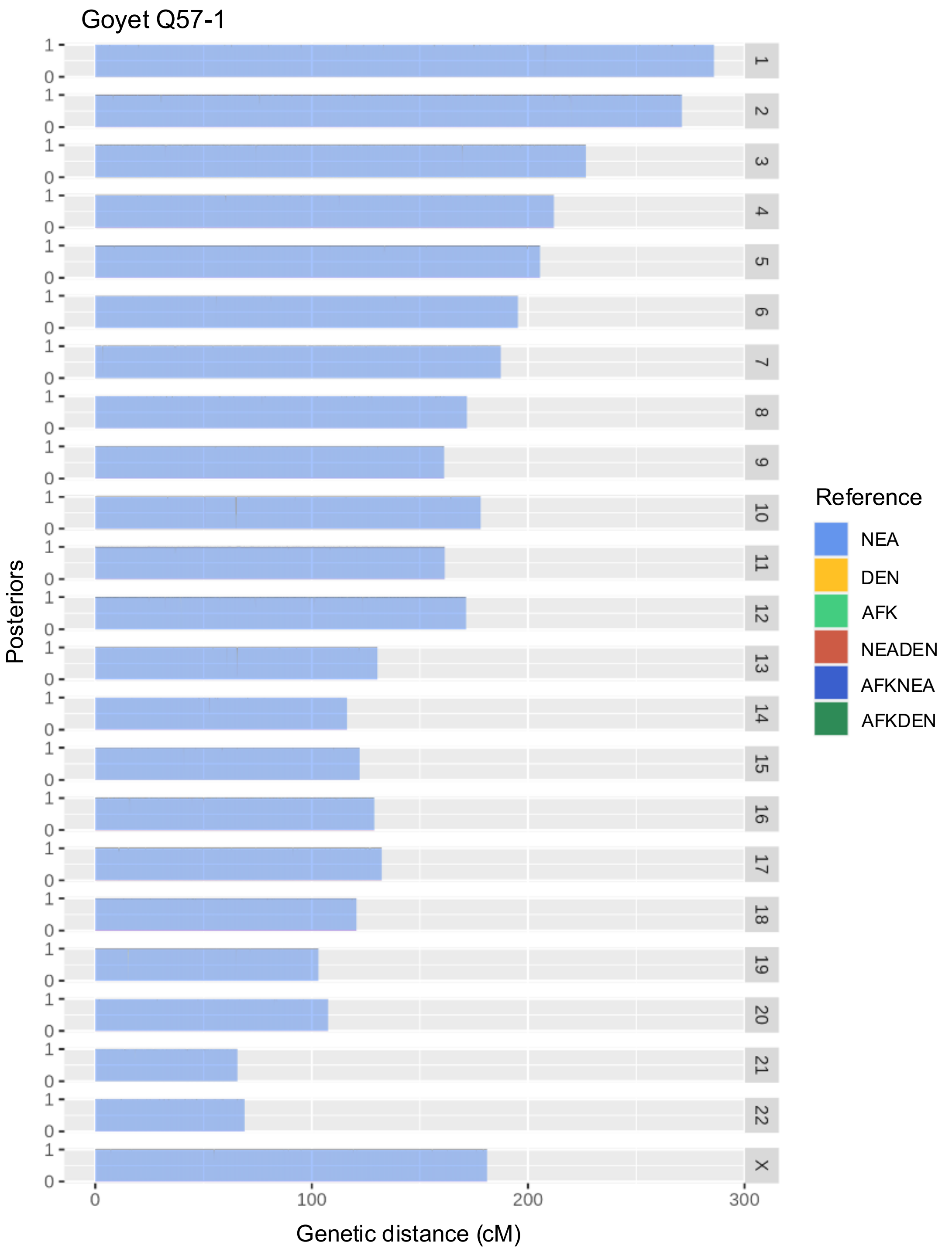

Supplementary Figure 80 Inferred local ancestry of Goyet Q57-1, using *admixfrog*. The Y-axis indicates the posterior probability in each 10 bp bin, and the X-axis the genomic position in genetic distance (cM) across each chromosome. The coloured reference ancestries correspond to NEA = Neandertal, AFR = African, DEN = Denisovan, and combinations of the three in case of heterozygous fragments.

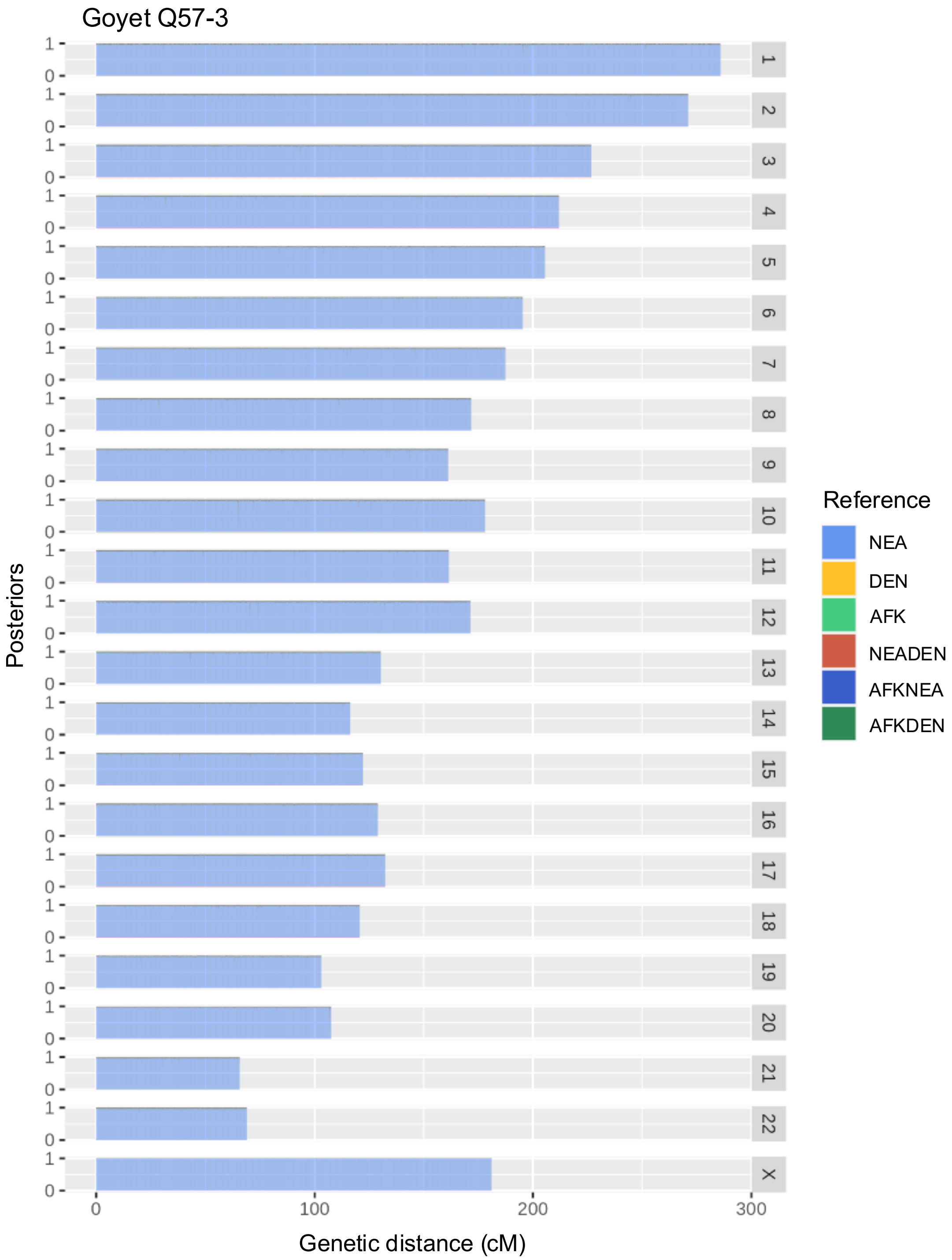

Supplementary Figure 81 Inferred local ancestry of Goyet Q57-3, using *admixfrog*. The Y-axis indicates the posterior probability in each 10 bp bin, and the X-axis the genomic position in genetic distance (cM) across each chromosome. The coloured reference ancestries correspond to NEA = Neandertal, AFR = African, DEN = Denisovan, and combinations of the three in case of heterozygous fragments.

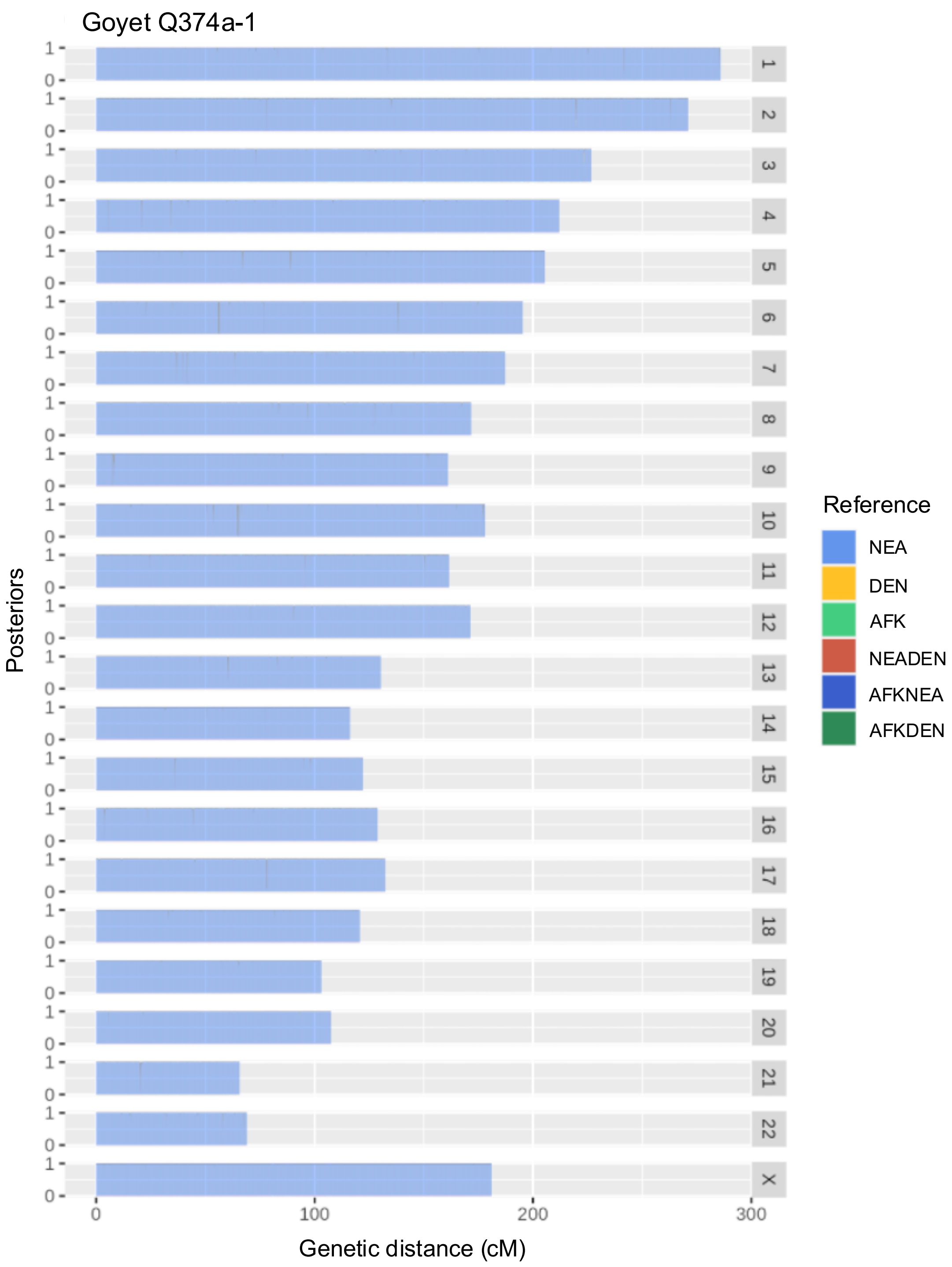

Supplementary Figure 82 Inferred local ancestry of Goyet Q374a-1, using *admixfrog*. The Y-axis indicates the posterior probability in each 10 bp bin, and the X-axis the genomic position in genetic distance (cM) across each chromosome. The coloured reference ancestries correspond to NEA = Neandertal, AFR = African, DEN = Denisovan, and combinations of the three in case of heterozygous fragments.

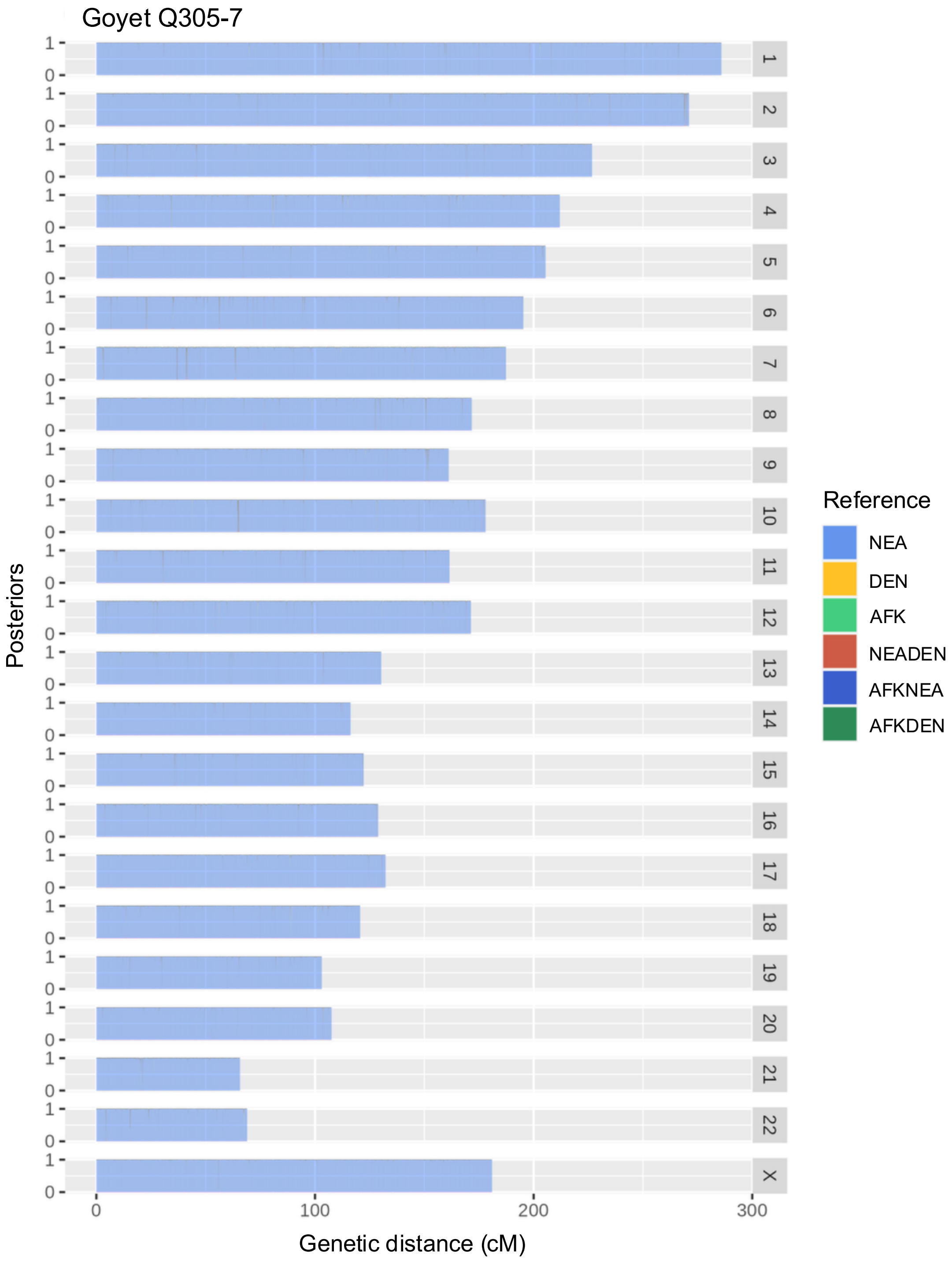

Supplementary Figure 83 Inferred local ancestry of Goyet Q305-7, using *admixfrog*. The Y-axis indicates the posterior probability in each 10 bp bin, and the X-axis the genomic position in genetic distance (cM) across each chromosome. The coloured reference ancestries correspond to NEA = Neandertal, AFR = African, DEN = Denisovan, and combinations of the three in case of heterozygous fragments.

To further gauge the driving factor for the "AFR", i.e., putatively modern human assignments, we compared the target data with the allele frequencies of each of the reference populations in the putative segments (Extended Data Fig. 8 and Supplementary Figure 84). In all the putative segments, we did not find any sites where Neandertals and modern humans are fixed for different alleles; rather all sites are variable in Neandertals, modern humans or both, and the modern human ancestry assignment had been solely based on differences in allele frequencies between modern humans and Neandertals

Given that all of the identified fragments are very short, inconsistently inferred, and close to the cut-off of 0.2cM, there are two possible explanations. One, they stem from an introgression event in the distant past, close to the detection limit, and not stemming from recent gene flow. One possibility is that these segments stem from an event shared with other Neandertals[13–15]. The other possibility is that these are false positives, due to incomplete lineage sorting, and can be explained by uncertainties in the recombination rate. In modern humans, recombination rates are known to be population-specific[16], and we know little about the exact recombination map in Neandertals, making it hard to validate these fragments. The finding that there are several segregating, but no fixed sites between Neandertals and modern humans, is consistent with this hypothesis.

In summary, while we identify a few candidate fragments for modern human ancestry in Late Neandertals, we are unable to validate them, and they are too short to stem from the recent out-of-Africa expansion of modern humans.

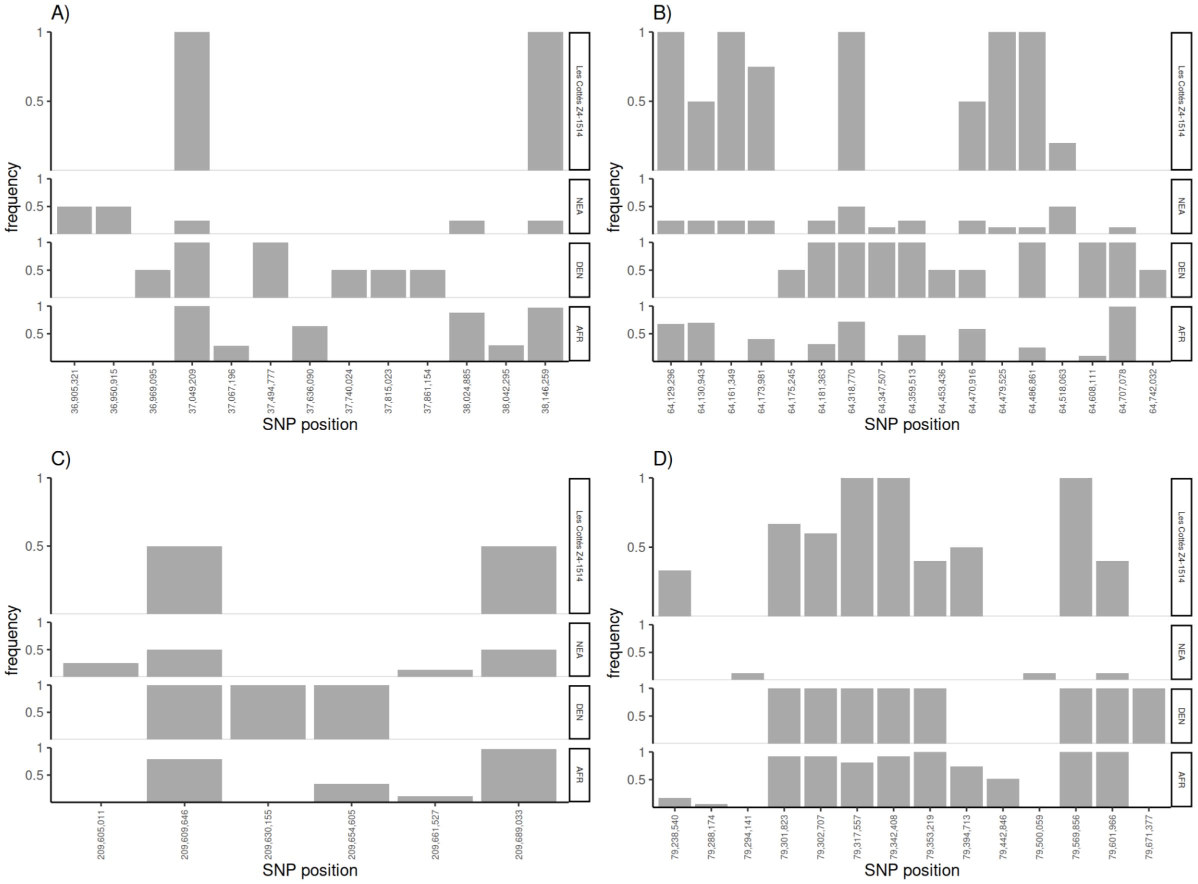

Supplementary Figure 84: Allelic frequencies at each position of the AFR segment identified in Les Cottés Z4-1514. A), B), C), and D) represent the different identified fragments.

The lack of convincing evidence for modern human introgression might be surprising given that temporally all the studied Neandertal remains stem from a period in which modern humans were already present in Northern Europe[17–19], and many examples exist of early Upper Palaeolithic humans with Neandertal ancestry segments in their genomes as a result of recent admixtures[3,4,20]. Some of the archaeological sites included in this study, namely the Troisième caverne of Goyet and Grotte du Renne (Arcy-sur-Cure), have yielded both Neandertal and modern human remains[21,22]. While in the first case the radiocarbon dates indicate that these two hominins were not contemporary, the modern human and Neandertal remains from Arcy-sur-Cure were found in the same Châtelperronian layer X, and have thus been interpreted as contemporaneous[22].

References

1. Iasi, L. N. M. *et al.* Neanderthal ancestry through time: Insights from genomes of ancient and present-day humans. *Science* **386**, eadq3010 (2024).

2. Sümer, A. P. *et al.* Earliest modern human genomes constrain timing of Neanderthal admixture. *Nature* **638**, 711–717 (2025).

3. Prüfer, K. *et al.* A genome sequence from a modern human skull over 45,000 years old from Zlatý kůň in Czechia. *Nat. Ecol. Evol.* **5**, 820–825 (2021).

4. Hajdinjak, M. *et al.* Initial Upper Palaeolithic humans in Europe had recent Neanderthal ancestry. *Nature* **592**, 253–257 (2021).

5. Gravel, S. Population Genetics Models of Local Ancestry. *Genetics* **191**, 607–619 (2012).

6. Peter, B. M. 100,000 years of gene flow between Neandertals and Denisovans in the Altai mountains. Preprint at https://doi.org/10.1101/2020.03.13.990523 (2020).

7. Prüfer, K. snpAD: an ancient DNA genotype caller. *Bioinformatics* **34**, 4165–4171 (2018).

8. Prüfer, K. *et al.* A high-coverage Neandertal genome from Vindija Cave in Croatia. *Science* **358**, 655–658 (2017).

9. Prüfer, K. *et al.* The complete genome sequence of a Neanderthal from the Altai Mountains. *Nature* **505**, 43–49 (2014).

10. Mafessoni, F. *et al.* A high-coverage Neandertal genome from Chagyrskaya Cave. *Proc. Natl. Acad. Sci.* **117**, 15132–15136 (2020).

11. The 1000 Genomes Project Consortium *et al.* A global reference for human genetic variation. *Nature* **526**, 68–74 (2015).

12. Meyer, M. *et al.* A High-Coverage Genome Sequence from an Archaic Denisovan Individual. *Science* **338**, 222–226 (2012).

13. Kuhlwilm, M. *et al.* Ancient gene flow from early modern humans into Eastern Neanderthals. *Nature* **530**, 429–433 (2016).

14. Peyrégne, S., Kelso, J., Peter, B. M. & Pääbo, S. The evolutionary history of human spindle genes includes back-and-forth gene flow with Neandertals. *eLife* **11**, e75464 (2022).

15. Li, L., Comi, T. J., Bierman, R. F. & Akey, J. M. Recurrent gene flow between Neanderthals and modern humans over the past 200,000 years. *Science* **385**, eadi1768 (2024).

16. Zhou, Y., Browning, B. L. & Browning, S. R. Population-Specific Recombination Maps from Segments of Identity by Descent. *Am. J. Hum. Genet.* **107**, 137–148 (2020).

17. Abrams, G. *et al.* Investigating the co-occurrence of Neanderthals and modern humans in Belgium through direct radiocarbon dating of bone implements. *J. Hum. Evol.* **186**, 103471 (2024).
18. Hublin, J.-J. The modern human colonization of western Eurasia: when and where? *Quat. Sci. Rev.* **118**, 194–210 (2015).
19. Mylopotamitaki, D. *et al.* Homo sapiens reached the higher latitudes of Europe by 45,000 years ago. *Nature* **626**, 341–346 (2024).
20. Fu, Q. *et al.* An early modern human from Romania with a recent Neanderthal ancestor. *Nature* **524**, 216–219 (2015).
21. Posth, C. *et al.* Pleistocene Mitochondrial Genomes Suggest a Single Major Dispersal of Non-Africans and a Late Glacial Population Turnover in Europe. *Curr. Biol.* **26**, 827–833 (2016).
22. Gicqueau, A. *et al.* Anatomically modern human in the Châtelperronian hominin collection from the Grotte du Renne (Arcy-sur-Cure, Northeast France). *Sci. Rep.* **13**, 12682 (2023).
