# Supplementary material for: Genetic diversity of late Neanderthals in northwestern Europe
Source: Nature. 2026 Jun 24;655(8122):409–17. doi: 10.1038/s41586-026-10625-1 (PMC13345965; doi:10.1038/s41586-026-10625-1)
Supplement: Supplementary file 2 — Reporting Summary [file 41586_2026_10625_MOESM2_ESM.pdf]

nature portfolio

Corresponding author(s): Bossoms Mesa, Alba; Hajdinjak, Mateja; Kelso, Janet; Peter, Benjamin M.

Last updated by author(s): 29th April 2026

Reporting Summary

Nature Portfolio wishes to improve the reproducibility of the work that we publish. This form provides structure for consistency and transparency in reporting. For further information on Nature Portfolio policies, see our Editorial Policies and the Editorial Policy Checklist.

Statistics

For all statistical analyses, confirm that the following items are present in the figure legend, table legend, main text, or Methods section.

n/a	Confirmed	
☐	☒	The exact sample size (*n*) for each experimental group/condition, given as a discrete number and unit of measurement
☐	☒	A statement on whether measurements were taken from distinct samples or whether the same sample was measured repeatedly
☐	☒	The statistical test(s) used AND whether they are one- or two-sided<br>*Only common tests should be described solely by name; describe more complex techniques in the Methods section.*
☒	☐	A description of all covariates tested
☒	☐	A description of any assumptions or corrections, such as tests of normality and adjustment for multiple comparisons
☐	☒	A full description of the statistical parameters including central tendency (e.g. means) or other basic estimates (e.g. regression coefficient) AND variation (e.g. standard deviation) or associated estimates of uncertainty (e.g. confidence intervals)
☐	☒	For null hypothesis testing, the test statistic (e.g. *F*, *t*, *r*) with confidence intervals, effect sizes, degrees of freedom and *P* value noted<br>*Give P values as exact values whenever suitable.*
☐	☒	For Bayesian analysis, information on the choice of priors and Markov chain Monte Carlo settings
☒	☐	For hierarchical and complex designs, identification of the appropriate level for tests and full reporting of outcomes
☒	☐	Estimates of effect sizes (e.g. Cohen's *d*, Pearson's *r*), indicating how they were calculated

Our web collection on statistics for biologists contains articles on many of the points above.

Software and code

Policy information about availability of computer code

Data collection	No software was used for data collection.
Data analysis	All software packages used for analysis are cited in the Online Methods section and in the Supplementary Information and all used packages are publicly available:<br><br>All sequence data processing included adapter trimming and read merging using leeHom (version 1.2.18, https://bioinf.eva.mpg.de/leehom/), BWA (version 0.5.10-evan.9-1-g44db244, https://github.com/mpieva/network-aware-bwa), bam-rmdup (version 0.6.3, https://github.com/mpieva/biohazard-tools).<br><br>For mitochondrial DNA analysis: mafft (version 7.453, https://github.com/GSLBiotech/mafft) and BEAST2 (version 2.1.3, https://github.com/CompEvol/beast2), FigTree (ersion 1.4.4, http://tree.bio.ed.ac.uk/software/figtree) .<br><br>For nuclear DNA analysis: samtools (version 1.3.1-21, https://github.com/samtools), AuthentiCT (version 1.0.1, https://github.com/StephanePeyregne/AuthentiCT), snpAD (version 0.3.11, http://bioinf.eva.mpg.de/snpAD), PSMC (version 0.6.5, implemented in a pipeline for ancient DNA data in https://github.com/StephanePeyregne/calibratePSMC), Ensembl Variant Effect Predictor (version 112, https://www.ensembl.org/info/docs/tools/vep/index.html), ABAEnrichment R package (version 1.13.3, https://github.com/sgrote/ABAEnrichment), GOfuncR R package (version 1.23.2, https://github.com/sgrote/GOfuncR), OrganismDb R package (version 1.14.1, https://www.rdocumentation.org/packages/OrganismDbi/versions/1.14.1), FUNC (version 0.4.9, https://func.eva.mpg.de), gwascat R package (version 2.42.1, https://bioconductor.org/packages/gwascat), rethinking R package (version 2.13, https://github.com/rmcelreath/rethinking), picard LiftoverVcf (version 2.18.29, https://gatk.broadinstitute.org/hc/en-us/articles/360037060932-LiftoverVcf-Picard), KIN (version 3.1.3, https://github.com/DivyaratanPopli/Kinship_Inference), admixr (version 0.9.1, https://github.com/bodkan/admixr), ADMIXTOOLS (version 7.0,

https://github.com/DReichLab/AdmixTools) and admixfrog (version 0.7.2, https://github.com/BenjaminPeter/admixfrog).

For manuscripts utilizing custom algorithms or software that are central to the research but not yet described in published literature, software must be made available to editors and reviewers. We strongly encourage code deposition in a community repository (e.g. GitHub). See the Nature Portfolio guidelines for submitting code & software for further information.

Data

Policy information about availability of data

All manuscripts must include a data availability statement. This statement should provide the following information, where applicable:

- Accession codes, unique identifiers, or web links for publicly available datasets
- A description of any restrictions on data availability
- For clinical datasets or third party data, please ensure that the statement adheres to our policy

The Neandertal sequences from this study have been deposited in the European Nucleotide Archive under accession number PRJEB98484, to be released on the 24th of April. We have also uploaded all mitochondrial sequences fasta files and genotype calls of the nuclear genomes to the Edmond repository (https://doi.org/10.17617/3.F9N73O).

Private variant analyses of GN1 relied on the datasets gnomAD genomes (version 3.1.2) and gnomAD exomes (version 4.0), available at https://gnomad.broadinstitute.org; the Human Phenotype Ontology (HPO) available at https://hpo.jax.org; the Gene Ontology (GO) at https://current.geneontology.org/ontology/; as well as the Allen Brain Atlas, available at http://www.brain-map.org .

Comparative data of present-day human genomes used in this project include the "Simons Genome Diversity Project" available at: https://www.simonsfoundation.org/simons-genome-diversity-project/ and from the "1000 Genome project (phase 3)" available through the International Genome Sample Resource (IGSR) at: https://www.internationalgenome.org .

Research involving human participants, their data, or biological material

Policy information about studies with human participants or human data. See also policy information about sex, gender (identity/presentation), and sexual orientation and race, ethnicity and racism.

Reporting on sex and gender	N/A
Reporting on race, ethnicity, or other socially relevant groupings	N/A.
Population characteristics	N/A.
Recruitment	N/A.
Ethics oversight	N/A.

Note that full information on the approval of the study protocol must also be provided in the manuscript.

Field-specific reporting

Please select the one below that is the best fit for your research. If you are not sure, read the appropriate sections before making your selection.

☒ Life sciences ☐ Behavioural & social sciences ☐ Ecological, evolutionary & environmental sciences

For a reference copy of the document with all sections, see nature.com/documents/nr-reporting-summary-flat.pdf

Life sciences study design

All studies must disclose on these points even when the disclosure is negative.

Sample size

The number of genomes analysed in this study was determined by identifying those specimens that had sufficient levels of ancient DNA preservation for downstream sequencing and analysis. Neandertal specimens are extremely scarce, and preservation greatly varies. For this reason, depending on the case the data that could be generated varied:

- 34 different specimens from tfrom seven sites in the Meuse Basin (Goyet, Spy, Couvin, Trou Magrite, Engis, Walou and Fonds-de-Forêt) and two sites in France (Saint-Césaire and Arcy-sur-Cure) were screened for ancient DNA preservation.
- One specimen, Goyet Q56-1, had exceptional DNA preservation, which enabled us to generate a 22.4 fold coverage genome.
- 19 skeletal remains from six archaeological sites (Goyet, Spy, Arcy-sur-Cure, Trou Magrite, Fonds-de-Forêt and Les Cottés) had sufficient DNA preservation to enable the sequencing nuclear DNA analysis after enriching with the ArchaicPlus array. Moreover, of these, three new males were identified and therefore were also enriched for Y-chromosome sequences.
- Six additional specimens produced mitochondrial DNA data but not nuclear DNA data could be recovered.

All of this is illustrated in Figure 1 of the manuscript.

Data exclusions	We used pre-established criteria in ancient DNA research of excluding sequences form the sequencing data that did not map to the human genome, sequences that were shorter than 35 base pairs and sequences mapping with a low mapping quality (<25); all of which are excluded to avoid incorporating sequences that are not endogenous to the individual sequenced. For nuclear DNA analysis, a threshold of 2% present-day human contamination was established for further filtering. For mitochondrial DNA analysis, we compared two different thresholds of present-day human contamination (10% and 25%) when reconstructing the mitochondrial genome sequences. Libraries that did not pass these contamination estimate thresholds were excluded from the study.
Replication	When possible, we took multiple samples from the same specimen to generate independent single-stranded DNA libraries. The results of reproducibility of the data generation and analyses are reported across the Supplementary Tables 1,2,3 and 5. To allow reproducibility of the downstream analyses, all filtering steps and the comparative data used in this study are detailed in the Online Methods and the Supplementary Information. Moreover, all of the data obtained in this study can be downloaded from the repositories indicated in the "Data availability" statement.
Randomization	Randomization is not relevant to this study because it does not involve experimental assignment or intervention, but rather the analysis of rare ancient specimens. In ancient DNA research, sample selection is inherently constrained by DNA preservation and availability, not by randomized design. Accordingly, all specimens listed in the “Sample size” section were screened for endogenous DNA content, and genome-wide analyses were conducted on those that met established quality thresholds. This preservation-driven inclusion criterion is standard in the field and precludes the application of randomization.
Blinding	Blinding was not relevant as we sampled ancient hominin specimens that were selected for this study based on their archaeological context (i.e. age and provenance), thus blinding would be inappropriate given the scarcity and the value of the sampled material. Blinding in downstream analyses was not relevant given that we analysed genome-wide data of the Neandertal specimens in relation to publicly available datasets of present-day and ancient human genomes.

Reporting for specific materials, systems and methods

We require information from authors about some types of materials, experimental systems and methods used in many studies. Here, indicate whether each material, system or method listed is relevant to your study. If you are not sure if a list item applies to your research, read the appropriate section before selecting a response.

Materials & experimental systems

n/a	Involved in the study
☒	☐ Antibodies
☒	☐ Eukaryotic cell lines
☐	☒ Palaeontology and archaeology
☒	☐ Animals and other organisms
☒	☐ Clinical data
☒	☐ Dual use research of concern
☒	☐ Plants

Methods

n/a	Involved in the study
☒	☐ ChIP-seq
☒	☐ Flow cytometry
☒	☐ MRI-based neuroimaging

Palaeontology and Archaeology

Specimen provenance	All permissions were granted by our collaborators that are also listed as co-authors on this manuscript, and with the aim of conducting genetic analyses on the sampled Neandertal material.<br>Specimen of Saint Cesaire was sampled in 2006 and 2014, subset of the specimens from Goyet was sampled in 2014, the specimens of Arcy-sur-Cure and Les Cottes were sampled in 2015, and the specimens of Couvin, Engis and Walou were sampled in 2017, all in the clean room facility of the Max Planck Institute for Evolutionary Anthropology in Leipzig, Germany. The remainder of the specimens from Goyet, as well as Spy and Trou Magrite, were sampled in 2015 and 2016 in the clean room facility of the Royal Belgian Institute of Natural Sciences in Brussels, Belgium. The specimen from Fonds-de-Forêt was sampled in 2019, also in the clean room facility of the Royal Belgian Institute of Natural Sciences in Brussels.
Specimen deposition	All Neandertal specimens (bones, bone fragments and teeth) are deposited in their respective institutions in Belgium and France. Scientists wanting to conduct research on these specimens should obtain permits from the institutions housing the specimens.
Dating methods	No new archaeological dates are provided in this study. All of the calibrated dates used as priors for the bayesian genetic dating are described in Supplementary Tables 2.7 and 2.8

☒ Tick this box to confirm that the raw and calibrated dates are available in the paper or in Supplementary Information.

Ethics oversight	All approvals for specimen sampling have been obtained from the relevant institutions.

Note that full information on the approval of the study protocol must also be provided in the manuscript.

Plants

Seed stocks	N/A.
Novel plant genotypes	N/A.
Authentication	N/A.
