# Supplementary material for: Genetic diversity of late Neanderthals in northwestern Europe
Source: Nature. 2026 Jun 24;655(8122):409–17. doi: 10.1038/s41586-026-10625-1 (PMC13345965; doi:10.1038/s41586-026-10625-1)
Supplement: Supplementary file 12 — Peer Review File [file 41586_2026_10625_MOESM12_ESM.pdf]

nature portfolio

Peer Review File

Genetic diversity of the late Neandertals of North-Western Europe

Corresponding Author: Ms Alba Bossoms Mesa

Any redactions in this file are there to maintain patient confidentiality, the confidentiality of unpublished data, or to remove third-party material.

This file contains all reviewer reports in order by version, followed by all author rebuttals in order by version.

Version 0:

Reviewer comments:

Referee #1

(Remarks to the Author)

This paper is interesting for a number of reasons. First, from Eastern Neanderthals, there were genetic observations of endogamy and even consanguinity (the later only in Altai Neandertal), as well as findings of close kin relations within family groups (like in Chagyrskaya). However, it was difficult to know if these traits could be generalized, beyond the marginal, eastern range of Neanderthals. Also, the recent genome of Thorin Neanderthal in France, suggested a potential geographic structure among Late Neanderthals that could preserve some of the prior diversity, largely erased by a migration of eastern Neanderthals (around 105 ka, if we take the reference from the sediment from Galería de las Estatuas). The current work not only adds data from 27 new Neanderthals (one of them at high coverage, accounting now for four such individuals), but also corroborates and clarifies the potential geographical pattern and provides data from North-Western Europe, thus balancing the existing genetic data that until now was clearly shifted to the east (likely because of DNA preservation reasons). Interestingly, these new data do not show evidence neither for endogamy nor consanguinity; on the contrary, these Late Neanderthals lived in diverse and larger, connected group networks, even when modern humans were already impacting in the Middle East and even Eastern Europe. I think it is a work that has some interesting consequences that will need to be further explored from climatic, morphological and maybe even cultural data, and thus it can be of interest for a wide range of scientists.

Minor Points

I find the paragraph between lines 132-143 quite difficult to follow. I think I got the idea that a fragment from Tibia III proved to belong in fact to the individual represented by fragments labelled GN2 (Goyet Q57-1, Q57-2 and Q57-3). Previously Goyet Q305-7 and Q374a-1 were determined to be genetically identical. In Line 139 it is said "As Goyet Q54-4 and Goyet Q374a-1 were genetically distinct…"; I suppose the sentence should work also like this: "As Goyet Q54-4 was genetically distinct to both Goyet Q374a-1 and Goyet Q305-7…". If this is correct, I think the authors should explicitly say that Tibia III reconstruction has joined fragments from two? different individuals (it is unclear for the reader how many fragments have been used to reconstruct Tibia III -could it be more than 2?-). Anyway, this section could be clarified a bit for the general reader.

Line 153-154; this sentence is difficult to understand. If all sequenced individuals are unrelated each other, it is clear that the mother of neonate Q305-1 have not been sampled.

One of the most interesting findings is the genetic structure among Late Neandertals that was suggested in the Thorin's paper. Is this something that can be observed at a morphological level? While I assume the remains are too fragmentary to give a conclusive answer, I don't know, maybe there are morphological traits, for instance in dental morphology, that could correlate with these results. That is, are there any traits that could be common to Couvin, Thorin, Stajnia and Mezmaiskaya 1 and 3? Because if there is any hint of differentiation, we could screen the rest of the Neandertal fossil record, including remains with no DNA preserved, that could help us to understand the geographic distribution of this potential population

structure. The dental morphology of Thorin (Fuch et al 2024, JHE), does not seem to show obvious differences with Late Neanderthal teeth (with maybe the exception of large premolar roots, according to the authors), but I am unfamiliar with the rest of remains from this potential mtDNA clade.

The observation that no gene flow from modern humans to late Neandertals seem to exist has been postulated before. In Lalueza-Fox (2019) “Neanderthal assimilation?” Nat Ecol Evol, we can read: “Remarkably, the opposite signal -gene flow from Late Pleistocene modern humans into the late Neanderthals- has not been detected so far. It might be that modern humans could tolerate hybrids but Neanderthals could not” (the second sentence is speculative, of course, and there are alternative explanations). I think referencing it could provide previous context for this ongoing observation that stands in sharp contrast with what is seen in early modern humans.

Referee #2

(Remarks to the Author)
please see comments below

Referee #3

(Remarks to the Author)
This manuscript analyzes new genomic data from several late Neanderthals from northwest Europe. Previously, this region has been studied using a handful of low-coverage genomes. This paper introduces additional samples and re-sequences some of the previously available samples, including a new high-coverage genome. These genomic data are a valuable resource for the community. These analyses are possible through the development of new laboratory and computational methods tailored to highly fragmented ancient DNA. The methods are appropriate and clearly described. Further, the authors integrate the genomic results with archaeological data where possible.

I was very excited to read this manuscript. However, while the new analyses quantify Neanderthal variation and allele sharing during this period at finer resolution than previously possible, it is often not clear how the results move beyond confirming what was already known or strongly suspected. In a way, it is wonderful that we now know so much about Neanderthal genomic variation that a new high-coverage genome does not immediately yield fundamental new insights. Nonetheless, I think these new data have great utility. Beyond a few minor technical questions and the need to improve the presentation (especially of figures), my main comments focus on encouraging the authors to: 1) clarify how the results fill existing gaps in knowledge and 2) perform further analyses of these new genomes to yield insights of broad interest.

In that spirit, I highlight several results where the authors could clarify and expand on what we learn (or even could expect to learn) about Neanderthals from the additional genomes:

- The kinship and diversity results largely confirm what we know from Vindija 33.19. We expect the individuals here to either be more like Chagyrskaya or more like Vindija. Ultimately, how interesting is it that these late Neanderthals are not similar in kinship and genetic diversity to the Chagyrskaya and Denisova Cave Neanderthals? Can this simple comparison be expanded to speak to questions of function or richer population dynamics?

- Line 240: Similarly, the results on the population structure and geographical relationships are convincing, but not particularly exciting or surprising. Is this all that can be inferred?

- Given how close Vindija 33.19 and GN1 are, learning that Vindija is closer to the introgressing population does not add much new information. This is not surprising given their locations and the suspected location of introgression. Can these results help refine estimates of the dynamics or other aspects of the introgression as speculated about in lines 319-320?

- Throughout the paper, several possible explanations are proposed to support main results, but these are not formally evaluated or explored in ways that would yield new insights. For example, when discussing the asymmetry of human and Neanderthal ancestry on lines 284-290, several fascinating (and potentially testable) hypotheses are proposed, but no evidence is provided for any of them.

- Line 330: The analysis of accumulation of deleterious mutations and its relevance to Neanderthal disappearance is quite interesting, but very superficial. Deeper analysis of why this pattern might not be seen and placing it in the context of models from other species more relevant to Neanderthals than mammoths would be helpful.

- Lines 187-190: The authors raise an interesting hypothesis regarding the spread of a divergent maternal lineage.

- Lines 88-96: Links between behavior (burial practices vs. cannibalism) and genetic diversity are presented to interpret the genetic results, but these links seem largely speculative.

- Finally, the title of the manuscript reflects the spirit of my points above. What does the “Genetic diversity of the late Neandertals of North-Western Europe” tell us? Can the title be more informative, especially given that the observed variation falls within previously described ranges for non-Siberian Neaderthals. What should the take-home message of this paper be?

In addition to these suggestions for increasing impact, I highlight several minor questions and opportunities to improve the clarity of the presentation of results:

- Line 103: While the section on individual characterization and kinship is necessary, it is quite long and descriptive. The details of age and sex are not fundamental in downstream analyses. Perhaps this section can be streamlined (moved to supplement) and salient details added to a table?

- Line 157: It may be worthwhile to provide an initial summary about what is known about Neanderthals using uniparental markers and then placing the results within that context. Further, it may be helpful for readers to synthesize the nuclear and uniparental marker results somewhere in the text (perhaps the conclusion).

- Line 279: More analysis and description of the human to Neanderthal admixture is needed. Which individuals have it? Which ones don't? Why? What about ILS?

- Table 1: It was unclear how the results in Table 1 compare to those reported in text and supplementary data table 2. I suggest a main text table that provides additional information (e.g., sex, age of individual, age of sample, kinship) in addition to the SNPs covered and contamination. Also, I can't reconcile the numbers in Table 1 with the numbers reported on lines 120-122. Giving more detail and citing the Table earlier would help the reader.

- Line 347: This characterization is more complete and higher resolution, but not "comprehensive".

- The figures generally could be much more information rich. For example, some information on the strata in which the bones were found and ranges of occupation of the sites would be very helpful in setting up expectations. Perhaps both stratigraphy and time could be represented in Figure 1?

- Figure 3A is quite confusing. Suggest moving to supplementary and presenting a direct comparison of the optimal estimates separated by short and long HBD tracts.

- Figure 3B: What does the data presented here tell us that is new? Similar age estimates for all these samples have been previously reported.

- Figure 4: The divergent color scheme makes interpreting the matrix challenging. Consider a palette with a single color that varies in intensity with the F3 statistic. Why were samples with high contamination included?

- Figure 5A: The current color scheme and size make this figure panel nearly impossible to read. Consider plotting in larger size, higher resolution, and with multiple tracks instead of colors in the supplement. Perhaps the main text could include a plot of the different local ancestry length distributions?

- Figure 5B: An evolutionary tree would be helpful here as well as indications of which ancient humans contribute to modern populations.

- Extended Data Figure 8. What chromosome are these examples from? It's not clear from the figure text nor the caption.

Referee #4

(Remarks to the Author)
I co-reviewed this manuscript with one of the reviewers who provided the listed reports.

Referee #5

(Remarks to the Author)
The authors present new and important fine-scale data on the genetics of Late Neanderthals ~40 – 50kyr BP. In particular, this thought-provoking paper addresses regional and inter-regional diversity with some significant conclusions based on the analysis of material from Goyet and other sites in the Meuse basin. I restrict my comments to the archaeological implications of their research.
1. Cannibalism at Goyet: lines 93-5. I suggest they reference Cole 1 who concludes that Goyet is an example of nutritional cannibalism (op.cit Table 3). However, he also points to the low nutritional return of hominins compared to the other prey species that were available (op.cit Table 6). The archaeogenetics reported here establishes that the Goyet sample comprises at least six adult females, while males are present only as a neonate and child/adolescent (Line 152; Extended Date Fig.1 and Table 5.6). The neonate's mother is not in the sample and none of the individuals are related (lines 151-2). These findings add significant detail to Wiβing's (et.al. Ref 65) demonstration that isotopically the Goyet Neanderthals came from a geological catchment outside the Meuse. So, at Goyet we have genetically unrelated Neanderthal individuals of various ages, dominated by females, all have been cannibalised, neither boiled nor roasted 2:6 but eaten raw, and all came from the same, but as yet unspecified geological catchment (summary table attached). δ34S data raises the possibility that SW France was their home catchment, but more work is needed. The lack of relatedness among these 'victims' and where they might have come from is a challenge to the orthodox view of Neanderthal populations as both demographically small and local rather than inter-connected and highly mobile which is the model applied to UPMH.
2. Definition of groups. Lines 93-4 states that the Goyet 'victims', 'represent a group that were cannibalised'. What exactly is

meant by ‘group’? The depositional history of the hominin samples is crucial to this assessment and needs discussing. The site and excavation histories in Supplementary Information H1 would benefit from an assessment of the confidence we can have in the archaeological evidence, much of which is very old and uncertain, to distinguish between a group cannibalised as a single event or individuals taken on multiple occasions but from the same genetic ‘group’. If they were indeed ‘a group’ that lived and died together in a single event, then they are a non-analogue group as judged by the ethnographic literature on small, mobile groups/societies. If, however, they were drawn periodically from the same genetic population then the data indicates a social relationship that existed between the raider (whoever they might be) and the raided (Goyet Neanderthals). Cannibalism is one expression of such asymmetrical relationships. It is a form of commensalism, a rite of incorporation by a ‘host’ who in this instance embodies, literally, opposed social categories. These might be strangers, enemies or others. In our western experience, we have a comparable commensal act in holy communion. It is comparable because it also incorporates social categories into a larger whole by embodying them. The difference is that this is done symbolically rather than literally. Neanderthal cannibalism is not without its own symbolism.
3. Predictive models. Line 94-96. It is unclear to me how this suggestion about genetic diversity follows from the previous sentence. Is the suggestion that there is a genetic group (akin perhaps to the Denisovans, unsuspected until revealed by aDNA) waiting to be discovered in Western Europe? In which case, and following the isotopic possibility of where the ‘victims’ catchment might be, what would the genetic history of SW France be expected to look like? In other words, will the fine-scale findings in this paper encourage archaeogenetics to become a predictive rather than reactive science?
4. General comment on regionality: A great strength of the paper is its focus on inter- and intra-regionality for the Neanderthals. Genetic connectivity between Mezmaiskaya and Les Cottés, is contrasted with the isolation of the late Neanderthals of Western Europe; and especially from UPMH as shown by the lack of introgression. The Western European Neanderthals are more similar among themselves than with those so far sampled to the East. This questions for me their statement in Line 329 that North-Western Europe as a region was a biogeographical crossroad during the Middle and Upper Pleistocene. The genetic evidence presented here for the Late Neanderthals suggests a cul-de-sac rather than a crossroads. Instead of the paper they cite (Ref 66) in support of the crossroad model, the authors should consider the synthesis in the same issue of Quaternary International (411) by Hérisson et al 3. This examines regional change at the timescale of Marine Isotope Stages. What emerges are regular phases of population contraction, most notably MIS 8, and expansion as seen in MIS 7. North-western Europe therefore fits a biotidal-refugium model as proposed by Vrba 4,5 and adapted elsewhere for Western Europe 6: Fig.1. Cyclical change that included glaciation, sea level rise/fall and faunal and floral turnover was the environmental framework of the Late Middle Pleistocene (427-126kyrBP: MIS 11-6) to Middle Upper Pleistocene (126 – 40kyr BP: MIS 5 – 3) within which Neanderthals evolved, adapted and exchanged genes. Hence regional populations were never static but shuffled geographically by cyclical biotidal-refugium forces. For the Late Neanderthals of MIS 3 these same forces have been modelled beginning with Van Andel and Davies 7,8, where a warm and cold phase were shown to have regional impacts on hominin populations. The warm event 45-42kyr BP emerged as particularly significant and is highly relevant to the present paper. I sense however that genetic and environmental regionality are differently conceived with the former mostly independent of the MIS framework provided by quaternary science. Yet, if we are to use all the evidence to explain the disappearance of Neanderthals whose last hurrah is so well documented in this paper, we need an independent framework within which to combine and interpret the hominin data now available from archaeogenetics and archaeology.
5. Summary table: To help the reader through the wealth of detail in the paper I suggest a summary table. An example is attached.
6. Copy editing: The caption to Extended Data Fig.1 needs changing. Last line, ‘Spy I belong to Spy I’ should read ‘Spy 8 belong to Spy I’

1 Cole, J. Assessing the calorific significance of episodes of human cannibalism in the Palaeolithic. Scientific reports 7, 44707 (2017).
2 Rougier, H. et al. Neandertal cannibalism and Neandertal bones used as tools in Northern Europe. Scientific reports 6, 29005 (2016).
3 Hérisson, D. et al. The emergence of the Middle Palaeolithic in north-western Europe and its southern fringes. Quaternary International 411, 233-283 (2016).
4 Vrba, E. S. in Evolutionary History of the "Robust" Australopithecines (ed F.E.Grine) 405-426 (Aldine de Gruyter., 1988).
5 Vrba, E. S., Denton, G. H. & Prentice, M. L. Climatic influences on early hominid behavior. Ossa 14, 127-156. (1989).
6 Gamble, C. S. Human display and dispersal:a case study from biotidal Britain in the Middle and Upper Pleistocene. Evolutionary Anthropology 18, 144-156 (2009).
7 Van Andel, T. The climate and landscape of the middle part of the Weichselian glaciation in Europe: the Stage 3 Project. Quaternary Research 57, 2-8 (2002).
8 Van Andel, T., Davies, W., Weninger, B. & Jöris, O. in Neanderthals and modern humans in the European landscape during the last glaciation (eds T Van Andel & W Davies) 21-29 (McDonald Institute Monographs, 2003).

Version 1:

Reviewer comments:

Referee #1

(Remarks to the Author)
I think the authors have answered all the comments I had. Their focus on inter- and intra-regional genetic diversity, the large number of individuals with genetic data and the generation of one high coverage Neanderthal genome -the forth one- are

strengths of this work. Future integration of all these information in a wider paleoecological and even cultural context can have implications in a number of scientific fields.

Referee #3

(Remarks to the Author)
I thank the authors for their responses. They have largely addressed my previous specific concerns and many of the results are more clearly presented.

While I am sympathetic to the challenge pointed out in the response of identifying unifying take-home messages, I still feel that more is needed to underline the significance of the results.

For example, I think it is very important to discuss the conclusions presented here in light of the recent PNAS paper:
Archaeogenetic insights into the demographic history of Late Neanderthals
https://www.pnas.org/doi/10.1073/pnas.2520565123

In particular, they suggest a major decline in the Neanderthal mtDNA effective population size around 45 years ago.

I was also surprised that that the recent paper from many of the same authors on the genome of another early Neanderthal high-coverage sequence was not cited or discussed here:
A high-coverage Neandertal genome from the Altai Mountains reveals population structure among Neandertals
https://www.pnas.org/doi/abs/10.1073/pnas.2534576123

Finally, I strongly encourage the authors to make their variant calls available along with the raw read data to support reproducibility and future work.

Here are several additional minor suggestions that I believe would improve the clarity of the paper:

- Lines 43-44: Including the estimated age ranges is also important here for hypothesis framing.

- Lines 46-47: Some specificity is worthwhile here. Perhaps "northwestern Europe by X ka" rather than simply "Europe"?

Lines 49-50: To emphasize their genetic load argument, the authors could add a final sentence about genetic load and how genetic factors in their samples do not appear to explain Neanderthal disappearance. This would flow nicely after the current final sentence about well-connected groups.

- Lines 68-70: Do the authors mean to include "close kinship" here? It's remarkable that this kinship was detected in the available Chagyrskaya samples but we should expect some level of relatedness detected in a certain number of samples. Unless the Goyet Neanderthals reflect social organization not observed in any other hominind and/or the cultural practices yield such an odd pattern. Relatedly, on lines 153-156, does this simply reflect imperfect sampling at Goyet? I think the authors should include language here to be specific about whether they are arguing that this reflects sampling, culture, or biology rather than just a "stark contrast".

- Lines 92-93: "varying patterns of mobility" seems vague here. Perhaps the authors should introduce the possibility that some of the Goyet Neanderthal catchment area is potentially hundreds of kilometers away. I believe this is not explained in detail until the Discussion.

- Line 104: The younger date here seems higher than what is indicated in Figure 1C. Could the authors clarify this discrepancy? Does this reflect age vs calibrated age? I also strongly suggest another labeled tick for Figure 1C and relocating "Calibrated age (calBP)" elsewhere in the figure.

- Lines 256-258: Should we be cautious about an excess of ancestral alleles in AR-30 given the low number of informative sites compared to the other samples. Do the locations of the ancestral alleles themselves offer any further insight into this putative divergent lineage? The authors also invoke ascertainment bias in the Supplementary Text but this is omitted from the main text.

- L333-334: The statement that “[genetic load] did not immediately contribute to their disappearance” is unclear and too strong given what is shown. Perhaps rephrase to reflect that the evidence here does not support this hypothesis, but it is not clear they are sufficient to reject it. It would also be helpful to clarify what “immediately contribute” means and how this would be detectable in available data. Given that this is one of the main conclusions highlighted by the authors in the response, it would be valuable to give it a bit more context and description with a particular focus on how these analyses explicitly test this hypothesis. More evidence is needed than simply not seeing a significant difference in a few conservation metrics. Moreover, while low diversity is often cited as a potential contributor to Neanderthal extinction most experts I interact with don’t think of this as the major or main cause.

- Lines 373-376: I'm weary of using "predominantly" here. These results do not permit qualitative measurement of where introgression took place at a regional level. While they may rule out northwestern Europe, this was suspected for the past ~9 years.

- Figure 1A: I suggest using a different symbol for the high coverage nuclear genome, since the blue x is already used to represent a low coverage scenario. I was initially confused when I saw so many blue x's. Size shouldn't be the only discriminator.

Referee #4

(Remarks to the Author)
I co-reviewed this manuscript with one of the reviewers who provided the listed reports.

Referee #5

(Remarks to the Author)
Thank you for answering the points raised in my review. Your paper will generate much discussion and interest.

Open Access This Peer Review File is licensed under a Creative Commons Attribution 4.0 International License, which permits use, sharing, adaptation, distribution and reproduction in any medium or format, as long as you give appropriate credit to the original author(s) and the source, provide a link to the Creative Commons license, and indicate if changes were made.
In cases where reviewers are anonymous, credit should be given to 'Anonymous Referee' and the source.
The images or other third party material in this Peer Review File are included in the article's Creative Commons license, unless indicated otherwise in a credit line to the material. If material is not included in the article's Creative Commons license and your intended use is not permitted by statutory regulation or exceeds the permitted use, you will need to obtain permission directly from the copyright holder.
To view a copy of this license, visit https://creativecommons.org/licenses/by/4.0/

We would like to thank the reviewers for their thoughtful comments and efforts towards improving our manuscript. We have addressed comments specific to each reviewer below, in blue.

Please note that for the Article File resubmission, we provided both a clean manuscript file and a second file with tracked changes to facilitate the review process. As the line numbers between the two files differ, we refer to the line numbers in the clean, untracked file throughout.

Referee expertise:

Referee #1: palaeogenomics
Referee #2: radiocarbon dating
Referee #3: human evolutionary genomics
Referee #5: anthropology

Referees' comments:
Referee #1 (Remarks to the Author):

This paper is interesting for a number of reasons. First, from Eastern Neanderthals, there were genetic observations of endogamy and even consanguinity (the later only in Altai Neandertal), as well as findings of close kin relations within family groups (like in Chagyrskaya). However, it was difficult to know if these traits could be generalized, beyond the marginal, eastern range of Neanderthals. Also, the recent genome of Thorin Neanderthal in France, suggested a potential geographic structure among Late Neanderthals that could preserve some of the prior diversity, largely erased by a migration of eastern Neanderthals (around 105 ka, if we take the reference from the sediment from Galería de las Estatuas). The current work not only adds data from 27 new Neanderthals (one of them at high coverage, accounting now for four such individuals), but also corroborates and clarifies the potential geographical pattern and provides data from North-Western Europe, thus balancing the existing genetic data that until now was clearly shifted to the east (likely because of DNA preservation reasons). Interestingly, these new data do not show evidence neither for endogamy nor consanguinity; on the contrary, these Late Neanderthals lived in diverse and larger, connected group networks, even when modern humans were already impacting in the Middle East and even Eastern Europe. I think it is a work that has some interesting consequences that will need to be further explored from climatic, morphological and maybe even cultural data, and thus it can be of interest for a wide range of scientists.

We thank the reviewer for the insightful assessment of how these new data refine views on Western Late Neandertals.

Minor Points

I find the paragraph between lines 132-143 quite difficult to follow. I think I got the idea that a fragment from Tibia III proved to belong in fact to the individual represented by fragments labelled GN2 (Goyet Q57-1, Q57-2 and Q57-3). Previously Goyet Q305-7 and Q374a-1 were determined to be genetically identical. In Line 139 it is said "As Goyet Q54-4 and Goyet Q374a-1 were genetically distinct…"; I suppose the sentence should work also like this: "As Goyet Q54-4 was genetically distinct to both Goyet Q374a-1 and Goyet Q305-7…". If this is correct, I think the authors should explicitly say that Tibia III reconstruction has joined fragments from two? different individuals (it is unclear for the reader how many fragments have been used to reconstruct Tibia III -could it be more than 2?-). Anyway, this section could be clarified a bit for the general reader.

Thank you for bringing this to our attention. We understand that this paragraph could have been confusing and have rephrased this part of the manuscript and have also added the information into Table 1 (line 106).

The message that we wanted to highlight was that Goyet Q305-7, one of the fragments that had been initially refitted as part of right Tibia III from the individual GN2, was in fact genetically identical to the other individual, GN1. Thus, the Goyet Q305-7 fragment must have been part of the right Tibia V or the left tibia of GN1.

The rephrased text now reads as follows:

"Next, we studied genetic relatedness among 15 skeletal remains with less than 5% contamination using KIN[30](Supplementary Section 6). Among the nine specimens from Goyet, we identified two clusters of genetically identical remains: **the Goyet Neandertal 1 (GN1), composed of Goyet Q56-1, Q374a-1, and Q305-7; and the Goyet Neandertal 2 (GN2), composed of Goyet Q57-1, Q57-2 and Q57-3 (Fig. 1B). Our analysis showed that the Goyet Q305-7 and Q374a-1 fragments, which were previously refitted to two different right tibias (Tibia III and V, respectively), were genetically identical. After re-evaluating their refitting, we concluded that Goyet Q305-7 had been wrongly refitted. We took an additional sample of Tibia III from the fragment Goyet Q54-4, and found that it was genetically distinct from both Goyet Q374a-1 and Q305-7, and therefore did not belong to GN1.** Instead, Goyet Q54-4 was genetically identical and morphologically consistent with the set of remains from GN2 (see Supplementary section 6), demonstrating the potential of genetic analyses to inform the refitting of highly fragmented elements."

Line 153-154; this sentence is difficult to understand. If all sequenced individuals are unrelated each other, it is clear that the mother of neonate Q305-1 have not been sampled.

To clarify this point, we have edited the phrasing to:

"At Goyet, all the sequenced individuals were unrelated adult or adolescent females and juvenile males. **Notably, this included a neonate (Q305-1) whose mother is not represented among the analysed females.**"

One of the most interesting findings is the genetic structure among Late Neandertals that was suggested in the Thorin's paper. Is this something that can be observed at a morphological level? While I assume the remains are too fragmentary to give a conclusive answer, I don't know, maybe there are morphological traits, for instance in dental morphology, that could correlate with these results. That is, are there any traits that could be common to Couvin, Thorin, Stajnia and Mezmaiskaya 1 and 3? Because if there is any hint of differentiation, we could screen the rest of the Neandertal fossil record, including remains with no DNA preserved, that could help us to understand the geographic distribution of this potential population structure. The dental morphology of Thorin (Fuch et al 2024, JHE), does not seem to show obvious differences with Late Neanderthal teeth (with maybe the exception of large premolar roots, according to the authors), but I am unfamiliar with the rest of remains from this potential mtDNA clade.

The reviewer raises an interesting point about how to screen for specimens belonging to the Thorin/Mezmaiskaya/Stajnia mitochondrial clade in the future studies. Unfortunately, as the reviewer points out, there seems to be very limited distinct morphology that could guide these efforts, since the Thorin dental morphology falls within Late Neandertal variation. To our knowledge, no single morphological feature is shared by Couvin, Thorin, Stajnia, Mezmaiskaya 1 and 3 while being absent in other Neandertals, and therefore no diagnostic trait can presently be used to delimit this group with confidence.

Nevertheless, a few morphological features described in these specimens have been noted as unusual and may reflect the effects of drift in small or isolated populations. For example, the lower M3 at Stajnia exhibits a mid-trigonid crest, a feature not previously identified in Neandertals. This trait is otherwise common only in LM1 and LM2 and is considered plesiomorphic (Bailey, 2011, doi:10.1002/ajpa.21468).

In addition, the presence of supernumerary molars in Thorin (Zanolli et al., 2022, doi:10.48738/2022.iss2.809), which at the time of publication when no genetic data were available for this individual was interpreted as possible evidence of hybridization, could also result from drift in small populations. Experimental studies in mice have shown that the expression of this characteristic has a strong genetic component (Fleming et al., 2010, doi:10.1038/sj.bdj.2009.1177). While supernumerary molars have not been documented in Stajnia, Couvin, or Mezmaiskaya, their presence could perhaps serve as a preliminary indicator for the type of broad morphological screening suggested by the reviewer.

In summary, although no specific morphological traits can at present be used to identify this potential group of Neandertals with confidence, the idea merits further exploration in future work.

The observation that no gene flow from modern humans to late Neandertals seem to exist has been postulated before. In Lalueza-Fox (2019) "Neanderthal assimilation?" Nat Ecol Evol, we can read: "Remarkably, the opposite signal -gene flow from Late Pleistocene modern humans into the late Neanderthals- has not been detected so far. It might be that modern humans could tolerate hybrids but Neanderthals could not" (the second sentence is speculative, of course, and there are alternative explanations). I think referencing it could provide previous context for this ongoing observation that stands in sharp contrast with what is seen in early modern humans.

We thank the reviewer for highlighting this relevant study. We have now cited Lalueza-Fox (2021) to acknowledge that this observation was previously proposed based on a smaller dataset of six Neandertals from the putative contact period with modern humans, and to highlight that our results now provide additional support using a much larger dataset.

Referee #2 (Remarks to the Author):

Note that reviewer #2 only provided comments to the editors, which I share with you here so that you can make these minor changes in the revised manuscript files:

We thank the reviewer for the comments to the editor addressed below.

Some minor C14-based corrections in the SI:
-"nearest 10" (SI p79, p 81), this should be "nearest 10 years"

We have modified the text accordingly.

-unclear sentence in p.80: "Since these three fragments were all isolated from unidentified fauna from the site enabled to rule out contamination due to consolidating treatments with varnish, but the samples could have been contaminated between the stable isotopic measurements and the radiocarbon measurements."

We are grateful to the reviewer for pointing this out, we have revised this sentence:

“**Since these three fragments were all isolated from the unidentified fauna collection that did not undergo consolidating treatment – unlike the hominin remains – contamination originating from animal varnish is unlikely. However, the samples could have been contaminated in the laboratory between the stable isotopic and the radiocarbon measurements**.”

and in the main text:-p15, 49.783 cal BP and 39.960 cal BP these should be "," instead of "."

In this section and throughout the manuscript, we have tried to express ages consistently as kyr cal BP. We modified this sentence accordingly, to 49.8 – 40 kyr cal BP instead of 49.783 cal BP and 39.960 cal BP.

Referee #3 (Remarks to the Author):

This manuscript analyzes new genomic data from several late Neanderthals from northwest Europe. Previously, this region has been studied using a handful of low-coverage genomes. This paper introduces additional samples and re-sequences some of the previously available samples, including a new high-coverage genome. These genomic data are a valuable resource for the community. These analyses are possible through the development of new laboratory and computational methods tailored to highly fragmented ancient DNA. The methods are appropriate and clearly described. Further, the authors integrate the genomic results with archaeological data where possible.

I was very excited to read this manuscript. However, while the new analyses quantify Neanderthal variation and allele sharing during this period at finer resolution than previously possible, it is often not clear how the results move beyond confirming what was already known or strongly suspected. In a way, it is wonderful that we now know so much about Neanderthal genomic variation that a new high-coverage genome does not immediately yield fundamental new insights. Nonetheless, I think these new data have great utility. Beyond a few minor technical questions and the need to improve the presentation (especially of figures), my main comments focus on encouraging the authors to: 1) clarify how the results fill existing gaps in knowledge and 2) perform further analyses of these new genomes to yield insights of broad interest.

We thank the reviewer for this thoughtful evaluation and summary of our manuscript. Below, we provide detailed responses to each of the points raised and describe how we have addressed them in the revised manuscript.

In that spirit, I highlight several results where the authors could clarify and expand on what we learn (or even could expect to learn) about Neanderthals from the additional genomes:

- The kinship and diversity results largely confirm what we know from Vindija 33.19. We expect the individuals here to either be more like Chagyrskaya or more like Vindija. Ultimately, how interesting is it that these late Neanderthals are not similar in kinship and genetic diversity to the Chagyrskaya and Denisova Cave Neanderthals? Can this simple comparison be expanded to speak to questions of function or richer population dynamics?

Prior to our study, fine scale nuclear relationships among Neandertals that lived close in time and geographical proximity to one another had only been documented at a single archaeological site (Chagyrskaya Cave in the Altai mountains). Our work provides only a second, independent set of individuals from a different region, and shows a contrasting pattern. In contrast to Chagyrskaya, the Goyet assemblage mostly consists of unrelated individuals. An open question for the future studies is

whether the assemblages at other sites, such as Vindija, also include close relatives such as in Chagyrskaya or instead resemble Goyet.

Regarding genetic diversity, our dataset significantly improves representation of western Neandertals, which until now relied primarily on five individuals with a coverage higher than 1-fold, and only one of whom, Vindija 33.19, is a high coverage genome. By adding a much larger and geographically coherent sample of Neandertals, we strengthen inferences that were previously drawn primarily from a single high-coverage genome. We have emphasized this more clearly in the conclusions of the revised text.

Importantly, our findings re-assess the hypothesis that Late Neandertals declined and eventually disappeared due to their reduced genetic diversity and/or increased inbreeding. Thus, they are especially relevant in challenging the narrative both in the scientific community and in the general public. As detailed in later responses below, we now added additional functional analyses. Taken together, our results support the conclusion that isolation and loss of diversity were unlikely to have been primary drivers of Neandertal extinction.

- Line 240: Similarly, the results on the population structure and geographical relationships are convincing, but not particularly exciting or surprising. Is this all that can be inferred?

The finding of possible gene flow into the AR-30 (Arcy-sur-Cure) Neandertal from a deeper Neandertal population that precedes the split of Late Neandertals has never been reported before and adds a new layer of complexity to Late Neandertal population history. This deep Neandertal population currently remains unidentified, which opens new avenues of research.

Secondly, our more extensive set of Neandertals reaffirms previously proposed differences between Early-Eastern and Late-Western Neandertals, allowing us to corroborate patterns that could before only be speculated with a limited number of individuals. Furthermore, as highlighted by the reviewer #1, the new GN1 high-coverage genome now makes it possible to establish more balanced comparisons among Neandertal populations.

Thirdly, in terms of what else can be assessed about population structure with our current data, we attempted to elucidate how these newly-sequenced late Neandertals relate to Thorin, a Late Neandertal from Mandrin with evidence of long term isolation. Unfortunately, we were not able to obtain raw sequence data from this Neandertal to reprocess them in the same way as ours and other comparative data, and had to rely on the published, already-mapped and filtered sequences. As we described in Supplementary Section 7.2, Thorin was consistently an outlier in all our basic quality checks (Supplementary Figure 7.13) and thus we did not feel confident drawing strong conclusions from these data.

Fourthly, the discrepancies between Thorin’s radiocarbon and molecular dates (based on the bayesian dating of the mitochondrial genome) generated great interest among archaeological, palaeoanthropological, radiocarbon dating and palaeogenetic communities. Therefore, we believe it is an important finding that the Neandertal from Couvin poses a similar case.

- Given how close Vindija 33.19 and GN1 are, learning that Vindija is closer to the introgressing population does not add much new information. This is not surprising given their locations and the suspected location of introgression. Can these results help refine estimates of the dynamics or other aspects of the introgression as speculated about in lines 319-320?

Identifying Vindija 33.19 as genetically closer to the introgressing Neandertal population than GN1 provides a meaningful new insight. Firstly, because considering that GN1 lived closer in time to the latest introgression event, it would have been also plausible to speculate that it would be closer to the

introgressing population. Our results reject that possibility. Moreover, up until now, there were no significant differences among sequenced late Neandertal genomes in their proximity to the main Neandertal population that admixed with modern humans. Therefore, we demonstrate for the first time that even within Late Neandertal groups, there is detectable long-term population structure, which was not clear from previously available data.

Our results further help to refine the potential geographic context of the main introgression event by indicating where it is unlikely to have occurred, specifically in northern Europe. While earlier studies suggested that admixture probably took place in the Middle East, our data provide concrete evidence and thus contribute towards constraining the geographic parameters of the introgression, particularly by distinguishing among closely related Neandertal populations close to the time of the introgression and at fine resolution.

- Throughout the paper, several possible explanations are proposed to support main results, but these are not formally evaluated or explored in ways that would yield new insights. For example, when discussing the asymmetry of human and Neanderthal ancestry on lines 284-290, several fascinating (and potentially testable) hypotheses are proposed, but no evidence is provided for any of them.

In the manuscript, we outline four plausible scenarios that could explain the observed asymmetry of human and Neandertal gene-flow. We expand on these below:

(1) Timing. If admixture occurred shortly after a subset of modern humans left Africa, and those populations subsequently expanded and colonized the globe, all present-day non-Africans would be expected to share this common signature of gene flow. By contrast, Neandertals may have remained more geographically constrained and not all late Neandertals might have had a chance of encountering modern humans. Unless the specific Neandertal groups involved in the admixture are directly sampled, we would not expect to observe the signal of gene-flow from modern humans into late Neandertals, as it did not have enough time to spread to other Neandertal populations.

(2) Demographic imbalances. If modern humans had substantially larger effective population sizes than Neandertals, introgressed Neandertal ancestry would have been more likely to persist and spread within modern human populations, whereas introgressed modern human alleles entering small, possibly declining Neandertal groups could have been rapidly lost by drift. This demographic asymmetry could therefore contribute to the uneven distribution of respective ancestry.

(3) Sex-biased admixture. The observed asymmetry patterns could also be observed if, for example, interactions occurred mainly between male Neandertals and female modern humans. F1 offspring would have been integrated predominantly into modern human groups, leaving a stronger and more persistent ancestry signal in modern humans than in Neandertals.

(4) Differential incorporation of F1 individuals. It is also possible that hybrid children were socially incorporated into modern human communities but not into Neandertal ones. Under this scenario, only modern humans would retain detectable traces of admixture.

Unfortunately, these hypotheses are difficult to evaluate directly. We lack key empirical data, such as ancient DNA from the specific introgressing modern human populations, and realistic simulations would require complex demographic scenarios and highly subjective parameter choices. Moreover, the asymmetry we observe may reflect a combination of several—or even all—of the factors listed above, as well as others not yet considered (e.g. selection). Therefore, testing these hypotheses lies beyond the scope of our study. However, we hope that the dataset we present will provide a foundation for future work addressing this challenging question.

- Line 330: The analysis of accumulation of deleterious mutations and its relevance to Neanderthal disappearance is quite interesting, but very superficial. Deeper analysis of why this pattern might not be seen and placing it in the context of models from other species more relevant to Neanderthals than mammoths would be helpful.

We acknowledge that our analysis of accumulation of deleterious mutations was not detailed enough, and was mostly focusing on the ratio between synonymous and non-synonymous polymorphisms. We added two more analyses using polyphen and phyloP, based on the article by van der Walk et al. (2019, doi:10.1016/j.cub.2018.11.055), on Grauer's and mountain Gorillas. Even though the time scales vastly differ, this is one of the few species with genome-scale time-series data available. Both analyses show no qualitative or significant differences between early and late Neandertals, thus increasing the support for our claim that reduction of genetic diversity and accumulation of deleterious variants was not a major factor in the disappearance of Neandertals.

- Lines 187-190: The authors raise an interesting hypothesis regarding the spread of a divergent maternal lineage.

We thank the reviewer, and agree that we look forward to future studies in which more individuals belonging to this specific mtDNA clade can further elucidate their population history, especially through the additional analyses of nuclear data.

- Lines 88-96: Links between behavior (burial practices vs. cannibalism) and genetic diversity are presented to interpret the genetic results, but these links seem largely speculative.

We thank the reviewer for this comment. Particularly also in response to the issues raised by reviewer 5, we comprehensively reworked this section, and it is now more clear the speculative nature of this link, referring to diversity in general: "**[...] key aspects of Neandertal population structure in this region still remain unexplored. Although over 80 Neandertal remains have been identified at Spy and 101 at Goyet[23], it is uncertain how many distinct individuals these represent, with current morphology-based analyses estimating at least three individuals at Spy[19] and six at Goyet[23]. Despite their genetic similarity, mortuary practices of the Neandertals from Spy and Goyet are very different. At Spy, the anatomical connection between the bones suggests deliberate burial[24], although this interpretation has been questioned[25]. In contrast, the Neandertal remains at Goyet are highly fragmented, with anthropogenic modifications suggesting they represent a group that was cannibalised[20]. Together with isotopic data indicating varying patterns of mobility[26], this could suggest greater diversity among the late Neandertals in North-Western Europe than is represented in the current genetic data.**"

- Finally, the title of the manuscript reflects the spirit of my points above. What does the "Genetic diversity of the late Neandertals of North-Western Europe" tell us? Can the title be more informative, especially given that the observed variation falls within previously described ranges for non-Siberian Neaderthals. What should the take-home message of this paper be?

Given the variety of results presented in this paper, we think a broad non-speculative title is more appropriate to reach a wider audience. In this way, while archaeologists might be more interested in the absence of close-kin individuals in the assemblage in contrast to Chagyrskaya, and/or the applications of genetic kinship to morphological refitting to reconstruct skeletal remains; palaeogeneticists will likely focus on the results about population history, functional analysis and/or the lack of recent modern human ancestry in sequenced Neandertal genomes. This makes it challenging to find just one unifying take-home point for all disciplines. As reviewer #1 points out: "it is a work that has some interesting consequences that will need to be further explored from climatic, morphological and maybe even cultural data, and thus it can be of interest for a wide range of scientists".

In addition to these suggestions for increasing impact, I highlight several minor questions and opportunities to improve the clarity of the presentation of results:

- Line 103: While the section on individual characterization and kinship is necessary, it is quite long and descriptive. The details of age and sex are not fundamental in downstream analyses. Perhaps this section can be streamlined (moved to supplement) and salient details added to a table?

Following your suggestion, we have added the age and sex information to Table 1, as well as to the new supplementary Table 9 (suggested by reviewer #5).

However, while we agree that from a paleogenetic point of view these findings are not fundamental for the interpretation of the population genetic analyses and could therefore be omitted from the main text; we believe that they are especially relevant for other disciplines such as biological anthropology and archaeology. As reviewer #5 points out in their comment #1:

"These findings add significant detail to Wiβing's (et.al. Ref 65) demonstration that isotopically the Goyet Neanderthals came from a geological catchment outside the Meuse. So, at Goyet we have genetically unrelated Neanderthal individuals of various ages, dominated by females, all have been cannibalised, neither boiled nor roasted 2:6 but eaten raw, and all came from the same, but as yet unspecified geological catchment (summary table attached). δ34S data raises the possibility that SW France was their home catchment, but more work is needed. The lack of relatedness among these 'victims' and where they might have come from is a challenge to the orthodox view of Neanderthal populations as both demographically small and local rather than inter-connected and highly mobile which is the model applied to UPMH."

Thus, they provide context for research focusing on Neandertal behaviour (cannibalism) and locality. Moreover, the section about kinship and how the genetic results enabled a revision of the refitting of the different bones might be of interest to archaeologists dealing with similarly highly-fragmented skeletal assemblages.

- Line 157: It may be worthwhile to provide an initial summary about what is known about Neanderthals using uniparental markers and then placing the results within that context. Further, it may be helpful for readers to synthesize the nuclear and uniparental marker results somewhere in the text (perhaps the conclusion).

We have now re-worked that section (lines 158-191) to provide more context and background information on Neandertal uniparental markers, including references to recent papers that provide an overview on Neandertal MT variation (32, Andreeva et al., 2022; doi:10.1038/s41598-022-16164-9) and Y chromosome variation (37, Swiel et al.,2025; doi: 0.1186/s13059-024-03468-4).

We also followed the reviewers' suggestion and offer an interpretation summarizing our results in the conclusion (lines 365-371): "**Our results for both the nuclear data of Arcy-sur-Cure and the mtDNA genome of Couvin hint at a more complex population structure of late Neandertals, but higher-quality data will be needed to corroborate this. Taken together, the genetic data indicate substantial connectivity among late Neandertal groups within North-Western Europe, consistent with isolation-by-distance patterns. Rather than reflecting a permanent biogeographical crossroads, this connectivity likely resulted from episodic phases of regional openness within a broader framework of climatically driven population contraction and expansion during the Middle and Upper Pleistocene**[74]."

- Line 279: More analysis and description of the human to Neanderthal admixture is needed. Which individuals have it? Which ones don't? Why? What about ILS?

We have edited the main text (lines 279-287) to make it clearer that the focus of this section is detecting evidence of the most recent gene flow between Neandertals and modern humans. Our main goal was to highlight that we find no evidence for a recent gene flow from modern humans to late Neandertals.

Unfortunately, for older events, fragments detected by admixfrog are too small and it becomes very challenging to untangle them from ILS, which is why we refrain from a more detailed analysis here. The section now reads: "**As these Neandertals lived at most 500 generations after the first possible interactions with early modern humans, we would expect multiple introgressed modern human DNA tracts longer than 1 cM in their genomes if introgression took place. We identified potential tracts of modern human DNA in four of the ten Neandertals, but the longest tract we found was only 0.41 cM long, inconsistent with recent introgression (Fig. 5A, Supplementary Table 9.1). Since all of the putative modern human tracts we identified were short, and lacked fixed differences between Neandertals and modern humans (Extended Data Fig. 8, Supplementary section 9) they are consistent with previous evidence of much older gene flow from modern humans to Neandertals or with incomplete lineage sorting[65]**."

- Table 1: It was unclear how the results in Table 1 compare to those reported in text and supplementary data table 2. I suggest a main text table that provides additional information (e.g., sex, age of individual, age of sample, kinship) in addition to the SNPs covered and contamination. Also, I can't reconcile the numbers in Table 1 with the numbers reported on lines 120-122. Giving more detail and citing the Table earlier would help the reader.

We agree that additional details should be included in Table 1, as also noted by other reviewers. Accordingly, beyond coverage and contamination estimates, we have added columns indicating the specimens associated with each individual, their ages, and their genetic sex. We did not include a kinship column because all individuals are unrelated at least to the third degree. Information on sample age is now also reflected in Figure 1C.

Regarding the apparent discrepancy in SNP values reported in lines 120–122, we have clarified in the text that this difference arises from the level at which quality control was performed (line 117). Specifically, the statistics in lines 120–122 were calculated at the library level, to gain an overall view of the quality of the data generated before doing any downstream analyses, whereas the values presented in Table 1 correspond to data after merging libraries by specimen, as well as then those belonging to the same individual (i.e. after performing kinship analyses). In other words, the first section reports library-level statistics that are used to inform data curation, while subsequent sections and Table 1 refer to individual-level data. In relation to Supplementary Table 5, the values in lines 120–122 correspond to Supplementary Table 5.1 (library-level data), whereas those in Table 1 correspond to Supplementary Table 5.6 (curated data merged by individual).

Finally, we have also modified the main text so that the table is cited earlier (line 106).

- Line 347: This characterization is more complete and higher resolution, but not "comprehensive".

We have adjusted the sentence: "Together these genomes provide an overview of late Neandertal genetic diversity, and demonstrate that even low-coverage nuclear genome data, when integrated with their palaeoanthropological context, can **substantially** increase the resolution of within-Neandertal diversity."

- The figures generally could be much more information rich. For example, some information on the strata in which the bones were found and ranges of occupation of the sites would be very helpful in setting up expectations. Perhaps both stratigraphy and time could be represented in Figure 1?

We agree about the need to include the ages of the specimens to make it easier for the reader to contextualise the result, a point that has also been raised by reviewer #5. We have added a new Figure 1C with the radiocarbon dates by site. Moreover, following reviewer #5's suggestion, we have added this information to Supplementary Table 9, as well as Table 1, which is now cited earlier in the text (line 106).

Unfortunately, some of the sites were excavated during the 19th and the beginning of the 20th century, when the habitual procedures for site documentation fell short of today's standards. As a result, for some archaeological sites such as Goyet or Spy, it would be impossible to assign all samples to their layers in the figure following the reviewer's suggestion. More detailed information about the history and stratigraphy of each site can be found in Supplementary Section H1.

- Figure 3A is quite confusing. Suggest moving to supplementary and presenting a direct comparison of the optimal estimates separated by short and long HBD tracts.

We have updated Figure 3A according to the reviewer's comment.

- Figure 3B: What does the data presented here tell us that is new? Similar age estimates for all these samples have been previously reported.

While our study does not define any new branches in the Neandertal Y chromosome phylogeny or their relative ages, the novel aspect we wish to highlight in Figure 3B is the lineage assignment of Fonds-de-Forêt 1, Goyet Q305-1, and Trou Magrite 2422-36.

- Figure 4: The divergent color scheme makes interpreting the matrix challenging. Consider a palette with a single color that varies in intensity with the F3 statistic. Why were samples with high contamination included?

We have adjusted the colour palette of Figure 3D accordingly.

As for Figures 3A and 3C, we included the samples with lower quality data because these are the only data available for these specimens, and some of the analyses lead to interpretable insights. Thus, even with some contamination, we believe the findings to be qualitatively interpretable.

In the first case (Panel 4A), the D-statistics compare whether the Neandertals show more affinity to Vindija 33.19 versus Chagyrskaya 8, and to Vindija 33.19 versus GN1. Present-day human contamination could, at most, artificially make samples closer to the Neandertal that carries ancestry closer to the introgressing population, but considering the small amount of Neandertal ancestry in present-day non-Africans (~2%) we do not believe that this would have a significant impact.

In the second case, the split times are obtained through an f(A|B) analyses, in which the frequency of derived sites in each Neandertal genome is calculated on sites that are heterozygous for Vindija 33.19. Present-day modern human contamination would introduce an excess of ancestral variants at those sites, and therefore, would shift the split times to deeper in time. This is in line with the results depicted in Figure 3C.

- Figure 5A: The current color scheme and size make this figure panel nearly impossible to read. Consider plotting in larger size, higher resolution, and with multiple tracks instead of colors in the supplement. Perhaps the main text could include a plot of the different local ancestry length distributions?

Thank you for your feedback. We have implemented it. We also want to point out that the purpose of

the figure is to show that there are no reliable fragments for the most recent admixture event between Neandertals and modern humans. The very short fragments observed here, as mentioned in previous comments, are indicative of either an older event common to all Neandertals, or ILS, and therefore not informative for this purpose. Overall, the ancestry of all of the genomes analysed here appear uniformly Neandertal.

- Figure 5B: An evolutionary tree would be helpful here as well as indications of which ancient humans contribute to modern populations.

We have added a sentence to make it clear that all of the ancient modern human individuals that are singled-out in Figure 5B (including BK BB7-240, BK CC7-335, BK F6-620, Oase 1, Ranis 13, Ust'-Ishim and Zlatý kůň) have not contributed to present-day populations, and therefore a tree would not be useful in this regard.

- Extended Data Figure 8. What chromosome are these examples from? It's not clear from the figure text nor the caption.

We have updated Extended Data Figure 8 so that now the chromosome and positions are indicated in each panel.

Referee #4 (Remarks to the Author):

I co-reviewed this manuscript with one of the reviewers who provided the listed reports.

We thank the reviewer for contributing to the other review, and hope to have satisfactorily addressed the points raised there.

Referee #5 (Remarks to the Author):

The authors present new and important fine-scale data on the genetics of Late Neanderthals ~40 – 50kyr BP. In particular, this thought-provoking paper addresses regional and inter-regional diversity with some significant conclusions based on the analysis of material from Goyet and other sites in the Meuse basin. I restrict my comments to the archaeological implications of their research.

We thank the reviewer for providing detailed archaeological insights that will strengthen our paper, and address each of the points individually below.

1. Cannibalism at Goyet: lines 93-5. I suggest they reference Cole 1 who concludes that Goyet is an example of nutritional cannibalism (op.cit Table 3).

Thank you for pointing us towards this relevant article, we have added the reference in the main text.

However, he also points to the low nutritional return of hominins compared to the other prey species that were available (op.cit Table 6). The archaeogenetics reported here establishes that the Goyet sample comprises at least six adult females, while males are present only as a neonate and child/adolescent (Line 152; Extended Date Fig.1 and Table 5.6). The neonate's mother is not in the sample and none of the individuals are related (lines 151-2). These findings add significant detail to Wiβing's (et.al. Ref 65) demonstration that isotopically the Goyet Neanderthals came from a geological catchment outside the Meuse. So, at Goyet we have genetically unrelated Neanderthal individuals of

various ages, dominated by females, all have been cannibalised, neither boiled nor roasted 2:6 but eaten raw, and all came from the same, but as yet unspecified geological catchment (summary table attached). δ34S data raises the possibility that SW France was their home catchment, but more work is needed. The lack of relatedness among these 'victims' and where they might have come from is a challenge to the orthodox view of Neanderthal populations as both demographically small and local rather than inter-connected and highly mobile which is the model applied to UPMH.

We thank the reviewer for the comprehensive synthesis of the archaeological investigations undertaken at Goyet to date. We have incorporated the suggested references into the main text. In addition, since the submission of this manuscript for revision, we have published a complementary study on the morphology of the Goyet Neandertals (Cosnefroy et al., 2025; doi:10.1038/s41598-025-24460-3). As that work engages more directly with the archaeological aspects highlighted in the reviewer's comments, we consider that a detailed discussion of these points is better situated within the context of the Cosnefroy et al. paper, whereas the present manuscript focuses more on the new genetic findings.

2. Definition of groups. Lines 93-4 states that the Goyet 'victims', 'represent a group that were cannibalised'. What exactly is meant by 'group'? The depositional history of the hominin samples is crucial to this assessment and needs discussing. The site and excavation histories in Supplementary Information H1 would benefit from an assessment of the confidence we can have in the archaeological evidence, much of which is very old and uncertain, to distinguish between a group cannibalised as a single event or individuals taken on multiple occasions but from the same genetic 'group'. If they were indeed 'a group' that lived and died together in a single event, then they are a non-analogue group as judged by the ethnographic literature on small, mobile groups/societies. If, however, they were drawn periodically from the same genetic population then the data indicates a social relationship that existed between the raider (whoever they might be) and the raided (Goyet Neanderthals). Cannibalism is one expression of such asymmetrical relationships. It is a form of commensalism, a rite of incorporation by a 'host' who in this instance embodies, literally, opposed social categories. These might be strangers, enemies or others. In our western experience, we have a comparable commensal act in holy communion. It is comparable because it also incorporates social categories into a larger whole by embodying them. The difference is that this is done symbolically rather than literally. Neanderthal cannibalism is not without its own symbolism.

We acknowledge that the term "group" is a bit ambiguous when applied to Neandertal assemblages, as it could potentially refer to (#1) a single contemporaneous set of individuals, or (#2) a cohesive assemblage that spans more time and has some common elements (e.g. same geographical origin, population continuity, etc.).

Given the broad intervals of confidence both in the genetic and radiocarbon dates of the specimens, possibility #1 could only be firmly established by the identification of close kin through genetic analysis, which unfortunately is not the case given our results. Yet, these Neandertals could have been contemporaneous but not related. Therefore, we cannot accept or refute option #1.

Regarding option #2, we believe it plausible based on the similarity of how the bones were treated. Moreover, it also offers a more parsimonious explanation for the presence of immature individuals unrelated to the majority of the females. For a more extensive discussion on the repercussions this could have into the implications for group structure and behaviour we would refer to Consnefroy et al., 2025.

3. Predictive models. Line 94-96. It is unclear to me how this suggestion about genetic diversity follows from the previous sentence. Is the suggestion that there is a genetic group (akin perhaps to the Denisovans, unsuspected until revealed by aDNA) waiting to be discovered in Western Europe? In which case, and following the isotopic possibility of where the 'victims' catchment might be, what would

the genetic history of SW France be expected to look like? In other words, will the fine-scale findings in this paper encourage archaeogenetics to become a predictive rather than reactive science?

We apologise for any possible misunderstanding that could have arisen in this part. We did not intend to speculate in this section about any unsampled population of Neandertals in the region. Instead, our intention was to suggest that the diversity in behaviour (cannibalism vs burial) and locality (victims from a different area vs “local” Meuse-Valley Neandertals), combined with the advantages of a finer-scale approach, could potentially reveal at a greater genetic diversity than what was possible until now, due to the limited number of samples.

In this sense, it would have been plausible to find other Neandertal groups with a long history of isolation, such as Thorin. It is highly unlikely – albeit not impossible – to discover a totally novel group amidst the Neandertals of the Meuse Basin. And while we agree that it would be a very exciting development to move towards an increase in the predictive power of archaeology, this is unfortunately beyond the scope of our study.

4. General comment on regionality: A great strength of the paper is its focus on inter- and intra-regionality for the Neanderthals. Genetic connectivity between Mezmaiskaya and Les Cottés, is contrasted with the isolation of the late Neanderthals of Western Europe; and especially from UPMH as shown by the lack of introgression. The Western European Neanderthals are more similar among themselves than with those so far sampled to the East. This questions for me their statement in Line 329 that North-Western Europe as a region was a biogeographical crossroad during the Middle and Upper Pleistocene. The genetic evidence presented here for the Late Neanderthals suggests a cul-de-sac rather than a crossroads. Instead of the paper they cite (Ref 66) in support of the crossroad model, the authors should consider the synthesis in the same issue of Quaternary International (411) by Hérisson et al 3. This examines regional change at the timescale of Marine Isotope Stages. What emerges are regular phases of population contraction, most notably MIS 8, and expansion as seen in MIS 7. North-western Europe therefore fits a biotidal-refugium model as proposed by Vrba 4,5 and adapted elsewhere for Western Europe 6: Fig.1. Cyclical change that included glaciation, sea level rise/fall and faunal and floral turnover was the environmental framework of the Late Middle Pleistocene (427-126kyrBP: MIS 11-6) to Middle Upper Pleistocene (126 – 40kyr BP: MIS 5 – 3) within which Neanderthals evolved, adapted and exchanged genes. Hence regional populations were never static but shuffled geographically by cyclical biotidal-refugium forces.

For the Late Neanderthals of MIS 3 these same forces have been modelled beginning with Van Andel and Davies 7,8, where a warm and cold phase were shown to have regional impacts on hominin populations. The warm event 45-42kyr BP emerged as particularly significant and is highly relevant to the present paper. I sense however that genetic and environmental regionality are differently conceived with the former mostly independent of the MIS framework provided by quaternary science. Yet, if we are to use all the evidence to explain the disappearance of Neanderthals whose last hurrah is so well documented in this paper, we need an independent framework within which to combine and interpret the hominin data now available from archaeogenetics and archaeology.

We thank the reviewer for this insightful comment and agree that north-western Europe should not be viewed as a permanent biogeographical crossroads during the Middle and Upper Pleistocene. The genetic homogeneity observed among late western Neanderthals indeed points to phases of regional isolation, particularly during MIS 3. Following the synthesis of Hérisson et al. (2016), we now interpret north-western Europe within a biotidal-refugium framework, characterised by recurrent phases of population contraction (e.g. MIS 8) and expansion (e.g. MIS 7) at the scale of Marine Isotope Stages. Within this model, the region functioned as a temporally variable interface rather than a constant crossroads, becoming connected to wider Neanderthal networks only during climatically favourable windows. This dynamic perspective is consistent with models proposed by Van Andel and Davies,

notably highlighting the warm interval around 45–42 ka BP as a key phase of increased mobility and connectivity. It provides an independent environmental framework within which the genetic and archaeological data presented here can be jointly interpreted, reconciling episodic east–west connections with the late isolation of western Neanderthals. We have updated lines 369-371 accordingly to reflect this.

5. Summary table: To help the reader through the wealth of detail in the paper I suggest a summary table. An example is attached.

Thank you for providing this complete table. We agree that readers can greatly benefit from it, especially if they are unfamiliar with the previous research that has been conducted in the Neandertals from this area, so we have added it as Supplementary Data Table 9.

6. Copy editing: The caption to Extended Data Fig.1 needs changing. Last line, 'Spy I belong to Spy I' should read 'Spy 8 belong to Spy I'

Thank you, we have corrected this typo in the Extended Data Fig.1 caption.

1 Cole, J. Assessing the calorific significance of episodes of human cannibalism in the Palaeolithic. Scientific reports 7, 44707 (2017).
2 Rougier, H. et al. Neandertal cannibalism and Neandertal bones used as tools in Northern Europe. Scientific reports 6, 29005 (2016).
3 Hérisson, D. et al. The emergence of the Middle Palaeolithic in north-western Europe and its southern fringes. Quaternary International 411, 233-283 (2016).
4 Vrba, E. S. in Evolutionary History of the "Robust" Australopithecines (ed F.E.Grine) 405-426 (Aldine de Gruyter., 1988).
5 Vrba, E. S., Denton, G. H. & Prentice, M. L. Climatic influences on early hominid behavior. Ossa 14, 127-156. (1989).
6 Gamble, C. S. Human display and dispersal:a case study from biotidal Britain in the Middle and Upper Pleistocene. Evolutionary Anthropology 18, 144-156 (2009).
7 Van Andel, T. The climate and landscape of the middle part of the Weichselian glaciation in Europe: the Stage 3 Project. Quaternary Research 57, 2-8 (2002).
8 Van Andel, T., Davies, W., Weninger, B. & Jöris, O. in Neanderthals and modern humans in the European landscape during the last glaciation (eds T Van Andel & W Davies) 21-29 (McDonald Institute Monographs, 2003).

We would like to thank once more all the reviewers for their comments and efforts towards improving our manuscript. We address the remaining comments from Referee #3 below, point by point, in blue:

I thank the authors for their responses. They have largely addressed my previous specific concerns and many of the results are more clearly presented.

While I am sympathetic to the challenge pointed out in the response of identifying unifying take-home messages, I still feel that more is needed to underline the significance of the results.

For example, I think it is very important to discuss the conclusions presented here in light of the recent PNAS paper:
Archaeogenetic insights into the demographic history of Late Neanderthals
https://www.pnas.org/doi/10.1073/pnas.2520565123

In particular, they suggest a major decline in the Neanderthal mtDNA effective population size around 45 years ago.

I was also surprised that that the recent paper from many of the same authors on the genome of another early Neanderthal high-coverage sequence was not cited or discussed here:
A high-coverage Neandertal genome from the Altai Mountains reveals population structure among Neandertals
https://www.pnas.org/doi/abs/10.1073/pnas.2534576123

We now refer to the *Fotiadou et al., 2026* paper in lines 78 and 314; and to the *Massilani et al., 2026* paper in lines 54 and 58. While a major decline has been suggested based on the mtDNA around 45,000 years ago, we find no support for a genetic decline or significant differences in the genetic load between late Neandertals and Neandertals living 130,000 or 60,000 years ago.

Finally, I strongly encourage the authors to make their variant calls available along with the raw read data to support reproducibility and future work.

We agree, and are working on making the called genotypes available.

Here are several additional minor suggestions that I believe would improve the clarity of the paper:

- Lines 43-44: Including the estimated age ranges is also important here for hypothesis framing.

We have added this information to lines 40-41.

- Lines 46-47: Some specificity is worthwhile here. Perhaps "northwestern Europe by X ka" rather than simply "Europe"?

We have added this information to lines 45-46.

Lines 49-50: To emphasize their genetic load argument, the authors could add a final sentence about genetic load and how genetic factors in their samples do not appear to explain Neanderthal disappearance. This would flow nicely after the current final sentence about well-connected groups.

We have added this information to lines 48-50.

- Lines 68-70: Do the authors mean to include "close kinship" here? It's remarkable that this kinship was detected in the available Chagyrskaya samples but we should expect some level of relatedness detected in a certain number of samples. Unless the Goyet Neanderthals reflect social organization not observed in any other hominind and/or the cultural practices yield such an odd pattern. Relatedly, on lines 153-156, does this simply reflect imperfect sampling at Goyet? I think the authors should include language here to be specific about whether they are arguing that this reflects sampling, culture, or biology rather than just a "stark contrast".

In lines 66–70, we refer specifically to previously published findings from Chagyrskaya, where close kinship relationships were identified among individuals. We have used the term "close kinship" in that context to accurately reflect the conclusions from Skov et al., 2022.

Regarding the broader point raised by the reviewer, we disagree that some degree of relatedness can be expected in most archaeological assemblages with sufficient samples. Indeed, we chose our language here to emphasize the very different data pattern compared to the only other comparable Neandertal site with genome-wide data of multiple individuals, Chagyrskaya Cave in Russia. Overall we think that, based on the available data alone, we cannot confidently disentangle the relative importance of sampling, culture and biology, as they are not mutually exclusive and all three of them could have led to an assemblage of unrelated individuals.

We therefore do not interpret the observed pattern at Goyet as necessarily reflecting unusual social organization or cultural practice, but rather as potentially influenced by the specific formation history and sampling of the assemblage. We have revised the manuscript (lines 147–149) to clarify this point and to avoid overinterpretation: "As it is unclear whether this assemblage might represent a single community, we refrain from making inferences about their group structure or culture"

At the same time, we believe it remains relevant to note specific patterns such as the presence of a neonate without a genetically identified mother among the sampled individuals, as this observation may inform future archaeological interpretations of site formation and demographic composition.

- Lines 92-93: "varying patterns of mobility" seems vague here. Perhaps the authors should introduce the possibility that some of the Goyet Neanderthal catchment area is potentially hundreds of kilometers away. I believe this is not explained in detail until the Discussion.

We have updated lines 89-92 accordingly.

- Line 104: The younger date here seems higher than what is indicated in Figure 1C. Could the authors clarify this discrepancy? Does this reflect age vs calibrated age? I also strongly suggest another labeled tick for Figure 1C and relocating "Calibrated age (calBP)" elsewhere in the figure.

We have fixed the inconsistency, and now we report the same calibrated age ranges in lines 101-103 and Figure 1C.

- Lines 256-258: Should we be cautious about an excess of ancestral alleles in AR-30 given the low number of informative sites compared to the other samples. Do the locations of the ancestral alleles themselves offer any further insight into this putative divergent lineage? The authors also invoke ascertainment bias in the Supplementary Text but this is omitted from the main text.

We agree with the reviewer that some caution is warranted when interpreting patterns in AR-30 given its relatively low number of informative sites. However, the excess of ancestral alleles cannot be explained simply by low coverage for two reasons. First, while reduced SNP counts increase stochastic variance and widen confidence intervals, there is no obvious mechanism by which low coverage alone would systematically bias calls toward ancestral rather than derived alleles. Second, several other specimens in our dataset with comparable or even lower coverage (e.g. Goyet Q375-25, Goyet Q55-4, Goyet Q119-2) do not show any similar enrichment of ancestral alleles, suggesting that coverage alone is not sufficient to explain the pattern and is consistent instead with increased noise, as expected under the first point.

Regarding the Supplementary Text, we believe there may have been a misunderstanding of our intent. The sentence in question states: "Besides contamination [which is explored and ruled out as an explanation in the previous paragraphs], another plausible scenario that would explain this excess of ancestral alleles in AR-30 would be gene flow from a deeper Neandertal lineage. However, due to the low amount of data and ascertainment bias of the array we cannot corroborate this hypothesis." Here, our reference to ascertainment bias was intended specifically to point out the difficulty of delving into this question deeper with the capture data and the way the Archaic Plus capture array was designed. We did not intend to suggest that ascertainment bias could generate the observed excess of ancestral alleles in AR-30 specifically. Moreover, there is no reason an ascertainment bias would affect only the AR-30 Neandertal, but not the rest of the specimens. Current capture data might make it harder to determine exactly which deeply diverged Neandertal lineage could have contributed

to AR-30, e.g., if the gene-flow stems from a lineage not currently known/represented in the Archaic Plus design. The gene-flow from a deeper Neandertal lineage remains the most parsimonious hypothesis explaining observed data of AR-30.

- L333-334: The statement that "[genetic load] did not immediately contribute to their disappearance" is unclear and too strong given what is shown. Perhaps rephrase to reflect that the evidence here does not support this hypothesis, but it is not clear they are sufficient to reject it. It would also be helpful to clarify what "immediately contribute" means and how this would be detectable in available data. Given that this is one of the main conclusions highlighted by the authors in the response, it would be valuable to give it a bit more context and description with a particular focus on how these analyses explicitly test this hypothesis. More evidence is needed than simply not seeing a significant difference in a few conservation metrics. Moreover, while low diversity is often cited as a potential contributor to Neanderthal extinction most experts I interact with don't think of this as the major or main cause.

We agree with the overall statement that most experts would not have considered low genetic diversity/increased load as the major/main cause of extinction, but we think this analysis is important because it is one of the first attempts to actually quantify this directly. That said, we acknowledge that our analysis is limited and cannot rule out that load had some effect, so we adjusted the phrasing of lines 324-325 accordingly.

- Lines 373-376: I'm weary of using "predominantly" here. These results do not permit qualitative measurement of where introgression took place at a regional level. While they may rule out northwestern Europe, this was suspected for the past ~9 years.

While the fact that recent gene flow between Neandertals and modern humans probably did not take place in northwestern Europe had been speculated before, this is, to our knowledge, the first time such conjectures can be explicitly tested with nuclear data from a large number of Late Neandertals from northwestern Europe. Therefore, we believe it important to state it.

- Figure 1A: I suggest using a different symbol for the high coverage nuclear genome, since the blue x is already used to represent a low coverage scenario. I was initially confused when I saw so many blue x's. Size shouldn't be the only discriminator.

We have adjusted Figure 1A accordingly, so that now the high-coverage genome is represented by a blue triangle instead.

Late Neanderthals Meuse Basin											
				Isotopes				Genetic cluster	AMH	C14	Genetic data
			Sex	$\delta^{34}S$	Archaeology	Taphonomy	Kin	shared	introgression	calBP	samples
Spy	Spy I	adult	M	catchment within Meuse basin	bodies but burial unlikely	evidence of fatal injury	no data	shared with Goyet	none	54.320 - 42,160	4
	Spy II	adult	no data								
	Spy VI	18 month child	no data		reworked context	no data					
Goyet	Q305-1	neonate	M	catchment outside Meuse basin	cannibalism	eaten uncooked, no evidence for roasting or boiling	unrelated	shared with Spy	none	45,000 - 41,000	15
	1424-3D	6.5-12.5 year adolescent	M								
	C5-1	adult/adolescent	?								
	Q305-4	adult	F								
	Q376-25	adult	F								
	Q55-4	adult	F								
	Q119-2	adult	F								
	GN1	adult	F								
	GN2	adult	F								
	Extended Data Fg.1 and Table 5.6		Extended Data Fg.1 and Table 5.6	Wißing et al 2019: Fig 2	Fernandez-Jalvo and Andrews 2019	Fernandez-Jalvo and Andrews 2019				Supplementary information H1	
					Rougier et al 2016	Rougier et al 2016					

Late Neanderthals Meuse Basin											
			Sex	Isotopes $\delta^{34}S$	Archaeology	Taphonomy	Kin	Genetic cluster shared	AMH introgression	C14 calBP	Genetic data samples
Spy	Spy I	adult	M	catchment within Meuse basin	bodies but burial unlikely	evidence of fatal injury	no data	shared with Goyet	none	54.320 - 42,160	4
	Spy II	adult	no data								
	Spy VI	18 month child	no data		reworked context	no data					
Goyet	Q305-1	neonate	M	catchment outside Meuse basin	cannibalism	eaten uncooked, no evidence for roasting or boiling	unrelated	shared with Spy	none	45,000 - 41,000	15
	1424-3D	6.5-12.5 year adolescent	M								
	C5-1	adult/adolescent	?								
	Q305-4	adult	F								
	Q376-25	adult	F								
	Q55-4	adult	F								
	Q119-2	adult	F								
	GN1	adult	F								
	GN2	adult	F								
	Extended Data Fg.1 and Table 5.6		Extended Data Fg.1 and Table 5.6	Wißing et al 2019: Fig 2	Fernandez-Jalvo and Andrews 2019	Fernandez-Jalvo and Andrews 2019				Supplementary information H1	
					Rougier et al 2016	Rougier et al 2016					

Late Neanderthals Meuse Basin											
				Isotopes				Genetic cluster	AMH	C14	Genetic data
			Sex	$\delta^{34}S$	Archaeology	Taphonomy	Kin	shared	introgression	calBP	samples
Spy	Spy I	adult	M		bodies but burial unlikely	evidence of fatal injury					
	Spy II	adult	no data	catchment within Meuse basin			no data	shared with Goyet	none	54.320 - 42,160	4
	Spy VI	18 month child	no data		reworked context	no data					
Goyet	Q305-1	neonate	M								
	1424-3D	6.5-12.5 year adolescent	M								
	C5-1	adult/adolescent	?								
	Q305-4	adult	F								
	Q376-25	adult	F	catchment outside Meuse basin	cannibalism	eaten uncooked, no evidence for roasting or boiling	unrelated	shared with Spy	none	45,000 - 41,000	15
	Q55-4	adult	F								
	Q119-2	adult	F								
	GN1	adult	F								
	GN2	adult	F								
	Extended Data Fg.1 and Table 5.6		Extended Data Fg.1 and Table 5.6	Wißing et al 2019: Fig 2	Fernandez-Jalvo and Andrews 2019	Fernandez-Jalvo and Andrews 2019				Supplementary information H1	
					Rougier et al 2016	Rougier et al 2016					
